# Functional *D*- and *L*‑Naphthalenediimide-Peptides: Microwave-Driven Synthesis, Supramolecular Aggregation, and Multiphoton Fluorescence Lifetime Imaging Microscopy in Living Cells

**DOI:** 10.1021/acsbiomedchemau.5c00064

**Published:** 2025-07-18

**Authors:** Simone G. Giuffrida, David G. Calatayud, Fernando Cortezon-Tamarit, Haobo Ge, Vincenzo Mirabello, Dora-Maria Răsădean, Charareh Pourzand, Stanley W. Botchway, Pedro Estrela, G. Dan Pantoş, Ian M. Eggleston, Sofia I. Pascu

**Affiliations:** † Department of Chemistry, 1555 University of Bath, Claverton Down, Bath BA2 7AY, U.K.; ‡ Department of Inorganic Chemistry, Facultad de Ciencias, Universidad Autónoma de Madrid, Francisco Tomás y Valiente 7, 28049 Madrid, Spain; § Department of Life Sciences, University of Bath, Claverton Down, Bath BA2 7AY, U.K.; ∥ Centre for Therapeutic Innovation, University of Bath, Bath BA2 7AY, U.K.; ⊥ Central Laser Facility, Rutherford Appleton Laboratory, Research Complex at Harwell, STFC, Didcot OX11 0QX, U.K.; # Department of Electronic and Electrical Engineering, University of Bath, Claverton Down, Bath BA2 7AY, U.K.; ¶ Centre for Bioengineering & Biomedical Technologies (CBio), University of Bath, Claverton Down, Bath BA2 7AY, U.K.

**Keywords:** multiphoton time-correlated single photon, amino acid-tagged naphthalenediimides, gastrin-releasing peptide receptor, multiphoton fluorescence lifetime imaging, fluorescent peptide conjugates

## Abstract

We report the microwave-assisted synthesis of a novel family of peptide-linked optical imaging probes incorporating the *L*-[7,13] bombesin fragment (denoted *L*-[7,13]­BBN) as a functional building block currently used for targeting the gastrin-releasing peptide receptor (GRPR) in cancer cells. Given the importance of chirality in probe design, we synthesized and evaluated both *L*- and *D*-amino acid-substituted naphthalenediimide (NDI), namely, the monopeptide (*L*-**3**) and corresponding bis-peptide (*L*-**4**) conjugates. These bioconjugates were characterized using NMR, fluorescence spectroscopy, including excitation–emission mapping, and mass spectrometry, confirming their spectroscopic tunability, water solubility, and ability to form supramolecular aggregates. Aggregation behavior was demonstrated by scanning electron microscopy (SEM) and Time-Correlated Single-Photon Counting (TCSPC) spectroscopy, while circular dichroism studies revealed a stereochemistry-driven self-assembly influenced by 4-iodophenylalanine modifications. Additionally, a new, desymmetrized NDI-based bioconjugate (*L*-**6**), which incorporates the *L*-[7,13]­BBN fragment and a functional BODIPY fluorescent label, was synthesized in a stepwise manner via the microwave-assisted methods developed hereby. Cytotoxicity assays showed that these are benign, nontoxic probes at the time of imaging experiments and up to 72 h observation. Cellular uptake and localization properties of all compounds were assessed using confocal laser-scanning microscopy correlated with multiphoton fluorescence lifetime imaging microscopy (MP FLIM). This imaging method provided insights into the distinct behaviors of mono- vs bis-substituted peptide conjugates in live PC-3 prostate cancer cells, known to overexpress GRPR, and in A431 cells, known to overexpress the epidermal growth factor receptor (EGFR). Notably, the *L*- and *D*-stereochemistries of the BBN­[7,13] fragment played a crucial role in modulating the uptake and subcellular localization of bioconjugates of type 3 and 4 in lysosomes while the presence of the BODIPY unit additionally directed the biolocalization of compound *L*-**6** toward the endoplasmic reticulum of multiple cellular environments, including in living PC-3 and A431 cells. These findings are relevant for the design of new biologically active probes, including proteolysis-inactive, peptide conjugates for cancer biomarker detection and imaging.

## Graphical abstract

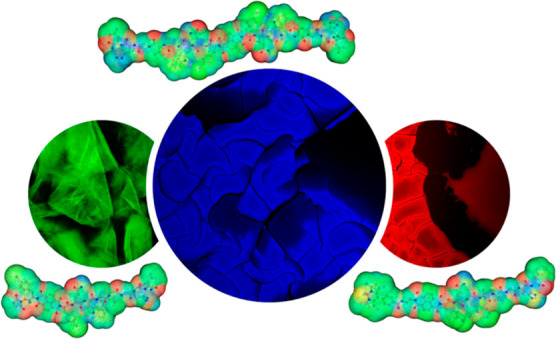

## Introduction

Biosensing, either electrochemically^1,2^ or optically,^3^ has recently become crucial for detecting cancer cells due to the increased demand for reliable, sensitive, selective, and low-cost diagnostic methods. Through coupling with bioimaging techniques such as fluorescence microscopies (e.g., confocal microscopy, multiphoton microscopy, and super-resolution microscopy),^4^ biosensing has evolved to become a pillar in studying biological processes and detecting cancerous cells or tissues.^5^ In this context, organic and metal complex-based small molecule imaging probes are attractive tools for biosensing as their ease of functionalization can provide the ability to be tuned in a wide spectral range, along with subsequent improvement of their features for biomedical use.^6^ However, although several such imaging probes have been proposed over the years, only a few of them have proved to be promising and effective for fluorescence bioimaging.

BODIPY (4,4-difluoro-4-bora-3a,4a-diaza-*s*-indacene) has been considered as one the most promising fluorescent probes for cellular uptake investigations.^6^ This molecule and its functional derivatives present strong UV–visible absorptions and intense fluorescence emissions associated with high quantum yields.^7^ Furthermore, these fluorescent probes seem to show stability under physiological conditions and biocompatibility. All these characteristics, combined with their biorthogonal ligation capabilities, have made these molecules efficient tags and scaffolds for potential *in vivo* imaging probes. Imaging probes capable of response through the two-photon excited emission ranges (approximately from 600 to 1350 nm) can show significant advantages over cellular imaging using one-photon excitation only.^8^ These advantages include decrease in tissue autofluorescence and light scattering in cells and tissue,^9^ while improved tissue depth penetration allows imaging selectivity and noninvasive confinement to the focal volume. We^12,13^ and others^14,15^ have highlighted various BODIPY-functionalized compounds in the context of the design and testing of new probes for multimodal imaging applications.^8,10,11^

Naphthalimides and the corresponding naphthalenediimides (NDIs) have been a focus of considerable research as probes for fluorescence imaging and biosensing.^10,11^ They are chemically inert, with resistance to significant photobleaching when exposed to excitation in the range 400–560 nm, and show a range of quantum yields depending on the nature of the peripheral functionalities involved.^16^ Their well-established two-photon cross sections (in the range of 0.5 to 1 · 10^–20^ cm^4^/GW)^17^ make them relevant for multiphoton excitation for fluorescence imaging applications in living cells.^18–20^ This family of molecules presents an extended, planar, and electron-deficient π-system that interacts through van der Waals and π–π stacking interactions with other aromatic substrates via their quadrupole moment. Electron-deficient NDIs can also interact with electron-rich species to form supramolecular donor–acceptor complexes and display self-assembling behavior on surfaces and thin films that are substituent-driven.^21^ The optical characteristics of NDIs associated with the expanded aromatic π-system can be tuned^22,23^ to shift absorption maxima and fluorescence emissions^16,24^ toward the useful range for general imaging. Moreover, the tunability of the optical features of NDIs is manifested even in their supramolecular aggregates, which can modify the absorption maxima and fluorescence emissions.^25–27^

The imide moieties of NDIs can be modified for different purposes, for example, to increase water solubility or targeting efficiency. As such, amino acid-tagged NDIs^28,29^ have been used in various biological applications including as constituents of biodegradable polymers,^30^ as binders for G-quadruplexes,^31^ or for the recognition of natural bioactive molecules.^32^ Recent reports^10,11^ have also demonstrated that the incorporation of amino acids in the NDI moiety provides systems with fluorescence emission detectable in living cells and also efficient uptake in cancer cells compared to healthy cells. These results have suggested that such scaffolds could be further derivatized with specific peptide fragments^10^ to provide selective imaging probes for cancer.

We^33^ and others^34–38^ have investigated the applications of targeting peptides for the cellular delivery of fluorescent molecules, of relevance to photodynamic therapy^34,35^ as well as multimodality bioimaging by optical imaging^36^ and positron emission tomography.^37,38^

In particular, bombesin (BBN), a natural 14-amino acid peptide, has been the subject of sustained interested as a tumor targeting agent due to its high affinity for a family of receptors that includes the gastrin-releasing peptide receptor (GRPR, BB2R), which is overexpressed in various forms of human cancer:^39,40^ molecular fragments of bombesin, particularly the [7,13] (QWAVGHL) and the [7,14] (QWAVGHLM) peptide sequences, have been linked with different kinds of scaffolds such as nanoparticles,^41–46^ folates,^47^ nanorods,^48^ macrocycles,^49^ and metal complexes^50^ and a range of bombesin-based compounds have demonstrated the potential for therapeutic^51,52^ and/or imaging applications.^37,53–55^

Furthermore, there is growing interest in *D*-peptide technology, particularly in the development of proteolytically stable *D*-peptide ligands for targeting clinically relevant receptors, which could serve as the foundation for novel fluorescent imaging probes.^56–58^ In this context, detailed spectroscopic studies of peptide-targeted probes in both enantiomeric forms at the cellular level can provide valuable insights into the receptor affinity of *D*-peptides compared to those of their *L*-peptide counterparts. Such studies may uncover unexpected and useful leads for targeting additional receptors, expanding the potential applications of *D*-peptide-based probes in biomedical research. Here, we report on our investigations into new fluorescent NDI bombesin peptide conjugates in which either *D*- or *L*-[7,13] bombesin peptide fragments are anchored onto the flat, aromatic, and rigid NDI fluorophore. The self-assembly properties of the bioconjugates derived from the presence of a specific chiral peptide and their intrinsic fluorescence emissive properties in solution, the solid state, and living cancer cells are described along with fluorescence lifetime measurements as an analytical tool for cellular environmental sensing. We aimed to present hereby a compelling and comprehensive study on the synthesis and characterization of novel peptide-linked optical imaging probes targeting the known gastrin-releasing peptide receptor (GRPR) in cancer cells by exploring the optical properties of the as-prepared biomaterials and unravelling their tunability, water solubility, self-assembly, and aggregation behavior in solutions and in thin film.

## Results and Discussion

### Synthesis and Characterization of New NDI–Peptide Bioconjugates

The synthetic pathway to obtain NDI-based *mono*- and *bis*-bombesin peptide variants is shown in Scheme 1. Pairs of enantiomeric *mono*- and *bis*-peptide derivatives (*L*- or *D*-**3** and *L*- or *D*-**4**, respectively) were synthesized and characterized spectroscopically, to probe the effect of stereochemistry upon absorption and interactions with the cancer cells. A heterofunctionalized NDI conjugate with a *L*-BBN­[7,13] peptide and a BODIPY moiety was also prepared. The design strategy for this compound **6** was to produce an alternative fluorescent NDI also targeted to receptors at the prostate cancer cell surface by virtue of its BBN­[7,13] fragment.

**Scheme 1:**
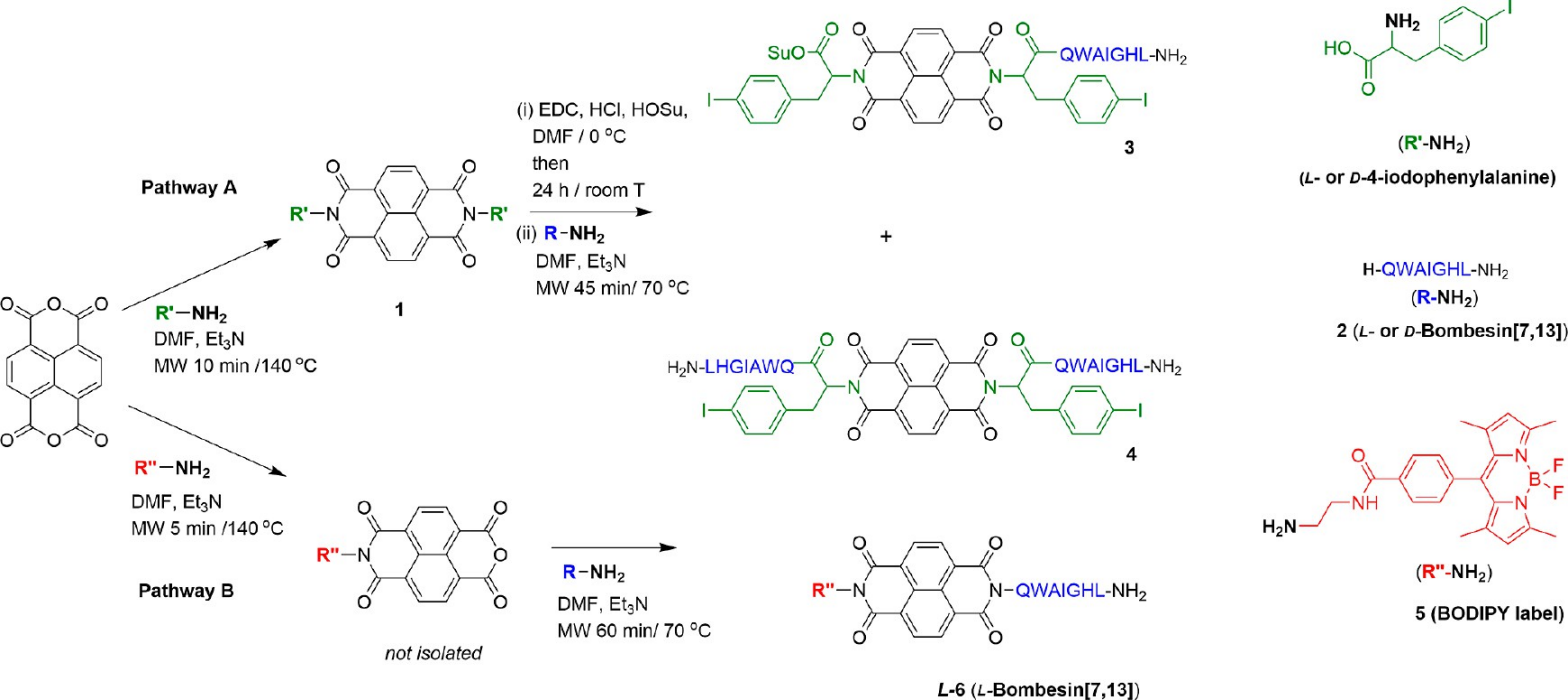
Microwave-Assisted Synthesis of the *mono*- and *bis*-Peptide and Amino Acid-Tagged NDI-Conjugates. Pathway A: Compounds 1-4, Whereby either *L*- or *D*-Peptide Forms were Isolated in Each Case; Pathway B: Compound **6**, where the de-symmetrized NDI is conjugated to BODIPY- and the *L*-[7,13]­BBN Peptide. Detailed Reaction Conditions are given in the Experimental Section and the Supporting Information

The first step in the preparation of the peptide-tagged compounds was to functionalize NDA with 4-iodophenylalanine amino acid linkers. These were introduced using a modification of a method for the desymmetrization of NDI with primary amines and amino acids. The reaction between naphthalene dianhydride and 4-iodo-*L*-phenylalanine or 4-iodo-*D*-phenylalanine (Figure 1, pathway A) led to the formation of the *L*- as well as the *D*-enantiomers of compound **1** denoted *L*-**1** or *D*-**1** (*L*-enantiomer or *D*-enantiomer, respectively). Direct desymmetrization and functionalization of the starting material (Figure 1, pathway B) was also achieved by our microwave-assisted approach using one equivalent of the BODIPY-ethylene diamine derivative **5**. Subsequently, the BODIPY-tagged NDI formed *in situ* (not isolated) was converted by direct microwave-assisted coupling to the bioconjugate 6, following treatment with one equivalent of the *L*-bombesin­[7,13] **2**. Compound **6** (incorporating simultaneously a functional BODIPY tag and *L*-bombesin­[7,13]) was also isolated by semipreparative HPLC and characterized by spectroscopic methods (*vide infra* and the Supporting Information).

**Figure 1:**
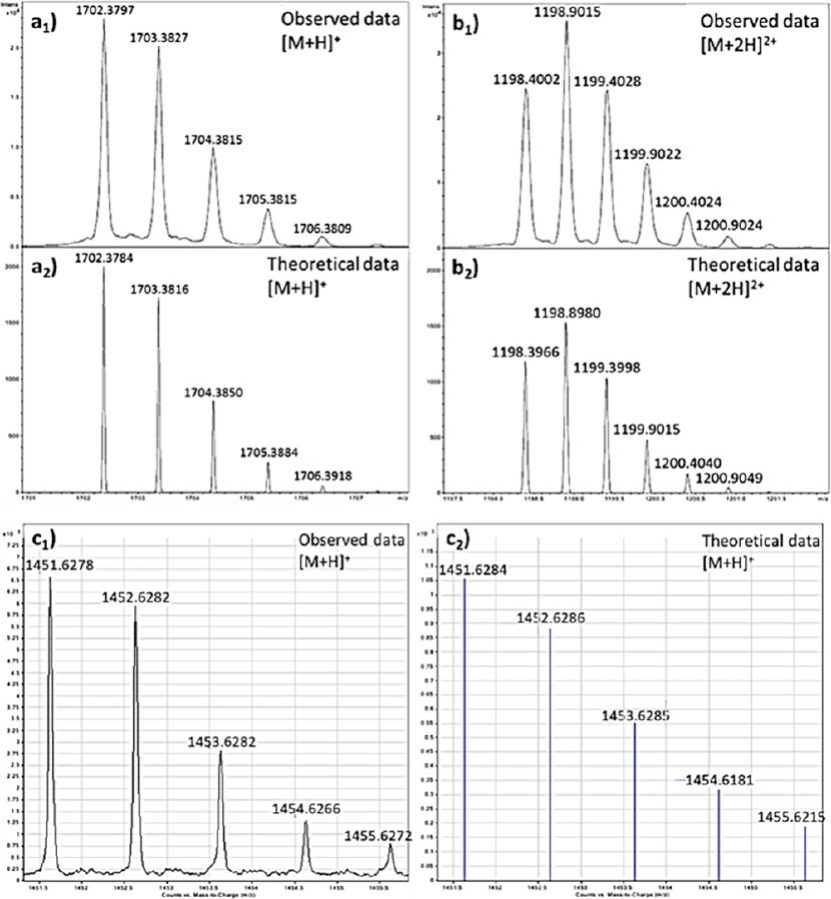
Mass spectrometry investigations: Positive ESI-TOF isotopic patterns of 3 (a_1,2_), 4 (b_1,2_), and 6 (c_1,2_). a_1_,b_1_,c_1_ observed isotopic patterns for the ions [M + H]^+^, [M + 2H]^2+^, and [M + H]^+^, respectively; a_2_,b_2_,c_2_ theoretical isotopic patterns for the ions [M + H]^+^, [M + 2H]^2+^, and [M + H]^+^, respectively.

Subsequently, to allow coupling of the peptide units of both enantiomeric forms of **1**, the carboxylic acid groups were activated by conversion to succinimidyl esters (EDC·HCl/HOSu).^10,11^ The bombesin [7,13] fragment peptides were obtained by automated solid-phase peptide synthesis using *L*- or *D*-amino acids (compounds *L*-**2** and *D*-**2**, respectively) and were then appended to the NDI scaffold. The coupling between the activated amino acid-NDI derivatives and the peptide of choice (*L*- or *D*-bombesin [7,13] fragment, compound **2**) was then performed with microwave activation, leading to the formation of two main products (**3** and **4**). The *L*- and *D*-forms of the *mono*- and *bis*-peptide conjugates (*L* **-3** and *D*-**3**, *L*-**4** and *D*-**4**, respectively) were isolated by extensive reverse phase semipreparative HPLC protocols and characterized by analytical HPLC (S50–S56) and solution spectroscopic methods in DMSO, including ^1^HNMR (Figures S32–S44) and DOSY (for the largest bis-peptide conjugate, system, *L*-**4**, Figure S44), UV–vis and fluorescence spectroscopies (see below and Figures S57–S63).

#### Investigations of New NDI-Peptide Conjugates by Mass Spectrometry

Compounds *L* **-3**, *L* **-4**, *D* **-3**, *D* **-4**, and *L*-**6**. were characterized by mass spectrometry using the positive ESI-TOF technique and Figure 1 reports the observed and theoretical isotopic patterns of compounds **3**, **4**, and **6**. Two main ions were found for *L* **-3**, which corresponded to the [M + H]^+^ and [M + Na]^+^ species, and their mass-to-charge ratios were consistent within ±5 mDa. An intense peak at around 1198 *m*/*z* was observed for *L* **-4**, which correlates to the [M + 2H]^2+^ species and is within ±5 mDa of the expected mass modeled from theoretical isotopic pattern. Similar observations were made for *D* **-3** and *D* **-4** (see Table 1). In the mass spectrometry analysis of *L*-**6**, the ion at around 1451 nm is within ±5 mDa from the simulated isotopic pattern for the expected [M + H]^+^ species. Table 1 summarizes the key found and calculated mass spectrometry data for *L* **-3**, *L* **-4**, *D* **-3**, *D* **-4**, and *L*-**6**, and more detailed information is given in the Supporting Information.

**Table 1:** Summary of Mass Spectrometry Analysis Data Obtained; Corresponding Isotopic Patterns Are Given in Figure 1 and the Supporting Information, Where Extensive Information on Mass Spectrometry Characterization and HPLC Details Are Depicted (Figures S7–S31)

compound	(ESI-MS) (found *m*/*z*)	calculated mass (Da)
*L* **-3**	[M + H]^+^ = 1702.3797	1701.3711
	[M + Na]^+^ = 1724.3672	1724.3609
*L* **-4**	[M + 2H]^2+^ = 1198.4002	1197.3893
	[M + H]^+^ = 2395.7806	2394.7786
*D* **-3**	[M + H]^+^ = 1702.3811	1701.3711
	[M + Na]^+^ = 1724.3710	1724.3609
*D* **-4**	[M + 2H]^2+^ = 1198.4050	1197.3893
	[M + H]^+^ = 2395.8557	2394.7786
		2394.7786
*L*-**6**	[M + H]^+^ = 1451.6278	1450.623

#### Circular Dichroism Investigations

The chirality-induced behavior of compounds *L* **-3**, *L* **-4**, *D* **-3**, and *D* **-4** was studied using circular dichroism (CD) in DMSO solutions (200 μM) and the resulting spectra are shown in Figure 2. Interestingly, the amino acid chirality has been transferred to the NDI chromophore as indicated by the Cotton effects observed at 380.5 nm. This highlights the successful synthesis of enantiomeric pairs **3** and **4**. The expected contributions of the tryptophan residues of the BBN[7–13] peptides at around 280 nm were not observed in the CD spectra due to their overlap with the absorption of DMSO in that region. We also analyzed the ellipticity of the compounds at variable temperatures from 25 up to 70 °C (with details given Figures S46–S49) to determine if chirality and thus the conformation of the bioconjugates are affected by temperature. There were no substantial changes in the range of temperatures studied, which may suggest that the *L* vs. *D* chirality of *L* **-3**, *L* **-4**, *D* **-3**, and *D* **-4** can be clearly distinguished, and the conformation of these species remained unchanged between 25 and 70 °C.

**Figure 2:**
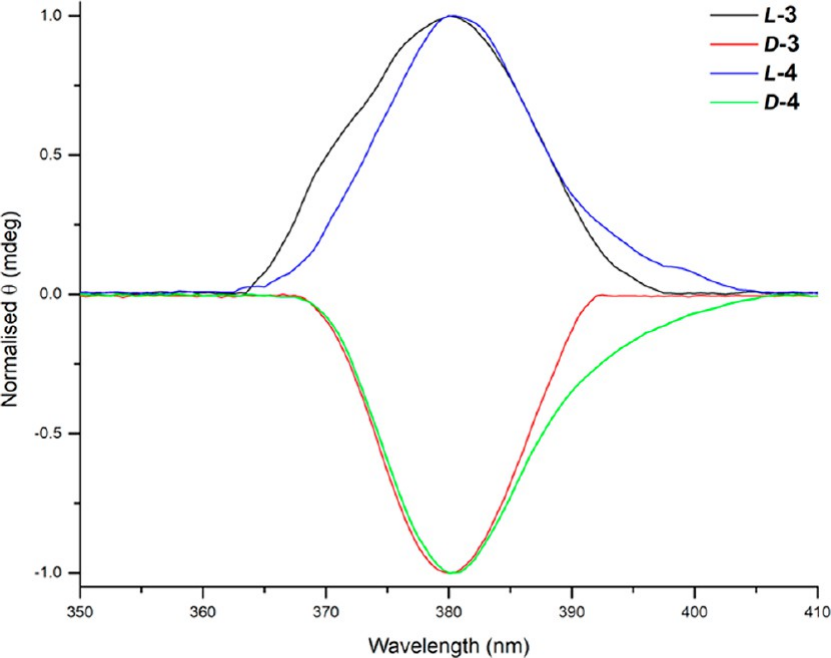
Circular dichroism spectroscopy of compounds *L* **-3** (blue), *D* **-3** (yellow), *L* **-4** (red), and *D* **-4** (green), recorded in DMSO solutions (200 μM), recorded at the room temperature. Variable temperature experiments are included in the Supporting Information.

#### UV–Visible and Fluorescence Spectroscopies

The photophysical properties in solution of all of the NDI–peptide conjugates were further assessed by UV–visible and fluorescence spectroscopies explored in terms of excitation–emission maps (EEM) given in Figures 3 and S57–S63 and Tables S1–S4). The UV–visible profiles of **3** (both enantiomers) showed three λ_max_ bands at 280, 360, and 380 nm. The first two absorption bands are assignable to *n*–π* and π–π* transitions of the aromatic rings of tryptophan and histidine present on the bombesin peptide fragment. On the other hand, the two absorption bands with maxima at 360 and 380 nm derive from the π–π* electronic transition of the aromatic core of the NDI. The same features were found for both enantiomers of **4**. Furthermore, it was noticed that the intensity of the bands at around 280 nm is significantly enhanced compared to the spectra of compounds type which is consistent with the presence of an additional peptide moiety in compounds type for both *L*- and *D*-enantiomers.

**Figure 3:**
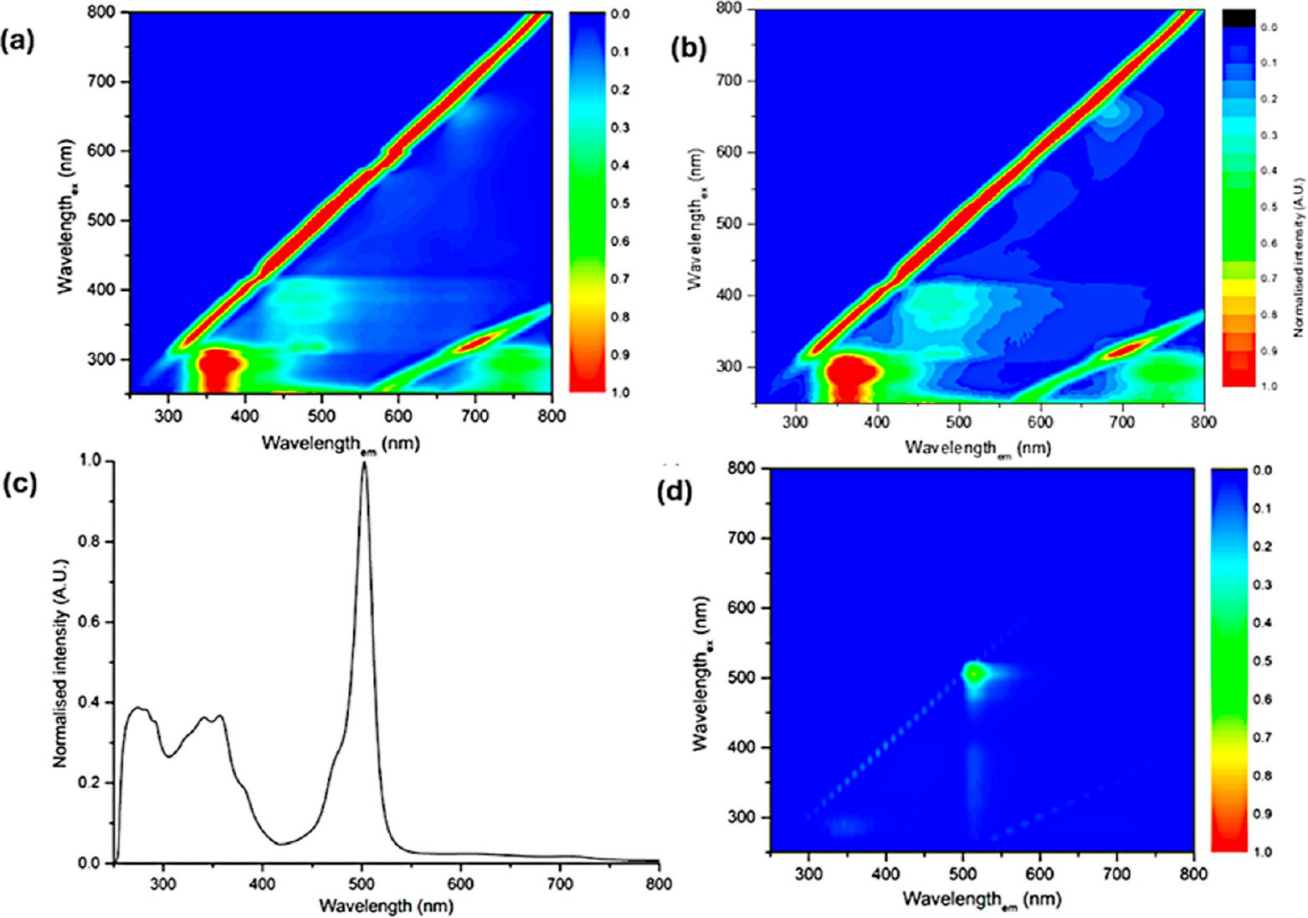
Comparison of excitation–emission maps (EEM) of *D* ***-*3** (a) and (b) *D* **-4** (both at 200 μM in DMSO). The corresponding EEMs of *L* **-3** and *D* **-3** show bands in the same regions of the spectrum and are given in the Supporting Information. (c) UV–visible spectrum and (d) excitation–emission map of *L*-**6** (50 μM in DMSO).

The UV–visible spectrum of compound *L*-**6** showed four different absorption maxima at ca. 280, 360, 380, and 500 nm. The band around 280 nm may attribute to the bombesin peptide fragment, while the peaks at 360 and 380 nm are characteristic of interacting, stacked NDI cores. The intense maximum at 500 nm, is the characteristic π–π* transitions of the BODIPY moiety (Figure 3). Fluorescence excitation–emission matrix (EEM) maps indicated that both enantiomers of **3** emit in the range between 300 and 500 nm when excited between 250 and 300 nm. Additionally to another emission range between 400 and 500 nm, at the excitation wavelengths in the range of 350–400 nm was also observed. The enantiomers of **4** show weak fluorescence emissions in the same ranges as their *mono*-peptide versions. As expected for the case of *L* **-6**, the fluorescence emission is dominated by the BODIPY moiety,^60^ with emission between 500 and 560 nm upon excitation ranges between 470 and 520 nm, analogous to compound **5**, a typical BODIPY. The excitation–emission maps indicate the presence of emissions at 700–800 nm for **3** and **4**, upon excitations between 200 and 320 nm, indicative of the occurrence of excimers in the solution phase 200 μM in DMSO.

#### Aggregation and MM+ Modeling of the Supramolecular Peptide Stacks

We investigated the potential for supramolecular aggregation of the NDI–peptide derivatives to better understand the nature of the aggregation observed both in the solid state and in solution. Molecular mechanics calculations were performed to estimate the most stable aggregation states and conformations of these molecules using a MMFF94 Force Field in the solid state and water solution states. All derivatives exhibited a tendency to aggregate due to intermolecular π–π stacking interactions between the aromatic rings of the NDI core (Figure 4). These stacking interactions bring potential donor and acceptor NDI units into proximity, facilitating photoinduced electron transfer (PET), which explains the observed reduction in fluorescence lifetimes. As shown in Figure 4c, the BODIPY-containing compound *L* **-6** displayed the shortest intermolecular distance (3.475 Å), followed by *L* **-3** (3.665 Å), and finally *L* **-4** (3.796 Å), whereas an identical behavior was predicted for *L* **-3** and *L* **-4**.

**Figure 4:**
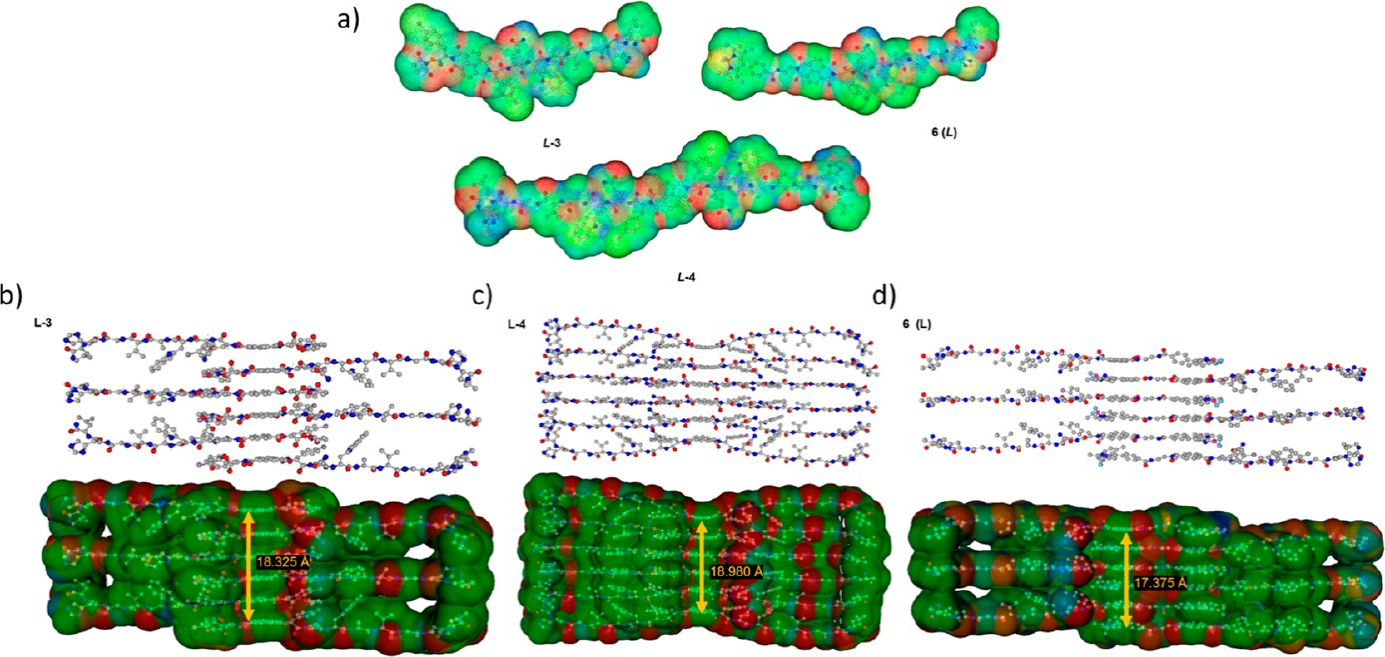
(a) Computationally calculated structures (MM+) and solvent accessible surfaces for *L* **-3**, *L* **-4**, and *L*-**6** (colors represent electrostatic potential). (b–d) Qualitative representations of stacks of these molecular dynamics calculated structures, showing postulated aggregated fragments and solvent-accessible surfaces in the three-dimensional packing of: (b) *L* **-3**, (c) *L* **-4**, and (d) *L*-**6**. The xyz coordinates for MM + optimized species of types **3**, **4**, and **6** are given in the Supporting Information.

To further investigate the aggregation behavior, we examined the surface morphologies of the isolated conjugates in the solid state at the micrometer scale using field emission scanning electron microscopy (FE-SEM). This analysis aimed to determine whether the aggregation properties of these peptide-based compounds influence their fluorescence emission. The FE SEM micrographs of the *D*- and *L*-enantiomers of **3** and **4** were recorded from dried powder after purification or after freeze-drying of 1 mg/mL solutions deposited in either MeCN/H_2_O (1:1) or DMSO solutions. Figure 5 depicts the typical micrographs that indicate that these conjugates preferentially aggregate into coil- or needle-shaped assemblies, phenomena likely driven by the expected intermolecular π–π stacking of the NDI core. Additionally, weak intra- and intermolecular interactions between the appended functional peptides may contribute to the observed aggregated morphologies in line with previously observed complex behavior of this peptide fragment in solution phase.^59^ Confocal laser scanning fluorescence microscopy was performed for the thin films deposited on glass-bottomed borosilicate slides using imaging Petri dishes. Micrographs were acquired using Airyscan detection for *L* **-3** (a1–e1), *L* **-4** (a2–e2), *D* **-3** (a3–e3), *D* **-4** (a4–d4), and *L* **-6** (a5–d5) where these images were recorded following overnight freeze-drying of films deposited from DMSO (Figures 6 and S67). Interestingly, for *L* **-3** and *D* **-3** imaged in dried thin-film conditions, the maximum emission was seen on the blue (λ_ex_ = 405 nm, λ_em_ = 420–480 nm) and green (λ_ex_ = 488 nm, λ_em_ = 495–550 nm) channels mainly for larger, globular aggregates and with only weak emission being visible for the thinner fiber-like entities and across drying lines in the red emission (λ_ex_ = 561 nm, λ_em_ = 555–620 nm), whereas for the rest of the compounds (*L*-**4** and *D*-**4**) in the series, there appears to be a broad emission observable in all channels. This is consistent with our observations from solution excitation–emission maps (Figures 3 and S57–S63) and further points to the overall ability of these aggregates to show fluorescence emission due to possible stacking and excited-state electron and energy dynamics. Confocal fluorescence microscopy of these thin films confirmed emission in the expected wavelength ranges, as observed in the fluorescence excitation–emission maps (EEM, given in Figure 3 and the Supporting Information). Specifically, emission was detected in the blue (λ_em_ = 420–480 nm), green (λ_em_ = 495–550 nm), and red (λ_em_ = 555–620 nm) channels. These Airyscan confocal micrographs also revealed that the molecules tend to aggregate into distinct sheet-like and globular morphologies, consistent with the surface aggregation patterns observed via field-emission scanning electron microscopy (FE SEM) (Figures 5 and S64–S66). For example, *L* **-4** exhibits a granular and globular morphology at the confocal imaging scale (Figure 6a­(2)–e­(2)), a feature that is also evident at the micrometer scale in FE SEM images (Figure 5c,d, and the Supporting Information).

**Figure 5:**
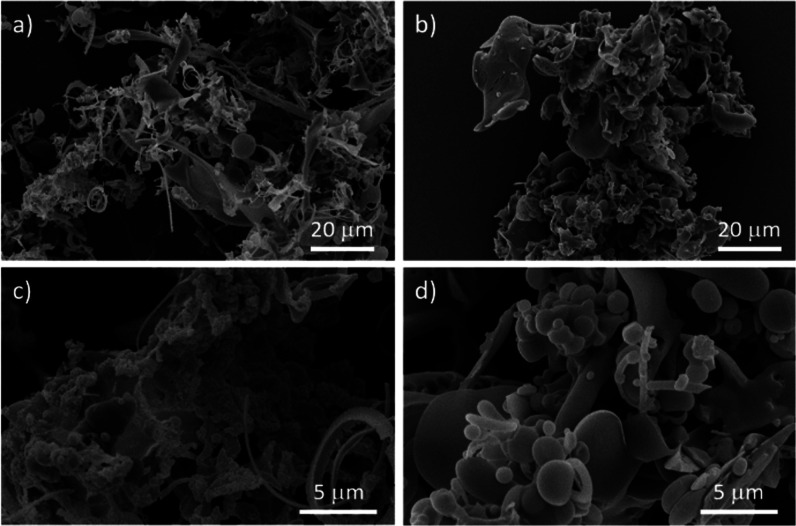
Supramolecular aggregation of mono- vs. double-substituted NDI–peptide conjugates: FE SEM micrographs of compounds *L* **-3** ((a,b)) and *L* **-4** ((c,d)) in dried thin films prepared after semiprep HPLC separation. Additional micrographs for thin films of *L* **-3**, *D* **-3**, *L* **-4**, and *D* **-4**, deposited from 1:1 H_2_O/MeCN or DMSO followed by freeze-drying overnight are given in the Supporting Information.

**Figure 6:**
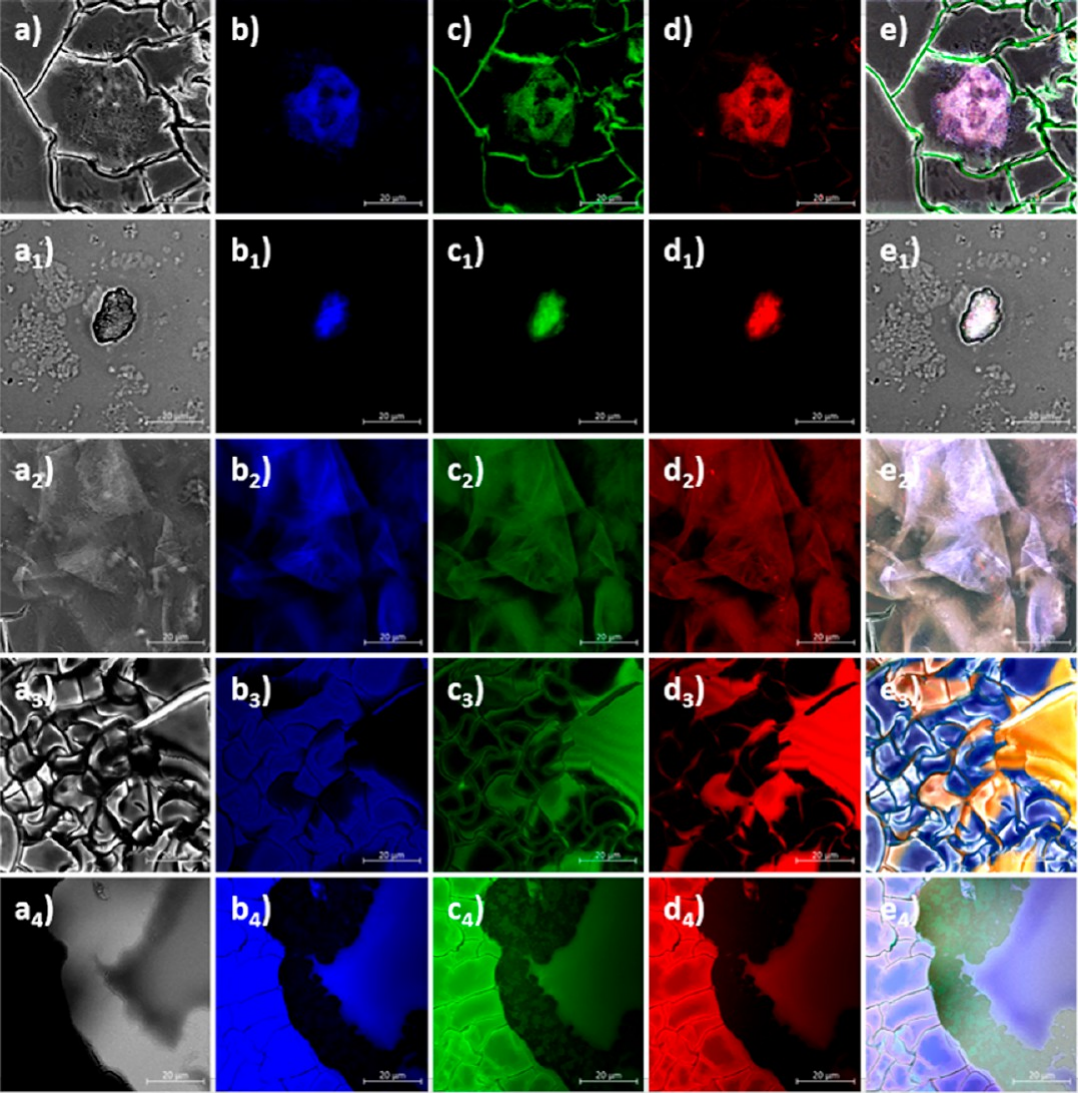
Supramolecular aggregation behavior imaged in thin films by confocal laser-scanning microscopy with an Airyscan detector. Dried films were obtained after the drop-casting of DMSO solutions of compounds onto borosilicate glass-bottomed Petri dishes suitable for imaging, followed by freeze-drying. a1–e1 *L* **-3**, a2–e2 *L* **-4**, a3–e3 *D* **-3**, a4–e4 *D* **-4**, and a5–e5 *L*-**6**. a1–5 Bright field; b1–5 blue channel: λ_ex_ = 405 nm, λ_em_ = 420–480 nm; c1–5 green channel: λ_ex_ = 488 nm, λ_em_ = 495–550 nm; d1–5 red channel: λ_ex_ = 561 nm, λ_em_ = 555–620 nm; e1–5 overlay of blue, green, and red channels. Scale bar: 20 μm.

#### Multiphoton Fluorescence Spectroscopy of *D*- vs *L*-Peptide Bioconjugates in Solution Phase

The fluorescence emission lifetime decays of the compounds in solution and in a cellular environment were investigated by time-correlated single photon counting (TCSPC) and fluorescence lifetime imaging microscopy (FLIM). These advanced spectroscopies were chosen due to the several advantages they offer over steady-state techniques. FLIM has been developed as a powerful tool to investigate biological processes such as cellular metabolism,^69^ biological O_2_ sensing,^70^ cellular uptake and distribution of species in living cells.^71^ A key point for the wide use of FLIM in biological science is that such measurements are independent of the species concentration, fluorescence excitation, and photobleaching. In addition, multiphoton or nonlinear FLIM uses near-infrared (NIR) wavelengths (700–1700 nm), which can penetrate cells and tissues to a greater depth and avoid significant phototoxicity that is observed with one-photon absorption in most cells.^61^

Prior to the cellular studies by FLIM, lifetime distribution decays of probes **3** (both enantiomers), **4** (both enantiomers), and *L* **-6** were studied by TCSPC in different concentrations and solvent systems. Figure 7 shows the lifetime decays in DMSO solutions at 0.01, 0.1, and 1 mM (a_1–3_) and in 1:1 DMSO/H_2_O mixture at 0.025, 0.25, and 2.5 mM (b_1–3_) of the fluorescent probes, accompanied by the observed general trends (Figures 7 and 8). The main fluorescence lifetime parameters obtained by TCSPC in the solution phase are summarized in Table 2, and further details are given in Figures S105–S133 and Tables S2–S4. The goodness of fit of the data points obtained is represented by the Chi–square parameter, χ^2^; τ_1_ is the short-lifetime component; τ_2_ is the long-lifetime component; and the parameters a_1_ and a_2_ represent the amplitude of the components.

**Figure 7:**
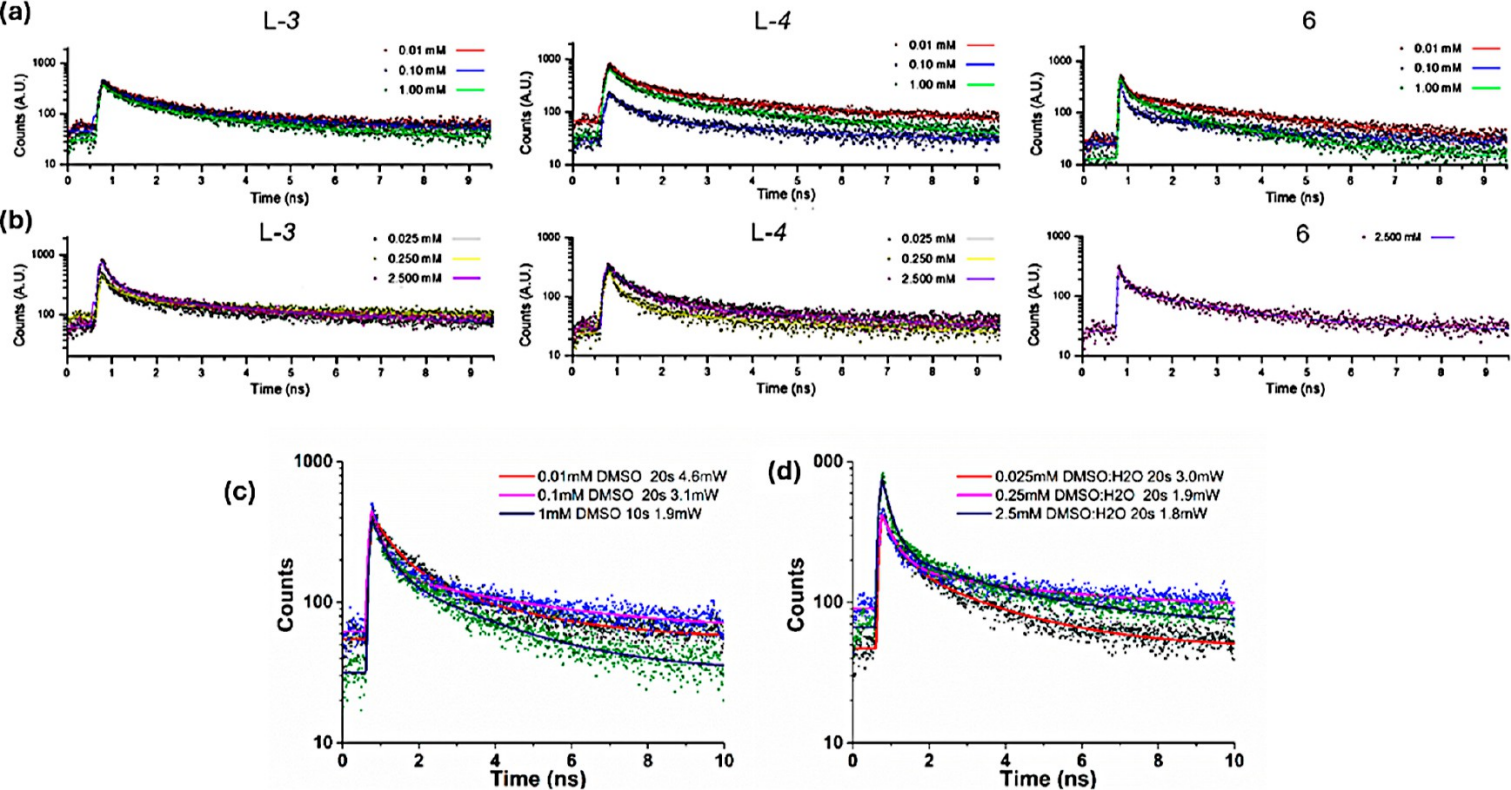
Two-photon excited point decays (TCSPC) for (a) *L* **-3**, *L*-**4**, and *L*-**6** (λ_ex_ = 910 nm) in DMSO; (b) *L* **-3**, *L* **-4**, and *L* **-6** in DMSO/H_2_O (1:1). (λ_ex_ = 910 nm). Two-photon excited point decay curves for *L* ***-*3** at 810 nm, within a range of concentrations, were obtained in DMSO (c) and DMSO/H_2_O (d).

**Figure 8:**
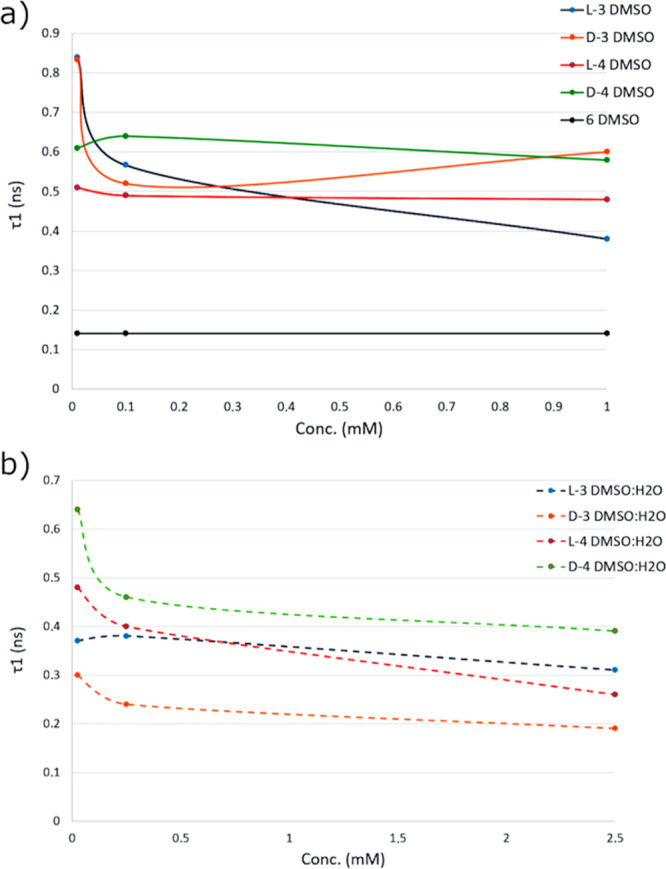
Schematic overview of the general trends observed for changes in 2-Photon lifetimes in solution, influenced by the aggregation behavior. The principal fluorescence lifetime components τ_1_ vs. concentration shows the effects of aggregation on the inhomogeneity of fluorescence lifetime in *D*- vs. *L*-peptides and corresponding mono- vs. double-substituted (*D*- vs. *L*-) NDI–peptide conjugates.

**Table 2:** Summary of the Parameters Obtained by TCSPC Analysis in Both Solvent Systems for All Four Compounds (910 nm)^a^

	DMSO						DMSO/H_2_O (1:1)					
molecule	Conc. (mM)	χ^2^	τ_1_ (ns)	a_1_ (%)	τ_2_ (ns)	a_2_ (%)	Conc. (mM)	χ^2^	τ_1_ (ns)	a_1_ (%)	τ_2_ (ns)	a_2_ (%)
*L* **-3**	0.01	1.04	0.84	62	7.35	38	0.025	1.23	0.37	82	10.01	18
	0,1	1.06	0.57	62	2.58	38	0.25	1.15	0.38	81	9.87	19
	1	1.15	0.38	63	2.54	37	2.5	1.27	0.31	77	3.45	23
*D* **-3**	0.01	1.06	0.83	73	4.3	27	0.025	1.20	0.30	73	3.27	27
	0.1	1.17	0.52	74	4.3	26	0.25	1.11	0.24	84	3.30	16
	1	1.19	0.60	80	6.6	20	2.5	1.32	0.19	86	3.73	14
*L* **-4**	0.01	1.74	0.51	75	4.76	25	0.025	1.15	0.48	84	9.57	16
	0.1	1.04	0.49	79	5.45	21	0.25	1.23	0.40	83	3.12	17
	1	1.44	0.48	77	4.29	23	2.5	1.16	0.26	86	4.97	14
*D* **-4**	0.01	1.05	0.61	83	7.55	17	0.025	1.05	0.64	64	13.15	36
	0.1	1.16	0.64	78	5.98	22	0.25	1.35	0.46	76	6.30	24
	1	1.10	0.58	75	4.77	25	2.5	1.18	0.39	82	3.28	18
*L*-**6**	0.01	1.21	0.14	68	3.34	32	2.5	1.04	0.15	65	2.25	35
	0.1	1.21	0.14	80	2.64	20						
	1	1.08	0.14	66	1.67	34						

a Note that discrepancies observed between *L*- versus *D*-fluorescence lifetime values are within instrument response, and any deviations above the expected 5–10% differences were observed at the higher concentrations, assignable to aggregate formation.

The lifetime decays of all the probes can be fitted by a double-exponential model with a χ^2^ close to 1, independent of the solvent used: this could be due to the aggregation behavior observed by SEM as noted above, consistent with earlier reports of supramolecular self-organization and nanotubular aggregate formation with simpler NDI-amino acid derivatives. This in turn may well generate the presence of oligomers of different sizes/shapes in solution, ultimately giving rise to real degenerate energy states observable by TCSPC.

The short-lived component (τ_1_) of *L* **-3** decreases as concentration in DMSO (a_1_) decreases because of changing aggregation behaviors with dilution and this behavior was also observed in *D* **-3** (a_2_). On the other hand, the τ_1_ remained approximatively the same in DMSO/H_2_O mixtures (b_1_ and b_2_). This data are comparable to the decays obtained for the NDI precursors, shown in the Supporting Information.

The first lifetime components of both enantiomers of **4** in DMSO (a_3_ and a_4_) remain unchanged as the concentration increases. In the mixture of 1:1 DMSO and H_2_O (b_3_ and b_4_), the values of τ_1_ decrease as concentrations rise. In this case, the lifetimes of the enantiomers of **4** can be attributed to the amphiphilic character expected to occur due to the presence of two moieties of the relatively hydrophilic [7,13]­BBN peptide linked to the NDI-core, the data for which are shown in the Supporting Information. Compound *L*-**6** shows similar characteristics, i.e., an unexpected decrease in lifetime with decreasing concentration in both solvent systems, and which we assign to aggregation behavior, mediated by the aromatic stacking of the NDIs combined with the hydrophobic/hydrophobic heterogeneous character of the conjugate and the aggregation behavior within these complex systems. In particular, the values of τ_1_ are approximatively 0.1 ns in both DMSO (a_5_) and DMSO/H_2_O (b_5_) solutions. Comparing these data with those of the BODIPY-linker precursor, compound **5** (data shown in Tables S2 and S3 and Figure S112), the τ_1_ values obtained for compound *L*-**6** are 10-fold lower than those for **5**. This is assignable to the self-aggregation of these peptide–NDI conjugates in solution,^62–73^ also as predicted hereby based on the SEM investigations of NDI–peptides in this family, discussed above.

According to the TCSPC results, backed by aggregation modeling, it seems that as the concentrations are diluted, the lifetimes reach higher values which we postulate might be due to the effect of the solvent molecules on the stackings is greater, weakening them and reducing the photo electron transfer (PET) effect that quenches the fluorescence (Tables 2, S3, and S4 and Figure 8). The trend of τ_1_ with respect to concentration and solvent reveals that generally similar trends are observed for *L* **-4** and *D* **-4** as well as for *L* **-3** and *D* **-3**. In general, diluted samples show higher fluorescence, likely due to the reduced presence of aggregation. As the concentration increases, π–π stacking effects become more pronounced, leading to decreased fluorescence. This is of relevance in the case of *L* **-3** and *D* **-3** compounds in the absence of water where it is seen that slightly increasing the concentration favors the aggregation by π–π interactions (as suggested by molecular mechanics modeling), which reduces the half-life times likely due to a photoelectron transfer effect (PET). In the case of compounds *L* **-4** and *D* **-4**, they present a smaller intermolecular interaction, as can be seen from the calculated intermolecular distance, so that the solvent does not seem to affect the lifetimes excessively; however for compounds *L* **-3** and *D* **-3** the presence of water produces a negative effect (shorter intermolecular distance). As can be seen, as the concentration increases, the lifetimes do not show large variations, since the interactions with the solvent molecules are minimized as there is a greater presence of molecules of the compounds and therefore an aggregation-induced fluorescence effect is produced. The observed trend with respect to the concentrations reaches a plateau where the concentration no longer impacts the fluorescence lifetime, supporting the hypothesis of aggregation-induced fluorescence. At low concentrations, an equilibrium exists between aggregated and nonaggregated states. As the concentration increases, this equilibrium shifts toward greater aggregation until a plateau is reached, consistent with previously reported observations in supramolecular aggregation behavior. The lifetime of compound *L*-**6** shows the lowest lifetime value, probably due to an energy transfer effect between BODIPY and NDI and its higher aggregation tendency mediated by donor-acceptor interactions. Emission lifetime values obtained in dilute solutions and associated trends suggest that the stacking effect does not significantly influence the fluorescence emission behavior in this compound. The occurrence of FRET is not surprising (Tables S2–S4) as the NDI core is well-known for its acceptor behavior, whereas the BODIPY tag can act as a donor unit, which may cause further aggregation in concentrated solutions and in thin film.

### Cellular Uptake in Living Cancer Cells

Compounds *L* **-3**, *L* **-4**, and *L* **-6** were tested for cytotoxicity against cancerous cell lines (PC-3), which showed that they are nontoxic at concentrations used for imaging and over 72 h of observations. The IC_50_ values were determined to be > 100 μM using the MTT and crystal violet cell viability assays (Figures S101–S102). This observation, taken together with spectroscopic characteristics described above for the bioconjugates, was used as a springboard for *in vitro* imaging assays. To visualize the uptake and localization of the compounds in a cellular environment, they were first added to living cancer cells and incubated under physiological conditions (see the Supporting Information). Imaging assays provided insights into the distinct behaviors of mono- vs. bis-substituted peptide conjugates in live PC-3 prostate cancer cells (known to overexpress GRPR) and in A431 cells, known to overexpress the epidermal growth factor receptor (EGFR). Cellular uptake and localization properties of all compounds were additionally assessed using confocal laser-scanning microscopy in a range of incubation conditions and living cells both GRPR-expressing (PC-3, LnCaP, MCF-7) and GRPR-negative (A431 and CHO). For the cell lines PC-3 and A431, these were also correlated with multiphoton fluorescence lifetime imaging (MP FLIM, *vide infra*). Both enantiomers of **3** and **4** were compared to the fluorescence emission of PC-3 cells incubated for 24 h with DMSO in serum free-medium, used as a control: from the micrograph of the control, cellular autofluorescence effects were minimized under the imaging parameters used (see the Supporting Information). As observed in the micrographs shown in Figure 9, compound *L* **-3** (micrographs a_1_-d_1_) mainly emits in the green and red channels of the excitation wavelengths of the laser of the confocal microscope, i.e., 488 nm. Compound *L* **-4** (micrographs a_2_-d_2_) showed a more intense emission in the green channel vs the red channel and the overall emission intensities in those channels appeared to be stronger than for the case of the corresponding NDI–peptide monoconjugate.

**Figure 9:**
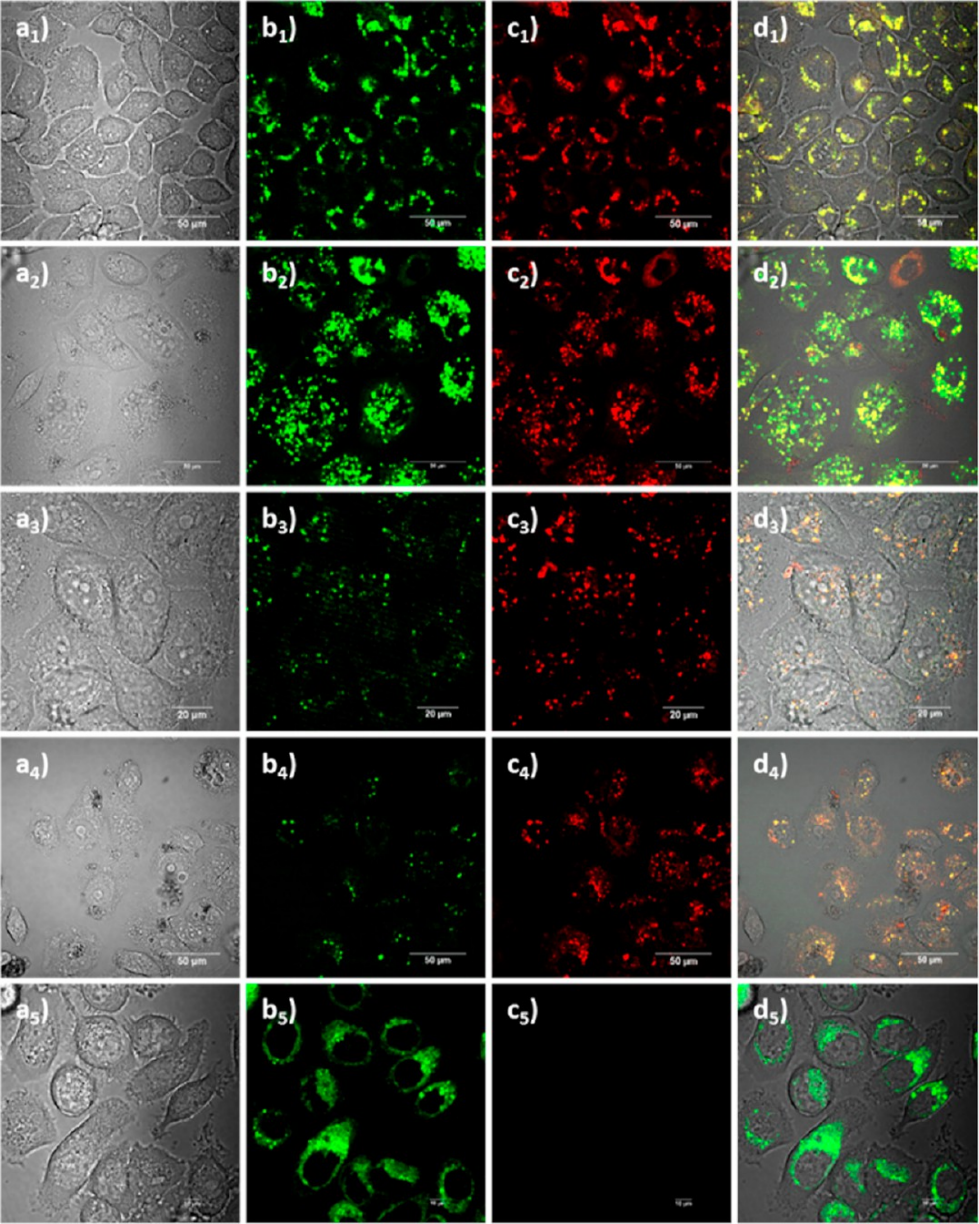
Confocal laser-scanning microscopy of PC-3 cells incubated at 37 °C with (a_1_-d_4_) solutions of 100 μM, in 1:99 DMSO:serum-free medium, over 24 h, as follows: (a_1_–d_1_) *L* **-3**, (a_2_–d_2_) *L* **-4**, (a_3_–d_3_) *D* **-3**, (a_4_–d_4_) *D* **-4**; and (a_5_–d_5_) a solution of 50 μM in 1:99 DMSO:serum-free medium of *L* **-6**, imaged after 20 min incubation. (a_1–5_) bright field channel; (b_1–5_) green channel (λ_em_ = 500–550 nm); (c_1–5_) red channel (λ_em_ = 570–750 nm); (d_1–5_) overlay of TD-green-red channels. λ_ex_ = 488.0 nm. A range of micrographs depicting confocal fluorescence microscopy experiments recorded under similar conditions in living A431 cells are also given in the Supporting Information.

To explore living cell interactions, we assessed the uptake and lysosomal localization of bioconjugates using confocal laser scanning microscopy coupled with multiphoton fluorescence lifetime imaging (MP FLIM, see below). An extensive range of micrographs depicting confocal fluorescence microscopy experiments recorded under similar conditions in living cells are given in Figures S58–S100. Experiments in live prostate cancer cell lines (PC-3 and additionally LnCaP) and epidermoid carcinoma cells (A431) demonstrated that the *L*- vs. *D*-stereochemistry of the bombesin fragment influences the uptake efficiency and subcellular distribution. We postulate that the presence of two *L*-[7,13]­BBN peptide moieties may improve the affinity with the cellular receptors^55,61,74^ and thus the *bis*-peptide compound *L* **-4** may be more efficiently absorbed than *L* **-3**. In addition, these results suggest that these fluorescent probes manifest different ranges in terms of fluorescence emission in an *in vitro* cell culture compared to in solution. In fact, the solutions in DMSO of *L* **-3** and *L* **-4** show weak fluorescence emissions in the range of the green channel (500–550 nm) when excited at 488 nm (see the Supporting Information).

Additionally, the uptakes of *L* **-3** and *L* **-4** were compared to their enantiomers *D* **-3** (a_3_–a_3_) and *D* **-4** (a_4_–a_4_), using the same techniques, to verify the effect of the stereochemistry on the cellular uptake and fluorescence properties. All four compounds were incubated under a range of conditions, including up to 24 h in PC-3 cells at 100 μM. Compounds *D* **-3** and *D* **-4** showed less cellular uptake than their enantiomers, which is consistent with a specific receptor-targeted uptake with the natural *L*-peptide derivatives.^61,62,75,65^ In addition, *D* **-3** and *D* **-4** showed intense fluorescence emission in the red channel. This was unexpected based on the fluorescence emission in solution, which is rather weak in the range between 570 and 750 nm upon excitation at 480 nm and assignable to aggregation and excimer formation within cytoplasmic cellular compartments.^66–68^ The cellular localization of compounds *L* **-3** and *L* **-4** was established using organelle-specific staining (see the Supporting Information). The results (complete data in the Supporting Information) revealed that *L* **-3** and *L* **-4** localize more in the lysosomes of PC-3 cells than in the nucleus or cytosol of the cells. This result suggests that these compounds could find application as novel agents to target lysosomes in cancer cells.^68,76^

Compound *L* **-6** emits in the green and red channels (500–550 and 570–750 nm, respectively) when excited at 488 nm. Regarding the uptake assays of compound *L* **-6** (Figure 9(a_5_–d_5_)), its fluorescence emission in PC-3 cells was visible after 20 min of incubation. This may suggest that the compound is taken up faster than the other 4 compounds and its fluorescence emission is due mainly to the BODIPY moiety, which showed the expected and well-characterized endoplasmic reticulum (ER) localization additionally to the membrane aggregation and lysosomal uptake. In addition, the fluorescence emissions *in vitro* are stronger than the observed fluorescence emission in solution. Compound *L* **-6** showed the most intense fluorescence emission in the series, which was observed in confocal fluorescence as localized in a dotted pattern partly on cell membrane and inside the cytoplasm, with clear indications of uptake within endoplasmic reticulum (ER), as expected for Bodipy-containing compounds,^12^ indicative of a rather complex uptake and localization process. This also supports the targeted uptake directed by the GRP receptors in PC-3 cells interplayed with BODIPY-driven cellular localization. To further probe this observation, a blocking experiment by confocal imaging was conducted (Figures S103–S104), whereby PC-3 cells were first incubated in the presence of a high concentration of *L*-[7–13]­BBN (1 mM) at 37 °C for 20 min. This peptide is only weakly fluorescent, and on this time scale (and up to 18 h of incubation time), confocal imaging cannot show distinguishable emission, to describe the uptake for *L*-[7,13]­BBN alone by this method (Figure S87). After the standard 20 min pretreatment time, the cells were washed with PBS and then the serum-free medium (SFM) was replaced. Immediately afterward, the PC-3 cells were incubated with conjugate *L* **-6** for an additional 20 min and imaged after washing off the excess of compound. The confocal fluorescence images acquired showed the biodistribution of *L* **-6** in the cytoplasm dispersed throughout the cytoplasm but to a significantly lesser extent than in the nonblocked experiment, with only the occasional presence of a punctuated distribution, remarkably different to the uptake observed for *L*-**6** alone. This result would point at a nonspecific uptake behavior under the evaluated conditions, which is driven not only by the targeting peptide (thus showing the GRP receptor recognition and a targeted behavior) but also by the BODIPY fluorophore (that shows a distribution in the cytoplasm known to be typically in the ER). In the case of the BODIPY-based intermediate fluorophore **5**, it has been observed that the maximum fluorescence intensity locations are observed inside the cellular cytoplasm, pointing consistently at ER biolocalization. These results correlate well with the observations made by single photon confocal microscopy of *L* **-6** in living cells and emphasize the role of *L*-[7,13]­BBN in targeting PC-3 cells: stated above, the cells incubated with *L*-**6** alone showed entirely different behavior in cells, which was mediated by the presence of BODIPY. In the case of L-6, the observed maximum fluorescence intensity is distributed over the exterior of the cell, with points of aggregation visible around the membrane.

### Multiphoton Fluorescence Lifetime Investigations

The fluorescence lifetime behavior in solution was assessed by TCSPC of single point decays whereas the distribution and localization of the probes in living cells was followed by extensive TCSPC-FLIM investigations in the A431 cell line, complemented by imaging under similar conditions in PC-3. Figure 10 shows the uptake and cellular distribution of compounds *L* **-3**, *L* **-4**, *D* **-3**, *D* **-4**, and *L*-**6**, the intensity images, the fluorescence lifetime micrographs, and lifetime statistical distribution curves of A431 cells incubated overnight at 37 °C with the probes. Analogous behaviors were found for the uptake in the PC-3 cell line (Figures S116–S134) under short-term (20 min to 1 h) as well as overnight (16–18 h) incubation times (Figure 11).

**Figure 10:**
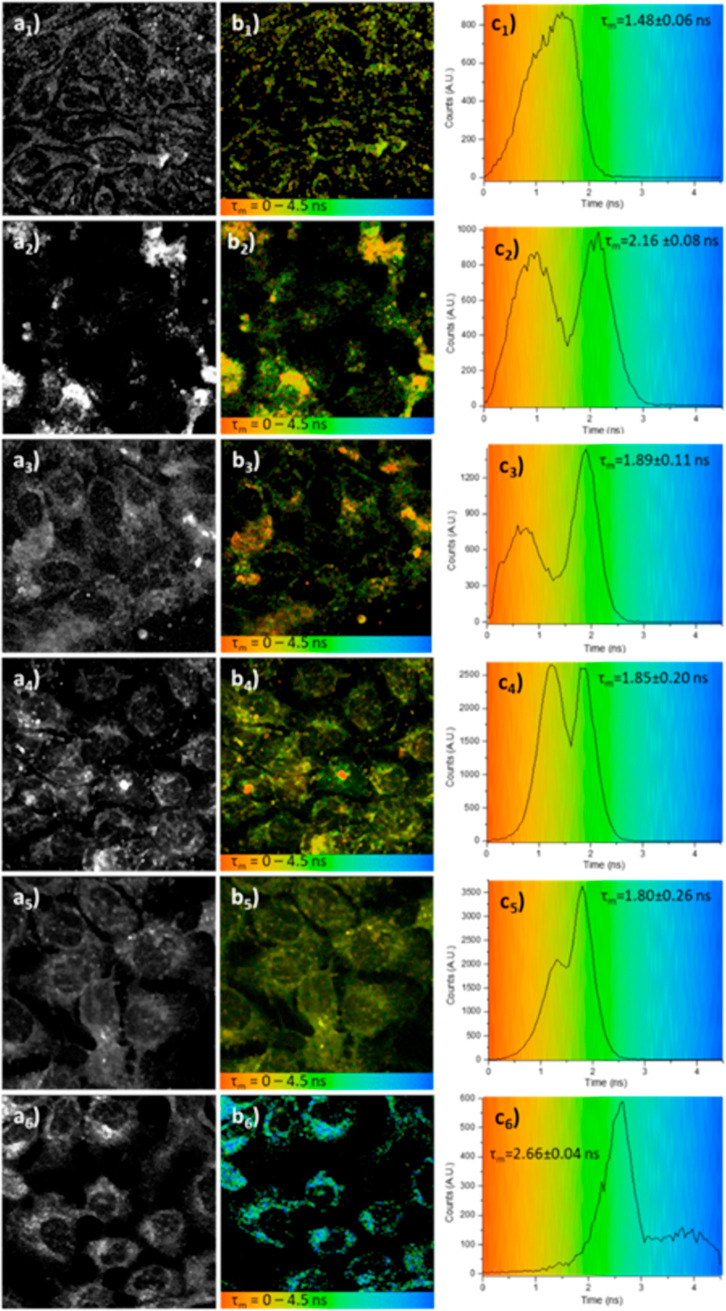
Two-photon fluorescence lifetime imaging of living A431 cells: (a_*x*_) intensity map, (b_*x*_) lifetime maps in a colored code, and (c_*x*_) lifetime distribution (λ_ex_ = 810 nm from a_1_–b_1_ to a_5_–b_5_, probe solutions 100 μM, in 1:99 DMSO/serum-free medium and at λ_ex_ = 910 nm for a_6_–b_6,_ probe solutions 50 μM, in 1:99 DMSO/serum-free medium). (a_1_–c_1_) DMSO/serum-free medium (1:99) control; (a_2_-c_2_) *L* **-3**; (a_3_–c_3_) *L* **-4**; (a_4_–c_4_) *D* **-3**; (a_5_–c_5_) *D* **-4**; (a_6_–d_6_) *L* **-6**. Field of view is 100 μm in each case. Additional micrographs depicting MP FLIM experiments recorded under similar conditions in living cells are given in the Supporting Information.

**Figure 11:**
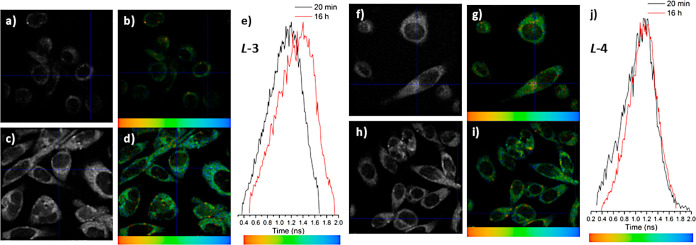
Two-photon fluorescence lifetime imaging of living PC-3 cells: (a_,_c,f,h) intensity maps; (b,d,g), (i) lifetime maps in rainbow-colored coded representations (0–2 ns scale); (e,j) lifetime distributions (λ_ex_ = 810 nm, imaged after 20 min vs. 18 h incubation times at 37 °C. Concentrations: 100 μM, in 1:99 DMSO:serum-free medium: (a–e) *L* **-3**; (a–e) *L* **-4**. Fields of view were 50 μm (c,d,f,g) and 100 μm (a–b and h–j). Additional micrographs depicting MP FLIM experiments recorded under similar conditions in living cells are given in the Supporting Information.

The images a_1_-c_1_ in Figure 10 depict the lifetime mapping of these compounds and the A431 cells incubated with 1:99 DMSO/SFM (serum-free medium) as controls for fluorescence lifetime imaging assays: the lifetime value (general auto fluorescence) of the distribution (τ_m_ = 1.48 ± 0.06 ns) from 2-photon excitation at λ_ex_ = 810 or 910 nm was then used as a reference for the study of the interaction of the probes with the cellular environment (shown in Figure 10 for *L* **-3**, *L* **-4**, *D* **-3**, *D* **-4**, and *L*-**6** and Figure 11, under similar conditions for probes *L* **-3**, *L* **-4**. Additional micrographs are shown in Figures S116–S133.

In the case of compound *L* **-3**, the fluorescence lifetime micrograph (Figure 10(b_2_)) shows that the probe is distributed in the cytosol of the cells, where it accumulates in cellular compartments. Furthermore, micrographs and the corresponding FLIM distribution maps (shown in Figure S10­(a2–c2)) indicate two distinct lifetime distributions (**c**_**2**_). The first is centered around 0.95 ± 0.06 ns, which may be due to the interaction of the probe with the cytoplasmic cellular environment—while the organelle localization (e.g., in lysosomes or mitochondria) is unclear; interestingly, the second component, at 2.16 ± 0.08 ns, may be due to the accumulation of the probe with a minimal interaction with the cellular milieu.^76^

A similar trend was noticed for *L* **-4** (a_3_–c_3_), which also showed two lifetime distributions, centered around 0.72 ± 0.05 ns and 1.89 ± 0.11 ns. The value of τ_m_ (the average of the two weighted lifetimes) suggests that the number of peptide moieties can affect the interactions within the cellular compartments. Compounds *D* **-3** and *D* **-4** presented two groups of lifetime distributions, in particular, *D* **-3** has a τ_1_ of 1.26 ± 0.19 ns and τ_2_ at 1.85 ± 0.20 ns (c_4_). Similarly, the distribution of *D* **-4** (c_5_) shows values of τ_1_ at 1.30 ± 0.15 ns and τ_2_ at 1.80 ± 0.26 ns.

The *D*-enantiomers *D* **-3** and *D* **-4** consistently gave the same values of τ_1_ and τ_2_ in their lifetime distributions (**c**_**4**_ and **c**_**5**_, respectively). *L* **-3**, as well as *L* **-4**, gave lower τ_1_ values than *D* **-3** and *D* **-4**, which suggests that the interaction with cells is affected by the stereochemistry of the molecules. FLIM measurements confirm that in all cases, the compounds with the *L*-enantiomer of the bombesin peptide (compounds **3**, **4**, and **6**) interact differently in live cells compared to their *D*-enantiomers.^62,63^ On the other hand, compounds *L*-**6** showed only one lifetime decay distribution (**c**_**6**_), centered at 2.66 ± 0.04 ns. The species is distributed into the cytosol of the cells, as shown in **b**_**6**_, and it may interact with some of the cellular compartments. Interestingly, the BODIPY-tagged peptide bioconjugate *L*-**6** presented a high fluorescence emission after only 20 min incubation, suggesting a potentially efficient and rapid labeling of cancer cells when compared to the other NDI–peptide probes investigated hereby.

MP FLIM measurements also allowed us to verify the hypothesis that the different number of peptide moieties grafted onto the NDI core (incorporating two vs. one BBN peptide fragments) may affect both the level of uptake into cancer cells and the nature of the fluorescence emission in the cellular environment, likely due to a differing aggregation behavior in an aqueous environment.

From single- and 2-photon fluorescence imaging correlated with lifetime imaging assays, we postulate that the *L*-constructs may be efficiently taken up by PC-3 cancer cells and incorporated within the lysosomes, due to a targeting peptide for these GRPR-expressing prostate cancer cells (the *L*-[7,13] fragment of the bombesin peptide), while the *D*-variants are likely to interact with all cancer cells, albeit less favorably. In addition, bioconjugate constructs incorporating both the blue-to-green emitting NDI core and the orange-to-red and far-red emitting functional BODIPY tag show the expected cellular biolocalization within the endoplasmic reticulum, which was also detectable by means of single- and two-photon fluorescence techniques. Consistently, the results of the blocking imaging experiments performed, where PC-3 were pretreated with a DMSO solution of *L*-[7,13]­BBS then further incubated with *L*-**6** showed, as expected, the significant decrease of the uptake efficiency in pretreated cells (Figures S103 and S104).

Further studies in our laboratories will pursue medicinal chemistry assays to complement MP FLIM microscopy evaluations, aiming to probe whether the inhibition of the GRPR receptors with well-known antagonists^76^ (e.g., JMV594, RC-3095) would elicit different cellular uptake efficiencies for *L*- vs *D*-enantiomers of bombesin-based conjugates. Considering that the chirality of the compounds influenced the uptake efficiency and subcellular distribution, this approach could lead to future new design and delivery avenues toward GRP-targeted peptide conjugates for cancer diagnostic and therapeutic applications.^76–81^

## Conclusions

In summary, we established the unprecedented laboratory-scale microwave-assisted synthesis of a novel series of optical imaging probes based on amino acid and peptide-linked naphthalenediimide (NDI) bioconjugates. We developed a new family of naphthalene diimide probes linked to *L*- vs *D*-bombesin fragments and evaluated the role chirality plays in their chemical and biological properties. These constructs incorporate *D*- and *L*-[7–13] bombesin fragments that selectively target the gastrin-releasing peptide receptor (GRPR) in living cells. Given the significance of optical imaging in biomedical research, particularly for cancer detection, we investigated the role of *D*- and *L*-stereochemistry in fluorescent peptide-based probes. Our design features NDI as a fluorescent linker, leveraging its favorable photophysical properties, including UV–visible absorption/emission, functional tunability, and capacity for supramolecular assembly in organic and aqueous environments.

We synthesized and characterized a new series of NDI–peptide bioconjugates, including monopeptide (*L* **-3**, *D* **-3**) and bis-peptide (*L* **-4**, *D* **-4**) derivatives, incorporating either one or two bombesin [7–13] (BBN[7–13]) units. Additionally, we developed an NDI-based bioconjugate *L*-6 bearing an *L*-BBN­[7–13] moiety and a BODIPY fluorescent tag by using analogous microwave-assisted protocols. These conjugates were extensively analyzed using NMR, fluorescence spectroscopy, and mass spectrometry, confirming their identity.

Aggregation studies on carbon surfaces and in organic media were performed by using scanning electron microscopy (SEM) and multiphoton TCSPC spectroscopy. Circular dichroism (CD) analysis revealed that the self-assembly and aggregation behaviors of these conjugates are dictated by the chirality of the [7–13] bombesin fragment and the presence of 4-iodophenylalanine on the NDI core. The planar aromatic nature of NDI enables facile functionalization, kinetic stability, and aggregation in organic solvents and cellular environments, making it an ideal scaffold for bioimaging applications. Notably, our amino acid-tagged NDI conjugates exhibit efficient cellular uptake and strong fluorescence emission in the cytoplasm of various cancer cell lines.

We also developed a new bioconjugate comprising a BODIPY dye with the *L*-bombesin fragment and discovered that the BODIPY unit influences the localization of the bioconjugate: CFM and MP FLIM results point at a nonspecific behavior where the uptake under the evaluated conditions is governed not only by the targeting peptide, showing a preference for the GRP receptors and a targeted behavior, but also by the BODIPY fluorophore that shows a distribution in the cytoplasm known to be typically in the endoplasmic reticulum.

We also demonstrated that NDI-based constructs form highly emissive supramolecular aggregates detectable via two-photon fluorescence imaging, thus expanding their potential as imaging tools. The depth and quality of analysis provided by MP FLIM, compared to simple emission images, allow for a detailed comparison of the behavior of enantiomeric peptides in cellular environments. This approach reveals that when aggregation dominates uptake, the spectroscopic behaviors of these peptides in living cells may become comparable.

Two-photon FLIM can provide information on the local environment, biolocalization, and the association of stacked versus molecular species in cells and is generally concentration-independent in the absence of aggregation, yet detailed comparisons of *D*- vs -*L* cellular uptake of enantiomeric conjugates were previously understudied. *D*-peptides can be used for targeted delivery and for transporting supramolecularly stacked materials into cells, and we confirmed that these enantiomeric *D*- and *L*-conjugates can exhibit analogous spectroscopic behaviors. Because *D*-peptides are protease-resistant and nonimmunogenic, switching from *L*- to *d* -amino acids makes peptides more stable; however, it is first necessary to confirm that these enantiomeric *D*- and *L*-conjugates exhibit analogous spectroscopic behaviors. Their behavior, along with their self-assembly properties, underscores their potential for imaging and targeting GRPR-overexpressing cancer cells.

To explore living cell interactions, we assessed the uptake and lysosomal localization of bioconjugates using confocal laser scanning microscopy coupled with multiphoton fluorescence lifetime imaging (FLIM). Experiments in live prostate cancer cells (PC-3) and epidermoid carcinoma cells (A431) demonstrated that the *L* vs *D* stereochemistry of the bombesin fragment influences the uptake efficiency and subcellular distribution. These insights are crucial for designing biologically active imaging agents for cancer detection.

Assays centered on MP FLIM further highlighted the differences in uptake and fluorescence behavior based on peptide stereochemistry and conjugation pattern. The presence of two GRPR-targeting peptides per NDI core significantly enhanced uptake and emission in cancer cells, validating our hypothesis that these bioconjugates efficiently interact with their environment, particularly in the *L*-stereoisomeric form.

Although the emission in solution is expected to be the same for *L* vs. *D* conjugates in fluorescently tagged peptides, this detailed spectroscopic characterization of *D* peptides in this context is unprecedented. Having established these behaviors, we can replace *L*-peptides with *D*-inversed peptides, yielding a peptide with a side chain in the correct orientation and greater metabolic stability. These findings contribute to a further understanding of peptide-based molecular probes in cancer research and highlight the role of chirality and self-assembly in cellular interactions. By optimizing NDI-based fluorescent constructs, this work paves the way for next-generation imaging probes in cancer diagnostics and peptide-targeted drug development.

## Experimental Section

### General Experimental Methods

All reagents were purchased from Sigma-Aldrich, Merck Chemicals, FluorousTechnologies, or Alfa-Aesar and were used as supplied without prior purification.

Synthesis of compounds (*L*- and *D*-) **1** and **2** was carried out using a Biotage Initiator Alstra + instrument. Synthesis of peptides **2** (*L*- and *D*-) was carried out on an Activotec Activo-P11 peptide synthesizer. Synthesis of compounds (*L*- and *D*-) **3**, (*L*- and *D*-) **4**, and **6** (*L*-) were performed in a microwave reactor Biotage Initiator 2.5 instrument. Solvents (anhydrous DMF, Et_2_O, DCM MeOH, THF, hexane, and MeCN) were used as purchased from Sigma-Aldrich and Merck.

Automated flash chromatography purification was performed in a Biotage Isolera Four system. Eluents and gradients were chosen as described in the compound procedure, using Biotage KP-Sil cartridges. The internal detector detects the absorbance of the solvents and samples passing through the detector flow cell in the range 200–400 nm. The flow rate (range between 1 and 200 mL/min) was chosen in accordance with the cartridge. Chromatography was also performed using a Biotage Isolera system equipped with a reverse-phase C18-silica cartridge (Sfar Bio C18–Duo 300 Å 20 mm, 30 g). A gradient of acetonitrile and water or methanol and water was used to purify and elute the compounds.

High-performance liquid chromatography (HPLC) was performed using a Dionex UltiMate 3000 preparative system, equipped with a 50 μL loop for analytical work, a 2 mL loop for semipreparative work, and an eight-channel UV/vis detector (Ultimate 3000 Diode Array Detector). For analytical work, a C18-silica column by Hamilton (PRP1, internal diameter 4.1 mm, length 150 mm, particle size 10 μm, pore size 100 Å) was utilized. Integration of the chromatograms was performed using commercial Chromeleon software. Methods used were:

Method A was applied using a Dionex Acclaim 120 C_18_ column (5 μm, 4.6 mm × 150 mm) with a flow rate of 0.5 mL/min. The mobile phase consisted of MeCN and H_2_O, both containing 0.1% TFA. Method: 0 min 5% MeCN; 1 min 5% MeCN; 31 min 95% MeCN; 41 min 95% MeCN; 51 min 5% MeCN; 58 min 5% MeCN.

Semipreparative HPLC (method B) was performed using a Nucleodur C_18_ HTEC (5 μm, 10 × 250 mm) at 1.8 mL/min or a Nucleodur C_18_ HTEC (5 μm, 21 × 250 mm) column at 4.0 mL/min. The same method was used for both columns: 0 min, 5% MeCN; 1 min, 5% MeCN; 21 min, 95% MeCN; 26 min, 95% MeCN; 31 min, 5% MeCN; 36 min, 5% MeCN.

Method C for the analytical HPLC characterization was carried out using a Waters C-18 column (4.6 × 250 mm) with UV/visible detection (254 nm). The gradient elution was 0.8 mL/min with 0.1% TFA milli-Q water as solvent A and 0.1% TFA/acetonitrile as solvent B. A reverse gradient was applied starting with A at 95%, going up to 5% A at 7.5 min, isocratic until 15 min and gradient until 95% A, then holding up to 18 min. All compounds (*D*-/*L*-)**1**–**4**, **5**, and **6** were deemed of analytical purity as indicated by HPLC (>95%). Full spectroscopic characterization and corresponding traces are given in the Supporting Information.

High-resolution mass spectrometry (MS) analyses were performed using a mass spectrometer equipped with an electrospray ion source (Bruker microTOF and MALDI-TOF with Linear and Reflectron analyzers). For peptides, an HPLC/MS method was chosen, eluting the injected sample over a reversed-phase C8-HPLC column, with a water/acetonitrile mobile phase (0.1% formic acid). Analysis of the mass spectrometry data was performed using commercial Bruker and Agilent software. Deviations between measured *m*/*z* values and predicted *m*/*z* values are given as absolute values in ppm.

Solution multinuclear NMR spectra were recorded using a Bruker Avance 500 UltraShield and an Agilent 500 MHz spectrometers. ^1^H and ^13^C chemical shifts are referenced to tetramethylsilane (TMS). ^1^H NMR spectra were recorded on a Varian Mercury VX300­(300 MHz) spectrometer, a Varian Unity (500 MHz) spectrometer, or a Bruker AVC 500 (500 Hz) spectrometer at 298 K and referenced to residual nondeuterated solvent peaks. Chemical shifts are quoted in ppm with resonances reported as either singlet (s), doublet (d), triplet (t), quartet (q), quintet (qt), and multiplet (m) resonances. Coupling constants, J, were measured to the nearest 0.1 Hz. Thirteen C NMR spectra were recorded on a Varian Mercury VX300 (300 MHz) spectrometer or a Varian Unity (500 MHz) spectrometer or on a Bruker AVC 500 (500 Hz) spectrometer at 298 K and were referenced to the residual solvent resonances.

Mass spectrometry was performed using a Bruker Micromass LCT time-of-flight mass spectrometer under the conditions of electrospray ionization (ESI-MS). Accurate masses are reported to four decimal places using tetraoctylammonium bromide (466.5352 Da) as an internal reference. Values were reported as a ratio of mass to charge in Daltons.

Electronic absorption spectroscopy (UV/vis) was performed using a PerkinElmer Lambda 19 spectrometer with UV Winlab software. Spectra were measured using 1.00 cm quartz cuvettes. HPLC characterization (analytical or semiprep HPLC) of compounds was performed by one of three methods A, B, C.

UV–visible experiments were performed on a PerkinElmer Lambda 650 spectrometer operating with UV WinLab 3 software with a scan rate of 500 nm·min^–1^. 2D-fluorescence spectroscopy was carried out using a PerkinElmer LS55 luminescence spectrophotometer with a scan rate of 500 nm·min^–1^, operating with an FL WinLab. All spectra were acquired using quartz cuvettes (path length = 1.00 cm) after recording the spectra for the appropriate reference probe.

Circular dichroism experiments were carried out on a Chirascan instrument with a Xe arc lamp cooled with N_2_. The operating range was from 280 to 700 nm, using a dual polarizing and dual dispersing monochromator, and the temperature range was from 25 to 70 °C, using a water cooler. All spectra were acquired using quartz cuvettes (path length = 1.00 cm) after recording the appropriate reference. Spectra were processed using Spectragraph v1.2.15 software.

Fluorescence spectra were recorded on a PerkinElmer LS55 fluorescence spectrometer using quartz cuvettes with a path of 1 × 1 cm. Fluorescence maps (EEM maps) were recorded with 10 nm excitation increments at a scan speed of 100 nm/min. Relative fluorescence quantum yields were determined with both the PerkinElmer LB650 and PerkinElmer LS55 spectrometers.

SEM images were acquired using a Joel JSM-6480 LV scanning electron microscope with a constant accelerating voltage of 10 kV under high vacuum (∼1.6 · 10^–5^ mbar). All samples were prepared from dilute suspensions of DMSO or MeCN/H_2_O of the compound dropped onto freshly cleaved Ruby Muscovite Mica (Agar Scientific Mica Sheets, AGG250) and dried overnight under a low vacuum. A conducting gold thin film was deposited on top of the samples for standard imaging.

Cell lines used (cancerous, PC-3, LnCap, MCF-7, A431, and healthy control, CHO) were obtained from the American type cell culture (ATCC). Cells culturing experimental details are given in the Supporting Information. Cells were seeded as monolayers in T75tissue culture falcon flasks and cultured in Roswell Park Memorial Institute (RPMI) 1640 media supplemented with 10% fetal bovine serum (FBS), *L*-glutamine, penicillin, and streptomycin. Cells were incubated at 37 °C, under an atmosphere of 5% CO_2_, and passaged with trypsin when 70–80% confluent. For fluorescence microscopy, cell monolayers were plated in glass-bottomed Petri dishes 3 days in advance to ensure adhesion to the surface, with an estimated 7.5 × 10 4 cells per dish.

Prior to microscopy experiments, cells were seeded onto sterile glass dishes and incubated for 48 h prior to the addition of fluorescent compounds to allow them to adhere to the surface. Compound stock solutions (1 mM) were prepared in DMSO. After aspiration, cells were washed 5 times with Hank’s Balanced Salt Solution (HBSS) before adding RPMI (990 μL) and the compound solution (10 mM solution, 10 μL giving a final concentration of 10 μM). Cells were then incubated for 20 h at 37 °C, washed three times with HBSS to remove traces of noninternalized compounds, and recovered with1 mL of serum-free RPMI.

Confocal microscopy was performed using a Nikon Eclipse Ti2-E inverted confocal microscope with an LU-N3 laser unit (405, 488, and 561 nm) or using a Nikon A1Rsi Laser Scanning Confocal Microscope system fitted with 60× oil objective lens. The microscope was also fitted with a motorized piezo z-stage, halogen lamp, and mercury lamp for visual fluorescence microscope. All images were processed by using functions within the NIS Elements software package.

Cell uptake studies using correlated confocal fluorescence microscopy and two-photon FLIM were performed on living PC-3 cells, adhering to glass bottom Petri dishes and incubated with the compound over 15, 20, and 30 min at 37 °C. Images of cells without probe uptake but in the presence of the DMEM and 1% DMSO alone were recorded as background lifetimes to account for cellular autofluorescence. The compounds were dissolved in a small amount of DMSO (1%) and added to the cell culture medium to give a concentration ranging between 10 and 100 μM. Fluorescence lifetime decays were measured for the complete field of view over 5 min intervals. Uptake reached a maximum within 30 min, and the images and data presented below were obtained after 20 min. FLIM images were recorded by raster scanning the focused NIR (910 nm) 200 fs pulsed laser light at 76 MHz (Coherent Mira F900, pumped by a Verdi V15 lasers), through a 60× water immersion objective with an NA or 1.2. A BG39 filter was used to filter the fluorescence following the multiphoton excitation and recorded (using a Hamamatsu R3809U) the point decays at every location within the cell (generating a minimum of 128 × 128) pixel image and lifetimes calculated at each pixel using the standard software Becker and Hickl SPCM Image^82^ package (ver. 4), using our previously described correlated imaging experimental setup.^83–86^ Airyscan super resolution images were acquired using a Zeiss LSM 880 inverted confocal microscope equipped with excitation laser lines at 405, 488, and 561 nm and fitted with a Plan-Apochromat 63x/1.4 Oil DIC M27 objective. All images were processed by using the Zen black software package.

### Cytotoxicity Evaluations: MTT Assays

After splitting the cells, cells (3 × 103 cells per well) were seeded on a sterile 96-well plate and incubated for 48 h in order to let the cells adhere to the plates. The compound of interest was then subsequently loaded at different concentrations into the wells and incubated for 48 h more. The concentrations used ranged between 250 μM or 0.01 mg/mL and 1 nM or 1 ng/mL (1% DMSO with compound, 99% RPMI and 10% FCS for PC-3). Subsequently, cells were washed three times with PBS, and 3-(4,5-dimethylthiazol-2-yl)-2,5- diphenyltetrazolium bromide (MTT) was added (0.5 mg/mL in serum-free medium (SFM)), followed by a 2 h incubation. After aspiration to remove the serum-free medium, 100 μL of DMSO was added to solubilize the precipitated formazan species, and 96-well plates were read by an ELISA plate reader, Molecular Devices Versa Max (BN02877). The absorption wavelength was 570 nm, and a 690 nm wavelength was used as a reference.

### Cytotoxicity Evaluations: Crystal Violet Assays

After splitting the cells, cells (3 × 103 cells per well) were seeded on a sterile 96-well plate and incubated for 48 h to let the cells adhere to the plates. The compound of interest was subsequently loaded at different concentrations into the wells and incubated for a further 48 h. The concentrations used ranged between 250 μM or 0.01 mg/mL and 1 nM or 1 ng/mL (1% DMSO with compound, 99% RPMI (10% FCS) for PC-3. Subsequently, the medium from the plates was carefully aspirated so as not to disturb the colonies, and the plates were gently washed with PBS. Next, a mixture of methanol/PBS (1:1) was added in a sufficient volume to cover the colonies, and it was left for 15 min. After this time, it was removed, and 100% methanol was added for a further 15 min. The methanol was then removed, and 0.5% crystal violet solution (in 20% methanol and 80% water) was added and left to allow sufficient staining. The crystal violet solution was removed by carefully rinsing the cell plates with an indirect flow of tap water. The plates were inverted and left on the bench at room temperature to dry the water. Methanol (200 μL) was added to each well, and the plate was incubated with its lid on for 20 min at room temperature on a bench rocker with a frequency of 20 oscillations per minute. The 96-well plates were then read with an ELISA plate reader, Molecular Devices Versa Max (BN02877). The absorption wavelength was 570 nm, and 690 nm wavelength was used as a reference.

### General Synthesis of the Mono- and Bis-Peptide and Amino Acid-Tagged NDI-Conjugates, *L*-**3** or *D*-**3**, *L*-**4** or *D*-**4**, Respectively

The starting peptides *L*-**2** or *D*-**2** were synthesized according to standard protocols, outlined in the Supporting Information. The corresponding Osu-activated NDI precursors *L*-**1 or** *D*-**1** were synthesized following adapted procedures, based on previously described protocols.^10,11,85–88^ In a pressure-tight microwave vessel, the peptides *L*-**2** or *D*-**2** (6 mg, 0.07 mmol) and the corresponding OSu-activated NDI precursors *L*-**1** or *D*-**1** (110 mg, 0.14 mmol) were dissolved in anhydrous DMF (5 mL). To the resulting mixture was added Et_3_N (0.20 mL, 14.33 mmol), and the reaction mixture was heated at 70 °C for 45 min in the microwave system. The solvent was partially removed under reduced pressure, and a light brown precipitate was obtained after the addition of Et_2_O. The bright brown solid was washed with Et_2_O and centrifuged 3 times at 10,000 rpm for 3 min. The bright brown solid was freeze-dried overnight and purified by semiprep HPLC (method B). The analogous procedure was applied to the coupling between *D*-**2** and *D*-**1**. Yields: 1.4 mg, 1.2% for *L*-**3**; 6.88 mg, 4.1% for *L*-**4**; 1.5 mg, 1.3% for *D*-**3**; 1.8 mg, 1.1% for *D*-**4**.

HPLC (method A): Rt = 37.1 min (*L*-**3** and *D*-**3**), 36.2 min (*L*-**4** and *D*-**4**). *L*-**3**: ESI^+^-TOF (CH_3_OH): *m*/*z* found, 1702.3797 [M + H]^+^, 1724.3672 [M + Na]^+^; calculated for C_74_H_77_I_2_N_15_O_17_: 1701.3711 Da. *D*-**3**: ESI^+^-TOF (CH_3_OH): *m*/*z* found, 1702.3811 [M + H]^+^, 1724.3710 [M + Na]^+^; calculated for C_74_H_77_I_2_N_15_O_17_: 1701.3711 Da. *L*-**4**: ESI^+^-TOF (CH_3_OH): *m*/*z* found, 1198.4002 [M + 2H]^2+^, 2395.7806 [M]^+^; calculated for C_108_H_128_I_2_N_26_O_22_: 2394.7786 Da. *D*-**4**: ESI^+^-TOF (CH_3_OH): *m*/*z* found, 1198.4050 [M + 2H]^2+^, 2395.8557 [M]^+^; calculated for C_108_H_128_I_2_N_26_O_22_: 2394.7786 Da.

### Stepwise Synthesis of Compound *L* **-6**, Incorporating *L*-[7,3]­BBN

#### Step 1: Synthesis of 8-Carboxyphenyl BODIPY

The known compound 8-carboxyphenyl BODIPY (4,4-difluoro-8-(4′-carboxyphenyl)-1,3,5,7-tetramethyl-4-bora-3a,4a-diaza-*s*-indacene) was synthesized as described in the Supporting Information.

#### Step 2: Synthesis of 2,5-Dioxopyrrolidin-1-yl 4-(5,5-Difluoro-1,3,7,9-tetramethyl-5*H*-4L4,5L4-dipyrrolo­[1,2-*c*:2′,1′-*f*]­[1,3,2]­diazaborinin-10-yl)­benzoate

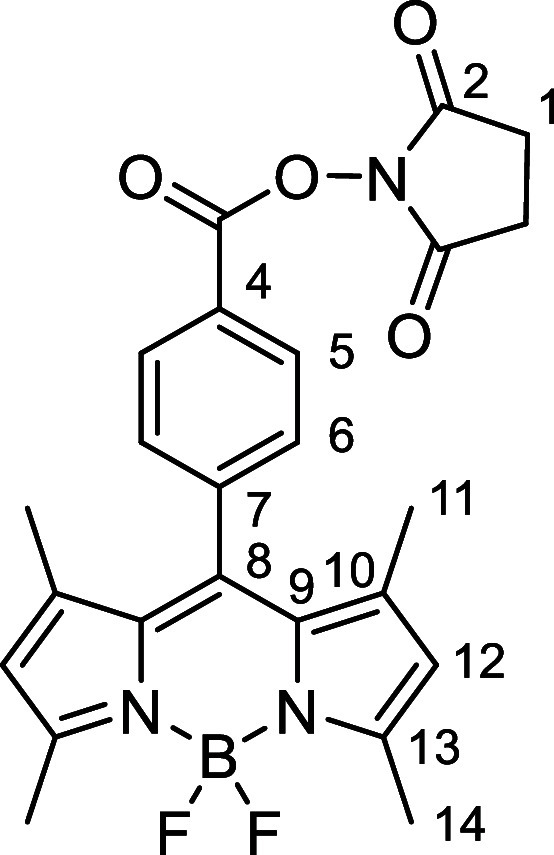



8-Carboxyphenyl BODIPY (0.100 g, 0.27 mmol), *N*-hydroxysuccinimide (0.065 g, 0.27 mmol), and EDC·HCl (0.107 g, 0.56 mmol) were dissolved in CH_2_Cl_2_ (60 mL). The reaction mixture was stirred at room temperature for 3 h. The solvent was removed under vacuum and the residue purified by flash column chromatography using hexane/ethyl acetate (1:1) as eluent. The product was obtained as a crystalline orange solid (0.135 g, 99%). ^1^H NMR (500 MHz, CDCl_3_, 25 °C): δ 8.27 (d, ^3^ *J* = 8.4 Hz, 2H, H-4), 7.50 (d, ^3^ *J* = 8.4 Hz, 2H, H-6), 6.01 (s, 2H, H-12), 2.94 (s, 4H, H-1), 2.56 (s, 6H, H-14), 1.38 (s, 6H, H-11). ^13^C NMR (125 MHz, CDCl_3_, 25 °C): δ 169.3 (C-2), 161.4 (C-3), 156.5 (C-13), 143.0 (C-10), 142.2 (C-8), 139.4 (C-7), 131.4 (C-5), 130.8 (C-4), 129.2 (C-6), 125.9 (C-9), 121.8 (C-12), 25.8 (C-1), 14.9 (C-14), 14.8 (C-11). Mass spectrum: ESI-MS calc. for C_25_H_25_BF_2_N_3_O_5_ [M – H]^−^: 496.1855; found, 496.1867. Elem. Anal. (%) found (calcd) for C_24_H_22_BF_2_N_3_O_4_: C, 61.78 (61.96); H, 4.88 (4.77); N, 8.89 (9.03). IR (solid): ν (cm^–1^) 2921, 2852, 1802, 1778, 1737,1542, 1065, 721. HPLC (method B): Rt (min) 10.64.

#### Step 3: Synthesis of Compound **5**

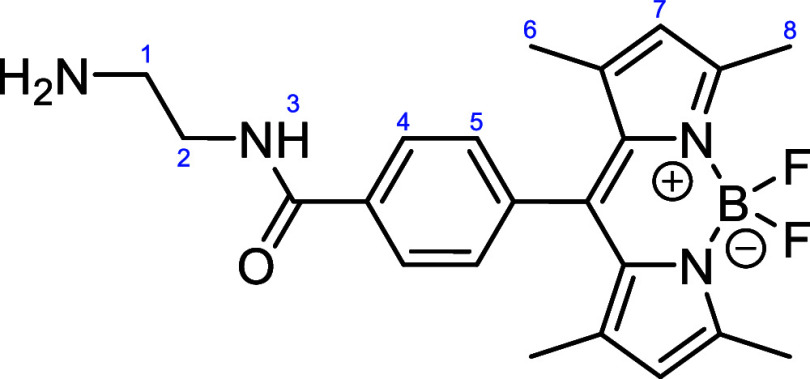



To a solution of BODIPY OSu ester (0.600 g, 1.29 mmol) and *N*, *N*-DIPEA (2.5 mL, 12.9 mmol) in DMF (50 mL) at 60 °C, *N*-6-(*tert*-butoxycarbonyl)­diamine (0.950 g, 3.86 mmol) was added portionwise, and the reaction mixture was stirred at 60 °C for 7 h. The solvent was removed under vacuum and the residue dissolved in CH_2_Cl_2_ and washed with water (3 × 50 mL). The organic layer was dried over MgSO_4_ and the solvent removed under a vacuum. The purification was performed by automated flash column chromatography using CH_2_Cl_2_/MeOH (0–10%) as eluent. The desired product, compound **5**, was obtained as a red solid (0.699 g, 91%). ^1^H NMR (500 MHz, DMSO-*d*_6_): δ 8.67 (*t*, 1H, H-3, *J* = 5.6 Hz), 8.06 (*d*, 2H, H-4, *J* = 7.8 Hz), 7.50 (*d*, 2H, H-5, *J* = 7.7 Hz), 6.19 (s, 2H, H-7), 3.35 (*q*, 2H, H-2, *J* = 6.1 Hz), 2.79 (*t*, 2H, H-1, *J* = 6.3 Hz, 1H), 2.46 (s, 6H, H-6), 1.34 (s, 6H, H-8).

#### Step 4: Synthesis of *L*-**6**

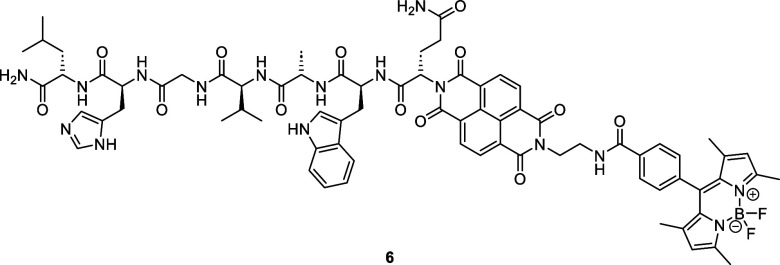



In a pressure-tight microwave vessel, NDA (42 mg, 0.16 mmol) and **5** (50 mg, 0.12 mmol) were dissolved in anhydrous DMF (1 mL) and Et_3_N (0.02 mL). The resulting mixture was sonicated for 5 min. When it was homogenized, the brown solution was heated in the microwave system at 40 °C for 5 min and then at 140 °C for a further 5 min. The solvent was removed by rotary evaporation, and the resulting light brown solid was obtained by automated flash chromatography (silica gel, gradient from 20% MeOH in DCM to 100% MeOH). This solid (44 mg, 0.06 mmol) was solubilized in anhydrous DMF (2 mL) with *L* **-2** (55 mg, 0.07 mmol) and Et_3_N (0.01 mL, 70 μmol). The mixture was heated at 70 °C for 1 h in a microwave system. The obtained dark red solution was washed with Et_2_O and centrifuged 3 times at 10,000 rpm for 3 min. The dark solid was freeze-dried overnight and purified by semipreparative HPLC to give the desired compounds *L*-**6** (69.2 mg, 30% yield). ESI^+^-TOF (CH_3_OH): *m*/*z* found, 1451.6278 [M + H]^+^; calculated for C_74_H_81_BF_2_N_16_O_13_: 1450.6230 Da. HPLC (method A): Rt = 40.2 min.

## Supplementary Material















## Data Availability

Data for this research are given in the Supporting Information and are available from the authors upon request.

## Notes

List of compounds, materials and methods, synthetic procedures of HPLC separations and mass spectrometry analysis, NMR spectroscopy, circular dichroism, HPLC traces of purified compounds used for cellular imaging, UV–visible and fluorescence spectroscopies, FRET Analysis, SEM experiments, in vitro assays, and 2-photon TCSPC and FLIM investigations (PDF)
xyz coordinates of the optimized geometries for the *L*-**3** molecule alone MM+ Model (XYZ)
xyz coordinates of the optimized geometries for *L*-**3** as a stack of molecules MM+ Modeled stacks (XYZ)
xyz coordinates of the optimized geometries for the *L*-**4** molecule alone MM+ Model (XYZ)
xyz coordinates of the optimized geometries for *L*-**4** as a stack of molecules MM+ Model stacks (XYZ)
xyz coordinates of the optimized geometries for the *L*-**6** molecule alone MM+ Model (XYZ)
xyz coordinates of the optimized geometries for the *L*-**6** as a stack of molecules, MM+ Model (XYZ)
