# Supplementary material for: Functional D- and L‑Naphthalenediimide-Peptides: Microwave-Driven Synthesis, Supramolecular Aggregation, and Multiphoton Fluorescence Lifetime Imaging Microscopy in Living Cells
Source: ACS Bio Med Chem Au. 2025 Jul 18;5(6):947–65. doi: 10.1021/acsbiomedchemau.5c00064 (PMC12715529; doi:10.1021/acsbiomedchemau.5c00064)
Supplement: Supplementary file 1 [file bg5c00064_si_001.pdf]

Supporting information

Functional D- and L-Naphthalenediimide-peptides: Microwave-Driven Synthesis, Supramolecular Aggregation and Multiphoton Fluorescence Lifetime Imaging Microscopy in Living Cells

Simone G. Giuffrida, [1] *David G. Calatayud,* [2]* *Fernando Cortezon-Tamarit,* [1] *Haobo Ge,* [1] *Vincenzo Mirabello,* [1] *Dora-Maria Răsădean,* [1] *Charareh Pourzand,* [3,4] *Stanley W. Botchway,* [5] *Pedro Estrela* [6,7] *Gheorge Dan Pantoş,* [1]* *Ian M. Eggleston,*[3,4]* *and Sofia I. Pascu*[1,4]*

[1] Department of Chemistry, University of Bath, Claverton Down, Bath, BA2 7AY, UK
E-mail: s.pascu@bath.ac.uk, g.d.pantos@bath.ac.uk
[2] Department of Inorganic Chemistry, Facultad de Ciencias, Universidad Autónoma de Madrid, Francisco Tomás y Valiente 7, 28049, Madrid, Spain
Email: david.gcalatayud@uam.es
[3] Department of Life Sciences, University of Bath, Claverton Down, Bath, BA2 7AY, UK
Email: ime203@bath.ac.uk
[4] Centre for Therapeutic Innovation, University of Bath, BA2 7AY, Bath, UK
[5] Central Laser Facility, Rutherford Appleton Laboratory, Research Complex at Harwell, STFC Didcot, OX11 0QX, UK
[6] Department of Electronic and Electrical Engineering, University of Bath, Claverton Down, Bath, BA2 7AY, UK
[7] Centre for Bioengineering & Biomedical Technologies (CBio), University of Bath, Claverton Down, Bath, BA2 7AY, UK

Table of Content

1 List of compounds S3

2 Materials and methods S4

3 Synthetic procedures S6

4 HPLC separations and mass spectrometry analysis S15

5 NMR spectroscopy S31

6 Circular dichroism S39

7 HPLC traces of purified compounds used for cellular imaging S41

8 UV-visible and fluorescence spectroscopies S45

9 FRET Analysis S49

10 SEM experiments S51

11 *In vitro* assays.. .... S54

12 2-Photon TCSPC and FLIM investigations……………………………………S85

1 List of compounds

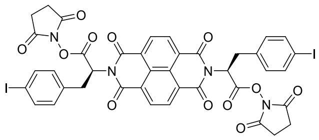

L-1

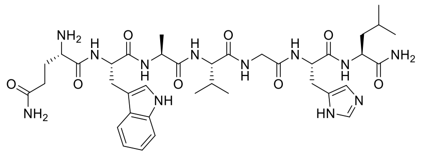

L-2

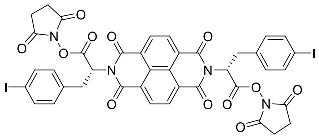

D-1

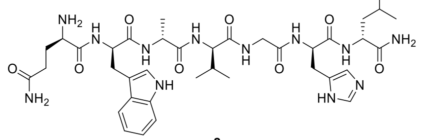

D-2

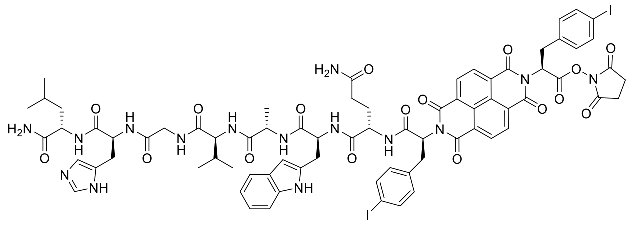

L-3

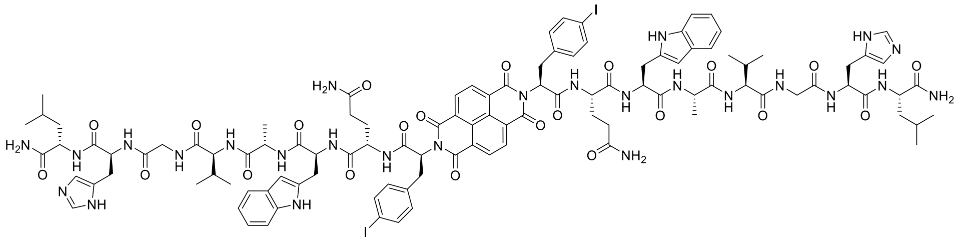

L-4

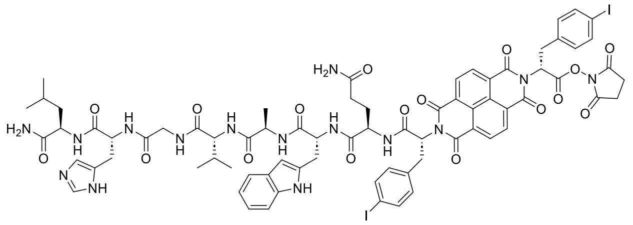

D-3

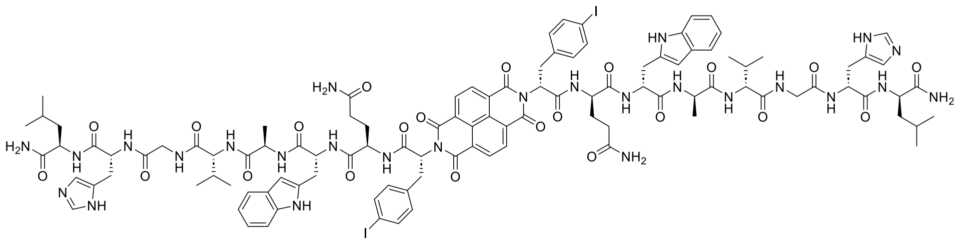

D-4

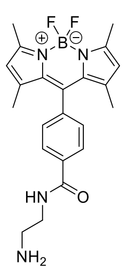

5

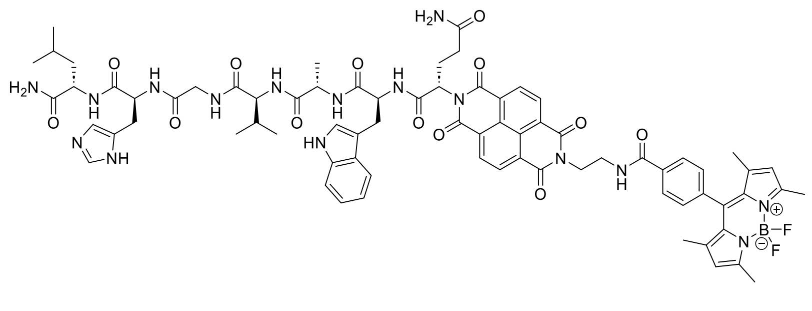

L-6

Figure S1. Compounds synthesised and fully characterised spectroscopically herein

2 Materials and methods

The syntheses of compounds (L- and D-) **3**, (L- and D-) **4** and **6** (L enantiomer) were performed in a microwave reactor *Biotage Initiator Alstra+* instrument. The solvents were anhydrous DMF, Et_2O, DCM, MeOH, THF, hexane and MeCN, and used as supplied from Sigma-Aldrich and Merck.

General preparation of L- and D-[7,13]BBN peptides involved automated Fmoc solid-phase peptide synthesis on Rink amide resin using either a *Biotage Initiator Alstra+* instrument or an *Activotec ACTIVO*-P11 peptide synthesizer fitted with a reactor heating jacket.

Solution multinuclear NMR spectra were recorded using a *Bruker Avance 500 UltraShield* and an *Agilent 500 MHz* spectrometers. ^1H and ^{13}C chemical shifts are referenced to tetramethylsilane (TMS).

Automated flash chromatography purification was performed using a *Biotage Isolera Four* system using *Biotage KP-Sil* cartridges. Eluents and gradients are described in the corresponding compound synthesis procedures. The flow rate (range between 1 and 200 mL/min) was chosen in accordance to the nature of each cartridge. The internal detector detected the absorbance of the solvents and samples passing through the detector flow cell in a range of 200-400 nm.

Analytical HPLC chromatograms were acquired in reverse phase (RP) mode under a range of conditions, as follows.

Method A was applied using a *Dionex Acclaim 120 C_{18}* column (5 µm, 4.6 x150 mm) with a flow rate of 0.5 mL/min. The mobile phase consisted of MeCN and H_2O, both containing 0.1% TFA. Method : 0 min 5% MeCN; 1 min 5% MeCN; 31 min 95% MeCN; 41 min 95% MeCN; 51 min 5% MeCN; 58 min 5% MeCN.

Semi-preparative HPLC (*Method B*) was performed using a *Nucleodur C_{18} HTEC* (5 µm, 10 x 250 mm) at 1.8 mL/min or a *Nucleodur C_{18} HTEC* (5 µm, 21 x 250 mm) column at 4.0 mL/min. The same method was used for both columns: 0 min 5% MeCN; 1 min 5% MeCN; 21 min 95% MeCN; 26 min 95% MeCN; 31 min 5% MeCN; 36 min 5% MeCN.

Method C for the analytical HPLC characterisation was carried out using a Waters C-18 column (4.6 x 250 mm) with UV/visible detection (254 nm). The gradient elution was 0.8 mL/min with 0.1% TFA milli-Q water as solvent A and 0.1% TFA/acetonitrile as solvent B. A reverse gradient was applied starting with A at 95%, going up to 5% A at 7.5 minutes, isocratic until 15 minutes and gradient until 95% A, then hold to 18 min.

High resolution mass spectrometry (MS) analyses were performed using a mass spectrometer equipped with an electro-spray ion source (*Bruker microTOF* and MALDI-TOF with Linear and Reflectron analysers).

Circular dichroism experiments were carried out on a *Chirascan* instrument with a Xe arc lamp, cooled with N_2. The operating range was from 280 to 700 nm, using a dual polarising and dual dispersing monochromator, and the temperature range was from 25 to 70 ^oC, using a water cooler. All spectra were acquired using quartz cuvettes (path length = 1.00 cm) after recording the appropriate reference. Spectra were processed using *Spectragryph v1.2.15* software.

UV-visible experiments were performed on a *Perkin-Elmer Lambda 650* spectrometer, operating with *UV Winlab 3* software with a scan rate of 500 nm min^{-1}. Excitation-eission maping (2D-fluorescence spectroscopy) was carried out in a *Perkin-Elmer LS55* luminescence spectrophotometer operating

with *FL WinLab*. All spectra were acquired using quartz cuvettes (path length = 1.00 cm) after recording the spectra for the appropriate reference probe.

SEM images were acquired using a *Jeol JSM-6480 LV* Scanning Electron Microscope with a constant accelerating voltage of 10 kV under high vacuum (~$1.6 \cdot 10^{-5}$ mbar). All samples were prepared from dilute suspensions of DMSO or ACN:H_2O of the compound dropped onto freshly cleaved Ruby Muscovite Mica (*Agar Scientific* Mica Sheets, AGG250) and dried overnight under low vacuum. A conducting gold thin film was deposited on top of the samples for standard imaging.

General cell culturing methods.

Cells were cultured at 37 °C in a humidified atmosphere in air and diluted once confluence had been reached. Cells were cultured in DMEM medium with 10% fetal calf serum (FCS) and 100 U/mL penicillin. Samples for fluorescence were prepared in the following way: surplus supernatant containing dead cell matter and excess protein was discarded; the live adherent cells were then washed with two 7 mL aliquots of Phosphate Buffer Saline solution to remove any remaining medium containing FCS. To resuspend the cells in solution, they were incubated in 2 mL of trypsin/EDTA (500 mg/L trypsin, 200 mg/L EDTA) solution for 3 min at 37 °C. After trypsinizing, 5 mL of DMEM was added to inactivate the trypsin and the solutionwas centrifuged for 5 min to remove any remaining dead cell matter. The supernatant liquid was poured off and DMEM was added to the cell matter left behind to give a sufficient concentration of cells. The cells were plated in a Petri dish containing a glass coverslip and left for 24 h to adhere. One hour before fluorescence imaging measurements were made, the DMEM was replaced with phenol red-free DMEM (e.g. that does not contain any fluorescent indicator dyes such as phenol red, therefore making it suitable for fluorescence imaging studies).

Microscopy Investigations in Living Cells.

Confocal fluorescence microscopy was performed using a *Nikon A1Rsi Laser Scanning Confocal Microscope* system fitted with 60X oil objective lens. The microscope was also fitted with a motorised piezo z-stage, halogen lamp and mercury lamp for visual fluorescence microscope. All images were processed using functions within the *NIS Elements* software package.

Fluorescence lifetimes and FLIM measurements were conducted at the Rutherford Appleton Laboratory (Central Lasers Facility, OCTOPUS Cluster, Research Complex at Harwell). Lifetime calculations were processed using *SPCImage 7.4* software.

Cell uptake studies using correlated confcal fluorescence microcopy and two-photon FLIM were performed on living PC-3, LNCap and A431 cells, adhering to glass bottom Petri dishes and incubated with the compound over 15, 20, and 30 min at 37°C. Images of cells without probe uptake but in the presence of DMEM and 1% DMSO alone were recorded as background lifetimes. The compounds were dissolved in a small amount of DMSO no larger than 2% and added to the cell culture medium to give a final concentration of 10 μM to 100 μM in cells plates. Decay lifetimes were measured for the complete field of view over 5 min intervals. Uptake reached a maximum within 30 min and the images and data presented below were obtained after 20 min. FLIM images were recorded by raster scanning the focused NIR (810 or 910 nm) 200 fs pulsed laser light at 76 MHz, through a 60× water immersion objective. A BG39 filter was used to filter the fluorescence following the multiphoton excitation and recorded (using a Hamamatsu R3809U) the point decays at every location within the cell (generating a minimum of 128 × 128) pixel image and lifetimes calculated at each pixel using the standard software Becker and Hickl SPCMImage package (ver. 4).

3 Synthetic procedures

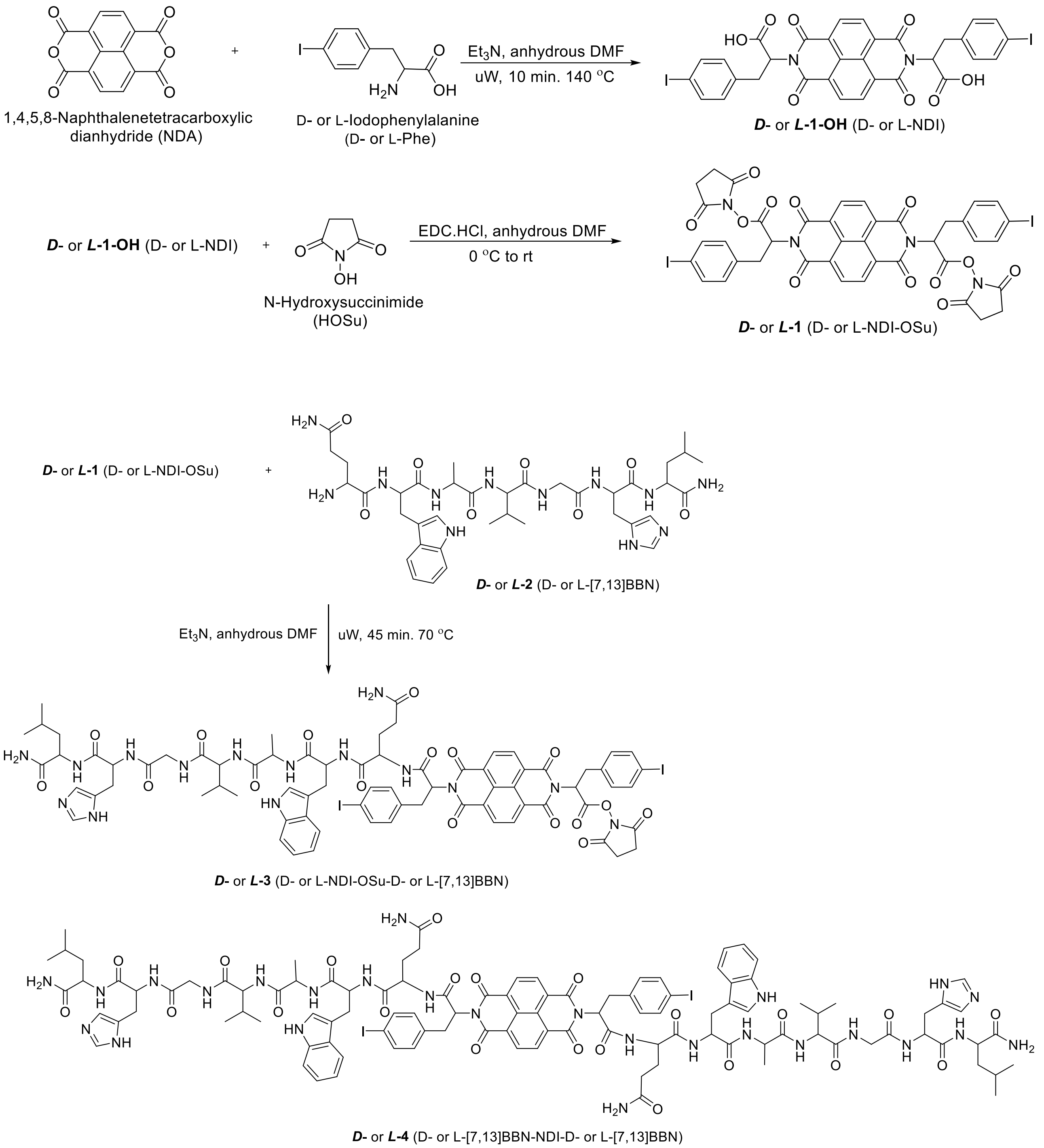

Scheme S1. Overview of microwave-driven routes as the main synthetic protocols for the mono- and bis-peptide and amino acid-tagged NDI-conjugates

Synthesis of bis(2,5-dioxopyrrolidin-1-yl) 2,2'-(1,3,6,8-tetraoxo-1,3,6,8-tetrahydrobenzo[lmn][3,8]phenanthroline-2,7-diyl)bis(3-(4-iodophenyl)propanoate) (L-1 or D-1)

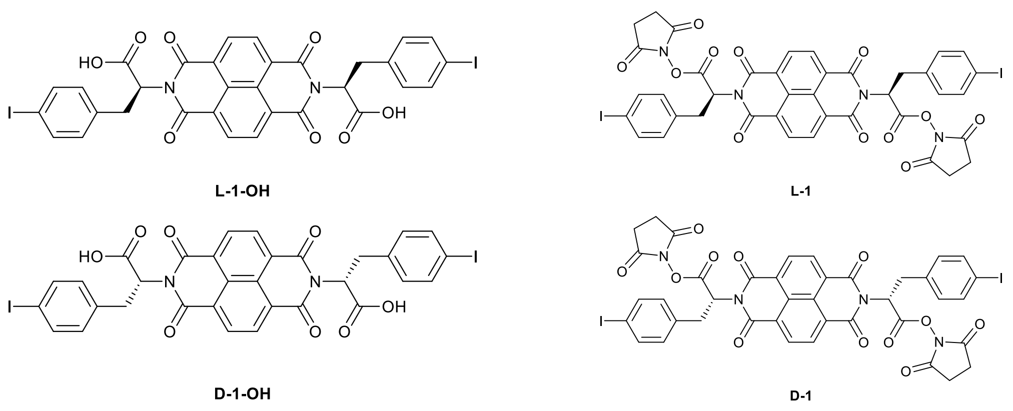

Figure S2. *L*- and *D*-stereoisomers of the NDI intermediates of type **1-OH** and compound **1**.

In a pressure-tight microwave vial, 1,4,5,8-naphthalenetetracarboxylic anhydride (NDA) (200 mg, 0.75 mmol) and the respective iodo-phenylalanine (438 mg, 1.50 mmol) were dissolved in anhydrous DMF (5 mL) and afterwards Et_3N (0.20 mL, 14.3 mmol) was added. The resulting suspension was sonicated until complete homogenisation. The reaction mixture was reacted in a Biotage Initiator Alstra+ microwave for 10 min at 140 °C. Subsequently, the solvent was partially removed under reduced pressure and a yellow precipitate was obtained after the addition of acetone and 1 M HCl. The solid was filtered and washed with acetonitrile, 1 M HCl, deionised water and n-pentane. Yields: 360.1 mg, 59% for *L*-1-OH; 366.3 mg, 60% for *D*-1-OH. Compound **1-OH** was obtained and used in subsequent reactions steps without any further purification.

^{1}H NMR (500 MHz, 298 K, DMSO-*d*$_6$): δ = 13.11 (*bs*, 2H, H-7), 8.67 (*s*, 4H, H-1), 7.51-7.49 (*m*, 4H, H-11), 7.03-7.01 (*m*, 4H, H-10), 5.84 (*dd*, 2H, H-5, *J* = 5.5 Hz, J = 9.3 Hz), 3.56 (*dd*, 2H, H-8', *J* = 5.6 Hz, *J* =14.22 Hz), 3.27 (*dd*, 2H, H-8'', *J* = 9.2 Hz, *J* = 14.3 Hz).

^{13}C NMR (125 MHz, 298 K, DMSO-d6): δ = 170.51 (C-4), 162.52 (C-6), 137.37 (C-11), 132.99 (C-10), 131.92 (C-1), 131.78 (C-3), 126.59 (C-9), 126.14 (C-2), 92.68 (C-12), 54.83 (C-5), 34.95 (C-8).

The respective enantiomers of 1-OH (124 mg, 0.15 mmol) and *N*-hydroxysuccinimide (94 mg, 0.82 mmol) were dissolved in anhydrous DMF (5 mL). The solution was stirred for 15 min in an ice bath and, in the meantime, a solution of EDC·HCl (232 mg, 1.20 mmol) in anhydrous DMF (5 mL) was prepared. The EDC·HCl solution was added to the imide solution and the resulting reaction mixture was stirred for 15 min. After that time, the ice bath was removed and the mixture was left for 24 h, under stirring, at room temperature. The solvent was partially removed, and the brown syrup that was obtained was suspended in acetonitrile (2 mL) and afterwards, deionised water was added. The dark yellow precipitate was filtered and washed with *n*-pentane. The yellow fine powder was dried under vacuum. No further purification was required. The same procedure was applied for the *D*-enantiomer. Yields: 125.5 mg, 83% for ***L*-1**; 126.3 mg, 84% for ***D*-1**.

^{1}H NMR (500 MHz, 298 K, DMSO-d6): δ = 8.72 (*s*, 4H, H-1), 7.53-7.50 (*m*, 4H, H-12), 7.09-7.07 (*m*, 4H, H-11), 6.32 (*ddd*, 2H, H-5, *J* = 2.7 Hz, *J* = 6.1 Hz, *J* = 8.7 Hz), 3.67-3.63 (*m*, 2H, H-9'), 3.43-3.38 (*m*, 2H, H-9''), 2.73 (*s*, 8H, H-8).

^{13}C NMR (125 MHz, 298 K, DMSO-d6) δ = 169.66 (C-6), 164.92 (C-7), 161.73 (C-4), 137.07 (C-12), 136.05 (C-10), 131.67 (C-1), 131.59 (C-11), 126.12 (C-3), 125.56 (C-2), 92.86 (C-13), 52. 28 (C-5), 33.36 (C-9), 25.41 (C-8).

Synthesis of the fragment [7,13] of bombesin peptide (L-2 or D-2)

Bombesin is an agonist of the Gastrin Releasing Peptide Receptor (GRPR), overexpressed in PCa cancer cells The full sequence contains 14 amino acids although shorter sequences show biological activity. For these studies, the C-terminal Met residue was omitted from the truncated bombesin fragments to enhance stability with respect to redox chemistry.

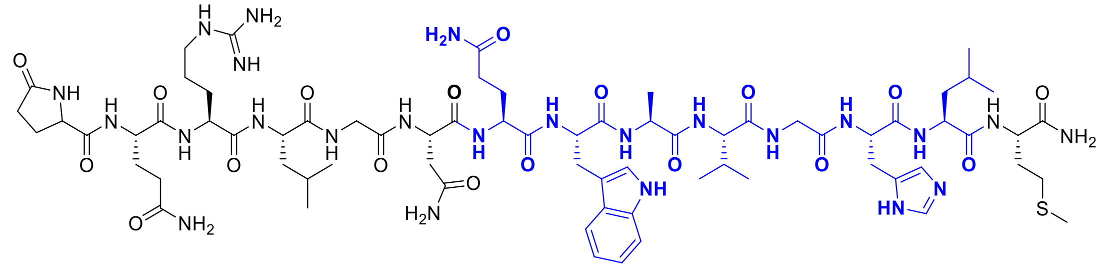

(a)

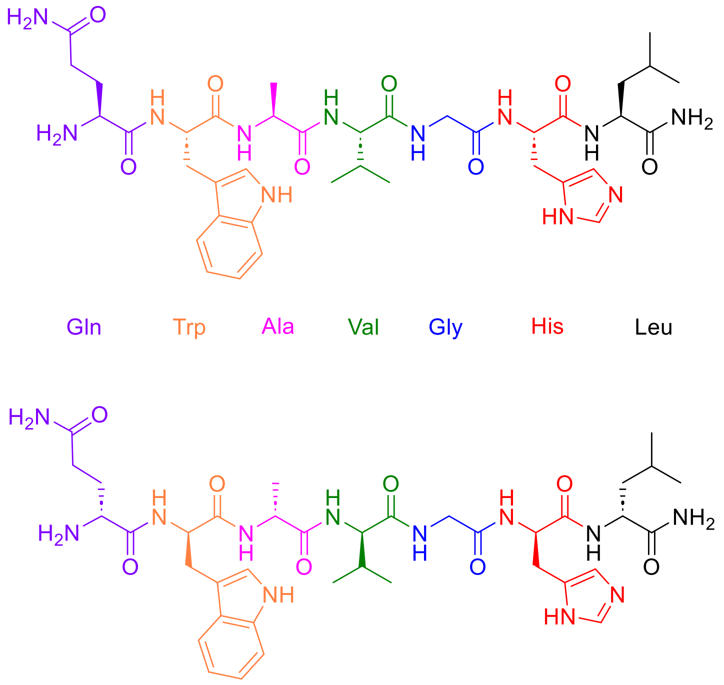

L-[7,13]BBN
(*L*-2)

D-[7,13]BBN
(*D*-2)

(b)

Figure S3. (a) Structural representation of bombesin with the L-[7, 13] fragment highlighted in blue. (b) Structural representations of the two synthesised enantiomers of the [7-13]BBN peptide derivatives.

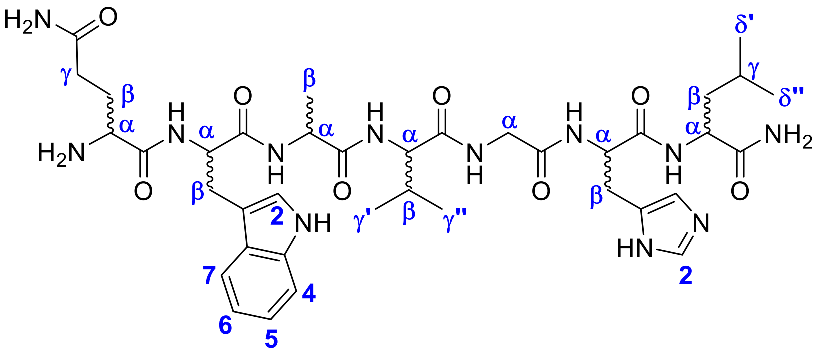

Figure S4. The [7-13] fragment of bombesin with labelling for residue-specific NMR assignments.

*L-[7, 13]BBN (**L-2**):* ***L*-2** was obtained by automated solid phase peptide synthesis on a Biotage Initiator Alstra+ microwave instrument , using 9-fluorenylmethoxycarbonyl (Fmoc) chemistry and Rink Amide MBHA Resin (500 mg, 0.59 mmol/g loading assumed). The side chains of histidine, glutamine and tryptophan were protected with trityl or *tert*-butoxycarbonyl, respectively (Fmoc-His(Trt)-OH, Fmoc-Gln(Trt)-OH and Fmoc-Trp(Boc)-OH). For each amino acid 3 equivalents were used and each coupling step was performed with HBTU (0.6 M in DMF), HOBt (3 M in DMF) and DIEA (2M in DMF) and the removal of the Fmoc group was performed with piperidine 20% (v/v) in DMF. After the coupling of all the amino acids in the sequence, the obtained peptide was cleaved from the resin and fully deprotected using a cocktail of TFA/H_2O/TIPS (95:2.5:2.5). The resin was mixed with the cocktail (2 mL/100 mg of resin) and left for 3 h under stirring. The solution was filtered and the peptide was precipitated with Et_2O. The white solid obtained was washed 3 times with Et_2O and centrifuged 3 times at 10000 rpm for 3 min. The white solid was freeze-dried overnight and purified by semi-preparative HPLC (method B, giving rise to the corresponding TFA salt).

^{1}H-NMR (500 MHz, 298 K, D_2O) δ = 8.53 (d, 1H, His-H2, *J* = 1.4 Hz), 7.62 (d, 1H, Trp-H4, *J* = 7.9 Hz), 7.50 (d, 1H, Trp-H7, *J* = 8.2 Hz), 7.28-7.23 (m, 2H, Trp-H2, Trp-H6), 7.21 (d, 1H, His-H4, *J* = 1.2 Hz), 7.17 (m, 1H, Trp-H5), 4.74 (dd, 1H, Trp-Hα, *J* = 8.8 Hz, *J* = 6.6 Hz), 4.66 (dd, 1H, His-Hα, *J* = 7.7 Hz, *J* = 6.7 Hz), 4.37-4.28 (m, 2H, Leu-Hα, Ala-Hα), 4.03 (d, 1H, Gln-Hα, *J* = 7.3 Hz), 3.99-3.87 (m, 3H, Gly-H2α, Val-Hα), 3.34 (dd, 1H, Trp-Hβ, *J* = 14.4 Hz, *J* = 6.4 Hz), 3.30-3.11 (m, 3H, Trp-Hβ, His-H2β), 2.43-2.33 (m, 1H, Gln-Hγ), 2.29-2.21 (m, 1H, Gln-Hβ), 2.11-2.00 (m, 3H, Gln-Hγ, Gln-Hβ, Val-Hβ), 1.71-1.52 (m, 3H, Leu-H2β, LeuHγ), 1.34 (d, 3H, Ala-CH_3, *J* = 7.2 Hz), 0.98 (d, 3H, Val-CH_3, *J* = 6.8 Hz), 0.96 (d, 3H, ValCH_3, *J* = 6.8 Hz), 0.92 (d, 3H, Leu-CH_3, *J* = 6.8 Hz), 0.86 (d, 3H, Leu-CH_3, *J* = 6.1 Hz).

ESI$^+$: m/z found: 405.2252 $[M+2H]^{2+}$,809.4429 $[M+H]^+$; calculated for $C_{38}H_{56}N_{12}O_8$: 808.4344.

*D-[7, 13]BBN (**D-2**):* ***D*-2** was obtained by automated solid phase peptide synthesis on an Activotec Activo-P11 automated peptide synthesizer using 9-fluorenylmethoxycarbonyl (Fmoc) chemistry and Rink Amide MBHA Resin (250 mg, 0.59 mmol/g loading assumed). The side chains of histidine, glutamine and tryptophan were protected with trityl or *tert*-butoxycarbonyl, respectively as before. The first residue was attached to the resin manually as follows: the resin was placed in a 10 mL plastic reactor and was swollen in DMF for 15 min. The Fmoc protection was removed by treatment with 20% piperidine/DMF (3 cycles: 12 min, then 2 x 3 min). After washing thoroughly with DMF to remove traces of piperidine, the deprotection was confirmed by a positive Kaiser test. Fmoc-Leu-OH (209 mg, 0.59 mmol, 4 eq) in DMF (2.5 ml) was preactivated by treatment with *N,N'*-diisopropylcarbodiimide (DIC, 94 µL, 0.96 mmol, 4 eq.) and HOBt (81 mg, 0.59 mmol, 4 eq). After 3 h, this solution was added to the deprotected resin, followed by DIEA (157 µL, 0.96 mmol, 6 eq) and the vessel was shaken for 45 min. The vessel was then transferred to the automated synthesiser, and the reaction vessel was fitted with a heating jacket (60 °C). Subsequent Fmoc deprotection steps were performed using 20% piperidine/DMF (3 ml, 5 + 10 min). Amino acid couplings were performed with 3 eq of each Fmoc-protected amino acid, 3 eq. of PyBOP (0.2 M in DMF) and 6 eq. of DIEA (0.4 M in DMF). After the synthesis, cleavage of the peptide from the resin was performed

by mixing a small amount of the resin with a mixture of TFA/H_2O/TIPS (95:25:25) for 2 h. The resin beads were removed by filtration and the peptide was precipitated by addition of anhydrous Et_2O. The white precipitate was collected by centrifugation and washed with further Et_2O. The solvent was removed, and the product was obtained as a white solid.

ESI$^+$: m/z found: 831.4277 $[M+Na]^+$; calculated for $C_{38}H_{56}N_{12}NO_8$: 831.4236.

Synthesis of 2,5-dioxopyrrolidin-1-yl 2-(7-(20-((1H-imidazol-5-yl)methyl)-8-((1H-indol-3-yl)methyl)-5-(3-amino-3-oxopropyl)-23-carbamoyl-1-(4-iodophenyl)-14-isopropyl-11,25-dimethyl-3,6,9,12,15,18,21-heptaoxo-4,7,10,13,16,19,22-heptaazahexacosan-2-yl)-1,3,6,8-tetraoxo-3,6,7,8-tetrahydrobenzo[lmn][3,8]phenanthrolin-2(1H)-yl)-3-(4-iodophenyl)propanoate (L-3 or D-3) and 2,2'-((2,2'-(1,3,6,8-tetraoxo-1,3,6,8-tetrahydrobenzo[lmn][3,8]phenanthroline-2,7-diyl)bis(3-(4-iodophenyl)propanoyl))bis(azanediyl))bis(N^1-(14-((1H-imidazol-5-yl)methyl)-17-carbamoyl-1-(1H-indol-3-yl)-8-isopropyl-5,19-dimethyl-3,6,9,12,15-pentaoxo-4,7,10,13,16-pentaazaicosan-2-yl)pentanediamide) (L-4 or D-4)

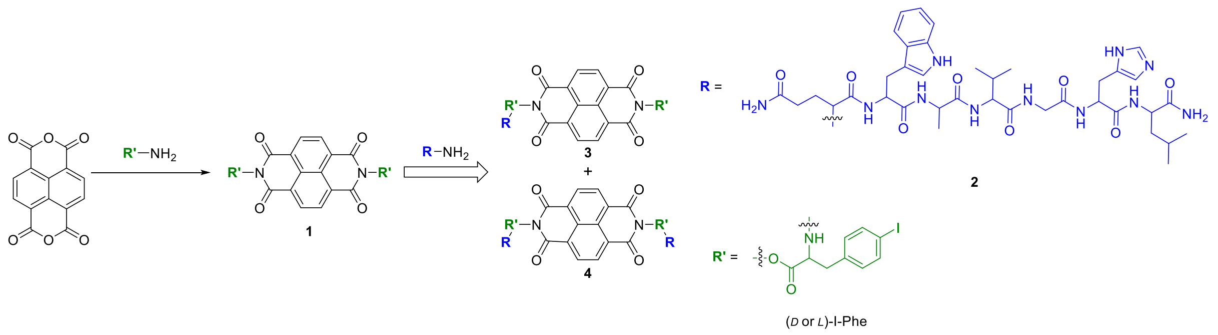

BBN \ NDI	L-1	D-1
**L-2**	**L-3 & L-4**	*L-D*
**D-2**	*D-L*	**D-3 & D-4**

Figure S6. Overview of the formation of the homochiral ***L-3***, ***L-4, D-3*** and ***D-4*** derivatives. The synthesis of the corresponding crossed BBN-NDI derivatives (denoted "*D-L*", "*L-D*") was also attempted on an analytical scale and the products analysed by mass spectrometry (see Section 4).

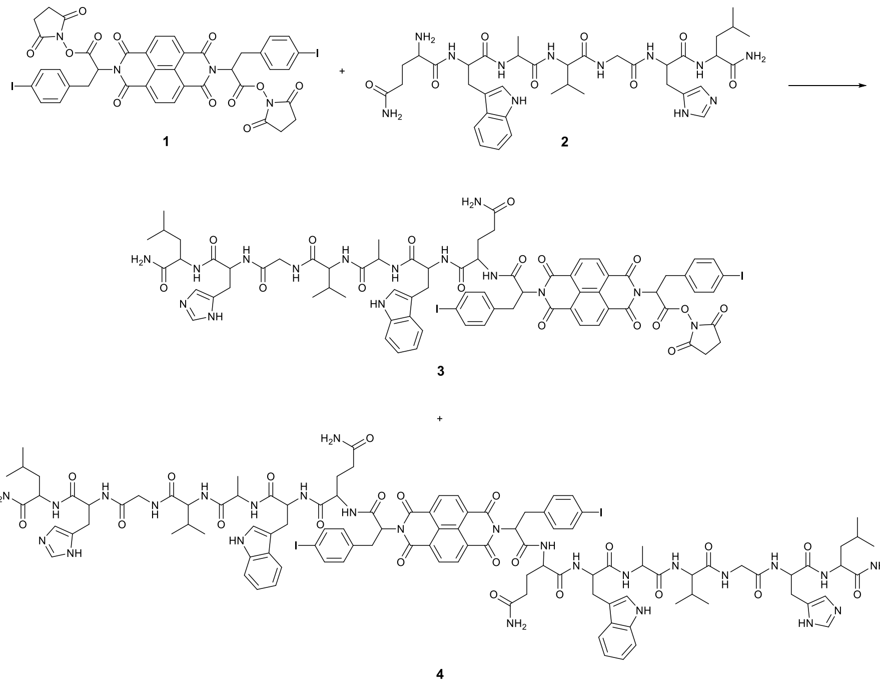

Scheme S2. Reaction pathway for the mono- and bis-peptide and amino acid-tagged NDI-conjugates, ***L*-3** or ***D*-3, *L*-4** or ***D*-4** respectively.

In a pressure-tight microwave vessel, ***L*-2** (6 mg, 0.07 mmol) and ***L*-1** (110 mg, 0.14 mmol) were dissolved in anhydrous DMF (5 mL). To the resulting mixture, Et_3N (0. 20 mL, 14.3 mmol) was added and the reaction mixture was heated at 70 ^oC for 45 min in a Biotage Initiator microwave system. The solvent was partially removed under reduced pressure and a light brown precipitate was obtained after the addition of Et_2O. The bright brown solid was washed with Et_2O and centrifuged 3 times at 10000 rpm for 3 min. The bright brown solid was freeze-dried overnight and purified by HPLC (Method B). The same procedure was applied to the coupling between ***D*-2** and ***D*-1**. (Yields after semi-prep separation and purification: 1.4 mg, 1.2% for ***L*-3**; 6.88 mg, 4.1% for ***L*-4**; 1.5 mg, 1.3% for ***D*-3**; 1.8 mg, 1.1% for ***D*-4**).

***L*-3**: ESI^+-TOF (CH_3OH): m/z found: 1702.3797 $[M+H]^+$, 1724.3672 $[M+Na]^+$; calculated for $C_{74}H_{77}I_2N_{15}O_{17}$: 1701.3711.
***D*-3**: ESI^+-TOF (CH_3OH): m/z found: 1702.3811 $[M+H]^+$, 1724.3710 $[M+Na]^+$; calculated for $C_{74}H_{77}I_2N_{15}O_{17}$: 1701.3711.
***L*-4**: ESI^+-TOF (CH_3OH): m/z found: 1198.4002 $[M+2H]^{2+}$, 2395.7806 $[M]^+$; calculated for $C_{108}H_{128}I_2N_{26}O_{22}$: 2394.7786.
***D*-4**: ESI^+-TOF (CH_3OH): m/z found: 1198.4050 $[M+2H]^{2+}$, 2395.8557 $[M]^+$; calculated for $C_{108}H_{128}I_2N_{26}O_{22}$: 2394.7786.
HPLC (method A): Rt = 37.1 min (***L*-3** and ***D*-3**), 36.2 min (***L*-4** and ***D*-4**).

Additionally, the analogous protocol was applied for the coupling reaction between ***L*-2** and ***D*-1** to give rise to the [L-BBN-D-INDI-OSu] conjugate (denoted ***L-D***). Similarly the reaction between ***L-1*** and ***D-2*** gave rise to [D-BBN-L-INDI-OSu] (denoted ***D-L***). These additional isomers of the desired compounds were only obtained on an analytical scale, and selected data for the separation and analysis of the coupling reaction is also included below.

In all cases, alternative syntheses routes were tested, involving 24 h stirring in DMF with reaction temperature rising from 0 ^oC to r.t. These led to the same products being formed, in all cases, and in comparable yields post-purification.

Optiised synthesis of 4,4-Difluoro-8-(4'-carboxyphenyl)-1,3,5,7-tetramethyl-4-bora-3a,4a-diaza-s-indacene (8-carboxyphenyl BODIPY)

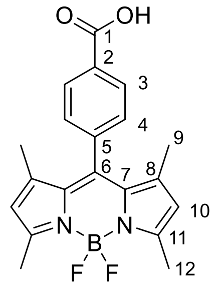

8-carboxyphenyl BODIPY

2,4-Dimethylpyrrole (5.00 g, 52.6 mmol) and 4-formylbenzoic acid (3.72 g, 22.5 mmol) were dissolved in CH_2Cl_2 (2 L) and 10 drops of TFA added. The reaction mixture was allowed to stir at room temperature for 1.5 h. 2,3-Dichloro-5,6-dicyano-1,4-benzoquinone (DDQ) (5.098 g, 22.5 mmol) was added portionwise followed by Et_3N (50 mL) and $BF_3 \cdot OEt_2$ (50 mL) added dropwise. The reaction was quenched by the addition of H_2O (1 L). The aqueous phase was extracted with CH_2Cl_2 (3x 600 mL). The organic fractions were collected, dried over $MgSO_4$ and the solvent removed under vacuum. The crude product was purified by flash column chromatography using CH_2Cl_2 / MeOH (0 – 20%) as eluent. After recrystallisation from THF/hexane, the product was obtained as a red solid (3.15 g, 38%).

^{1}H NMR (500 MHz, d_8-THF, 25 ºC): δ 8.19 (d, *J* = 8.2 Hz, 2H, H-3), 7.48 (d, *J* = 8.2 Hz, 2H, H-4), 6.03 (s, 2H, H-2, H-10), 2.49 (s, 6H, H-12), 1.38 (s, 6H, H-9).

^{13}C NMR (125 MHz, d_8-THF, 25 ºC): δ 167.2 (*C*OOH), 156.7 (C-11), 143.6 (C-5), 142.1 (C-8), 140.6 (C-6), 132.9 (C-7), 132.0 (C-2), 131.5 (C-3), 129.5 (C-4), 122.1 (C-10), 14.8, 14.7 (C-9, C-12). Mass spectrum: ESI-MS calc. for $C_{20}H_{18}BF_2N_2O_2$ $[M\text{-}H]^-$: 367.1429; found: 367.1435.

Elem. Anal. (%). Found (calc.) for $C_{20}H_{19}BF_2N_2O_2$: C, 64.05 (65.24); H, 5.18 (5.20); N, 7.21 (7.61).

IR (solid) ν (cm^{-1}): 2974, 1678, 1544, 1076, 1043, 972, 738.

HPLC (Method A): Rt (min) 10.22.

Synthesis of 2,5-dioxopyrrolidin-1-yl 4-(5,5-difluoro-1,3,7,9-tetramethyl-5H-4l4,5l4-dipyrrolo[1,2-c:2',1'-f][1,3,2]diazaborinin-10-yl)benzoate

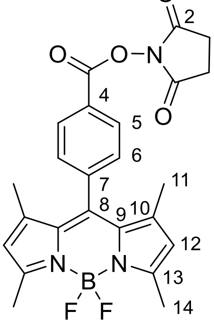

8-Carboxyphenyl BODIPY (0.100 g, 0.27 mmol), N-hydroxysuccinimide (0.031 g, 0.27 mmol) and EDC·HCl (0.107 g, 0.56 mmol) were dissolved in CH_2Cl_2 (60 mL). The reaction mixture was stirred at room temperature for 3 h. The solvent was removed under vacuum and the residue purified by flash

column chromatography using hexane / ethyl acetate (1:1) as eluent. The product was obtained as a crystalline orange solid (0.135 g, 99%).

^{1}H NMR (500 MHz, $CDCl_3$, 25 ºC): δ 8.27 (d, *J* = 8.4 Hz, 2H, H-4), 7.50 (d, *J* = 8.4 Hz, 2H, H-6), 6.01 (s, 2H, H-12), 2.94 (s, 4H, H-1), 2.56 (s, 6H, H-14), 1.38 (s, 6H, H-11).

^{13}C NMR (125 MHz, $CDCl_3$, 25 ºC): δ 169.3 (C-2), 161.4 (C-3), 156.5 (C-13), 143.0 (C-10), 142.2 (C-8), 139.4 (C-7), 131.4 (C-5), 130.8 (C-4), 129.2 (C-6), 125.9 (C-9), 121.8 (C-12), 25.8 (C-1), 14.9 (C-14), 14.8 (C-11).

Mass spectrum: ESI-MS calc. for $C_{25}H_{25}BF_2N_3O_5$ $[M-H]^-$: 496.1855; found: 496.1867.

Elem. Anal. (%). Found (calc.) for $C_{24}H_{22}BF_2N_3O_4$: C, 61.78 (61.96); H, 4.88 (4.77); N, 8.89 (9.03).

IR (solid): ν (cm^{-1}) 2921, 2852, 1802, 1778, 1737, 1542, 1065, 721.

HPLC (Method B): Rt (min) 10.64.

Synthesis of N-(2-aminoethyl)-4-(5,5-difluoro-1,3,7,9-tetramethyl-5H-4λ4,5λ4-dipyrrolo[1,2-c:2',1'-f][1,3,2]diazaborinin-10-yl)benzamide (5)

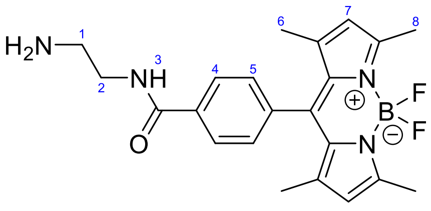

5

Method 1: A stirred solution of the preceding BODIPY OSu ester (110 mg, 0.22 mmol) in DMF (5 mL) was treated with DIEA (57 µL, 0.326 mmol) and the solution was cooled to 0 ºC in an ice bath. BOP (180 mg, 0.407 mmol) was added and the solution was stirred at 0 ºC for 30 min, then ethylenediamine (26 µL, 0.326 mmol) was added and the solution was allowed to warm to room temperature and stirred for 12 h. The DMF was removed under reduced pressure. The crude product was dissolved in $CHCl_3$ (25 mL) and washed with 1M HCl (20 mL), saturated aqueous sodium bicarbonate (100 mL), water (100 mL), brine (100 mL) and dried over anhydrous $MgSO_4$. The solvent was removed under reduced pressure and the crude residue was purified by silica gel chromatography using $CHCl_3$ / MeOH (0 – 5%) as eluant to give **5** (99 mg, 50%).

Method 2: A stirred solution of the preceding BODIPY-OSu ester (0.600 g, 1.29 mmol) and DIEA (2.5 mL, 14.4 mmol) in DMF (50 mL) at 60 ºC, was treated with ethylenediamine (1.06 mL, 14.2 mmol) added portionwise and the reaction mixture stirred at 60 ºC for 7 h. The solvent was removed under vacuum and the residue was dissolved in CH_2Cl_2 and washed with water (3 x 50 mL). The organic layer was dried over $MgSO_4$ and the solvent removed under vacuum. The purification was performed by automated flash column chromatography using CH_2Cl_2 / MeOH (0 – 10%) as eluent. The product was obtained as a red solid (0.699 g, 91%).

^{1}H NMR (500 MHz, DMSO-d_6) δ 8.67 (*t*, 1H, H-3, *J* = 5.6 Hz,), 8.06 (*d*, 2H, H-4, *J* = 7.8 Hz,), 7.50 (*d*, 2H, H-5, *J* = 7.7 Hz), 6.19 (s, 2H, H-7), 3.35 (*q*, 2H, H-2, *J* = 6.1 Hz), 2.79 (*t*, 2H, H-1, *J* = 6.3 Hz, 1H), 2.46 (s, 6H, H-6), 1.34 (s, 6H, H-8).

Synthesis of Compound L-6, (S)-N^1-((2S,5S,8S,14S,17S)-14-((1H-imidazol-5-yl)methyl)-17-carbamoyl-1-(1H-indol-3-yl)-8-isopropyl-5,19-dimethyl-3,6,9,12,15-pentaoxo-4,7,10,13,16-pentaazaicosan-2-yl)-2-(7-(2-(4-(5,5-difluoro-1,3,7,9-tetramethyl-5H-4λ^4,5λ^4-dipyrrolo[1,2-c:2',1'-f][1,3,2]diazaborinin-10-yl)benzamido)ethyl)-1,3,6,8-tetraoxo-3,6,7,8-tetrahydrobenzo[lmn][3,8]phenanthrolin-2(1H)-yl)pentanediamide

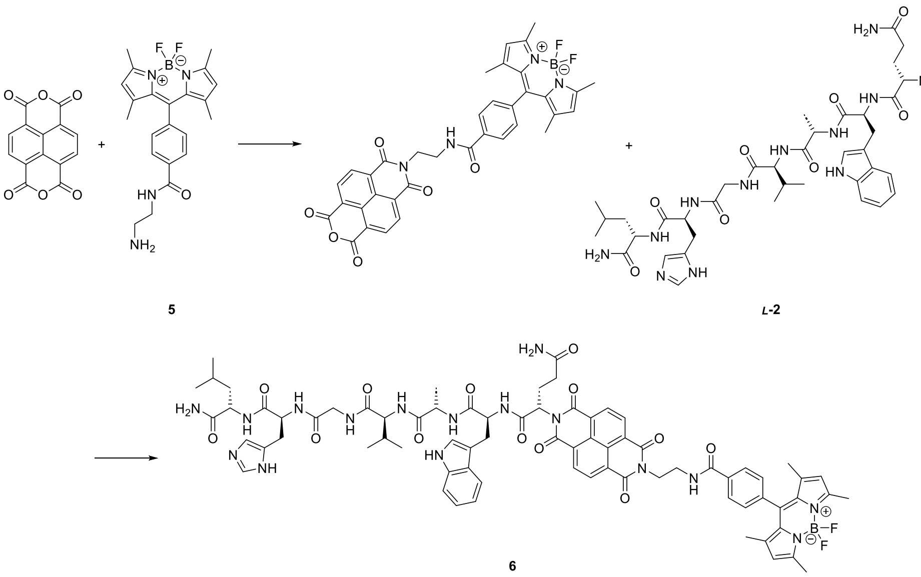

Scheme S3. Pathway for the microwave-driven synthesis of compound ***L*-6**.

In a pressure-tight microwave vessel, NDA (42 mg, 0.16 mmol) and **5** (50 mg, 0.12 mmol) were dissolved in anhydrous DMF (1 mL) and Et_3N (20 µL). The resulting mixture was sonicated for 5 min. When it was homogenised, the brown solution was heated in the microwave system at 40 ^oC for 5 min and then at 140 ^oC for further 5 min. The solvent was removed by rotary evaporation and the resulting light brown solid was obtained by automated flash chromatography (silica gel, gradient from 20% MeOH in DCM to 100% MeOH). This solid (44 mg, 0.06 mmol) was solubilised in anhydrous DMF (2 mL) with of ***L*-2** (55 mg, 0.07 mmol) and Et_3N (10 µL, 70 µmol). The mixture was heated at 70 ^oC for 1 h in the microwave system. The obtained dark red solution was washed with Et_2O and centrifuged 3 times at 10000 rpm for 3 min. The dark solid was freeze-dried overnight and purified by semi-preparative HPLC (69.2 mg, 30% yield).

ESI$^+$-TOF (CH_3OH): m/z found: 1451.6278 $[M+H]^+$; calculated $C_{74}H_{81}BF_2N_{16}O_{13}$: 1450.6230.

HPLC (method A): Rt = 40.2 min.

4. HPLC separations and Mass spectrometry analyses

(A) *Optimised HPLC chromatography separations of* ***L*-3**, ***L*-4**, ***D*-3**, ***D*-4** and ***L*-6**

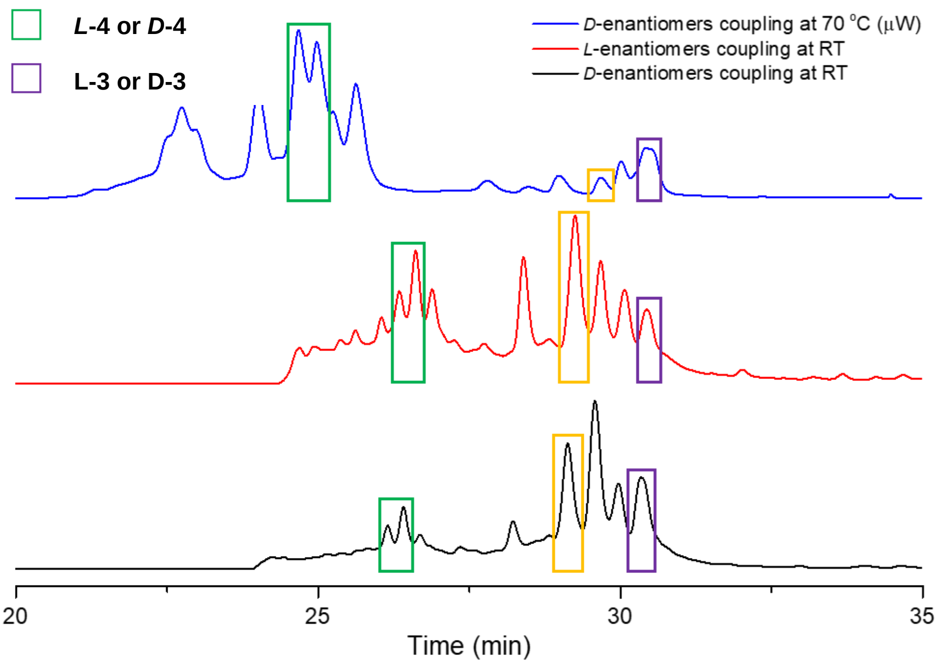

Figure S7. Comparative semi-preparative HPLC chromatograms of the µW-assisted synthesis of ***D*-3** and ***D*-4** (in blue), the room temperature syntheses of ***L*-3** and ***L*-4** (in red) and room temperature synthesis of ***D*-3** and ***D*-4** (in black). The fractions of ***L*-4** (or ***D*-4**) are selected in green; the purple rectangles display the fractions of ***L*-3** (or ***D*-3)**, which were then separated, collected and analysed, (Method B).

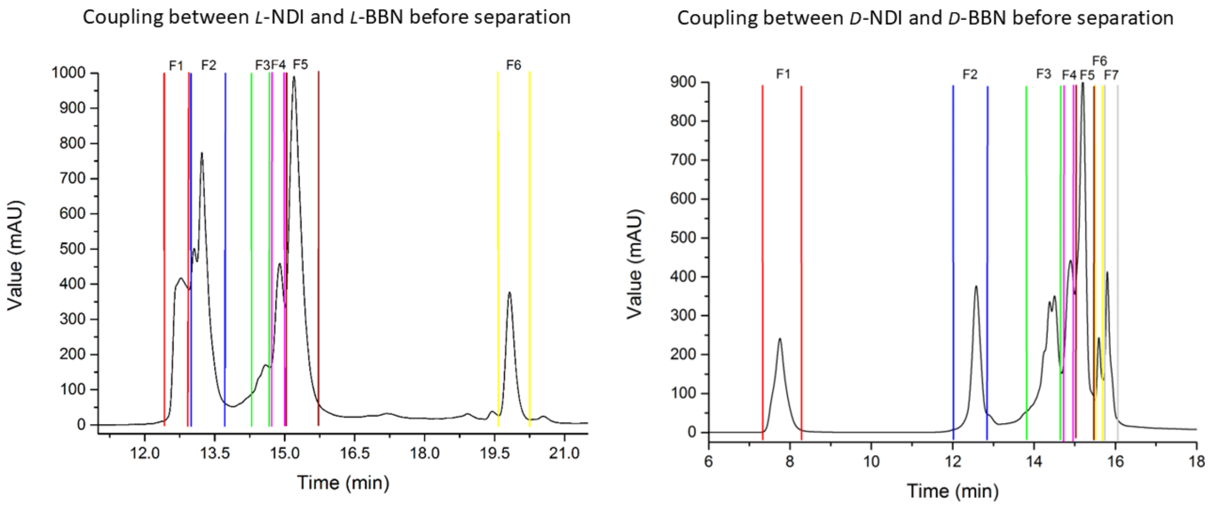

(a)

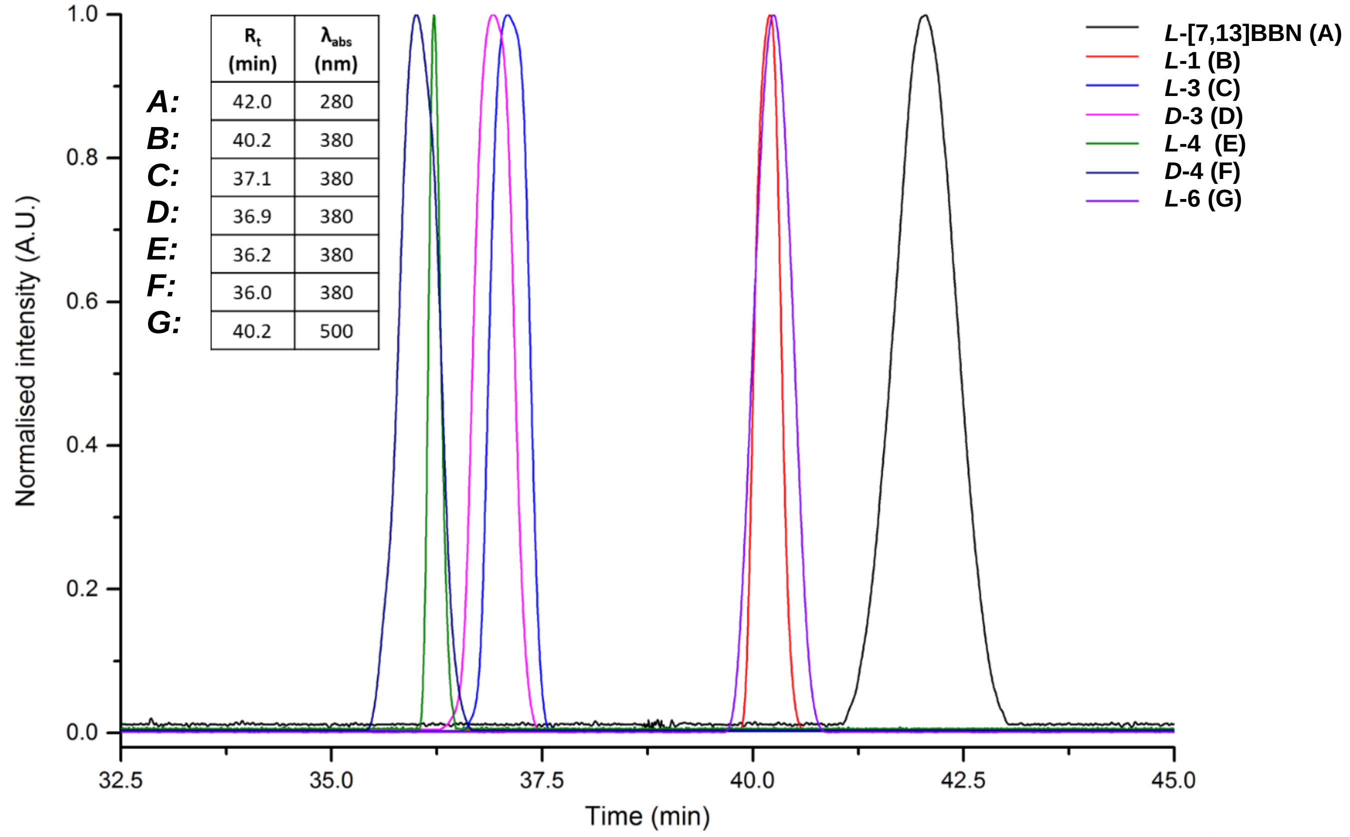

(b)

Figure S8. (a) Comparative semi-prep HPLC of the optimised coupling reactions between ***L*-[7,13]BBN** and ***L*-1** and corresponding coupling reactions between ***D*-[7,13]BBN** and ***D*-1**; (b) Analytical HPLC chromatograms of ***L*-[7,13]BBN** (**A,** black), ***L*-1** (**B,** red), ***L*-3** (**C,** blue), ***D*-3** (**D,** purple), ***L*-4** (**E,** green), ***D*-4** (**F,** navy blue) and **L-6** (**G,** dark purple). The inset table shows the corresponding retention times and the wavelengths of the recorded chromatograms (Method A).

(B) D-NDI-L-Bombesin Semi-prep HPLC traces and mass spectrometry of isolated species type (L-D):

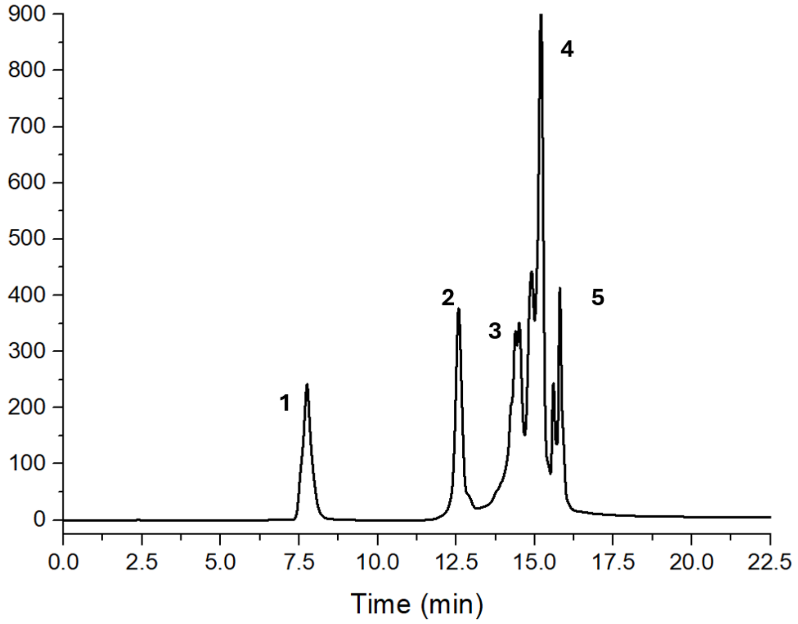

Figure S9. HPLC trace of the D-NDI-L-Bombesin reaction mixture prior to semi-prep HPLC separation (carried out using Method B).

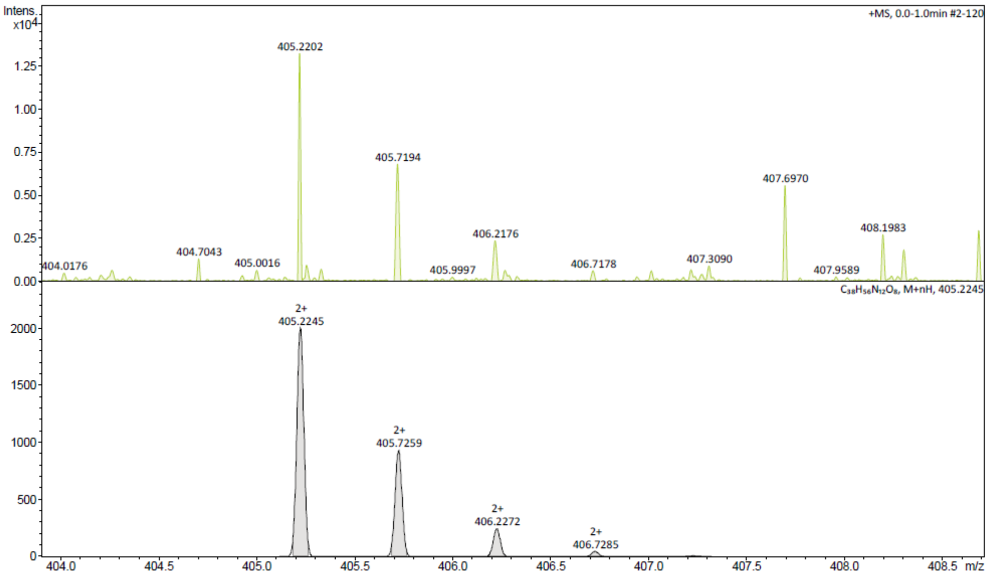

Figure S10. Mass spectrum of the isolated HPLC fraction 3 (L-[7,13]-bombesin).

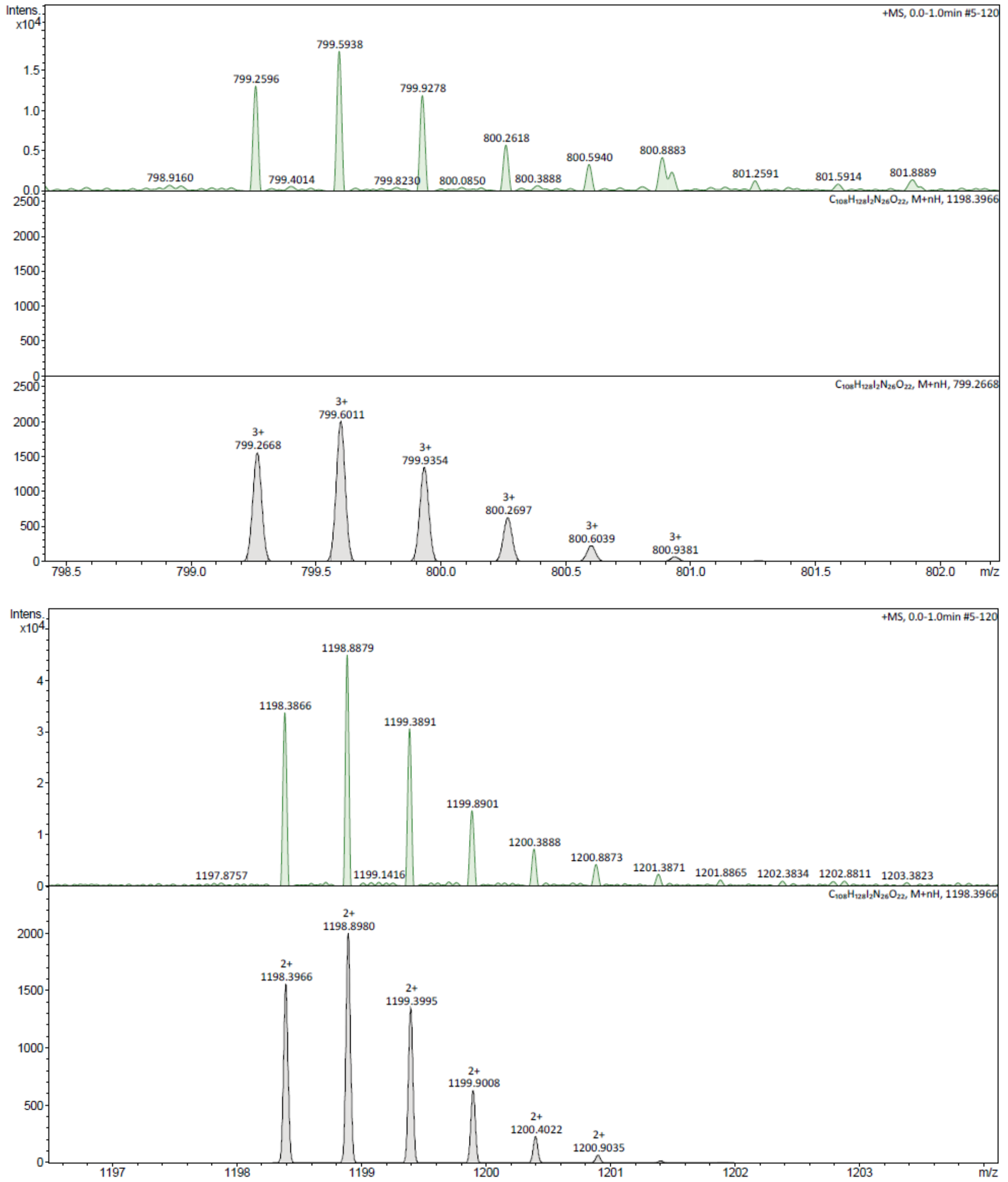

Figure S11. Mass spectra of the HPLC fraction 4, corresponding to D-NDI-[L-BBN]$_2$

.

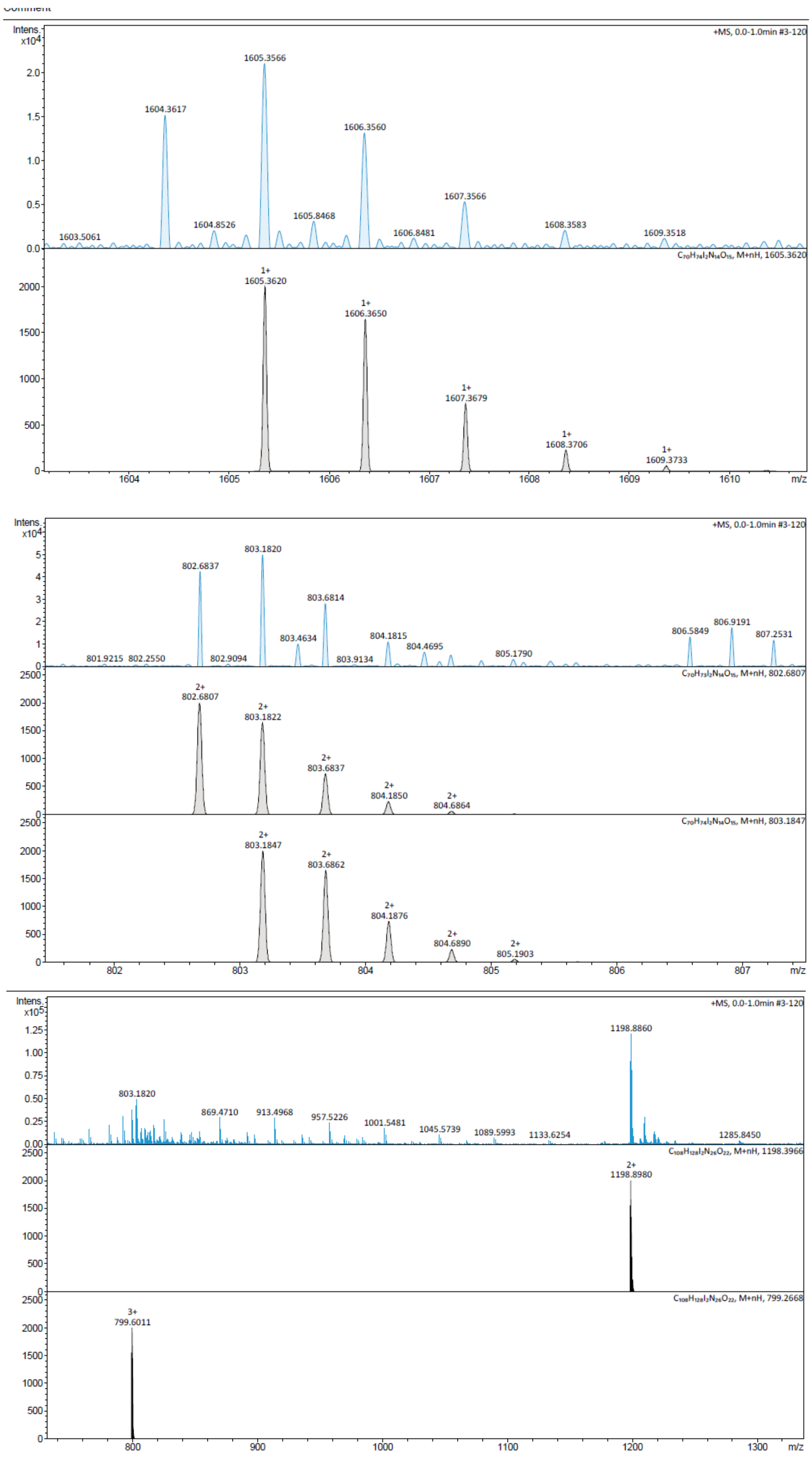

Figure S12. Mass spectra of the HPLC fraction 5 assignable to (OSu)-D-NDI-L-BBN and D-NDI-[L-BBN]$_2$

(C) L-NDI-D-Bombesin Semi-prep HPLC traces and mass spectrometry of isolated species type (D-L):

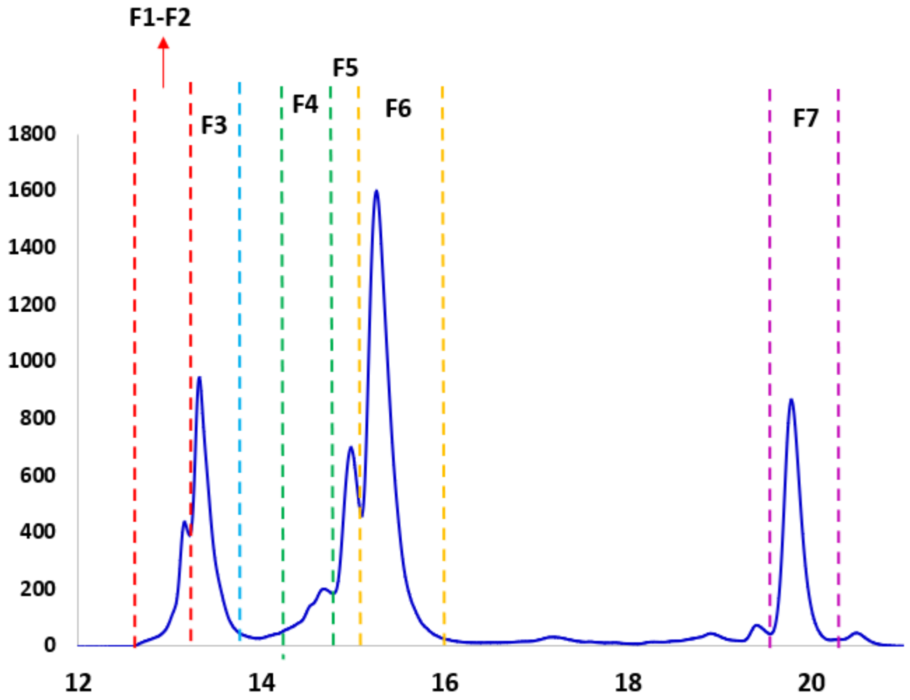

Figure S13. HPLC trace of the L-NDI-D-Bombesin crude product prior to semi-prep HPLC separation from starting materials (using Method B).

F1-F2

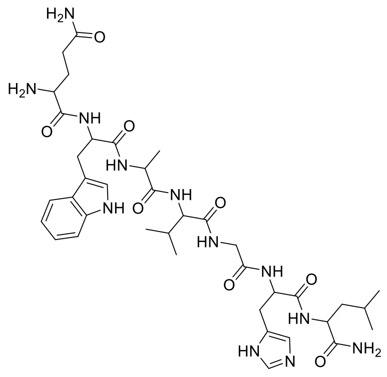

D-BBN

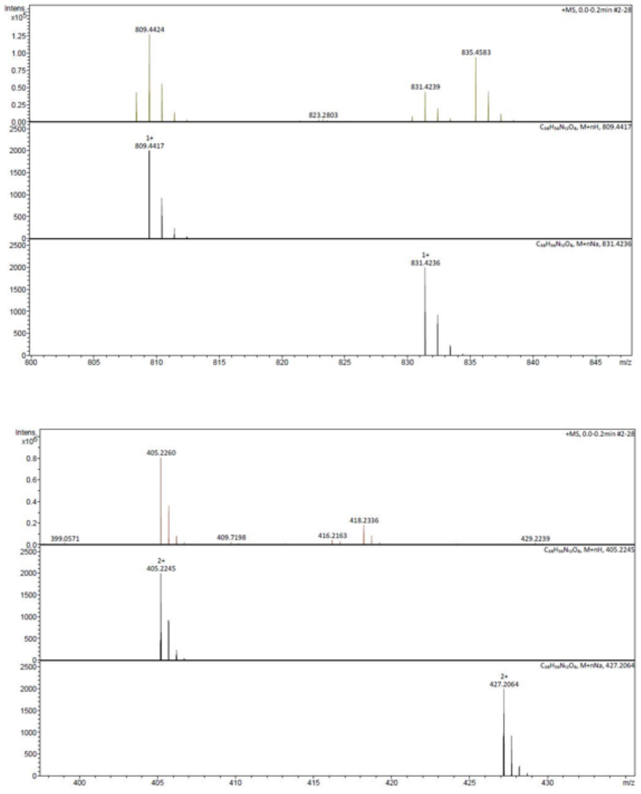

Figure S14. Mass spectrum of the HPLC fractions F1 and F2 (assignable to isolated D-[7,13]BBN)

F3

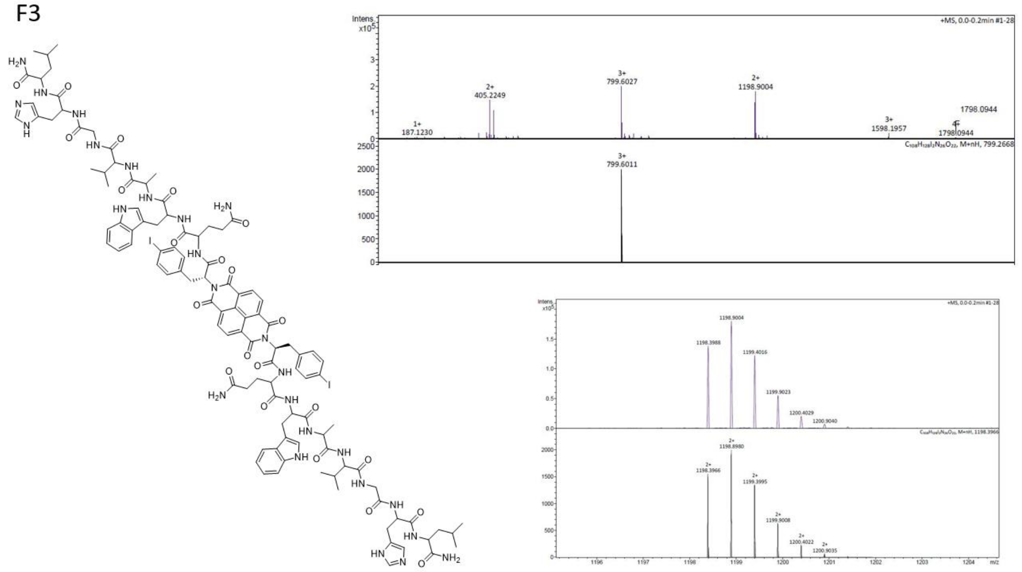

Figure S15. Mass spectrum of the HPLC fraction F3 (D-BBN-L-NDI-D-BBN)

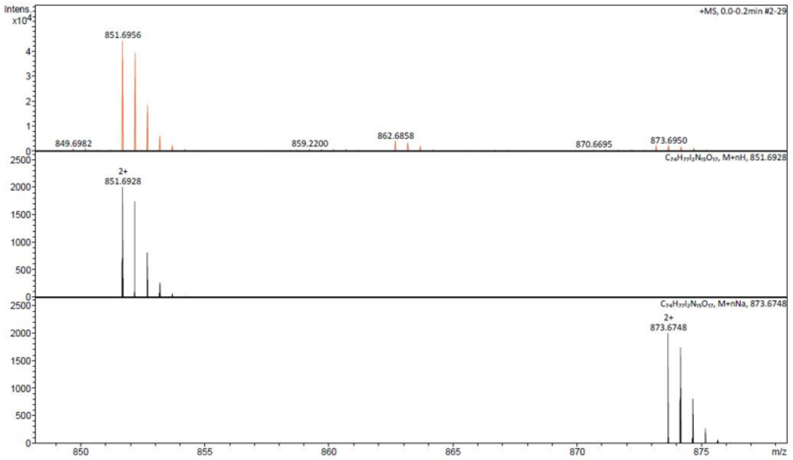

Figure S16. Mass spectrum of the HPLC fraction F4 (L-NDI-OSu-D-BBN)

F5

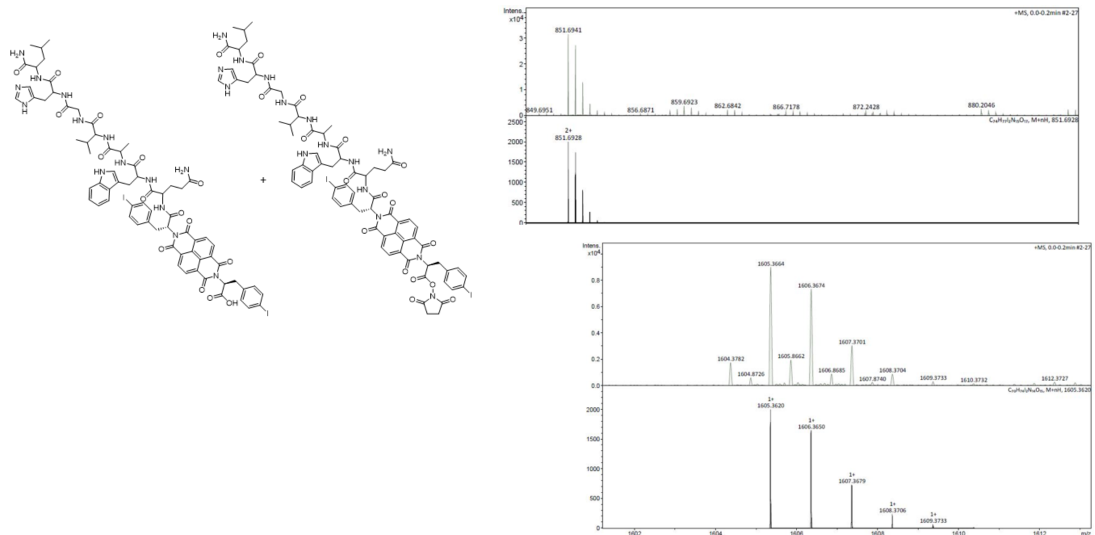

Figure S17. Mass spectrum of the HPLC fraction F5 (L-NDI-D-BBN + L-NDI-OSu-D-BBN)

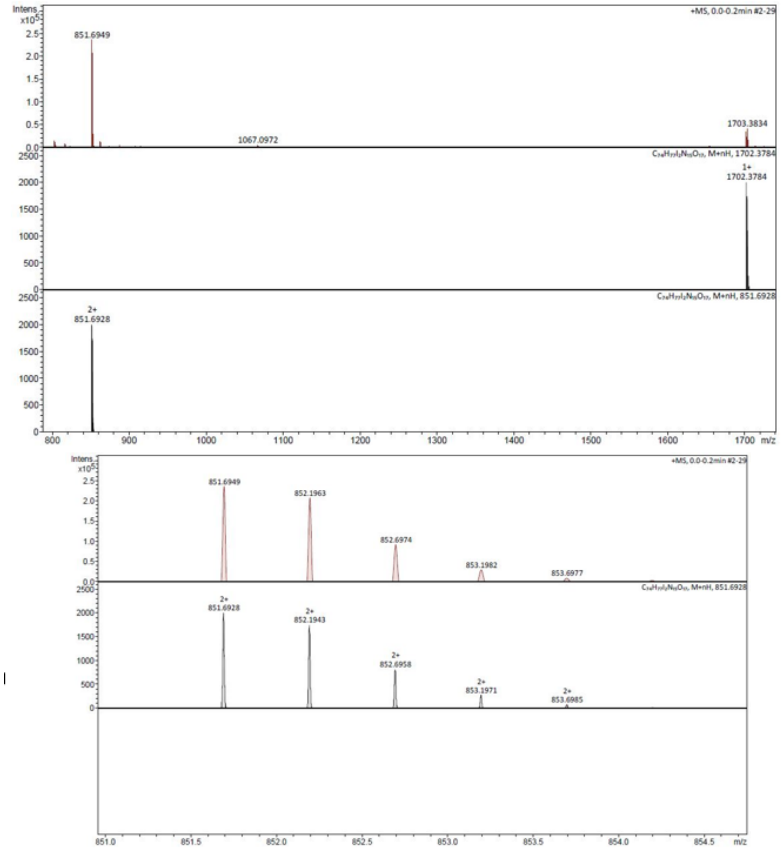

Figure S18. Mass spectrum of the HPLC fraction F6 (L-NDI-D-BBN)

F7

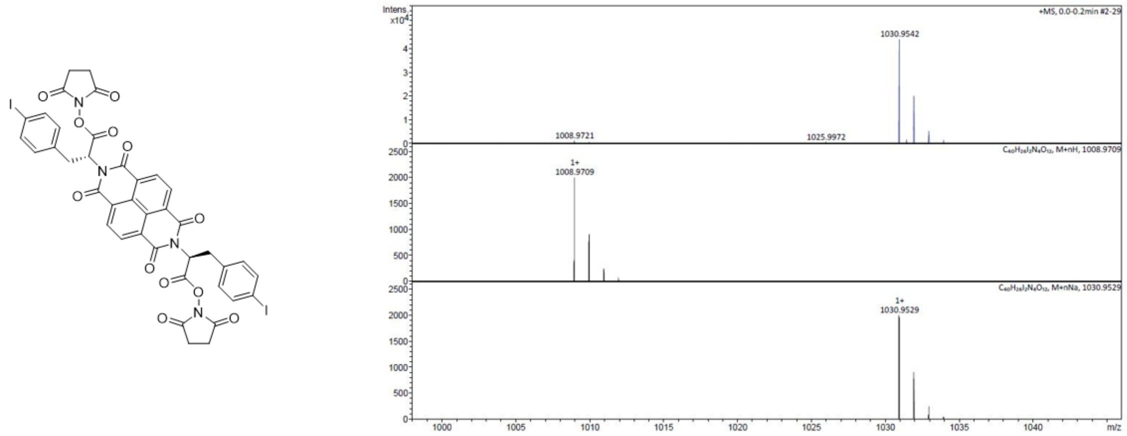

Figure S19. Mass spectrum of the HPLC fraction F7 (L-NDI-OSu)

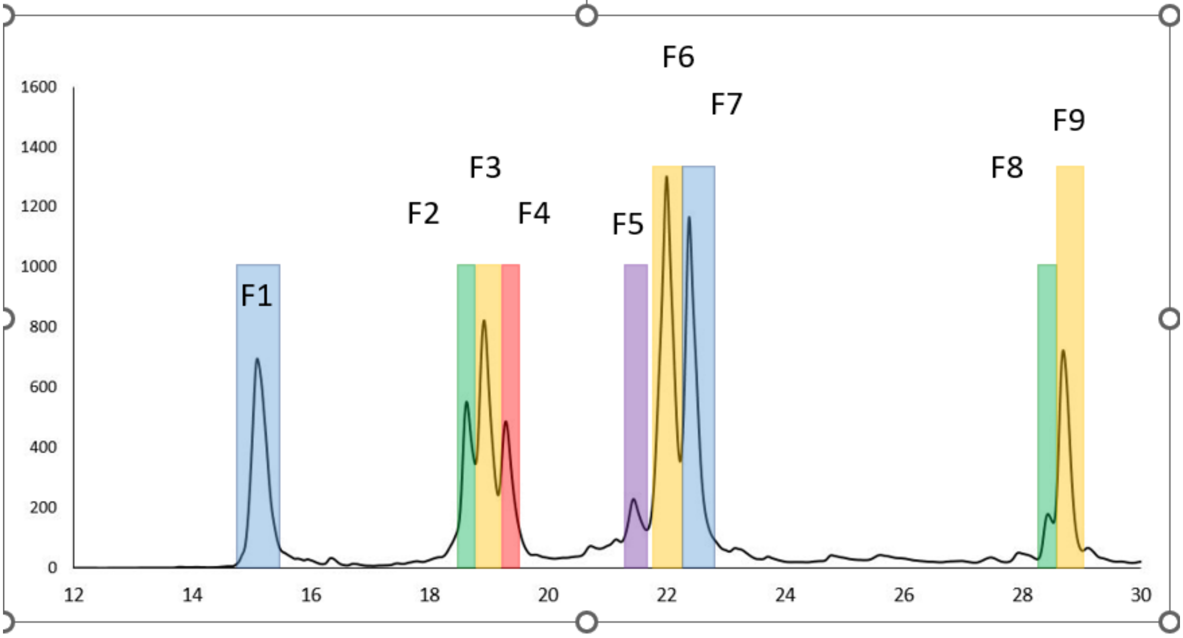

Figure S20. HPLC trace of the D-NDI-D-Bombesin crude mixture prior to separation (Method B).

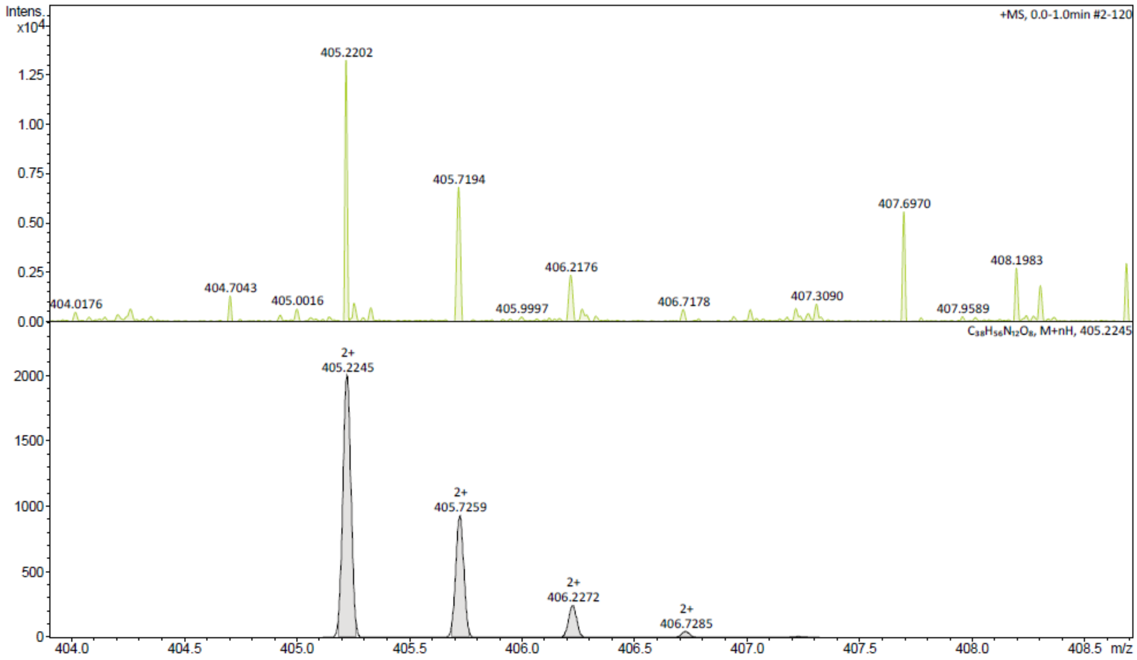

Figure S21. Mass spectrum of the HPLC fraction 1 (D-[7,13]-Bombesin).

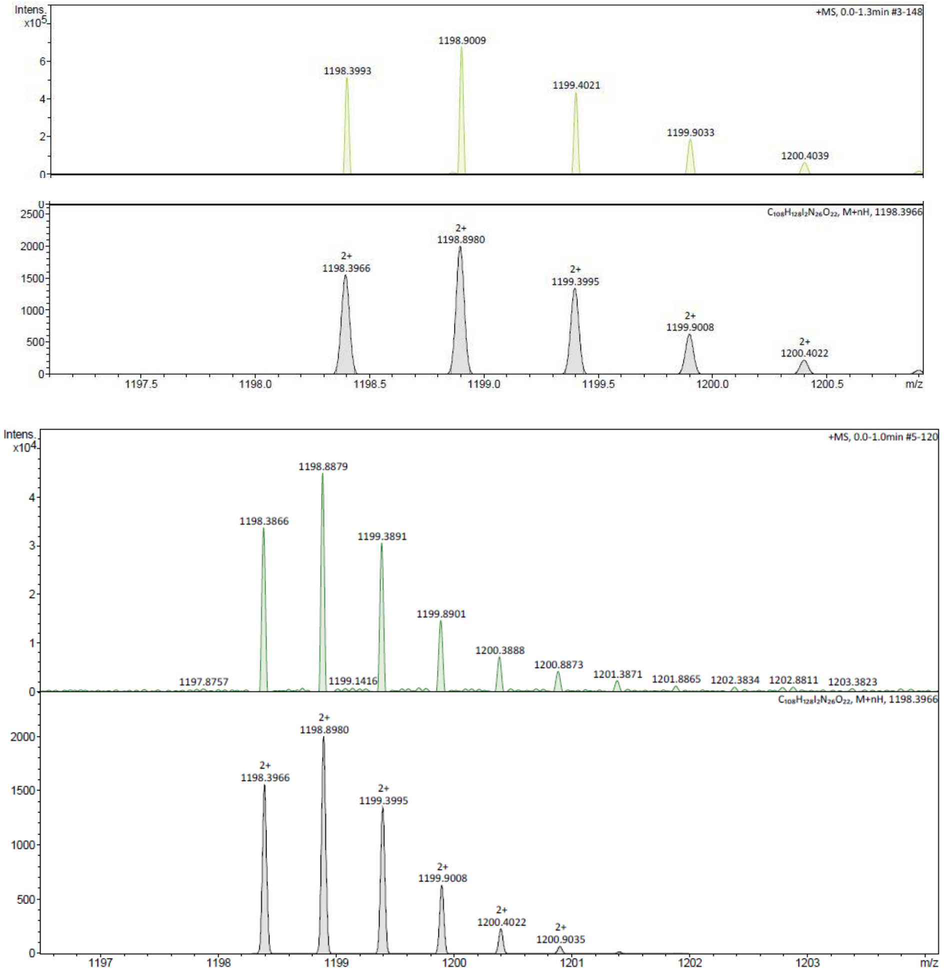

Figure S22. Mass spectra of the HPLC fraction 3, assignable to compound ***D-4***

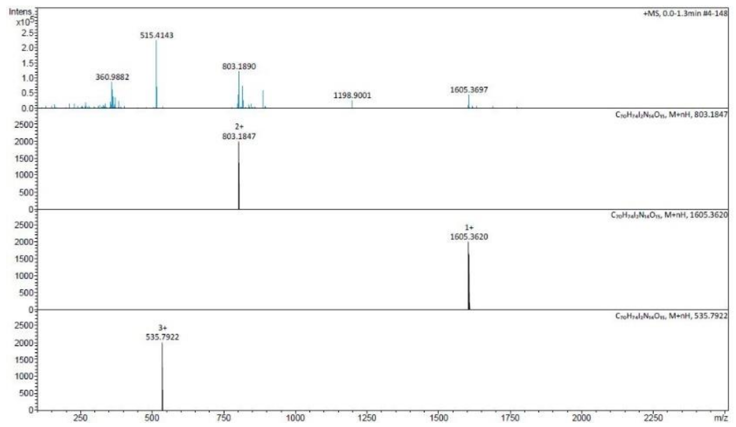

(i)

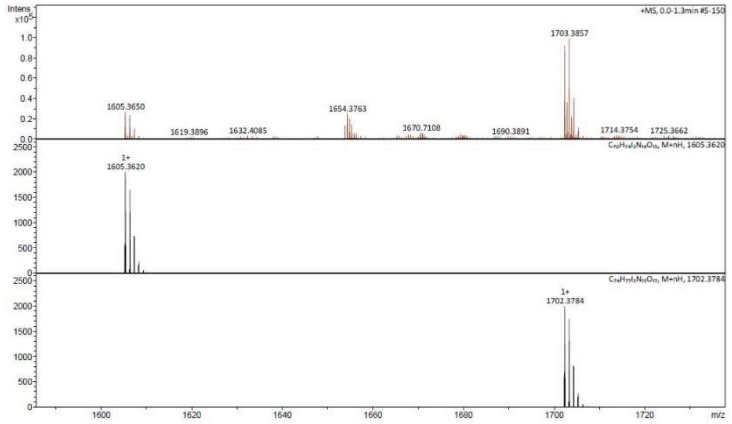

(ii)

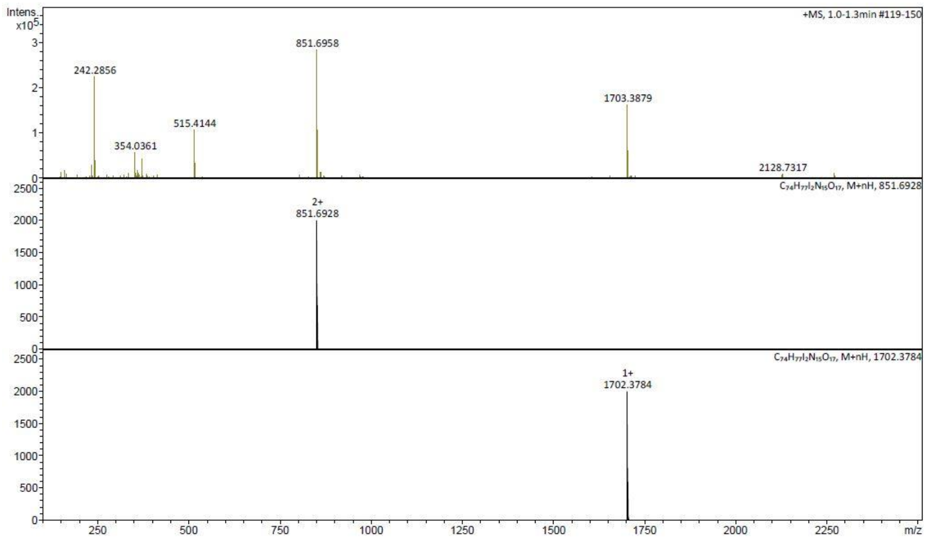

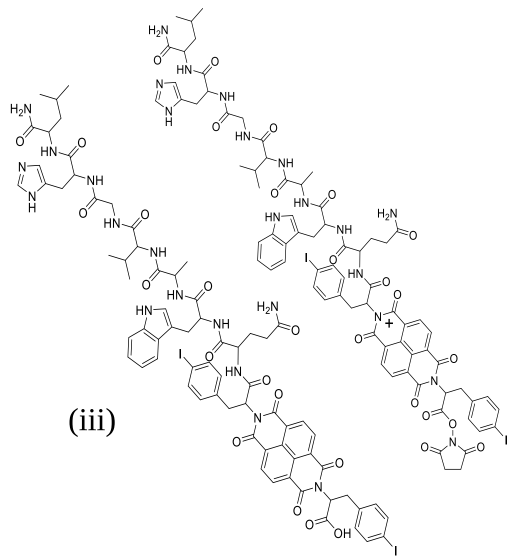

(iii)

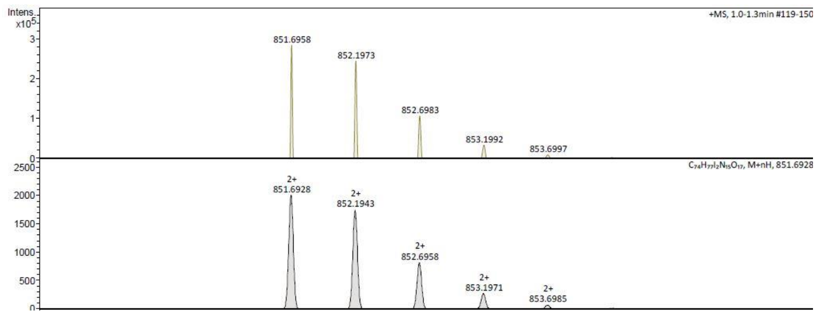

(iv)

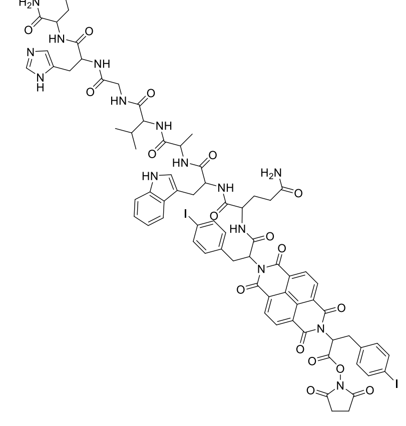

Figure S23. Mass spectra of the HPLC fractions 5 (i), 6 (ii) and 7 (iii-iv), assignable to compound ***D-3*** where fragments are showing presence as well as absence of the OSu moieties.

(E) Futher Mass spectrometry details and fragment assignments:

bis(2,5-Dioxopyrrolidin-1-yl) 2,2'-(1,3,6,8-tetraoxo-1,3,6,8-tetrahydrobenzo [lmn][3,8]phenanthroline-2,7-diyl)bis(3-(4-iodophenyl)propanoate) (L-1)

ESI$^+$-TOF (CH_3OH): m/z found: 1008.9721 [M+H]$^+$, 1030.9542 [M+Na]$^+$; calculated for $C_{32}H_{20}I_2N_{12}O_8$: 1007.9637.

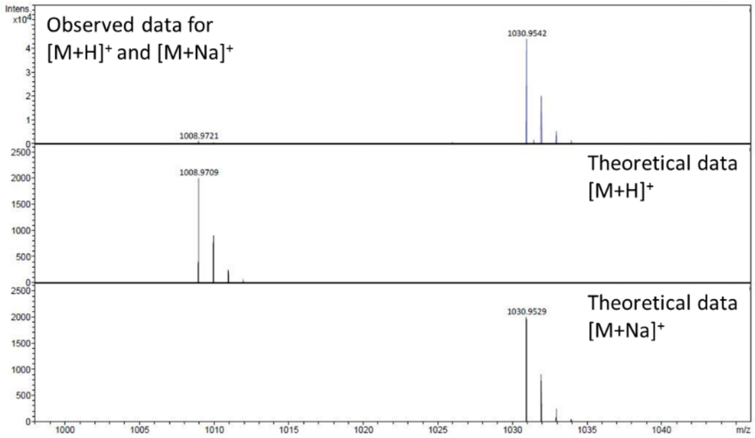

Figure S24. Observed (above) and calculated (below) isotopic patterns of ***L*-1** for [M+H]$^+$ and [M+Na]$^+$, respectively.

[7, 13]-Bombesin peptide (L-2 or D-2)

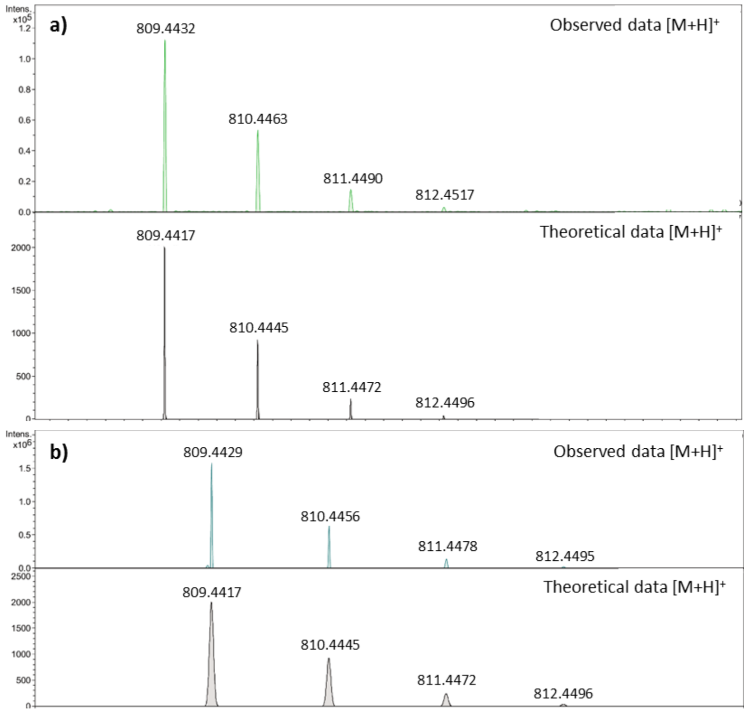

Figure S25. Mass spectrometry analysis of a) L-[7, 13-]bombesin ***L-2*** and b) D-[7, 13]-bombesin ***D-2***. The observed data of the [M+H]+ ion is shown on top, whilst its simulated isotopic pattern is below Figures show the observed and theoretical isotopic patterns for the ion [M+H]$^+$ of L- and D-, respectively. Both detected patterns fit within 5 mDa error with the simulated data.

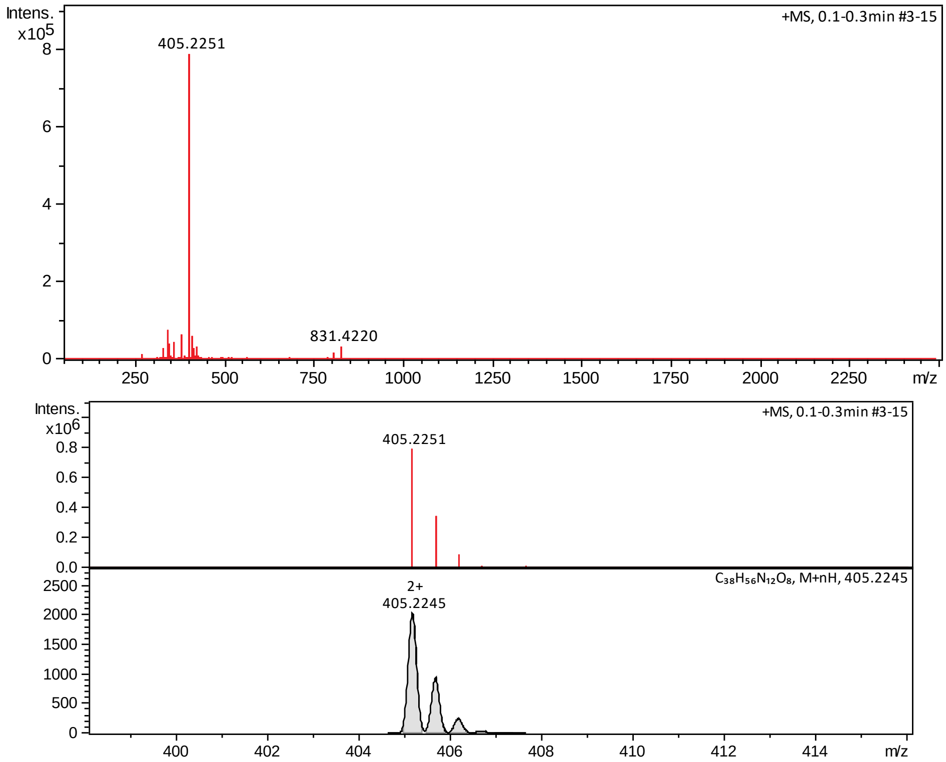

Figure S26. ESI-MS spectrum of the purified L-[7, 13]-bombesin fragment ***L*-2**.

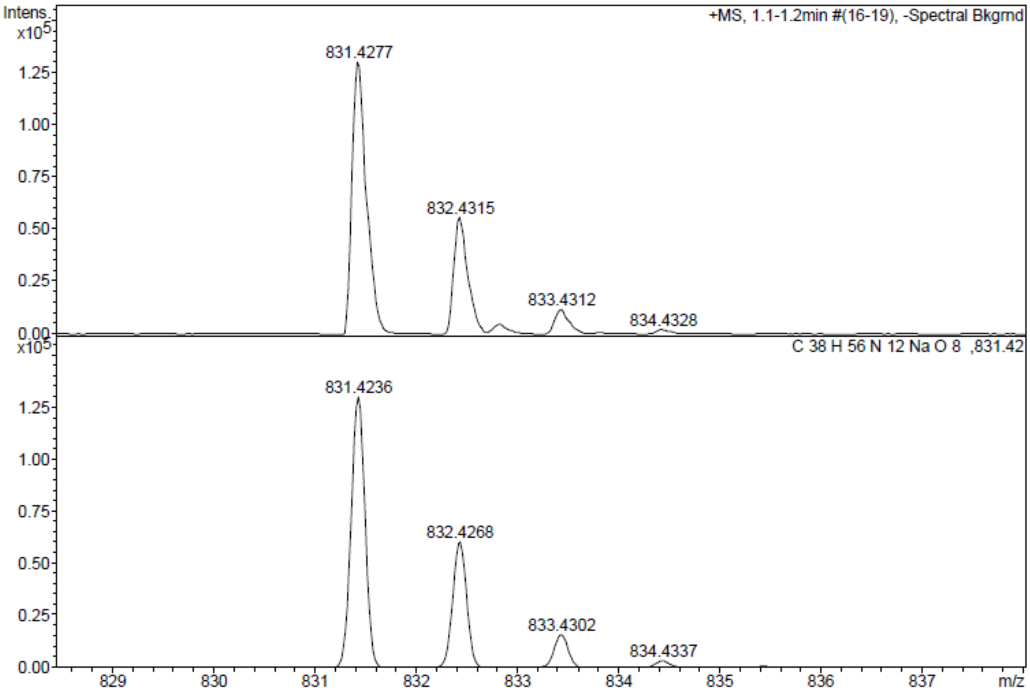

Figure S27. ESI-MS spectrum of the D-[7, 13]-bombesin fragment ***D*-2**.

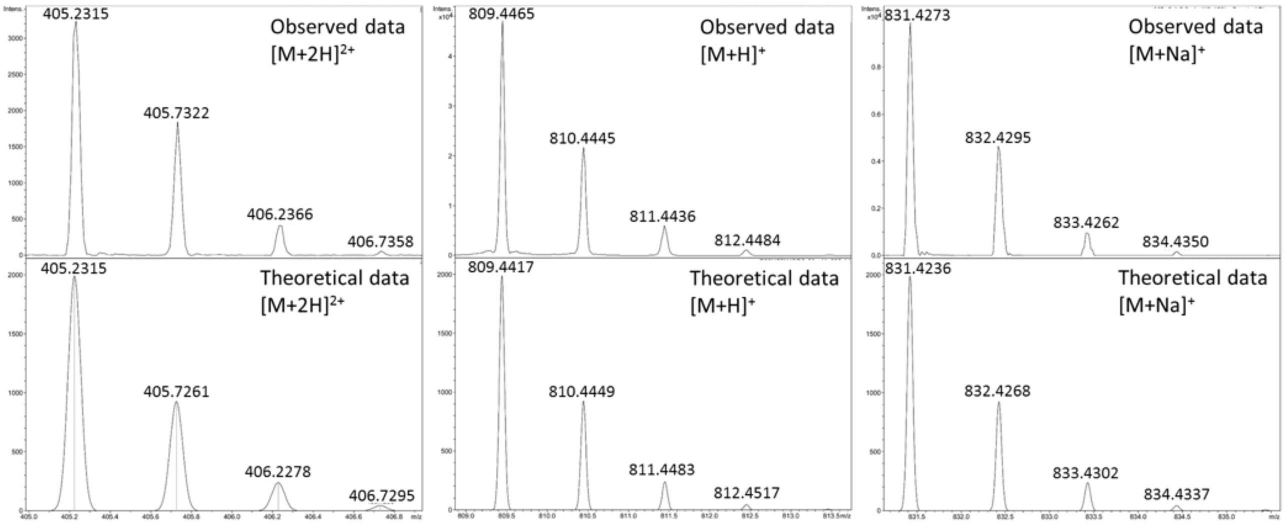

Figure S28. Observed (above) and calculated (below) isotopic patterns of ***L*-2** for $[M+2H]^{2+}$, $[M+H]^{+}$ and $[M+Na]^{+}$, respectively.

2,5-Dioxopyrrolidin-1-yl 2-(7-(20-((1H-imidazol-5-yl)methyl)-8-((1H-indol-3-yl)methyl)-5-(3-amino-3-oxopropyl)-23-carbamoyl-1-(4-iodophenyl)-14-isopropyl-11,25-dimethyl-3,6,9,12,15,18,21-heptaoxo-4,7,10,13,16,19,22-heptaazahexacosan-2-yl)-1,3,6,8-tetraoxo-3,6,7,8-tetrahydrobenzo[lmn][3,8]phenanthrolin-2(1H)-yl)-3-(4-iodophenyl)propanoate (L-3 or D-3)

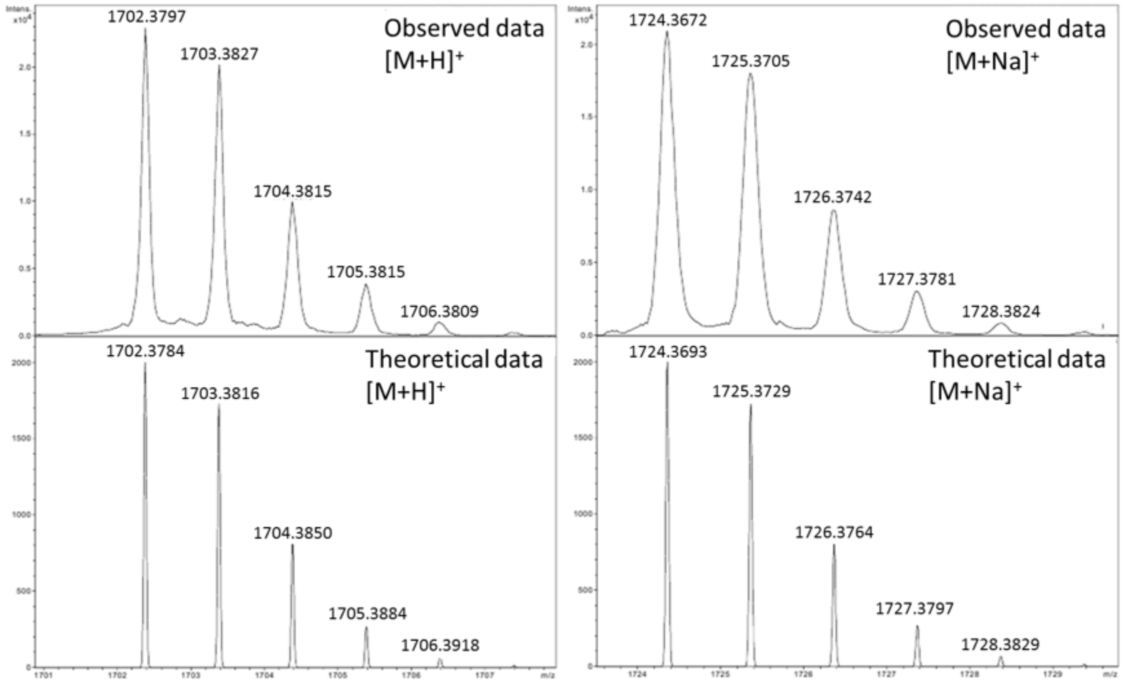

Figure S29. Observed (above) and calculated (below) isotopic pattern of ***L*-3** for $[M+H]^{+}$ and $[M+Na]^{+}$.

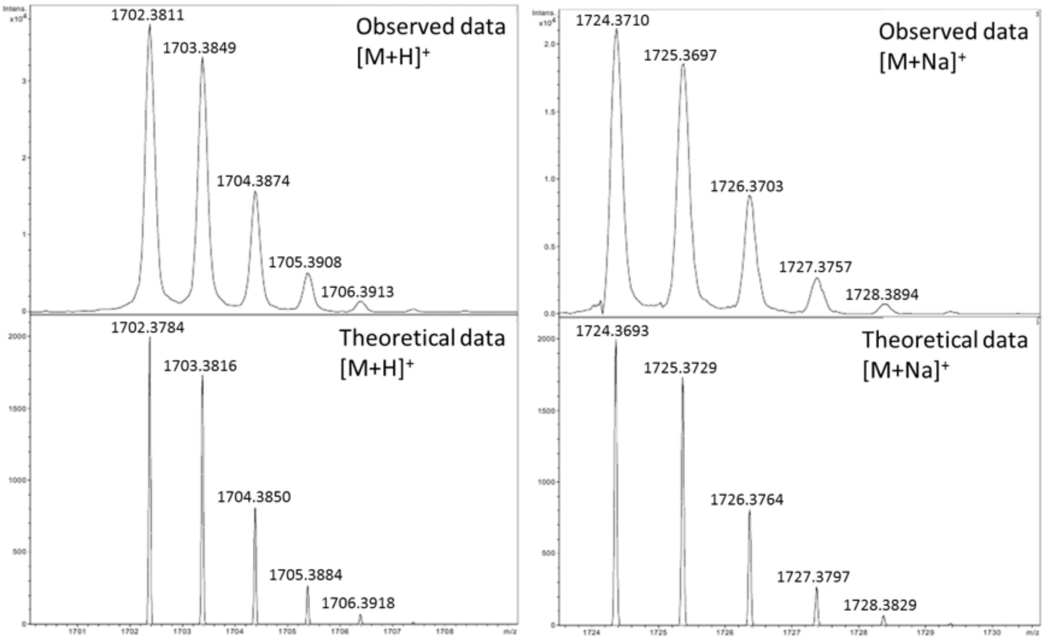

Figure S30. Observed (above) and calculated (below) isotopic pattern of ***D*-3** for $[M+H]^+$ and $[M+Na]^+$.

2,2'-((2,2'-(1,3,6,8-Tetraoxo-1,3,6,8-tetrahydrobenzo[lmn][3,8]phenanthroline-2,7-diyl)bis(3-(4-iodophenyl)propanoyl))bis(azanediyl))bis(N^1-(14-((1H-imidazol-5-yl)methyl)-17-carbamoyl-1-(1H-indol-3-yl)-8-isopropyl-5,19-dimethyl-3,6,9,12,15-pentaoxo-4,7,10,13,16-pentaazaicosan-2-yl)pentanediamide) (L-4 or D-4)

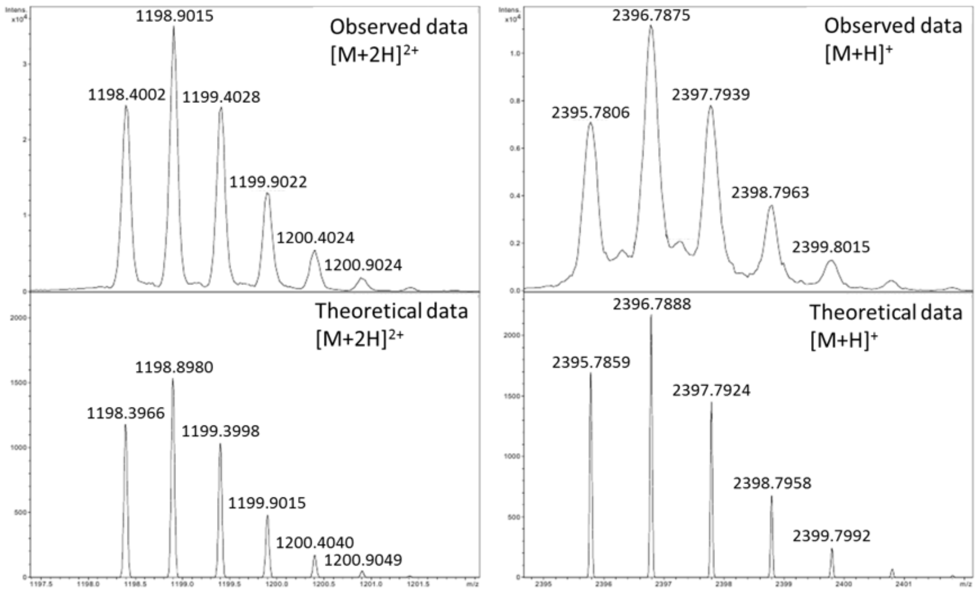

Figure S31. Observed (above) and calculated (below) isotopic patterns of ***L*-4** for $[M+2H]^{2+}$ and $[M+H]^+$, respectively.

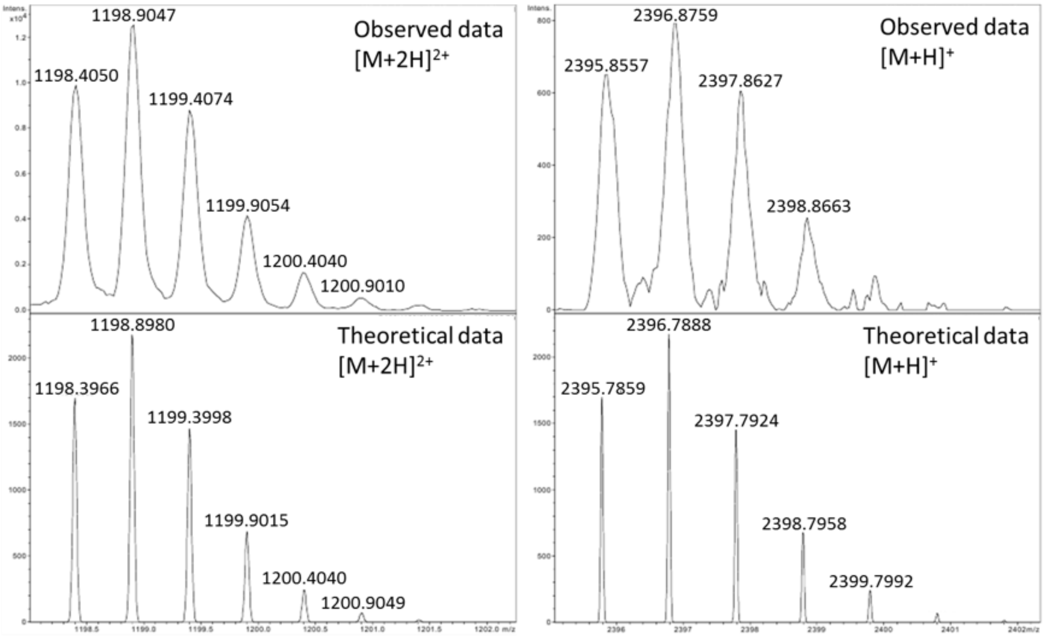

Figure S32. Observed (above) and calculated (below) isotopic patterns of ***D*-4** for $[M+2H]^{2+}$ and $[M+H]^{+}$, respectively**.**

Mass spectrum of (S)-N¹-((2S,5S,8S,14S,17S)-14-((1H-imidazol-5-yl)methyl)-17-carbamoyl-1-(1H-indol-3-yl)-8-isopropyl-5,19-dimethyl-3,6,9,12,15-pentaoxo-4,7,10,13,16-pentaazaicosan-2-yl)-2-(7-(2-(4-(5,5-difluoro-1,3,7,9-tetramethyl-5H-4λ⁴,5λ⁴-dipyrrolo[1,2-c:2',1'-f][1,3,2]diazaborinin-10-yl)benzamido)ethyl)-1,3,6,8-tetraoxo-3,6,7,8-tetrahydrobenzo[lmn][3,8]phenanthrolin-2(1H)-yl)pentanediamide (L-6)

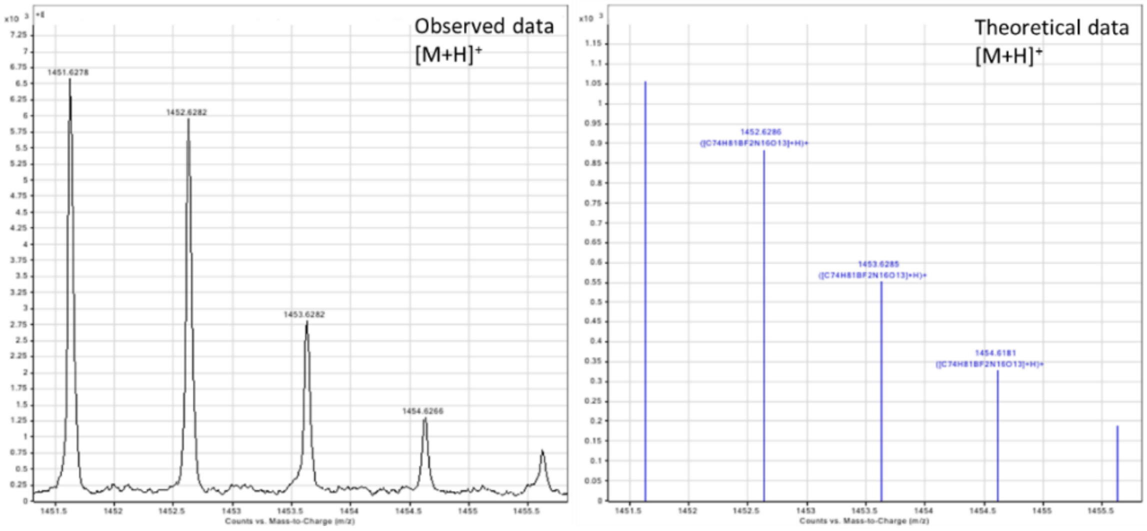

Figure S33. Observed (left) and calculated (right) isotopic patterns of ***L*-6** for $[M+H]^{+}$.

5. NMR spectroscopy and assignments

2,2'-(1,3,6,8-Tetraoxo-1,3,6,8-tetrahydrobenzo[lmn][3,8]phenanthroline-2,7-diyl)bis(3-(4-iodophenyl)propanoic acid) (L-1-OH or D-1-OH)

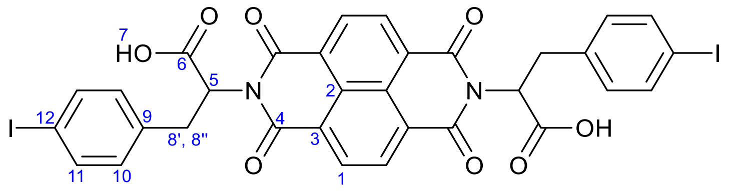

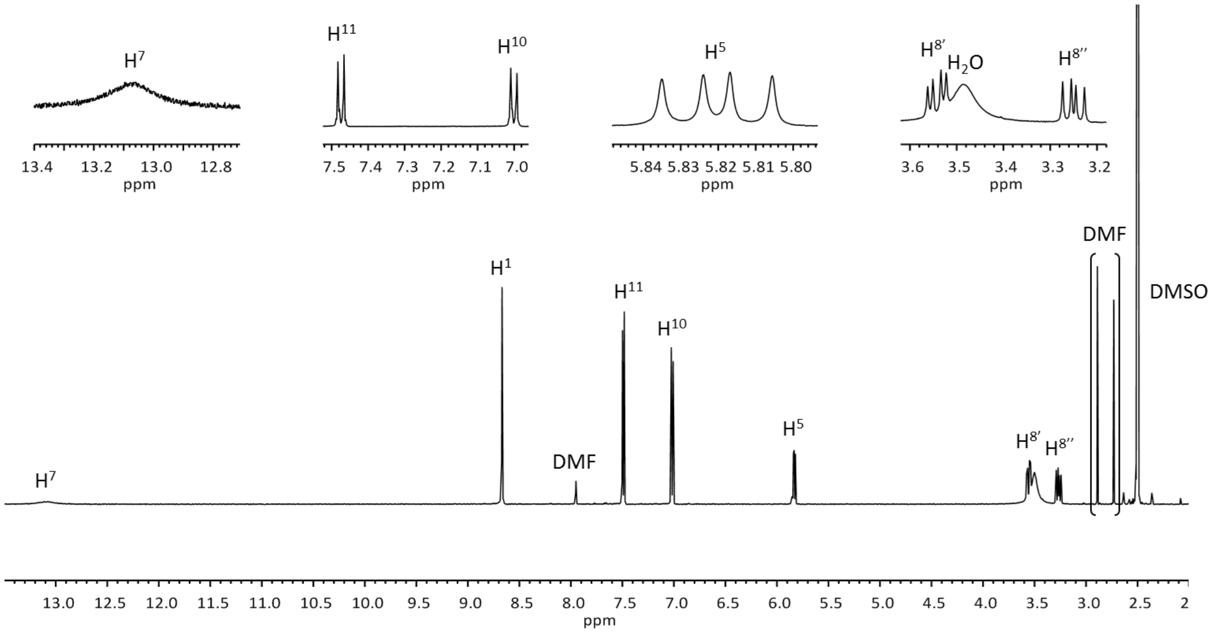

(a)

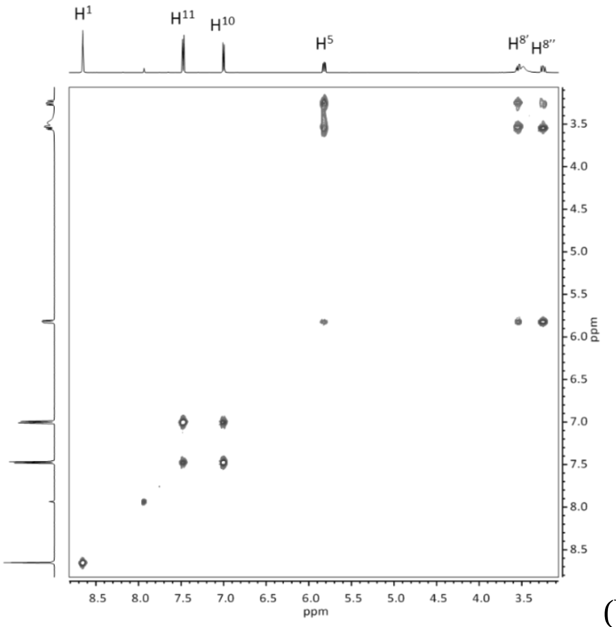

(b)

Figure S34. (a) ^{1}H NMR spectrum of L-1-OH and (b) ^{1}H-^{1}H COSY-NMR spectrum of L-1-OH (500 MHz, 298 K, DMSO-d$_6$). Identical spectra were obtained for D-1-OH.

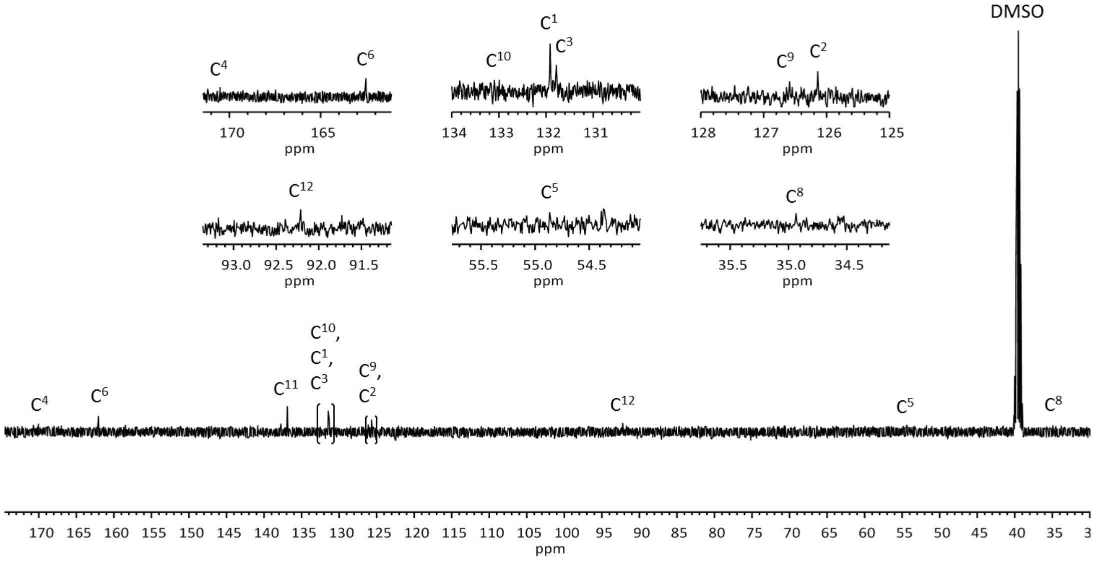

(a)

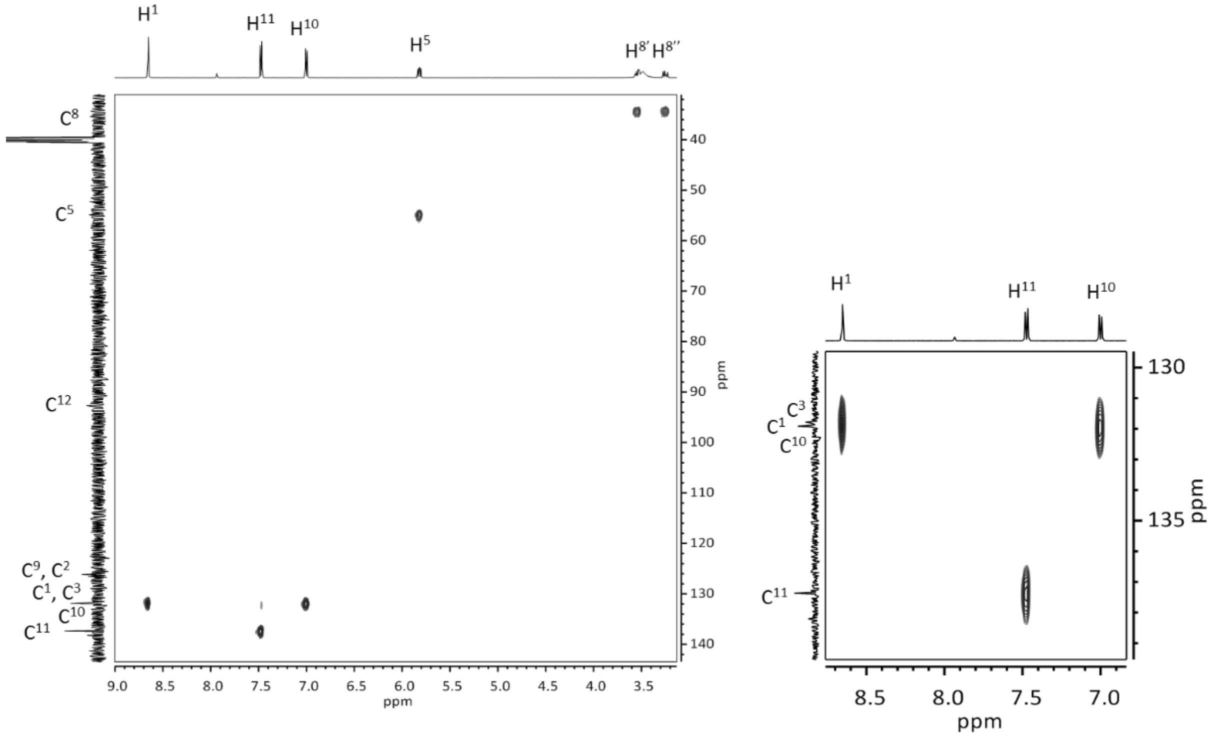

(b)

Figure S35. (a) $^{13}C\{^1H\}$ NMR spectrum of ***L*-1-OH**, 2,2'-(1,3,6,8-tetraoxo-1,3,6,8-tetrahydrobenzo[*lmn*][3,8]phenanthroline-2,7-diyl)*bis*(3-(4-iodophenyl)propanoic acid) (125 MHz, 298 K, DMSO-d_6). (b) ^1H-^{13}C HSQC-NMR spectrum of 2,2'-(1,3,6,8-tetraoxo-1,3,6,8-tetrahydrobenzo[*lmn*][3,8]phenanthroline-2,7-diyl)*bis*(3-(4-iodophenyl)propanoic acid) (500 MHz, 298 K, DMSO-d_6). Identical spectra were obtained for ***D*-1-OH**.

bis(2,5-Dioxopyrrolidin-1-yl) 2,2'-(1,3,6,8-tetraoxo-1,3,6,8-tetrahydrobenzo[lmn][3,8]phenanthroline-2,7-diyl)bis(3-(4-iodophenyl)propanoate (L-1 or D-1)

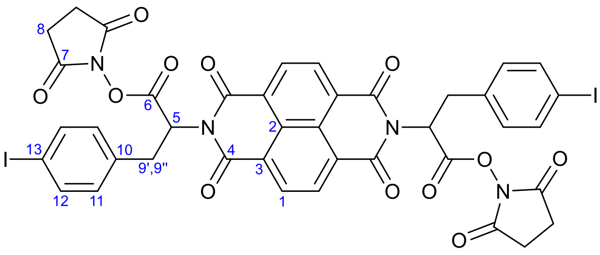

***L*-1** or ***D*-1**.

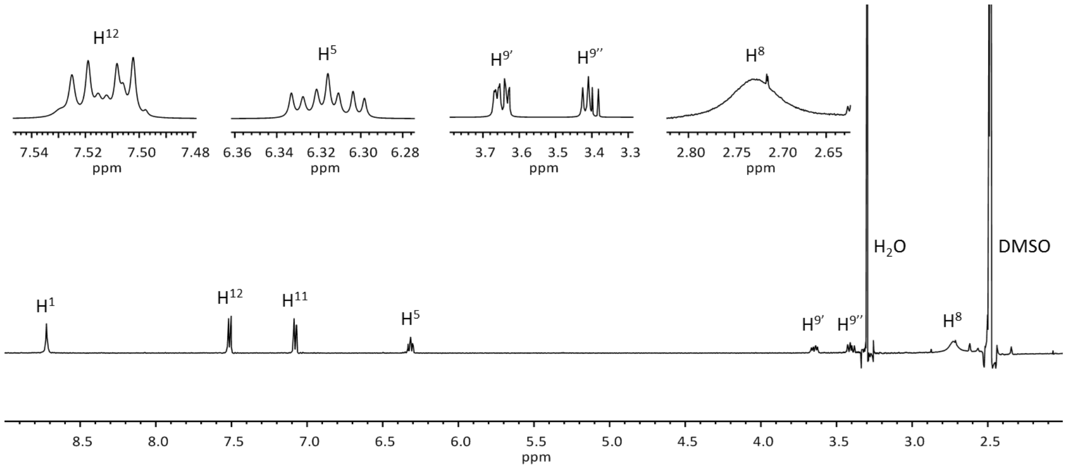

Figure S36. ^{1}H-NMR spectrum of **1** (500 MHz, 298 K, DMSO-d_6).

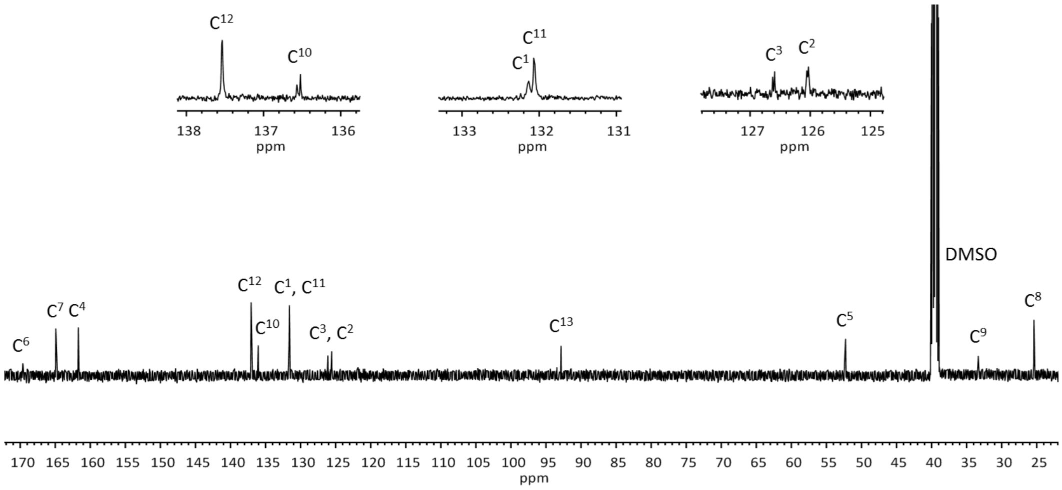

Figure S37. ^{13}C{^{1}H} NMR spectrum of **1** (125 MHz, 298 K, DMSO-d_6).

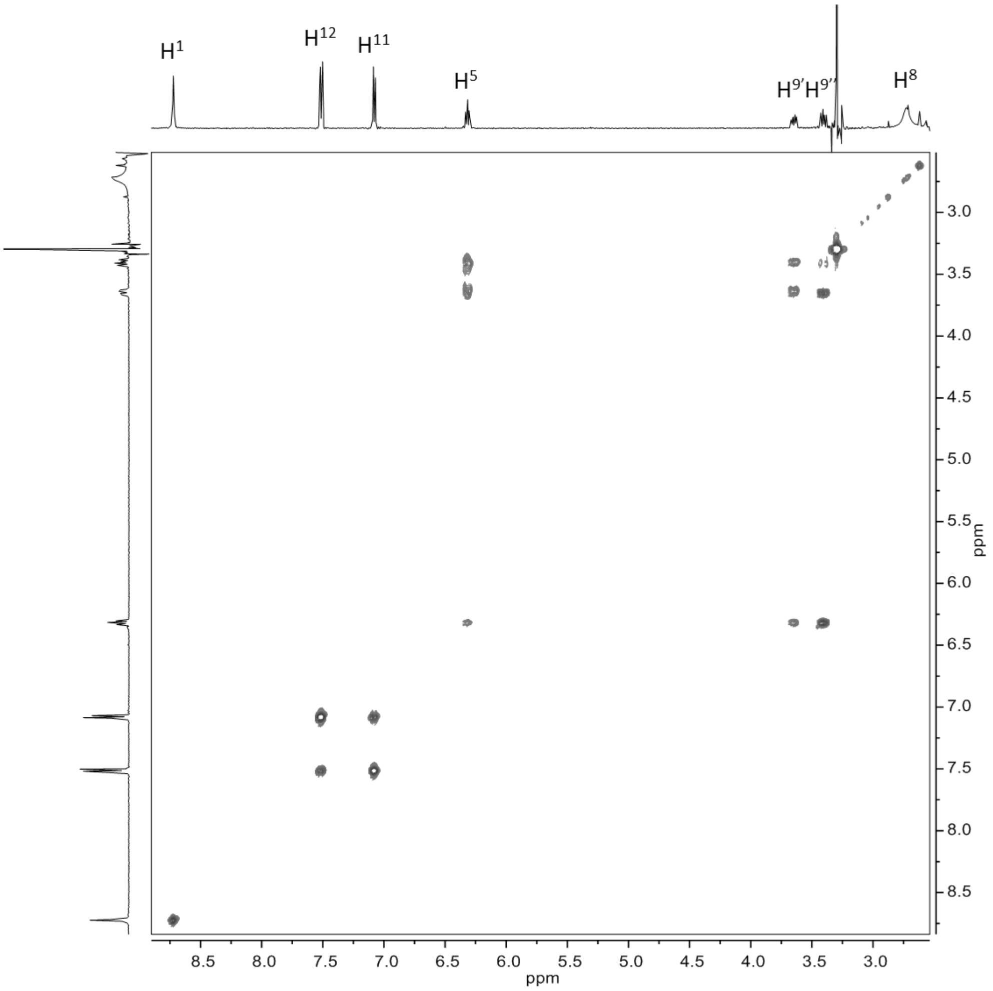

Figure S38. ^{1}H-^{1}H COSY-NMR spectrum of **1** (500 MHz, 298 K, DMSO-d_6).

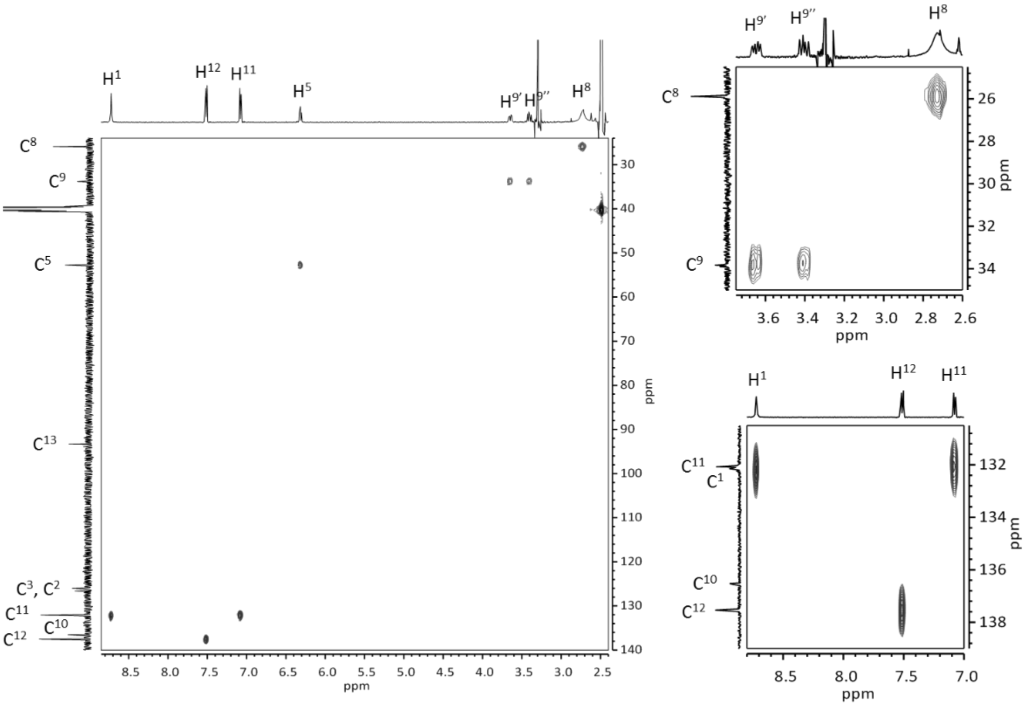

Figure S39. ^{1}H-^{13}C HSQC-NMR spectrum of **1** (500 MHz, 298 K, DMSO-d_6).

[7,13]-Bombesin peptide (L-2 or D-2)

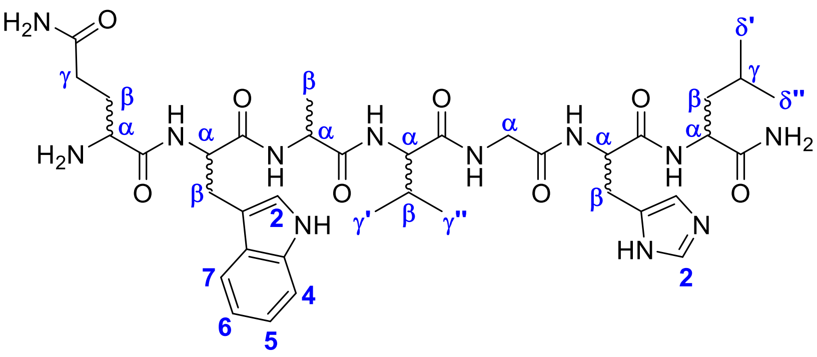

***L*-2** or ***D*-2**.

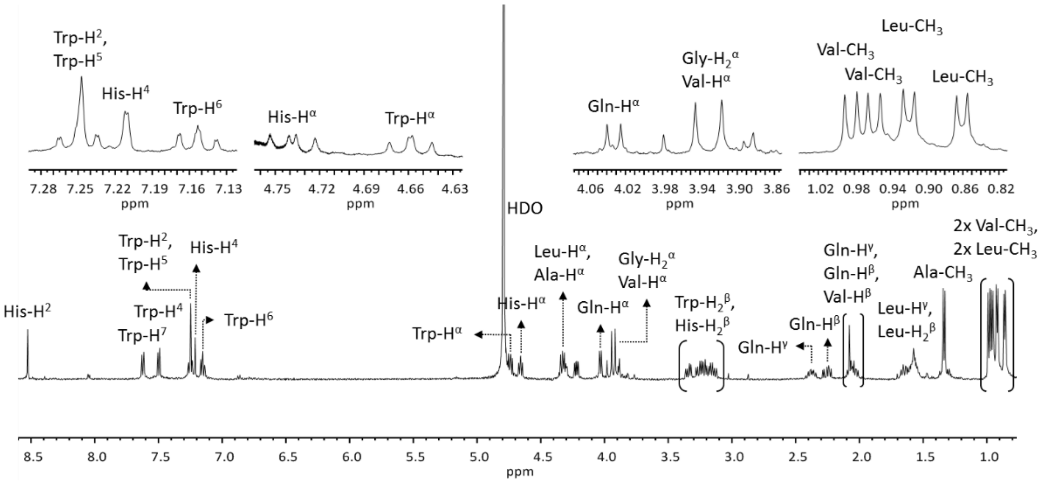

Figure S40. ^{1}H-NMR spectrum of ***L-2*** (500 MHz, 298 K, D_2O).

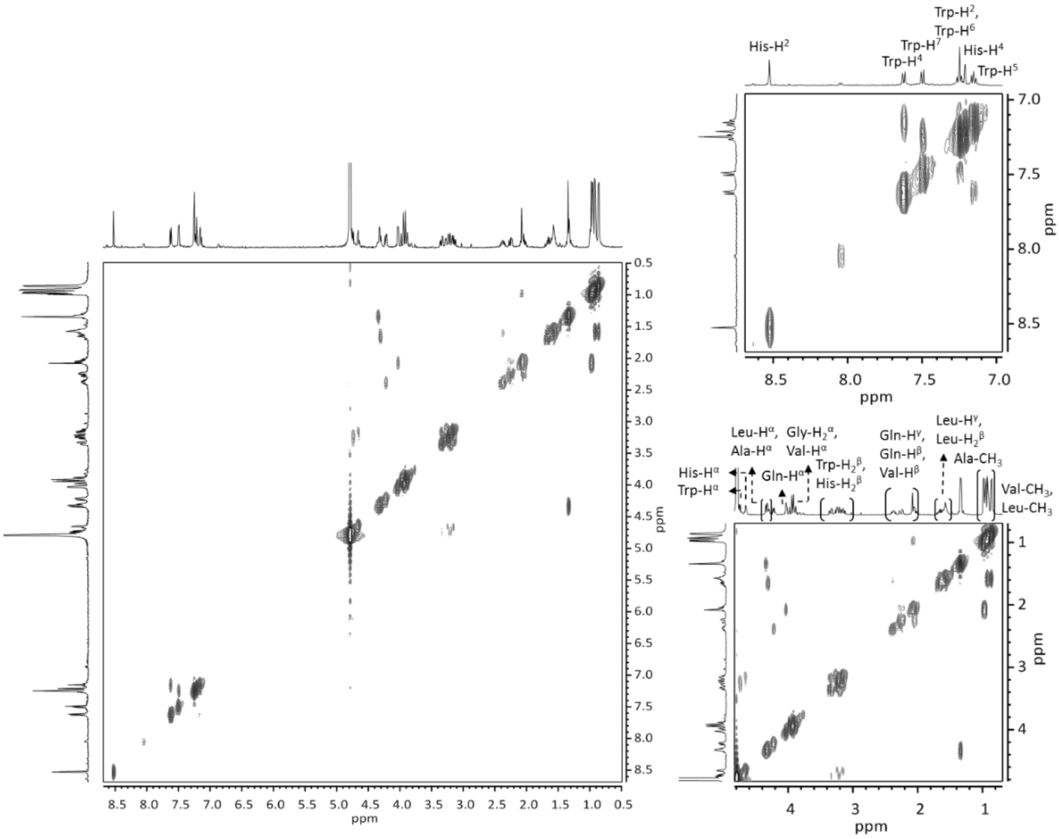

Figure S41. ^{1}H-^{1}H COSY-NMR spectrum of ***L-2*** (500 MHz, 298 K, DMSO-*d₆*).

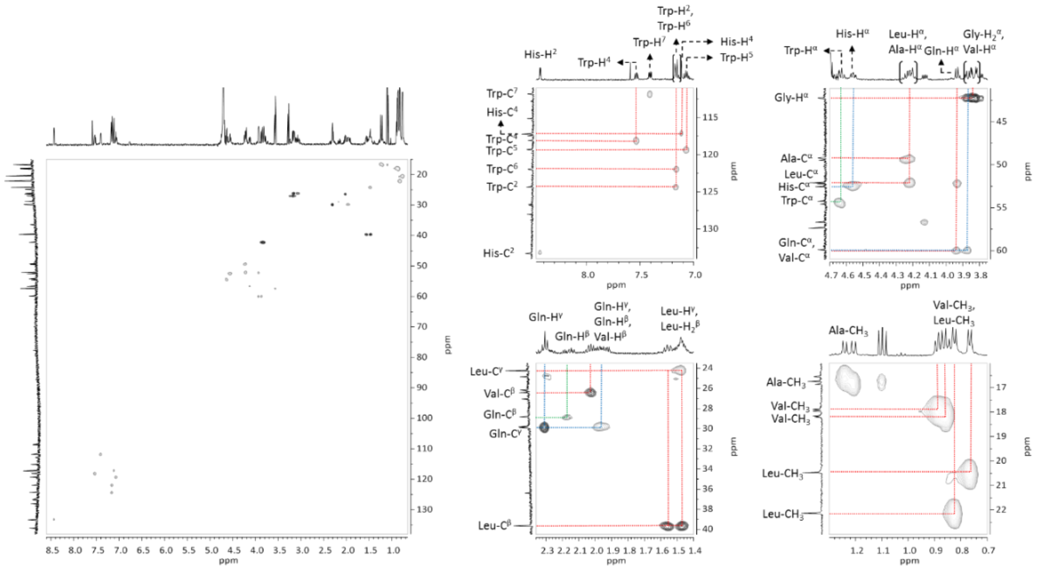

Figure S42. ^{1}H-^{13}C HSQC NMR spectrum of ***L-2*** (500 MHz, 298 K, DMSO-*d₆*).

N-(2-aminoethyl)-4-(5,5-difluoro-1,3,7,9-tetramethyl-5H-4λ4,5λ4-dipyrrolo[1,2-c:2',1'-f][1,3,2]diazaborinin-10-yl)benzamide (5)

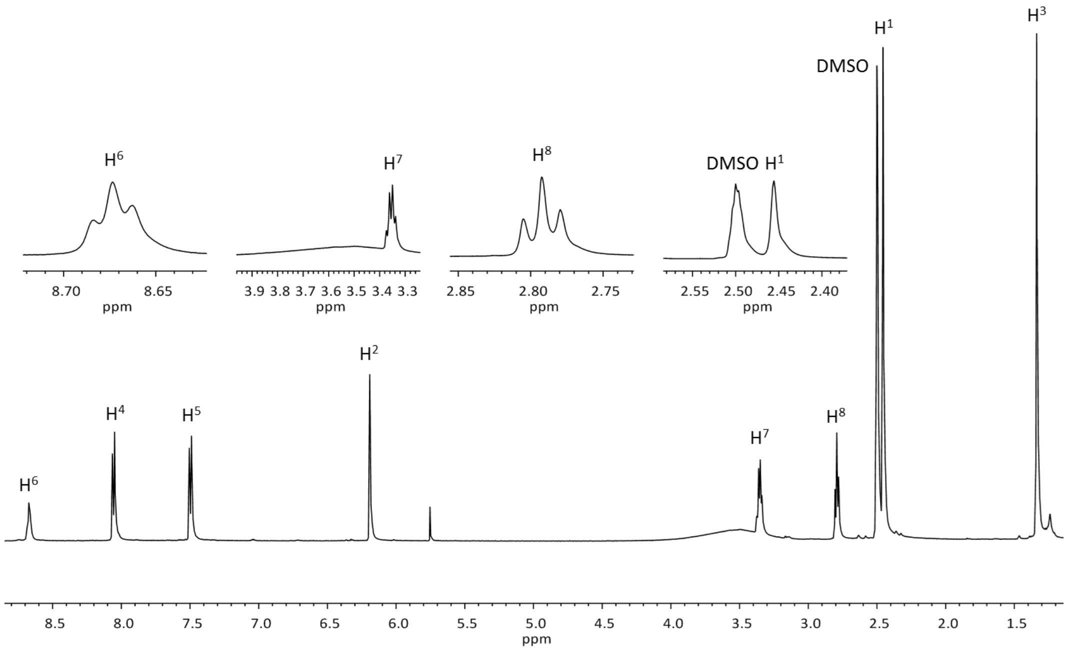

Figure S43. ^{1}H-NMR spectrum of **5** (500 MHz, 298 K, DMSO-d_6).

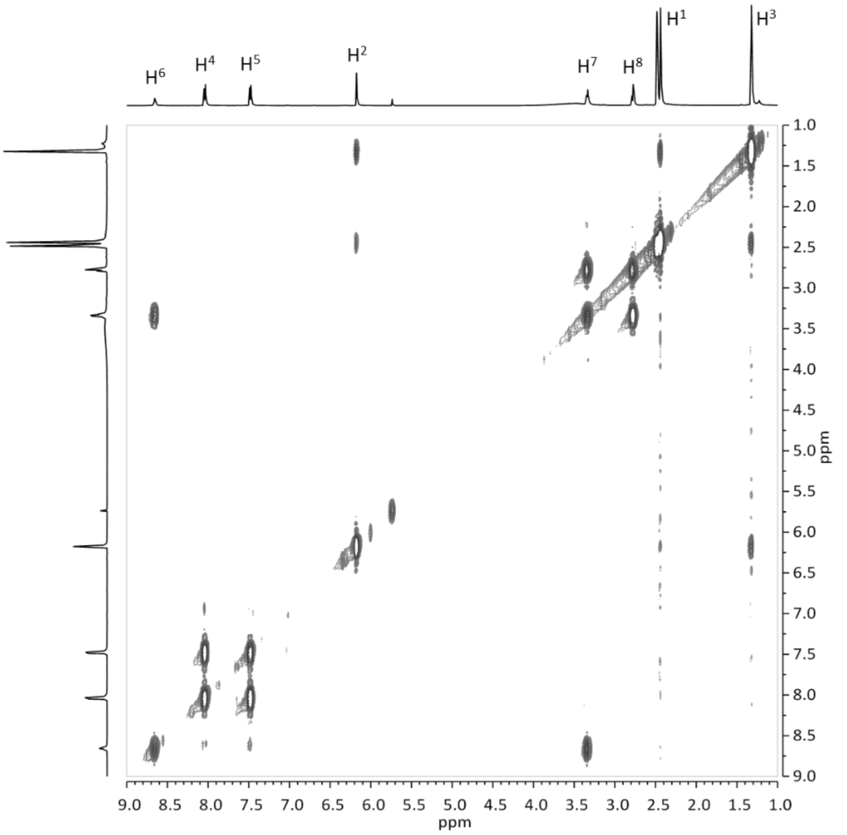

Figure S44. ^{1}H-^{1}H COSY-NMR spectrum of **5** (500 MHz, 298 K, DMSO-d_6).

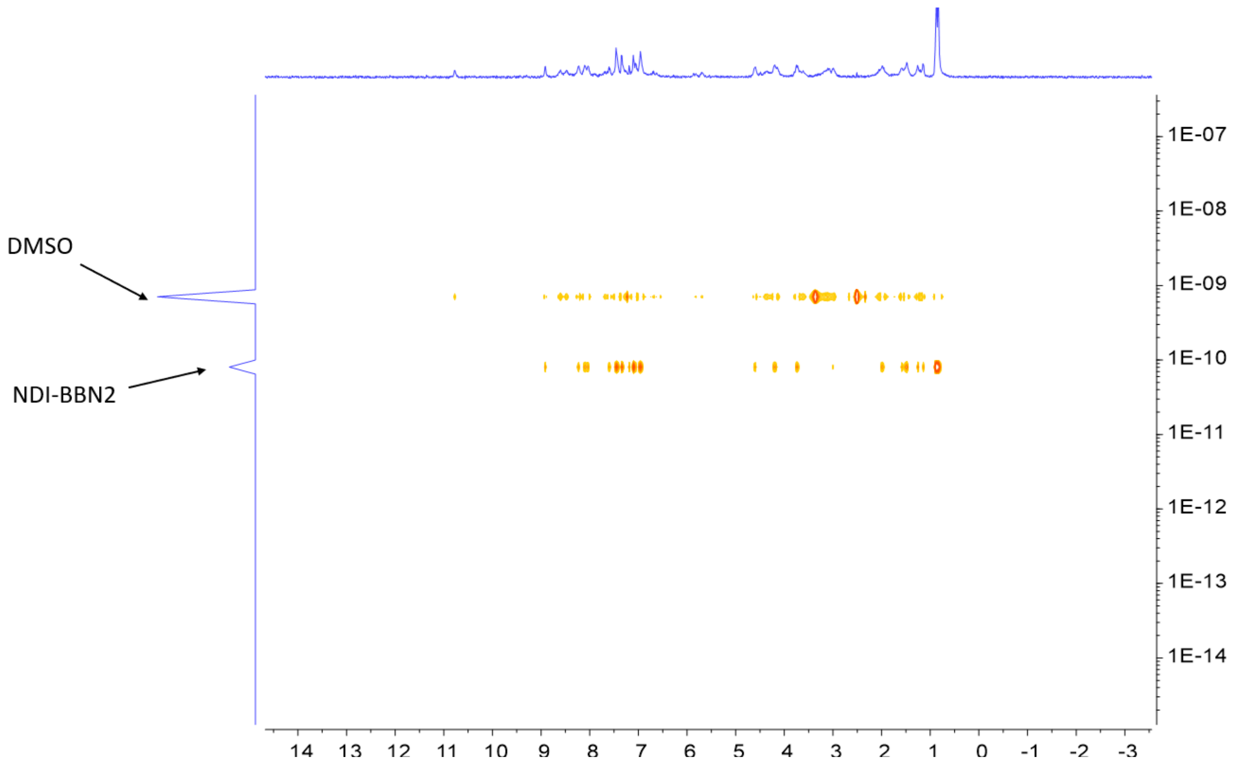

Figure S45. DOSY spectroscopy of ***L-4*** (400 MHz, 293 K, DMSO-d_6).

6. Circular dichroism spectra

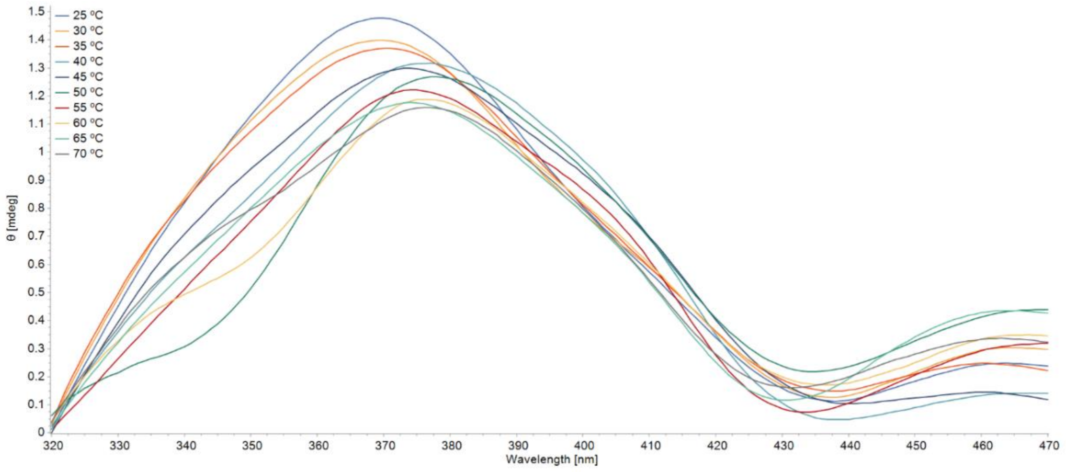

Figure S46. Circular dichroism profiles of ***L*-3** (200 µM in DMSO) recorded from 25 to 70 °C.

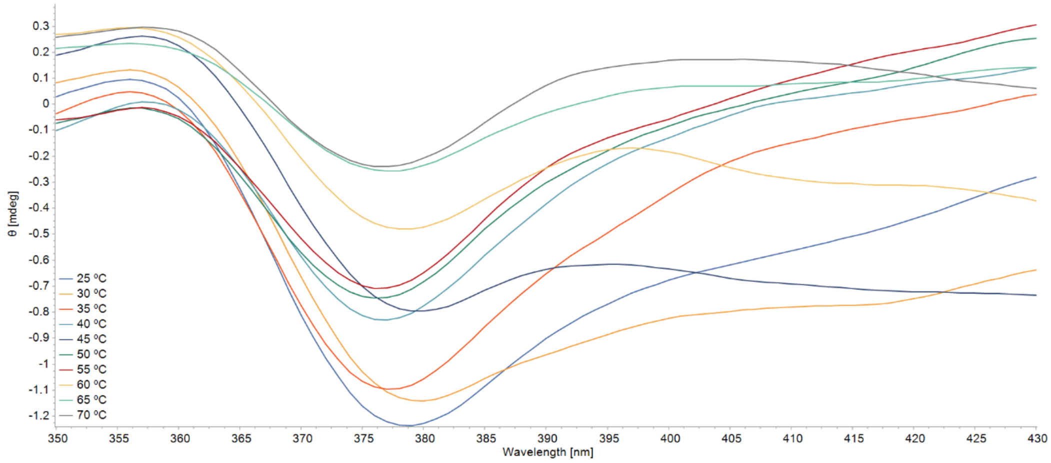

Figure S47. Circular dichroism profiles of ***D*-3** (200 µM in DMSO) recorded from 25 to 70 °C.

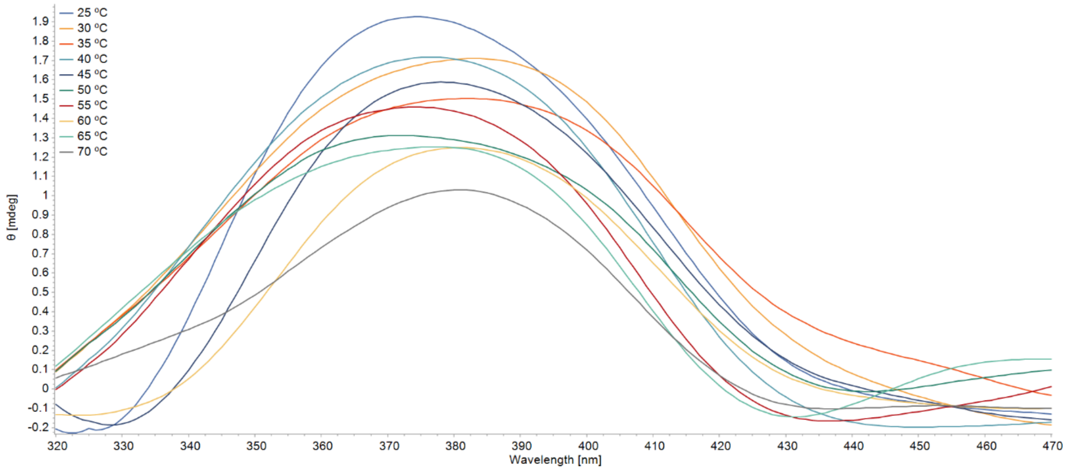

Figure S48. Circular dichroism profiles of ***L*-4** (200 µM in DMSO) recorded from 25 to 70 °C.

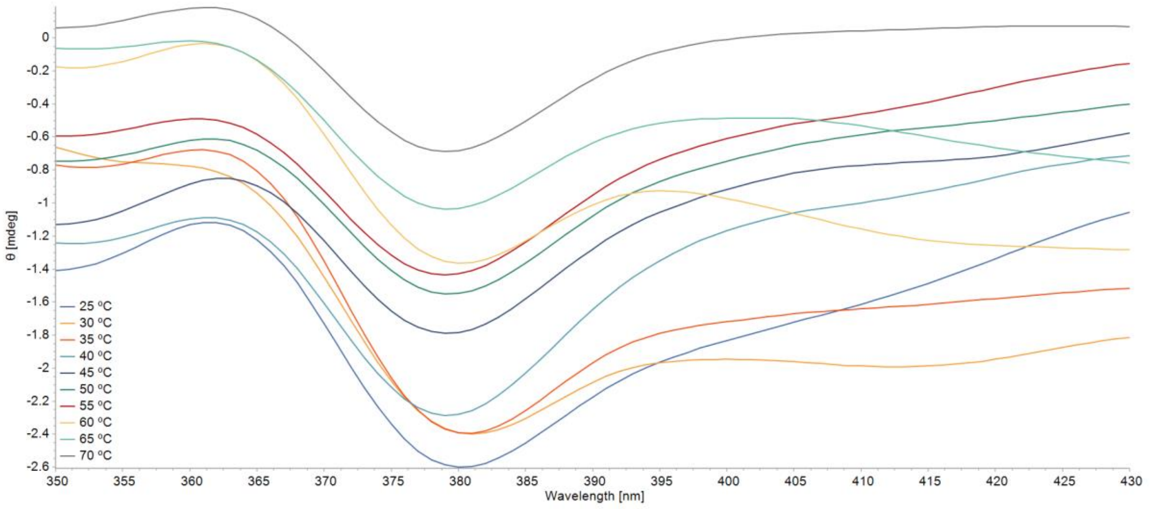

Figure S49. Circular dichroism profiles of ***D*-4** (200 µM in DMSO) recorded from 25 to 70 ºC.

7. HPLC traces of purified compounds used for cellular imaging

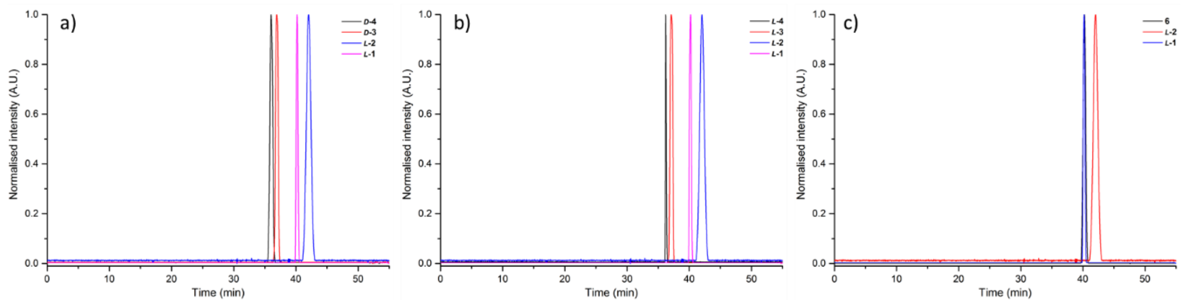

Figure S50. Analytical HPLC chromatograms of purified compounds. a) Comparative chromatograms of ***D*-3**, ***D*-4**, ***L*-1** and ***L*-2**. b) Comparative chromatograms of ***L*-3**, ***L*-4**, ***L*-1** and ***L*-2**; c) comparative chromatograms of ***L*-6**, ***L*-1** and ***L*-2**.

Table S1. Summary of retention times and wavelength of investigation using Method A.

Molecule	R_t (min)	λ_{abs} (nm)
***L*-1**	40.2	380
***L*-2**	42.0	280
***L*-3**	37.1	380
***D*-3**	36.9	380
***L*-4**	36.2	380
***D*-4**	36.0	380
***L*-6**	40.2	500

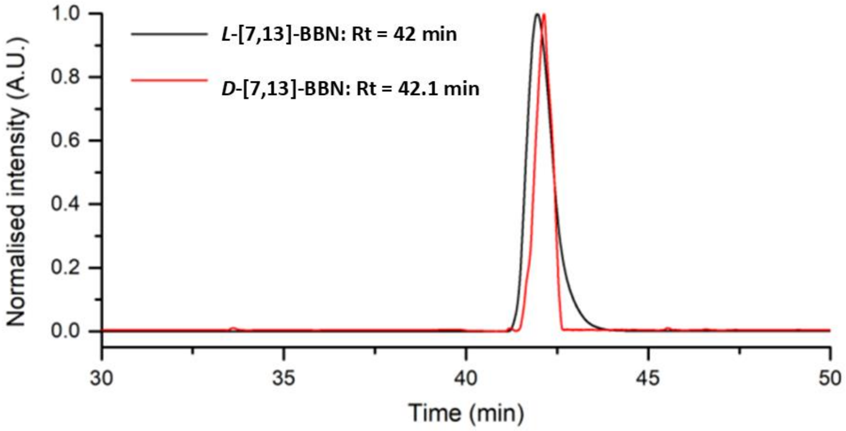

Figure S51. Comparative analytical HPLC of both enantiomers of the fragment [7-13] of the bombesin peptide, ***L*-2** (*L*-[7, 13]BBN), ***D*-2** (*D*-[7, 13]BBN). HPLC: Rt = 42.0 min (***L*-2**) and 42.1 min (***D*-2**).

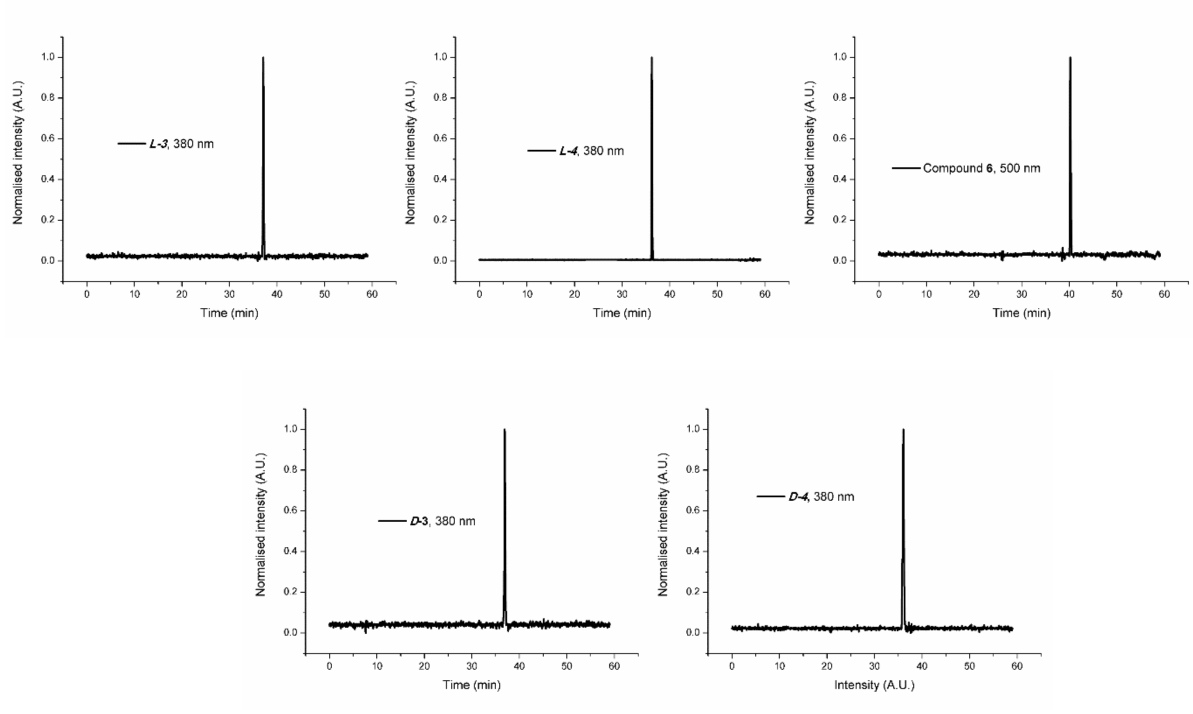

Figure S52. Comparative analytical HPLC of ***L*-3**, ***L*-4**, ***D-3***, ***D-4*** and ***L*-6** at a range of wavelengths

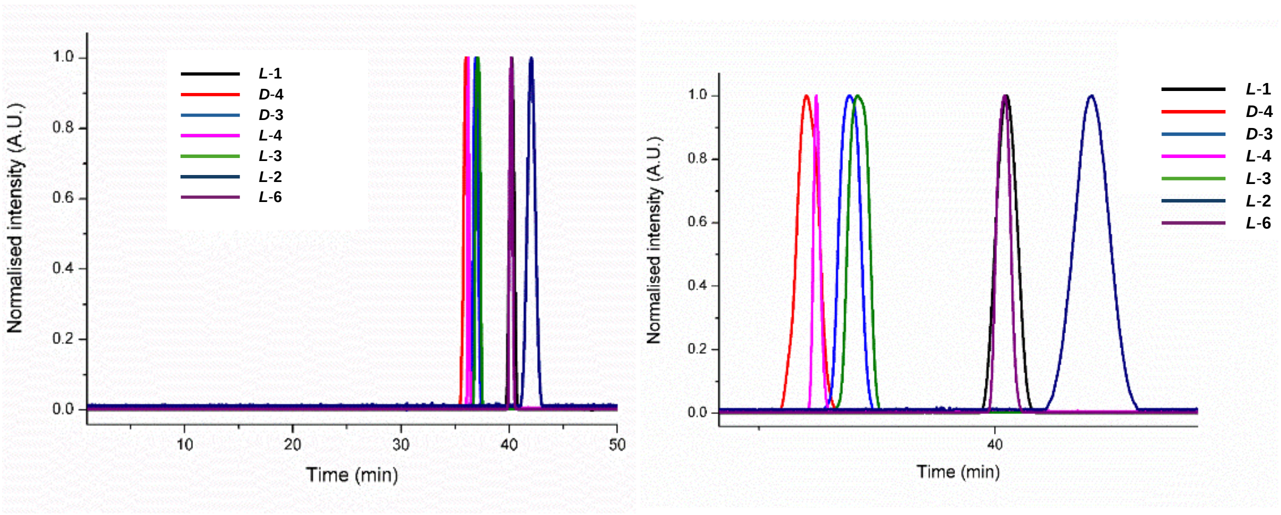

Figure S53. Comparative analytical HPLC chromatograms of ***L-2*** (navy blue), monitored wavelength 280 nm), ***L-1*** (black, monitored wavelength 380 nm), ***L-6*** (purple line, monitored wavelength 500 nm) ***L-3*** (green, monitored wavelength 380 nm), ***D-3*** (blue, monitored wavelength 380 nm), ***L-4*** (pink, monitored wavelength 380 nm), ***D-4*** (red, monitored wavelength 380 nm). *Note:* The mono-peptide probes (***L-3*** and ***D-3***) have retention times at ca. 37 min, distinct from those of the bis-substituted species ***L-4*** and ***D-4***. As expected each of the enantiomers (***L-3*** vs. ***D-3*** and ***L-4*** *vs.* ***D-4***) show similar retention times whereas the retention time of the free peptide is very different from the other probes. Overall, the peptide conjugates show retention times that are quite distinct from their NDI precursors ***L-1*** and ***D-1***, and peptide ***L-2*** (Method A).

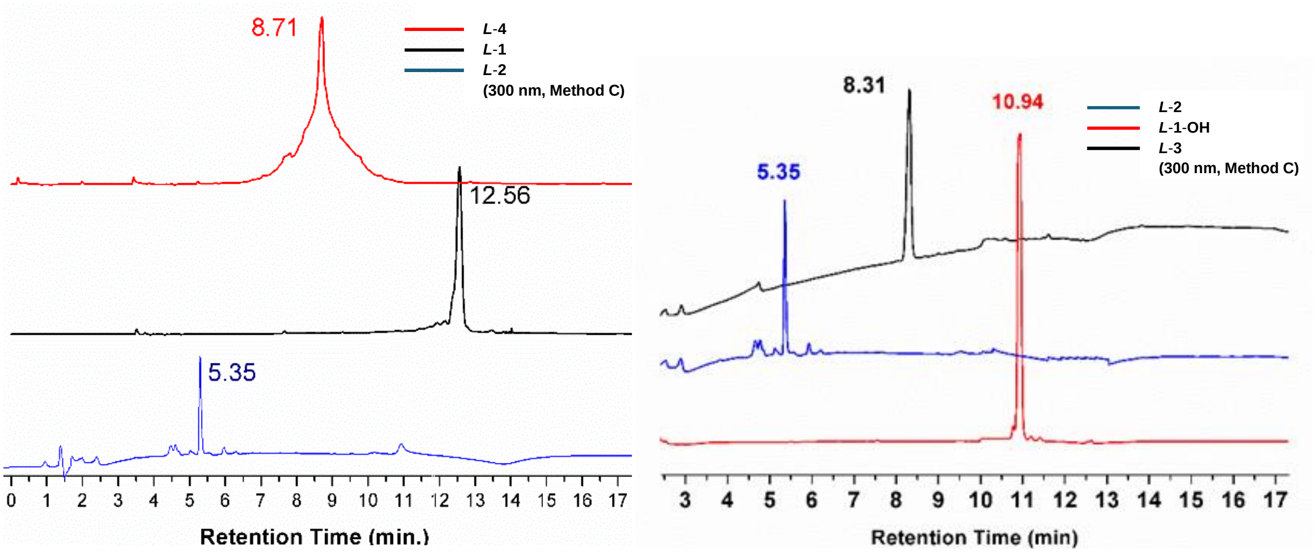

Figure S54. Comparison of HPLC traces of ***L*-3** and ***L*-4,** with relevant starting materials separated using semi-prep HPLC (Method B) and analysed after DMSO injection and using a modified HPLC method (Method C): Waters C-18 column (4.6 x 250 mm) with UV/visible detection (254 nm). The gradient elution was 0.8 mL/min with 0.1% TFA milli-Q water as solvent A and 0.1% TFA/acetonitrile as solvent B. A reverse gradient was applied starting with A at 95%, going up to 5% A at 7.5 minutes, isocratic until 15 minutes and gradient until 95% A, then hold to 18 min.

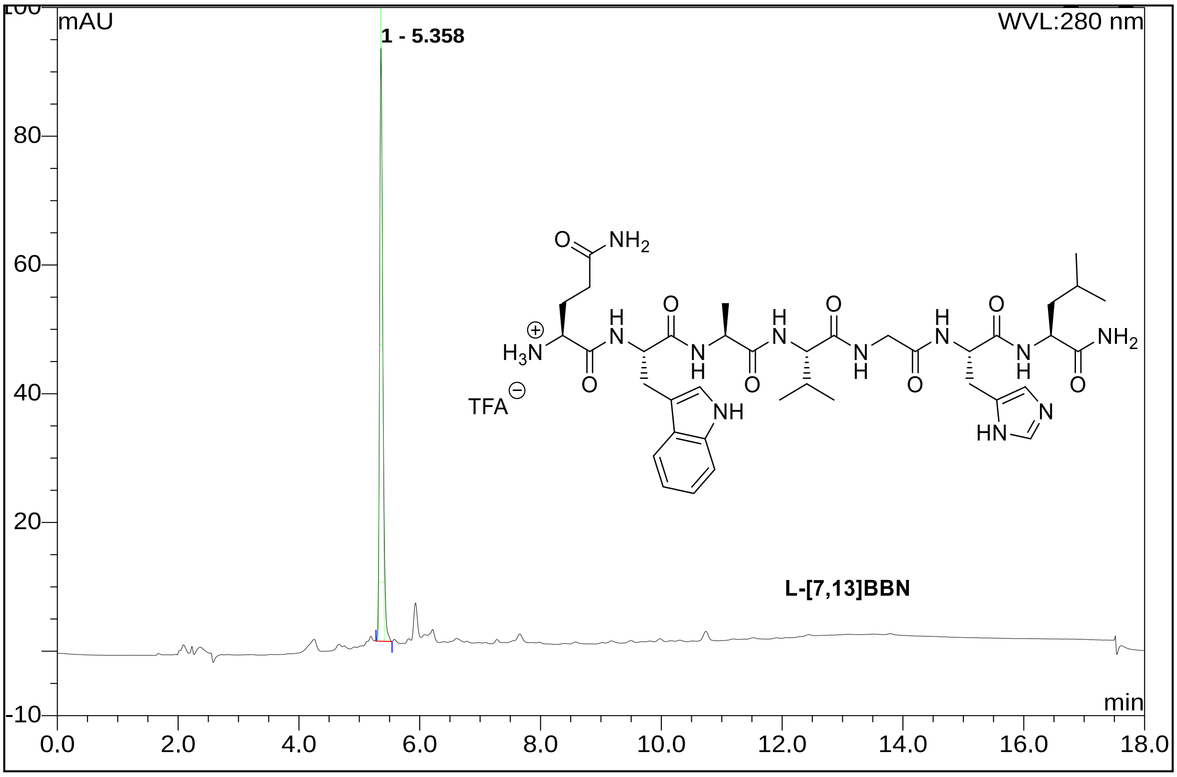

Figure S55. Normalised C18 HPLC chromatogram (semi-preparative) of the purified L-[7,13]-bombesin (280 nm) ***L*-2** injected from DMSO (Method C).

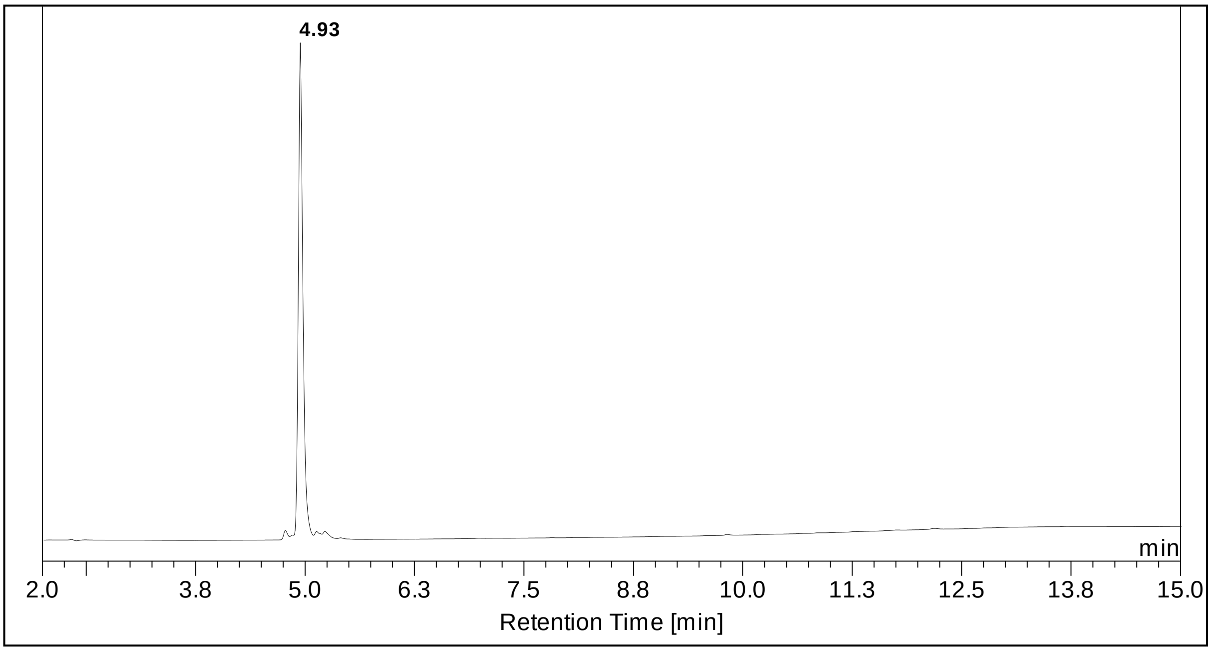

Figure S56. Normalised C18 HPLC chromatogram of the purified D-bombesin ***D*-2** (254 nm) injected from DMSO (Method B).

8. UV-visible and fluorescence spectroscopies

L-1

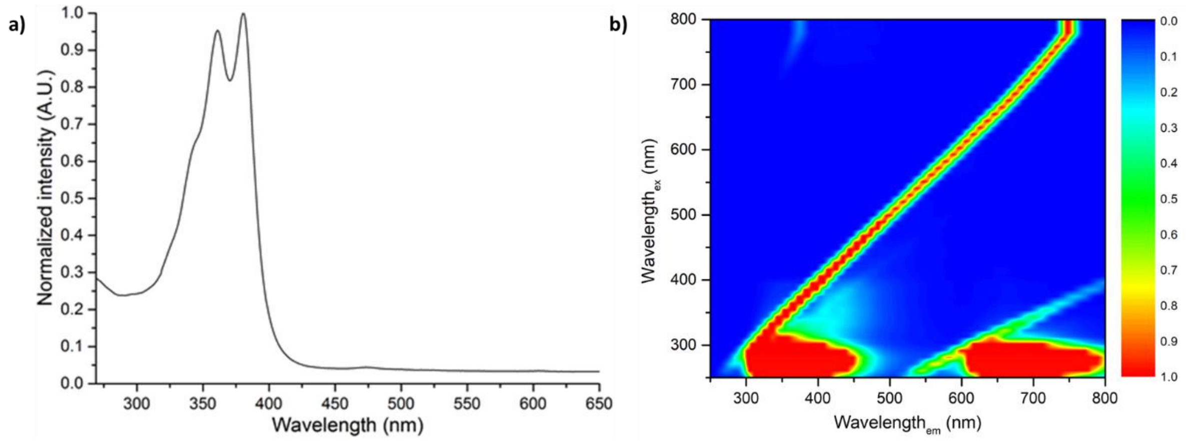

Figure S57. a) UV-visible spectrum and b) Excitation-Emission map of ***L*-1** (2.5 µM in DMSO).

L-2

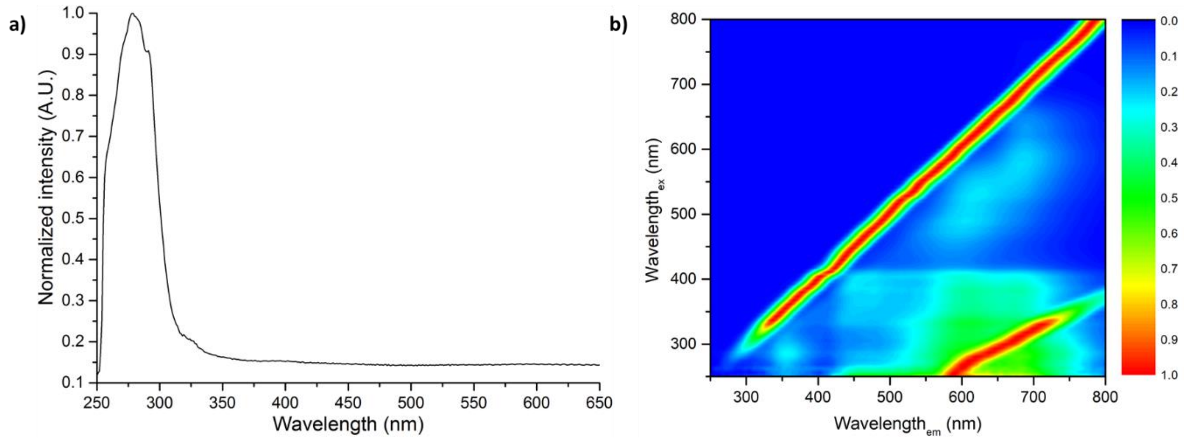

Figure S58. a) UV-visible spectrum and b) Excitation-Emission map of ***L-2*** (50 µM in DMSO).

L-3

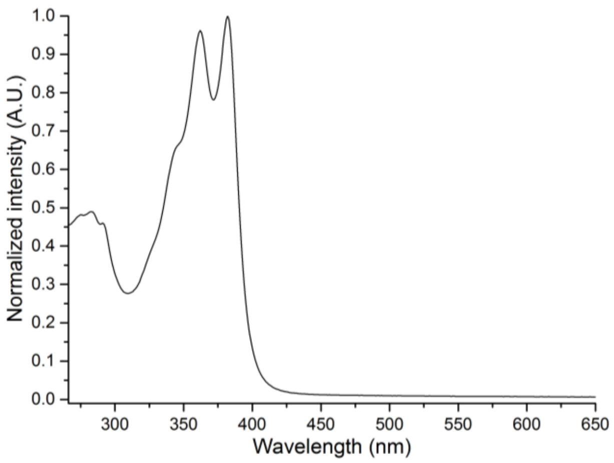

Figure S59. UV-visible spectrum of ***L-3*** (200 μM in DMSO).

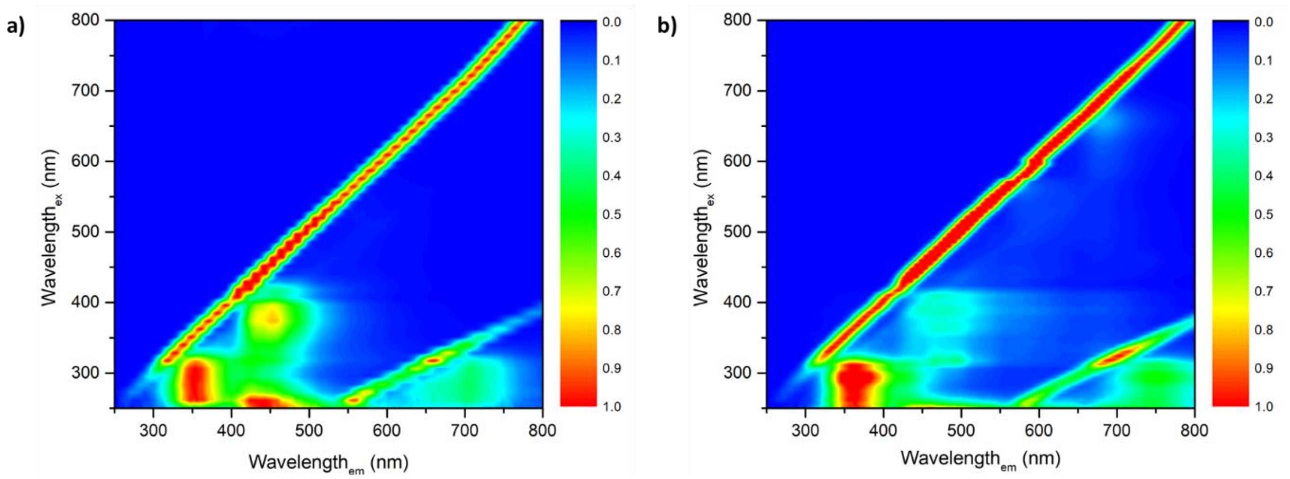

Figure S60. Comparison of excitation-Emission map of a) ***L*-3** and b) ***D*-3** (200 μM in DMSO).

L-4

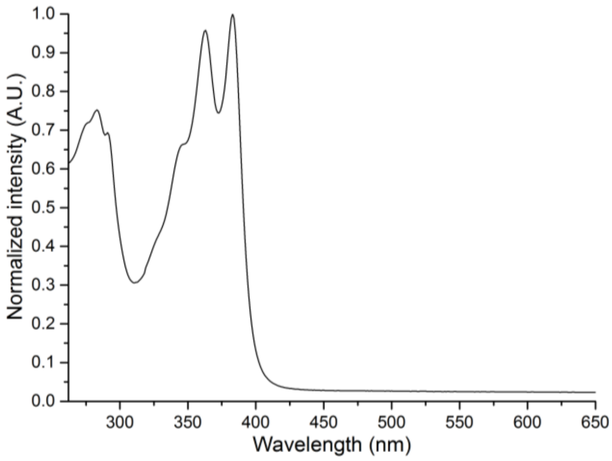

Figure S61. UV-visible spectroscopy of ***L-4*** (200 µM in DMSO).

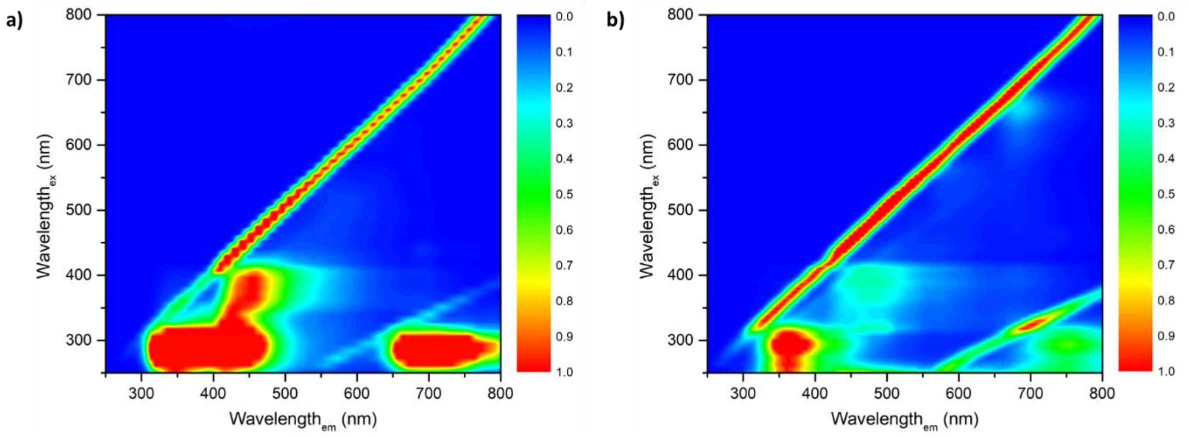

Figure S62. Excitation-Emission maps of a) ***L-4*** and b) ***D-4*** (200 µM in DMSO).

L-6

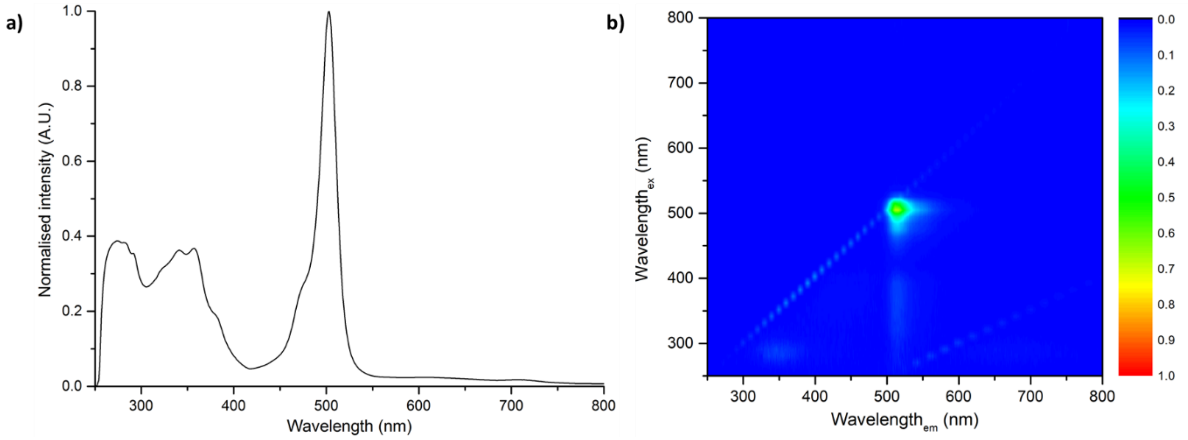

Figure S63. a) UV-visible spectrum and b) Excitation-Emission map of ***L*-6** (50 µM in DMSO).

Table S2. Summary of molar extinction coefficients and quantum yields of the synthesised molecules. The Φ were calculated using the following standards: a) Rhodamine B 2.6 nM in MeOH (Φ=0.70±0.02; λ_{ex}=348 nm; η=1.33); b) *L*-tryptophan 0.1 µM in H_2O (pH=6) (Φ=0.12±0.01; λ_{ex}=280 nm; η=1.33); c) fluorescein 2.3 nM in 0.1 M $NaOH_{(aq)}$ (Φ=0.95±0.03; λ_{ex}=496 nm; η=1.33)..

**Molecule** (conc.)	ε_{max} **(·10^3 L mol^{-1} cm^{-1})** (λ_{max})	**Φ** (λ^{em}_{max})
***L*-2** (50 µM DMSO)	4.4 (278 nm)	0.037 (340 nm)[b]
***D*-2** (50 µM DMSO)	3.1 (278 nm)	0.037 (341 nm)[b]
***L*-1** (2.5 µM DMSO)	472.0 (361 nm)	0.092 (427 nm)[a]
***D*-1** (2.5 µM DMSO)	493.3 (361 nm)	0.094 (426 nm)[a]
***L*-3** (200 µM DMSO)	14.9 (362 nm)	0.015 (429 nm)[a]
***D*-3** (200 µM DMSO)	16.6 (362 nm)	0.023 (428 nm)[a]
***L*-4** (200 µM DMSO)	16.7 (363 nm)	0.050 (433 nm)[a]
***D*-4** (200 µM DMSO)	14.7 (363 nm)	0.058 (434 nm)[a]
**5** (100 nM DMSO)	849.9 (503 nm)	0.046 (510 nm)[c]
***L*-6** (50 µM DMSO)	26.2 (503 nm)	0.003 (514 nm)[c]

9. FRET Analysis

Compound ***L*-6** showed an intense relative emission range at around 500 and 560 nm when it is excited between 470 and 520 nm. This range is particularly interesting for the *in vitro* studies through confocal microscopy in which the auto-fluorescence emission of cells is avoided. Furthermore, the fluorescence emission is dominated by the BODIPY moiety which is expected as it is widely used for imaging purposes. It appears that, in this spectrum, the emissions range assignable to the bombesin [7-13] and the NDI-core were only slightly perceptible. The molar extinction coefficients (ε) and the quantum yields (Φ) of the imaging probes and their precursors are summarised above.

Both enantiomers of BBN and INDI present molar extinction coefficients and quantum yields comparable which is expected from optical features of enantiomers. In addition, compounds INDI have molar extinction coefficients almost 100-fold than peptides BBN. As expected in ***L*-3, *D*-3, *L*-4** and ***D*-4**, the stereochemistry did not affect the lifetime decays of the molecules in different solvent conditions. Compound 6 (L-isomer) presented similar behaviour passing from DMSO to DMSO:H_2O (1:1). Particularly, it showed similar slow components approximatively 0.1 ns both in DMSO (a_5) and DMSO:H_2O (b_5) solutions. Surprisingly, the τ1 values obtained for ***L*-6** are ten-fold lower than the ones for the BODIPY derivative **5**. This could be due to a different aggregation of the species in solution.
From the values of τ_1 and τ_2 can be calculated the FRET efficiency (E_{FRET}), *i.e.* the probability of an energy transfer arising from the excitation event of each molecule[4]. It can be expressed as follows:

$$E_{FRET} = 1 - \frac{\tau_{FRET}}{\tau_0}$$

where τ_{FRET} correspond to the fast component (τ_1) and τ_0 is associated with the slow component (τ_2). The following tables resume the E_{FRET} of the building blocks used (Table S4) and the proposed fluorescent probes **3**, **4 (*L*-** and ***D*-** forms) and ***L*-6** (Table S5).

Table S3. Calculated FRET efficiency (E_{FRET}) values for the precursors investigated

	DMSO				DMSO:H_2O (1:1)			
**Molecule**	**Conc. (mM)**	**τ_{FRET} (ns)**	**τ_0 (ns)**	**E_{FRET} (%)**	**Conc. (mM)**	**τ_{FRET} (ns)**	**τ_0 (ns)**	**E_{FRET} (%)**
***L*-2**	0.01	0.91	11.06	91.77	0.025	0.46	13.85	96.68
	0.1	1.08	9.75	88.92	0.25	0.41	8.68	95.28
	1	0.85	7.50	88.67	2.5	0.40	9.10	95.60
***D*-2**	0.01	1.00	6.61	84.87	0.025	0.94	9.31	89.90
	0.1	0.99	7.58	86.94	0.25	0.85	8.40	89.88
	1	0.98	6.69	85.35	2.5	0.65	5.68	88.56
***L*-1**	0.01	0.98	9.95	90.15	0.025	0.47	7.82	93.99
	0.1	0.88	8.46	89.60	0.25	0.44	2.85	84.56
	1	0.79	9.81	91.95	2.5	0.40	1.77	77.40
***D*-1**	0.01	0.91	4.14	78.02	0.025	0.94	6.98	86.53
	0.1	0.84	4.61	81.78	0.25	0.66	3.17	79.18
	1	0.76	3.13	75.72	2.5	0.40	1.60	75.00
**5**	0.01	2.73	9.62	71.62	0.025	2.96	2.96	0.00
	0.1	2.99	6.16	51.46	0.25	2.70	6.40	57.81
	1	3.35	6.50	48.46	2.5	2.53	9.75	74.05

Table S4. Estimated FRET (E_{FRET}) values for compounds ***L*-3**, ***L*-4**, ***D*-3**, ***D*-4** and ***L*-6**

	DMSO				DMSO:H_2O (1:1)			
**Molecule**	**Conc. (mM)**	**τ_{FRET} (ns)**	**τ_0 (ns)**	**E_{FRET} (%)**	**Conc. (mM)**	**τ_{FRET} (ns)**	**τ_0 (ns)**	**E_{FRET} (%)**
***L*-3**	0.01	0.84	7.35	88.57	0.025	0.37	10.01	96.30
	0.1	0.66	5.53	88.07	0.25	0.38	9.87	96.15
	1	0.38	2.54	85.04	2.5	0.31	3.45	91.01
***D*-3**	0.01	0.88	6.57	86.61	0.025	0.30	3.27	90.83
	0.1	0.60	6.66	90.99	0.25	0.24	3.30	92.73
	1	0.52	4.30	87.91	2.5	0.19	3.73	94.91
***L*-4**	0.01	0.51	4.76	89.29	0.025	0.48	9.57	94.98
	0.1	0.49	5.45	91.01	0.25	0.40	3.12	87.18
	1	0.48	4.29	88.81	2.5	0.26	4.97	94.77
***D*-4**	0.01	0.61	7.55	91.92	0.025	0.64	13.15	95.13
	0.1	0.64	5.98	89.30	0.25	0.46	6.30	92.70
	1	0.58	4.77	87.84	2.5	0.39	3.28	88.11
***L*-6**	0.01	0.14	3.34	95.81	2.5	0.15	2.25	93.33
	0.1	0.14	2.64	94.70				
	1	0.14	1.67	91.62				

Note. The values of the calculated FRET efficiency of the proposed probes are overall close to 90%. In particular, in DMSO:H_2O mixture, these values are slightly higher than in pure DMSO. This result suggested that such solvent mixture increases the interactions among molecules than in pure DMSO and therefore increases their FRET events. Comparing the data obtained from the enantiomers of **3** and **4** against corresponding free INDI and BBN precursors, it can be noticed that the FRET efficiency values of the NDI-based probes are slightly higher than their corresponding precursors. The presence of both building blocks in the same structure may help the interactions of the molecules thanks to a combination of weak interactions as H-bonds and π-π stackings. Compound ***L*-6** showed higher E_{FRET} values in both solvent systems than its precursor **5**. In this case, the presence of the peptide fragent ***L***-[7,13]BBN and the NDI-core in the structure may improve the interactions among molecules as a result of more FRET events in solution. Comparing the enantiomers of **3** and **4**, it can be noticed that their ε values are similar, as expected. On the other hand, the quantum yields of the *bis*-peptide derivatives are higher than their *mono*-versions. The presence of two moieties of the peptide on the same structure may play an important role in enhancing the quantum yield of the entire molecule.
Compound ***L*-6** showed values of ε and Φ very different from the ones of its precursor **5**. In particular, the molar extinction coefficient of ***L*-6** is almost 35-fold less than **2** and the quantum yield is 5 times lower.

10.SEM experiments

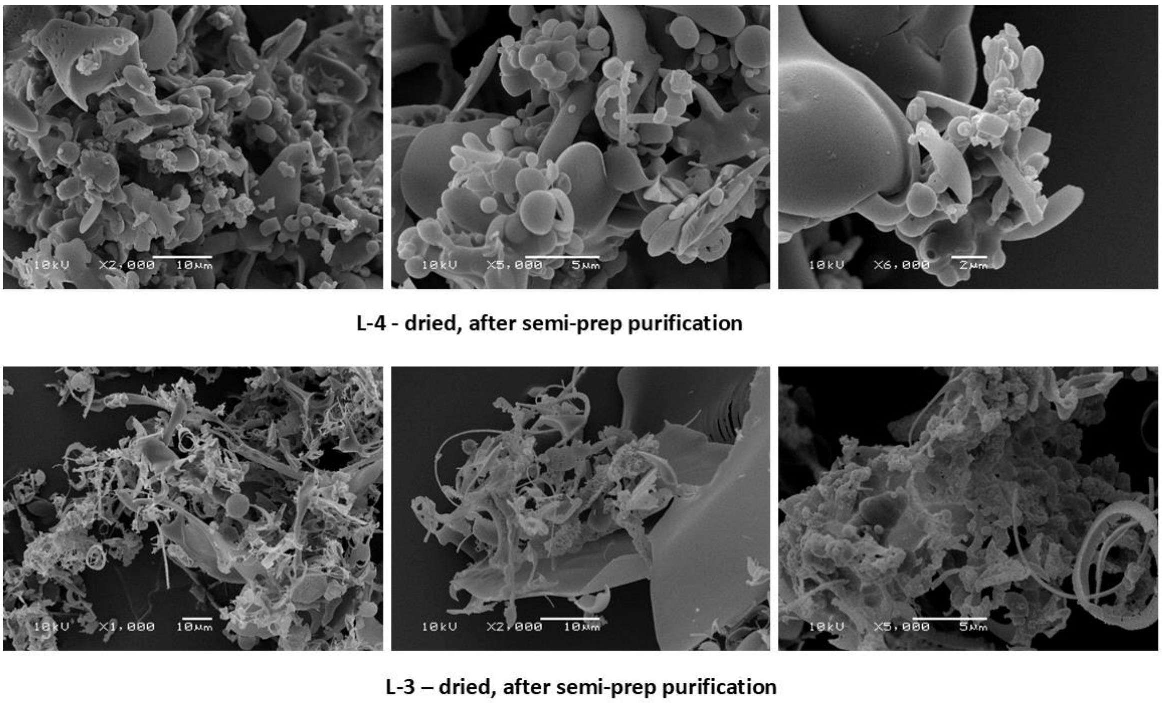

Figure S64. FE SEM micrographs of a) ***L*-3** and b) ***L*-4** after HPLC purification and freeze-drying overnight.

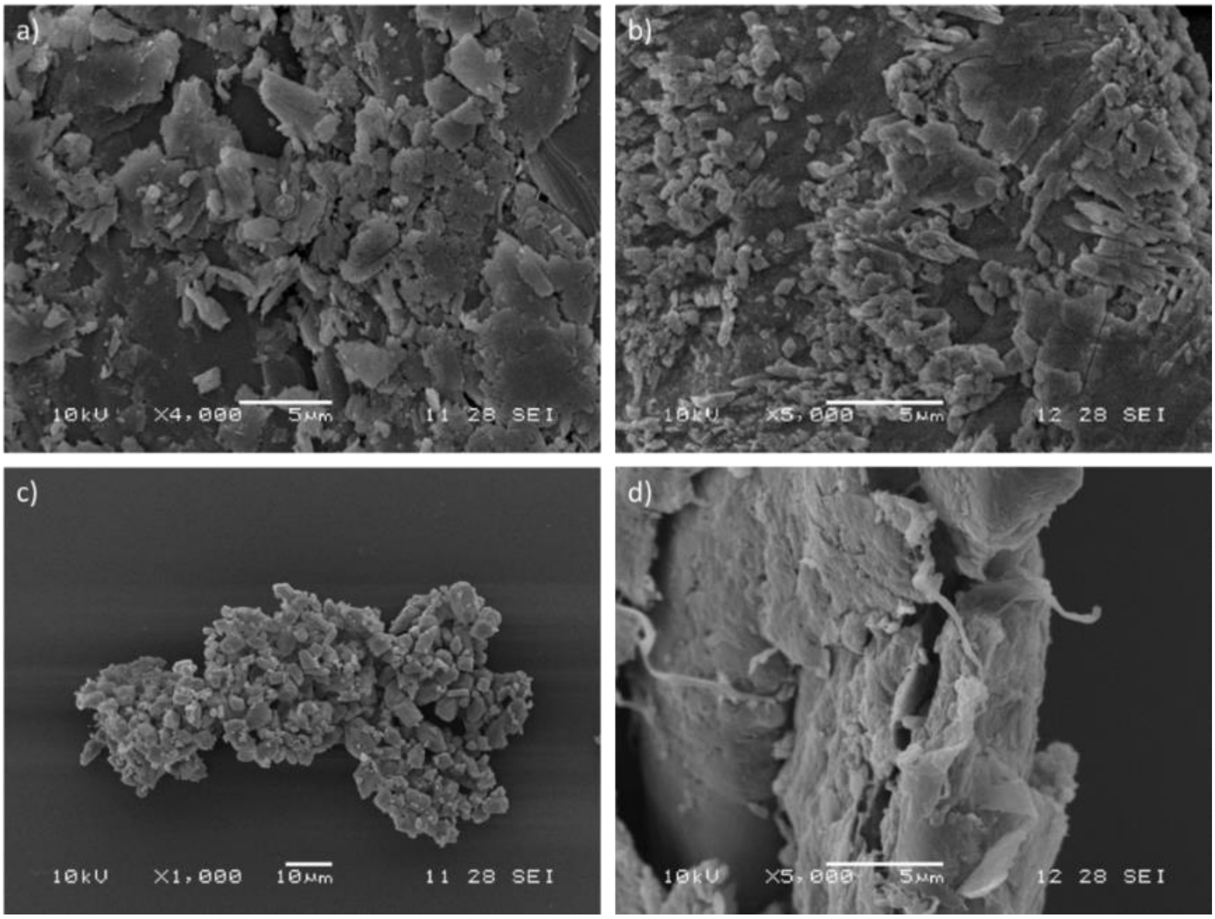

Figure S65. Alternative FE SEM micrographs of a) ***L*-3**, b) ***D*-3**, c) ***L*-4** and d) ***D*-4** after freeze-drying 1 mg/mL solutions of H_2O:CH_3CN (1:1).

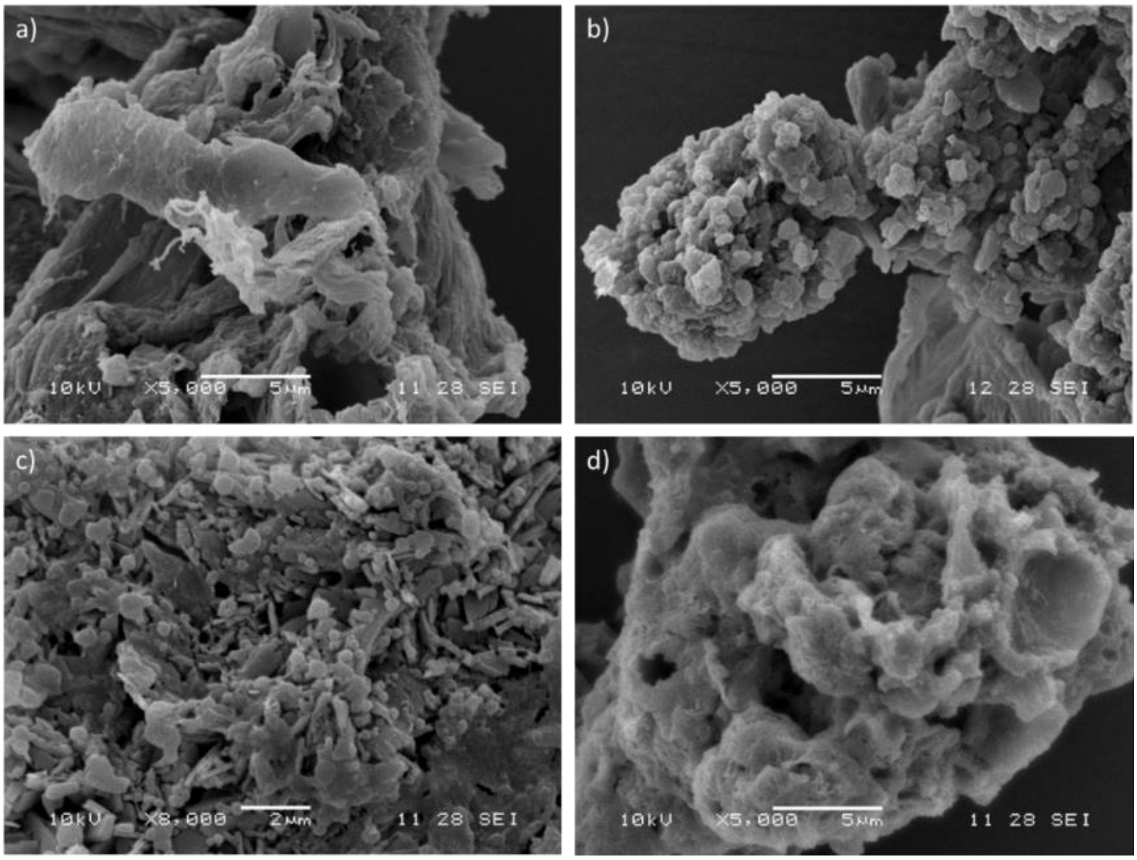

Figure S66. Alternative FE SEM micrographs of a) ***L*-4**, b) ***D*-4**, c) ***L*-3** and d) ***D*-3** after the overnight freeze-drying of corresponding 1 mg/mL solutions of DMSO (1 mg.mL).

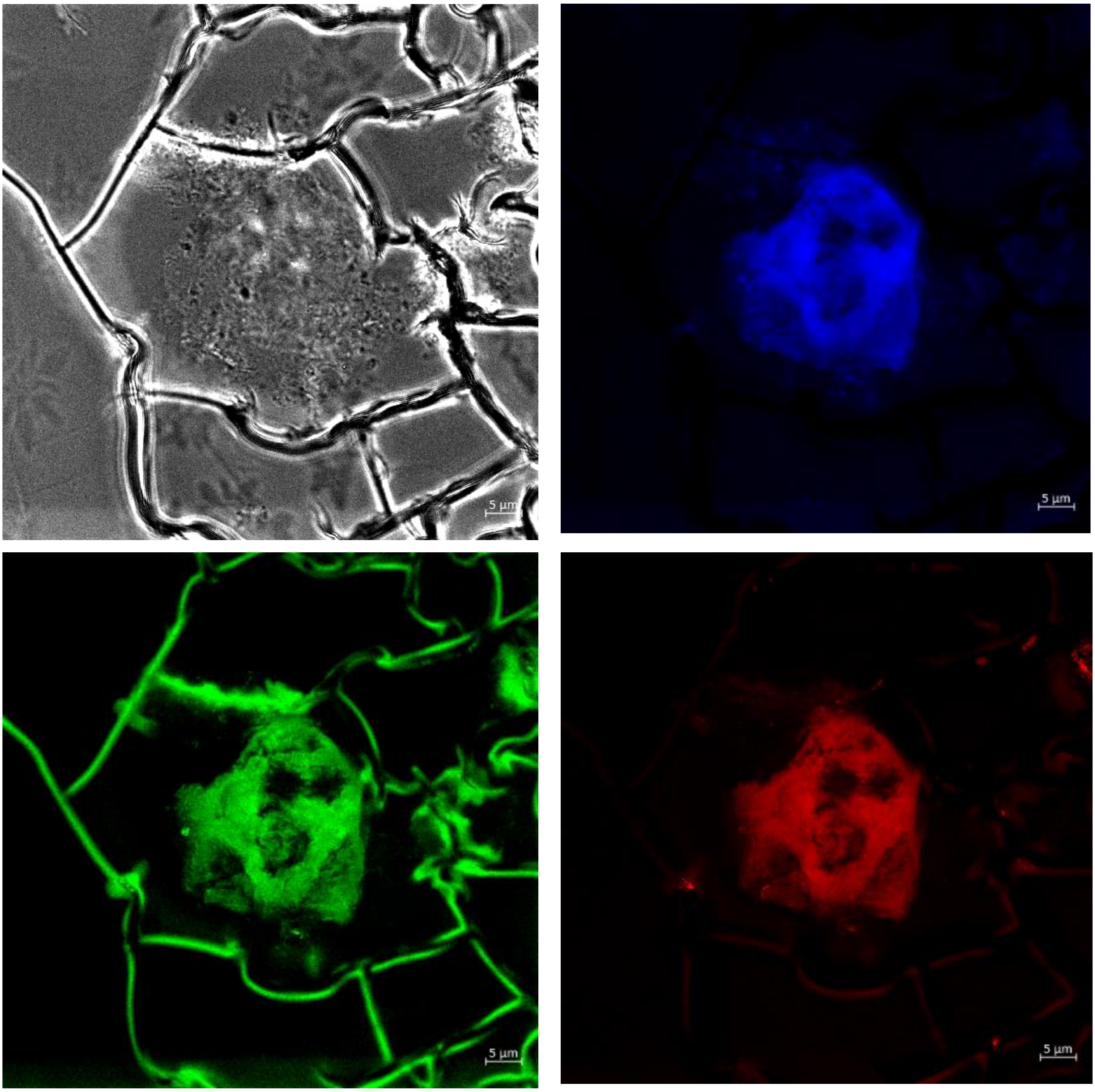

Figure S67. Selected high magnification confocal laser-scanning micrographs (obtained with Airyscan detector) of compound ***L*-3** showing bright field; blue channel: λ_{ex} = 405 nm, λ_{em} = 420-480 nm; green channel: λ_{ex} = 488 nm, λ_{em} = 495-550 nm; red channel: λ_{ex} = 561 nm, λ_{em} = 555-620 nm. The sample was placed in thin film on a Petri dish and freeze-dried overnight. Scale bar = 5 µm.

11. *In vitro* assays

For the two photon imaging experiments, laser light at a wavelength of 580-630 nm was obtained from an optical parametric oscillator pumped by a mode locked Mira titanium sapphire laser (Coherent Lasers Ltd) producing 180 fs pulses at 75 MHz. This laser was pumped by a solid state continuous wave 532 nm laser (Verdi V18, Coherent Laser Ltd). The oscillator fundamental output of 810±2 nm was used. The laser beam was focused to a diffraction limited spot through a water immersion ultraviolet corrected objective (Nikon VC x60, NA1.2) and specimens illuminated at the microscope stage of a modified Nikon TE2000-U. The focused laser spot was raster scanned using an X-Y galvanometer (GSI Lumonics). Fluorescence emission was collected without de-scanning, bypassing the scanning system and passed through a colored glass (BG39) filter. The scan was operated in normal mode and line, frame and pixel clock signals were generated and synchronised with an external fast microchannel plate photomultiplier tube used as the detector (R3809-U, Hamamatsu, Japan). These were linked *via* a Time-Correlated Single Photon Counting (TCSPC) PC module SPC830. Emission spectral detection was carried out using an Acton Research Component 275 spectrometer and an Andor iDus 740-BU CCD camera.

PC-3 cells culture and fluorescence imaging

PC-3 cells were cultured at 37 °C in a humidified atmosphere of 5% CO_2 in air and split once confluence had been reached, using DMEM medium with 10% foetal calf serum (FCS), 200 U mL^{-1} *L*-glutamine and 100 U mL-1 penicillin. The medium contained no fluorescent indicator dyes such as phenol red and was therefore suitable for use in fluorescence imaging studies.
Samples for fluorescence imaging were prepared in the following way: surplus supernatant after culturing (containing dead cell matter and excess proteins) was discarded. The live adherent cells were then washed with two 5 mL aliquots of phosphate buffer saline solution to remove any remaining medium containing FCS since this contains protease inhibitors which inactivate trypsin, thus inhibiting the resuspension of the cells. For re-suspension in solution, the cells were incubated in 3 mL of trypsin–EDTA (500 mg trypsin, 200 mg EDTA) solution for 5 min at 37 °C. After trypsinizing, fresh DMEM (10% FCS) was added to the suspended cells to give a sufficient concentration of cells (ca. 50000 cells/mL). The cells were plated in a Petri dish with a glass cover slip (MaTek) and left for 24 h to adhere before fluorescence imaging measurements were made.

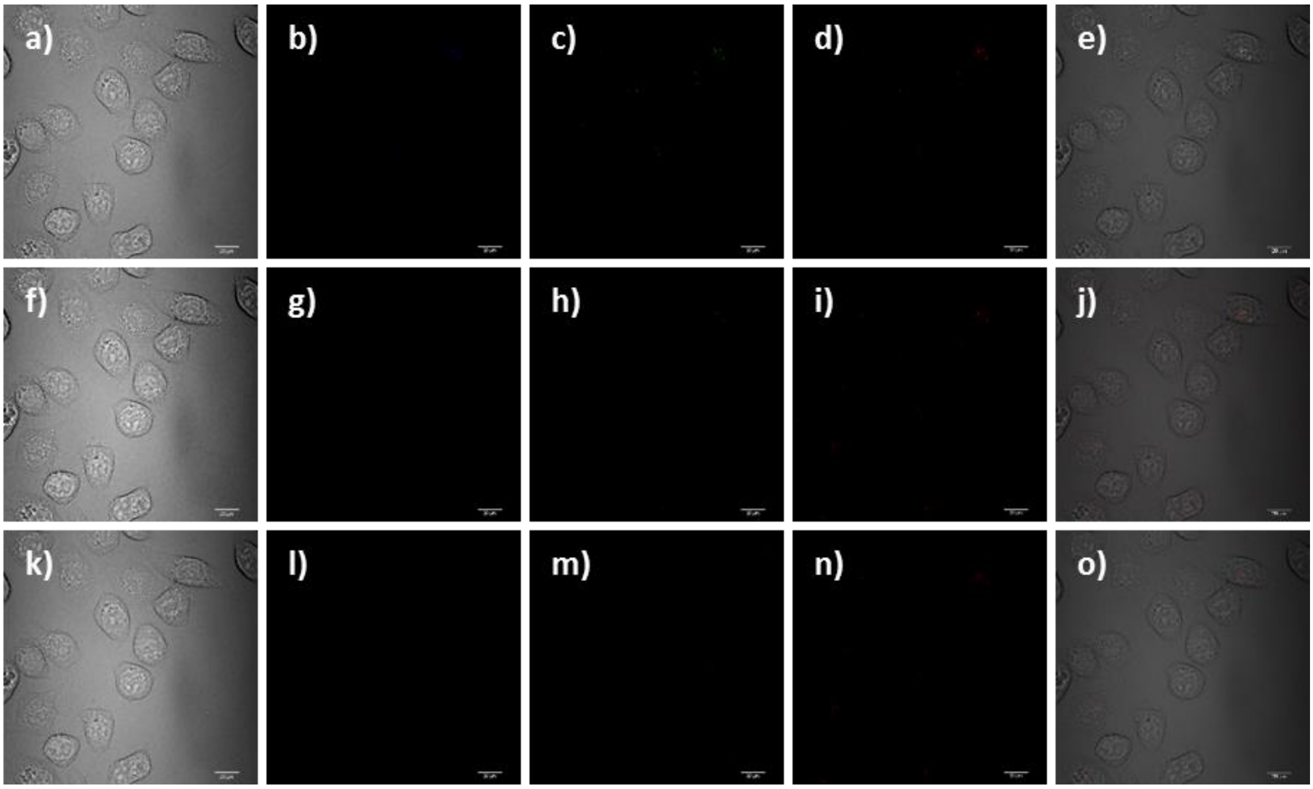

Figure S68. Confocal laser-scanning microscopy of PC-3 cells incubated at 37 °C for 18 h with: a-o) 1% DMSO control. a,f,k) bright field channel; b,g,l) blue channel (λ_{em}=417-477 nm); c,h,m) green channel (λ_{em}=500-550 nm); d,i,n) red channel (λ_{em}=570-750 nm); e,j,o) overlay of the blue-green-red channels. a-e) λ_{ex}=405 nm; f-j) λ_{ex}=488 nm; k-o) λ_{ex}=561 nm. Scale bar: 20 µm.

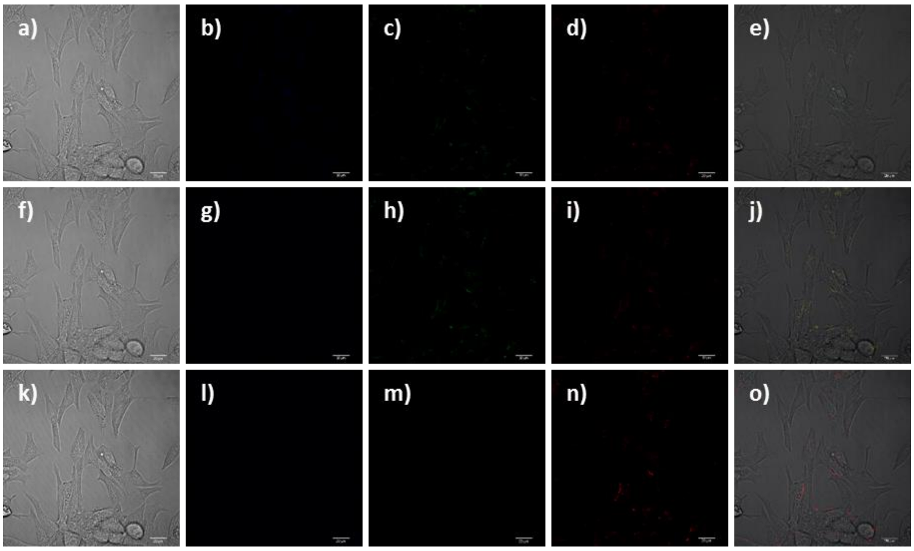

Figure S69. Confocal laser-scanning microscopy of LnCap cells incubated at 37 °C for 18 h with a-o) 1% DMSO control. a,f,k) bright field channel; b,g,l) blue channel (λ_{em}=417-477 nm); c,h,m) green channel (λ_{em}=500-550 nm); d,i,n) red channel (λ_{em}=570-750 nm); e,j,o) overlay of the blue-green-red channels. a-e) λ_{ex}=405 nm; f-j) λ_{ex}=488 nm; k-o) λ_{ex}=561 nm. Scale bar: 20 µm.

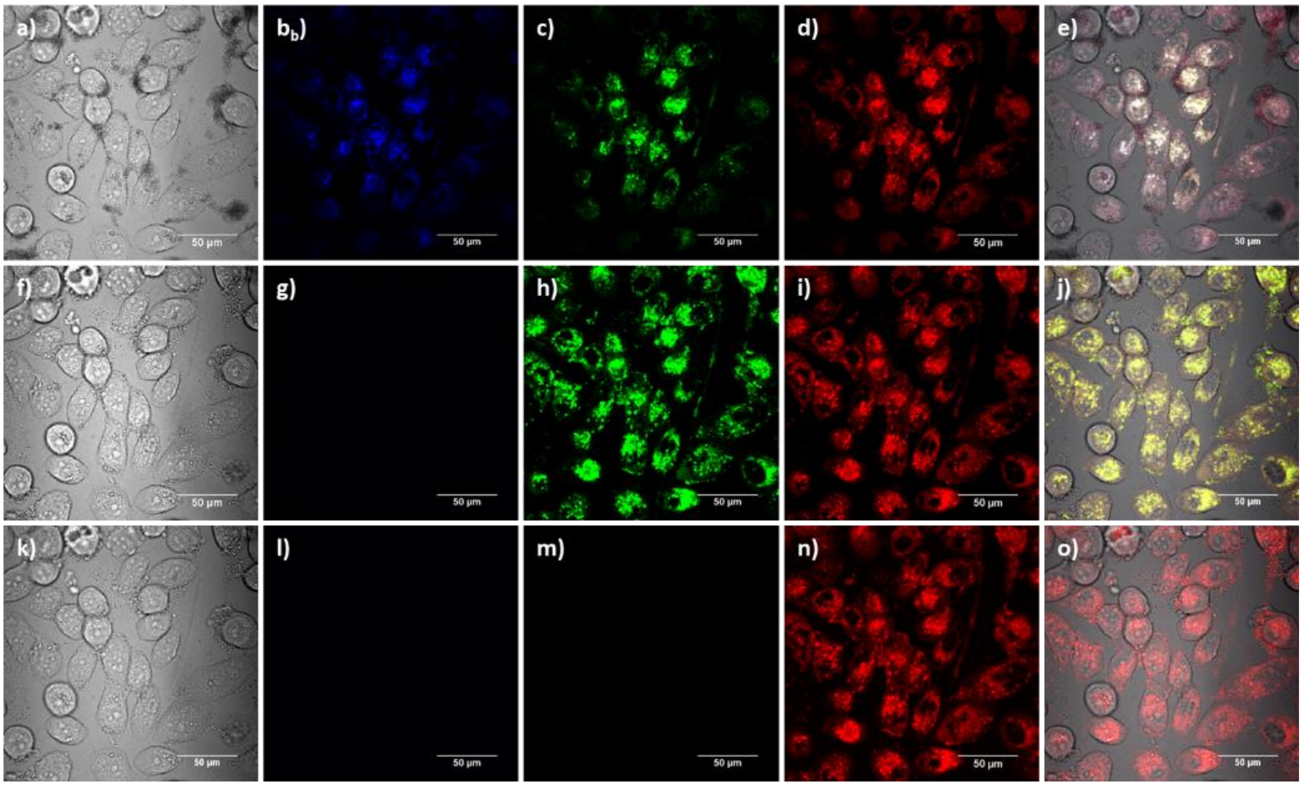

Figure S70. Confocal laser-scanning microscopy of PC-3 cells incubated at 37 °C for 18 h with: a-o) ***L*-1** (100 µM, in 1:99 DMSO:serum-free medium). a,f,k) bright field channel; b,g,l) blue channel (λ_{em}=417-477 nm); c,h,m) green channel (λ_{em}=500-550 nm); d,i,n) red channel (λ_{em}=570-750 nm); e,j,o) overlay of the blue-green-red channels. a-e) λ_{ex}=405 nm; f-j) λ_{ex}=488 nm; k-o) λ_{ex}=561 nm. Scale bar: 20 µm.

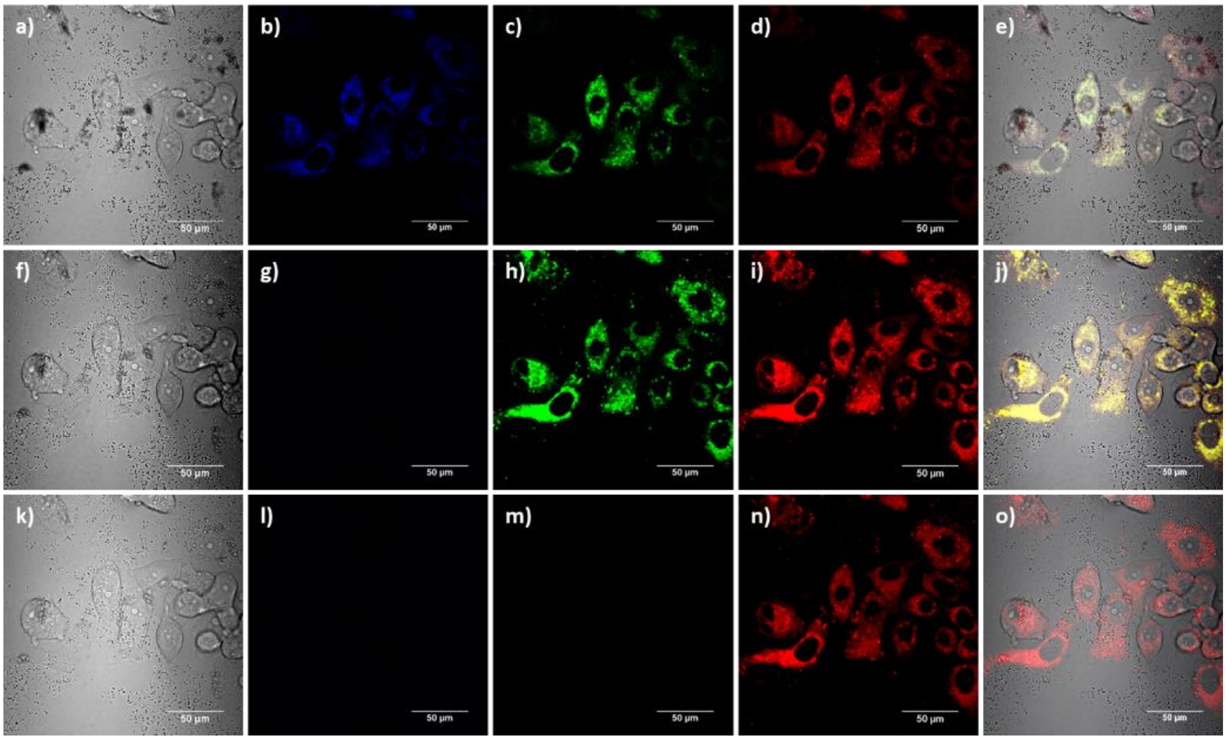

Figure S71. Confocal laser-scanning microscopy of PC-3 cells incubated at 37 °C for 18 h with a-o) ***D*-1** (100 µM, in 1:99 DMSO:serum-free medium). a,f,k) bright field channel; b,g,l) blue channel (λ_{em}=417-477 nm); c,h,m) green channel (λ_{em}=500-550 nm); d,i,n) red channel (λ_{em}=570-750 nm); e,j,o) overlay of the blue-green-red channels. a-e) λ_{ex}=405 nm; f-j) λ_{ex}=488 nm; k-o) λ_{ex}=561 nm. Scale bar: 50 µm.

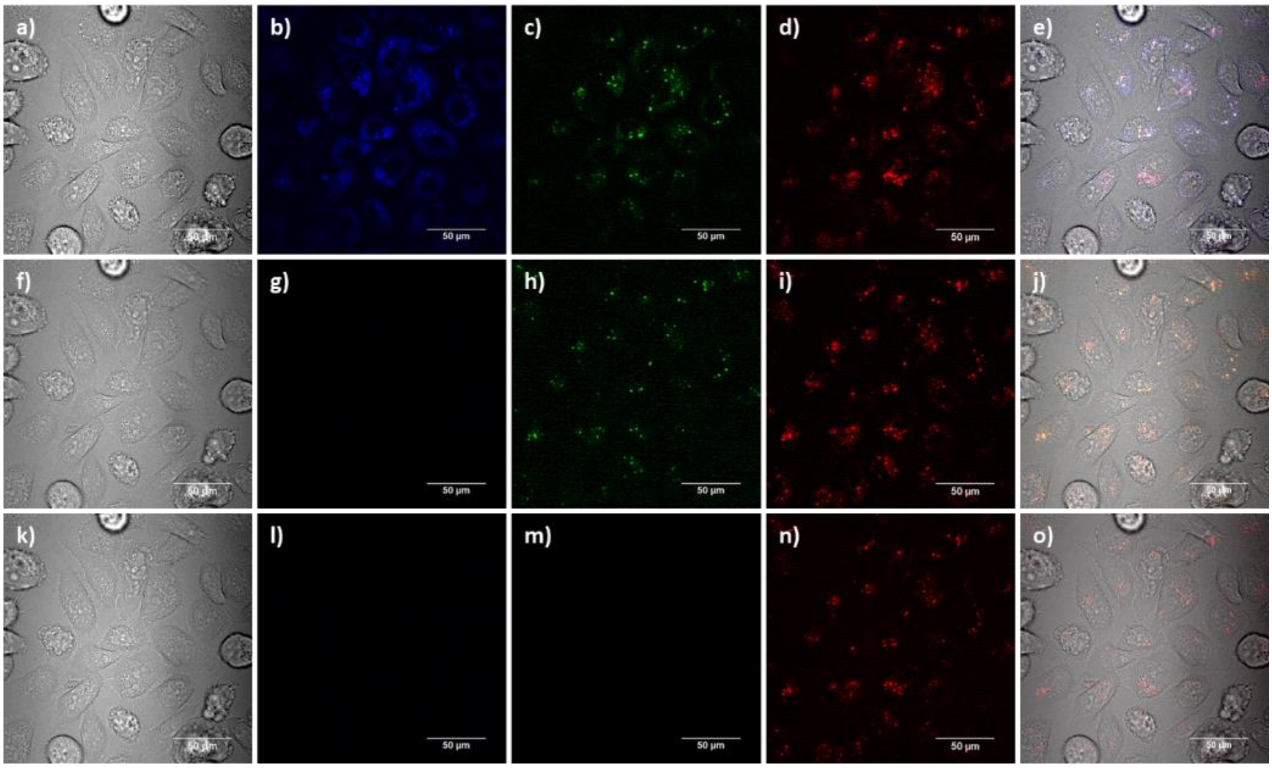

Figure S72. Confocal laser-scanning microscopy of PC-3 cells incubated at 37 °C for 18 h with a-o) ***L*-2** (100 µM, in 1:99 DMSO:serum-free medium). a,f,k) bright field channel; b,g,l) blue channel (λ_{em}=417-477 nm); c,h,m) green channel (λ_{em}=500-550 nm); d,i,n) red channel (λ_{em}=570-750 nm); e,j,o) overlay of the blue-green-red channels. a-e) λ_{ex}=405 nm; f-j) λ_{ex}=488 nm; k-o) λ_{ex}=561 nm. Scale bar: 20 µm.

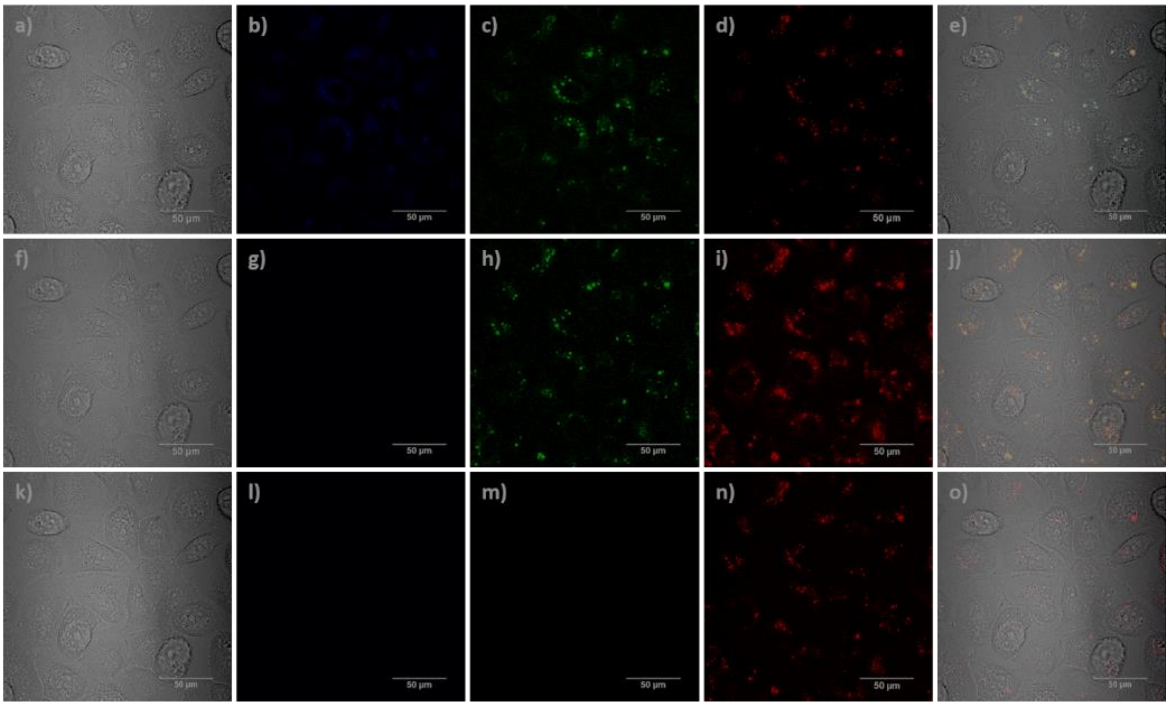

Figure S73. Confocal laser-scanning microscopy of PC-3 cells incubated at 37 °C for 18 h with a-o) ***D*-2** (100 µM in 1:99 DMSO:serum-free medium). a,f,k) bright field channel; b,g,l) blue channel (λ_{em}=417-477 nm); c,h,m) green channel (λ_{em}=500-550 nm); d,i,n) red channel (λ_{em}=570-750 nm); e,j,o) overlay of the blue-green-red channels. a-e) λ_{ex}=405 nm; f-j) λ_{ex}=488 nm; k-o) λ_{ex}=561 nm. Scale bar: 20 µm.

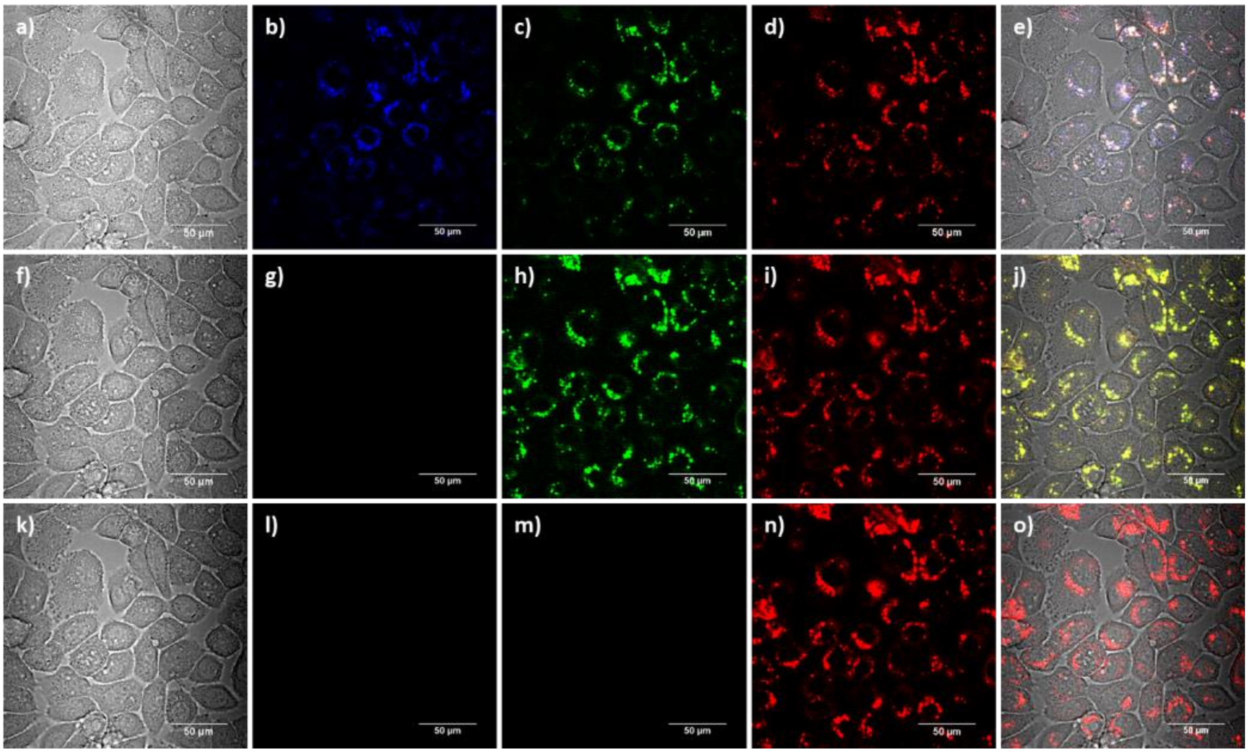

Figure S74. Confocal laser-scanning microscopy of PC-3 cells incubated at 37 °C for 18 h with a-o) ***L*-3** (100 µM in 1:99 DMSO:serum-free medium). a,f,k) bright field channel; b,g,l) blue channel (λ_{em}=417-477 nm); c,h,m) green channel (λ_{em}=500-550 nm); d,i,n) red channel (λ_{em}=570-750 nm); e,j,o) overlay of the blue-green-red channels. a-e) λ_{ex}=405 nm; f-j) λ_{ex}=488 nm; k-o) λ_{ex}=561 nm. Scale bar: 20 µm.

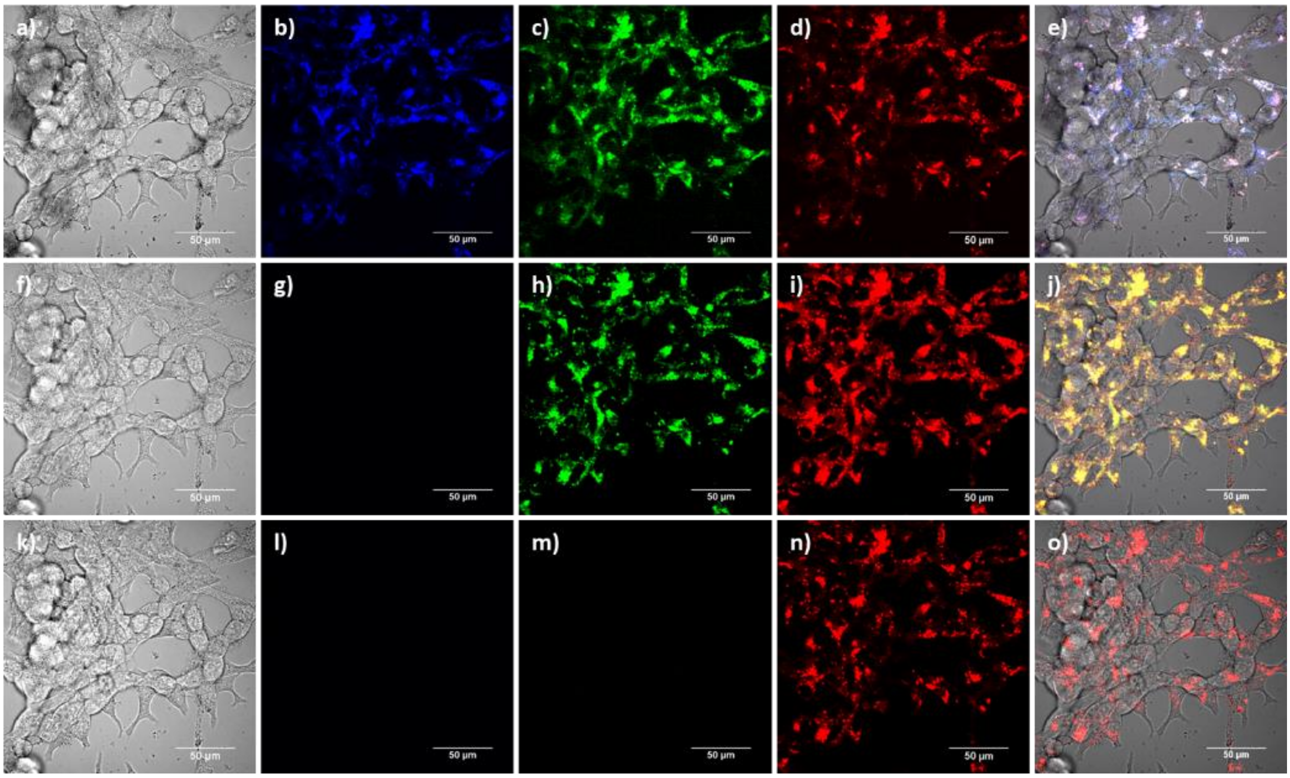

Figure S75. Confocal laser-scanning microscopy of LnCap cells incubated at 37 °C for 18 h with a-o) ***L*-3** (100 µM in 1:99 DMSO:serum-free medium). a,f,k) bright field channel; b,g,l) blue channel (λ_{em}=417-477 nm); c,h,m) green channel (λ_{em}=500-550 nm); d,i,n) red channel (λ_{em}=570-750 nm); e,j,o) overlay of the blue-green-red channels. a-e) λ_{ex}=405 nm; f-j) λ_{ex}=488 nm; k-o) λ_{ex}=561 nm. Scale bar: 20 µm.

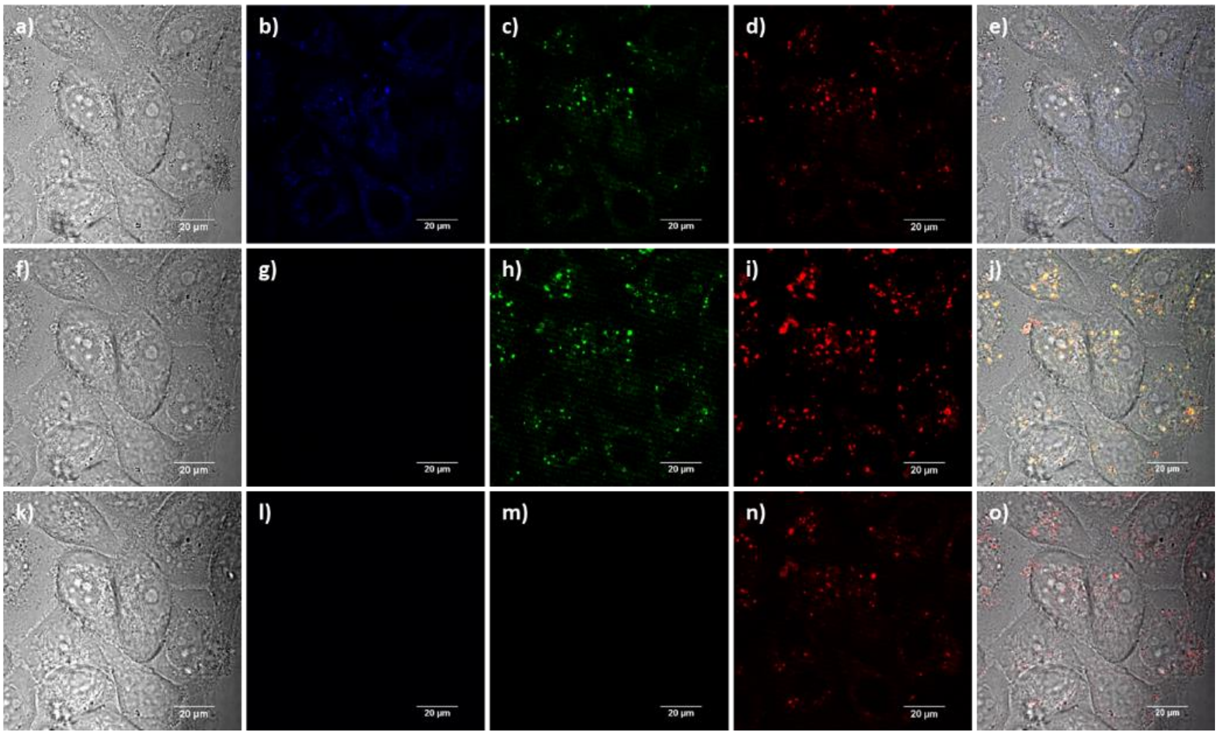

Figure S76. Confocal laser-scanning microscopy of PC-3 cells incubated at 37 °C for 18 h with a-o) ***D*-3** (100 µM in 1:99 DMSO:serum-free medium). a,f,k) bright field channel; b,g,l) blue channel (λ_{em}=417-477 nm); c,h,m) green channel (λ_{em}=500-550 nm); d,i,n) red channel (λ_{em}=570-750 nm); e,j,o) overlay of the blue-green-red channels. a-e) λ_{ex}=405 nm; f-j) λ_{ex}=488 nm; k-o) λ_{ex}=561 nm. Scale bar: 20 µm.

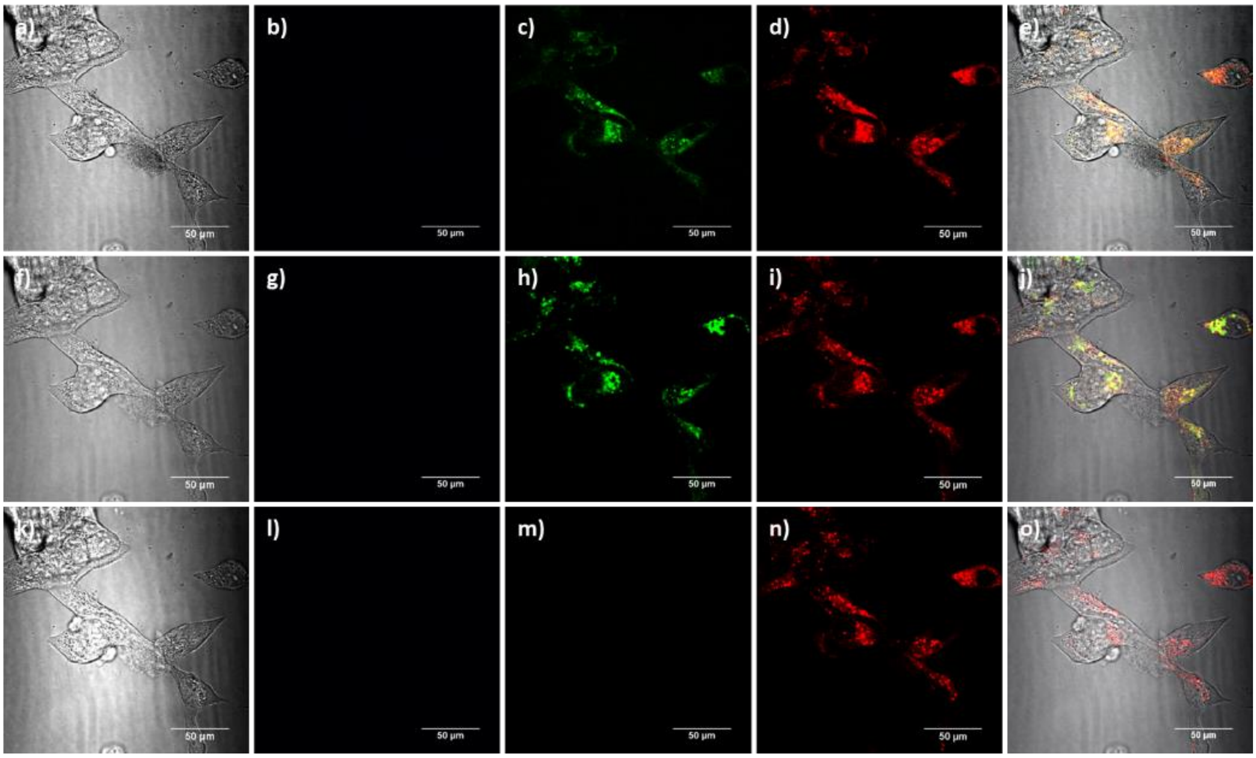

Figure S77. Confocal laser-scanning microscopy of LnCap cells incubated at 37 °C for 18 h with a-o) ***D*-3** (100 µM in 1:99 DMSO:serum-free medium). a,f,k) bright field channel; b,g,l) blue channel (λ_{em}=417-477 nm); c,h,m) green channel (λ_{em}=500-550 nm); d,i,n) red channel (λ_{em}=570-750 nm); e,j,o) overlay of the blue-green-red channels. a-e) λ_{ex}=405 nm; f-j) λ_{ex}=488 nm; k-o) λ_{ex}=561 nm. Scale bar: 20 µm.

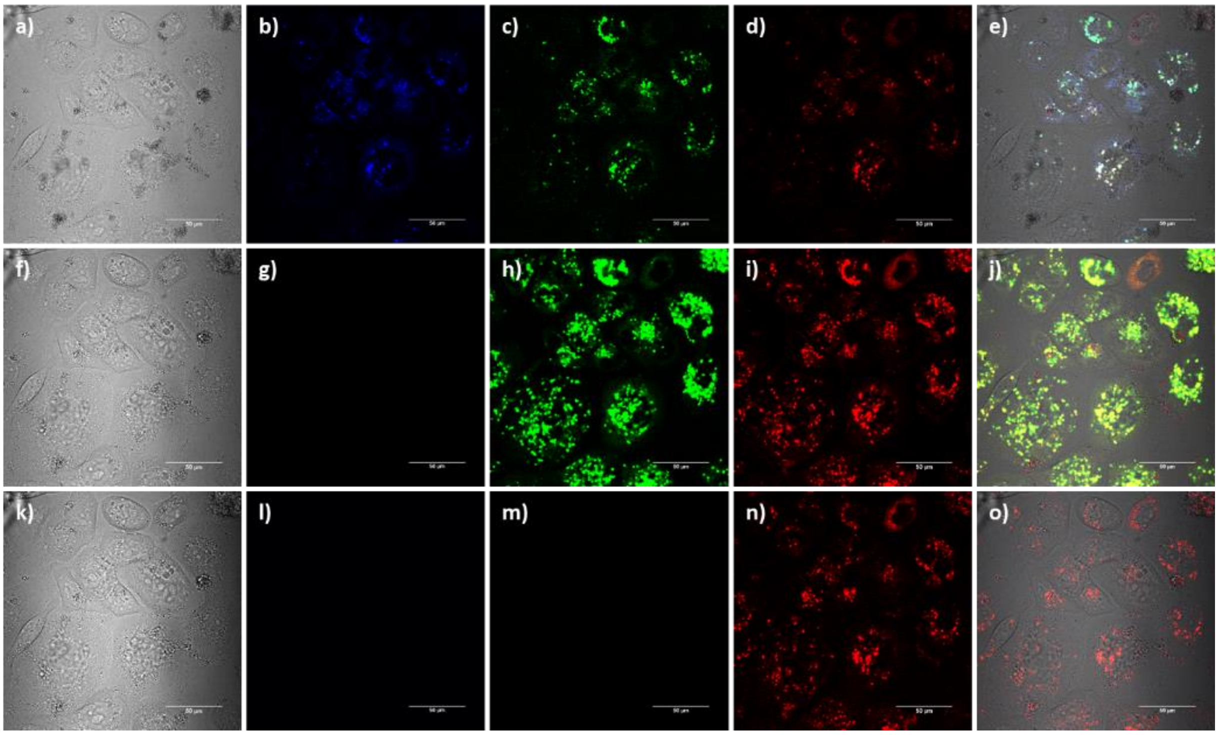

Figure S78. Confocal laser-scanning microscopy of PC-3 cells incubated at 37 °C for 18 h with a-o) ***L*-4** (100 µM in 1:99 DMSO:serum-free medium). a,f,k) bright field channel; b,g,l) blue channel (λ_{em}=417-477 nm); c,h,m) green channel (λ_{em}=500-550 nm); d,i,n) red channel (λ_{em}=570-750 nm); e,j,o) overlay of the blue-green-red channels. a-e) λ_{ex}=405 nm; f-j) λ_{ex}=488 nm; k-o) λ_{ex}=561 nm. Scale bar: 20 µm.

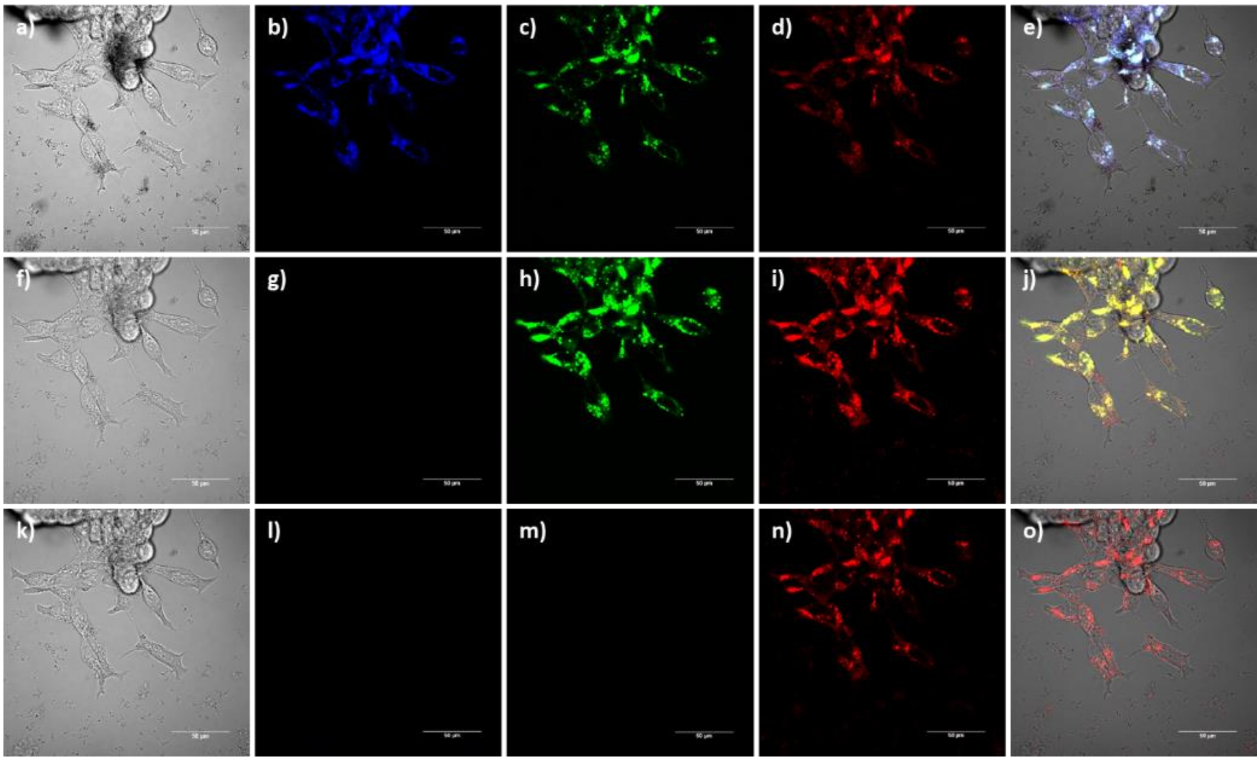

Figure S79. Confocal laser-scanning microscopy of LnCap cells incubated at 37 °C for 18 h with a-o) ***L*-4** (100 µM in 1:99 DMSO:serum-free medium). a,f,k) bright field channel; b,g,l) blue channel (λ_{em}=417-477 nm); c,h,m) green channel (λ_{em}=500-550 nm); d,i,n) red channel (λ_{em}=570-750 nm); e,j,o) overlay of the blue-green-red channels. a-e) λ_{ex}=405 nm; f-j) λ_{ex}=488 nm; k-o) λ_{ex}=561 nm. Scale bar: 20 µm.

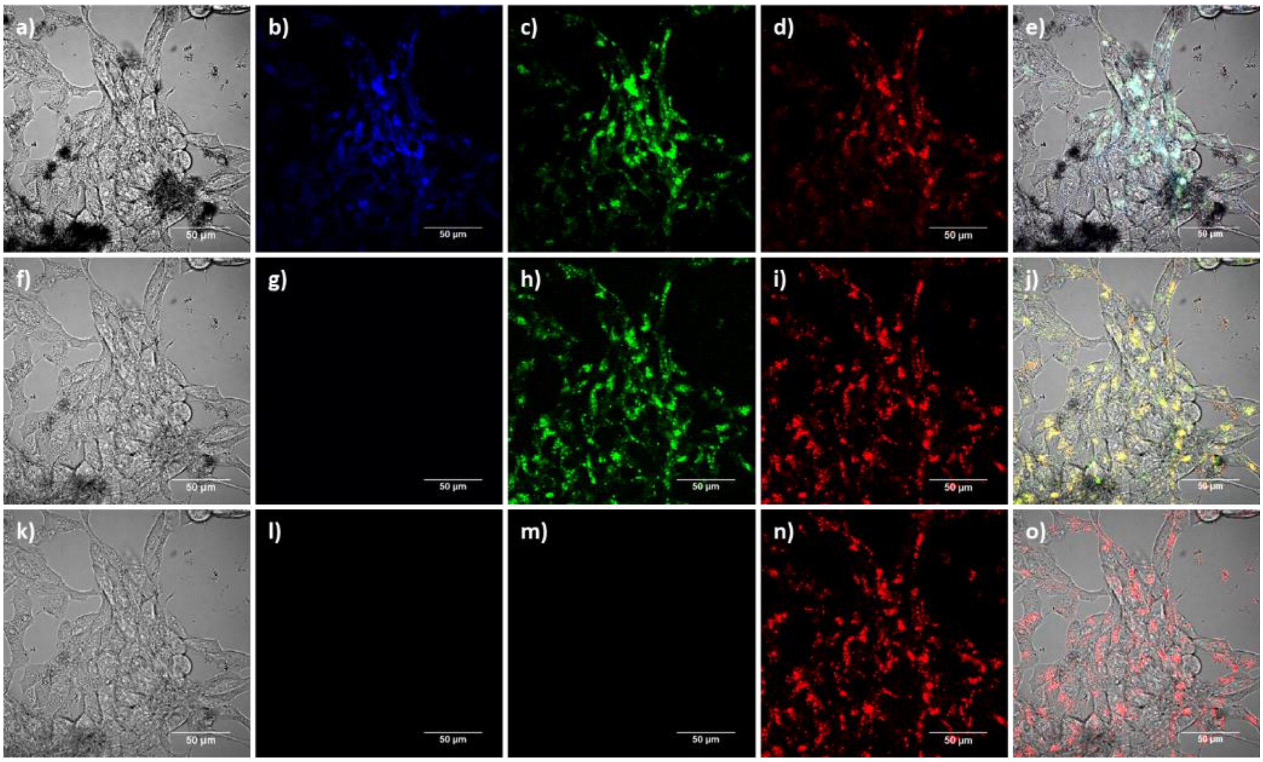

Figure S80. Confocal laser-scanning microscopy of PC-3 cells incubated at 37 °C for 18 h with a-o) ***D*-4** (100 µM in 1:99 DMSO:serum-free medium). a,f,k) bright field channel; b,g,l) blue channel (λ_{em}=417-477 nm); c,h,m) green channel (λ_{em}=500-550 nm); d,i,n) red channel (λ_{em}=570-750 nm); e,j,o) overlay of the blue-green-red channels. a-e) λ_{ex}=405 nm; f-j) λ_{ex}=488 nm; k-o) λ_{ex}=561 nm. Scale bar: 20 µm.

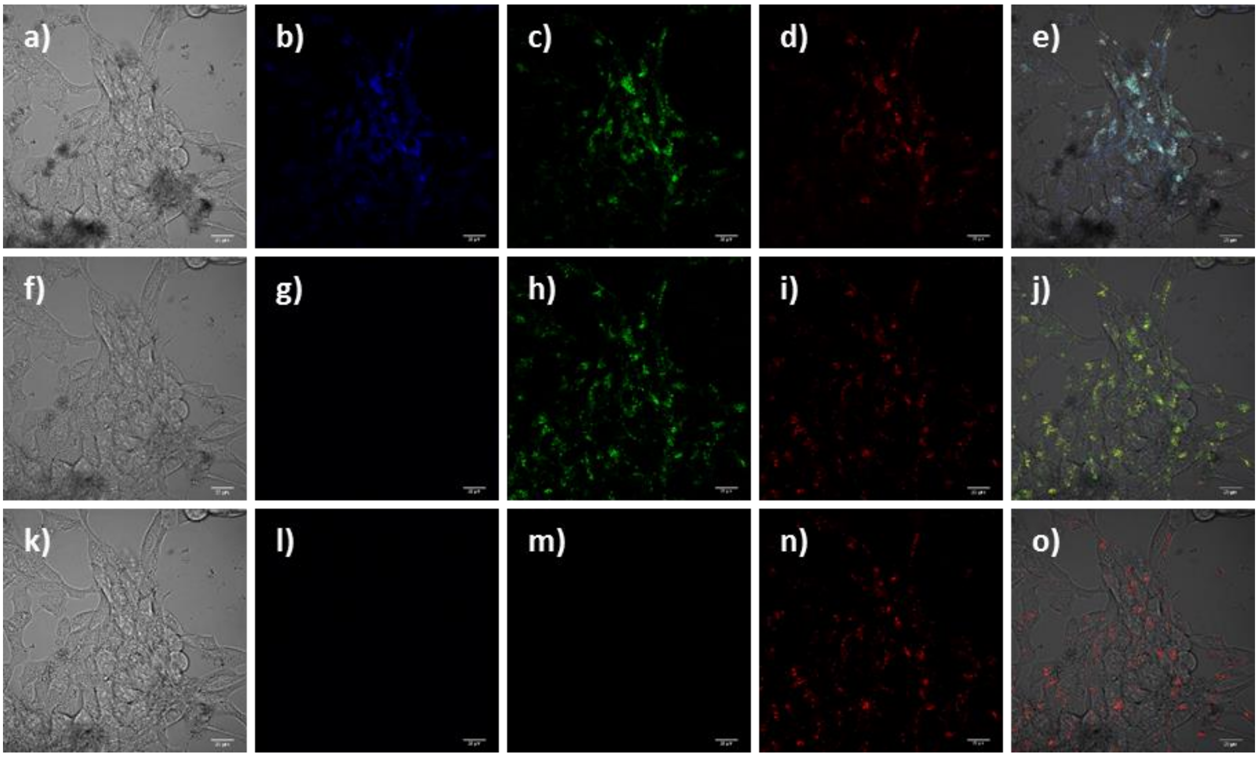

Figure S81. Confocal laser-scanning microscopy of LnCap cells incubated at 37 °C for 18 h with a-o) ***D*-4** (100 µM in 1:99 DMSO:serum-free medium). a,f,k) bright field channel; b,g,l) blue channel (λ_{em}=417-477 nm); c,h,m) green channel (λ_{em}=500-550 nm); d,i,n) red channel (λ_{em}=570-750 nm); e,j,o) overlay of the blue-green-red channels. a-e) λ_{ex}=405 nm; f-j) λ_{ex}=488 nm; k-o) λ_{ex}=561 nm. Scale bar: 20 µm.

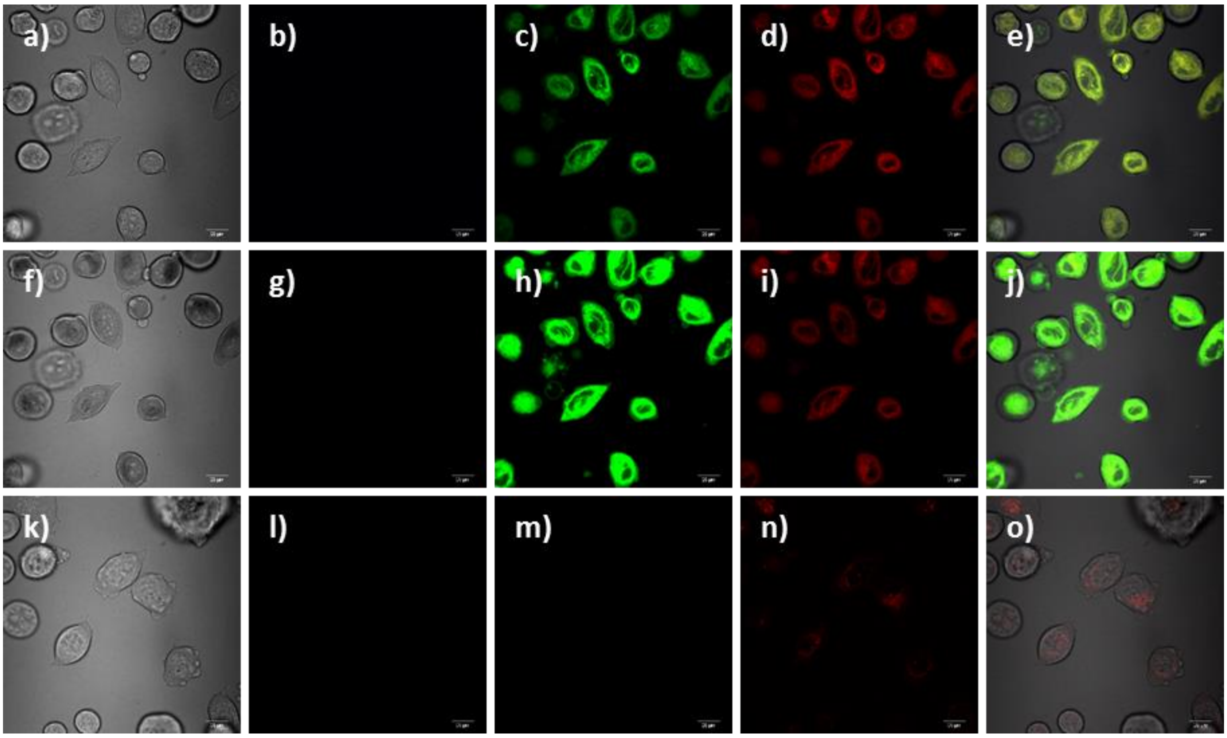

Figure S82. Confocal laser-scanning microscopy of LnCap cells incubated at 37 °C for 18 h with a-o) **5** (100 µM in 1:99 DMSO:serum-free medium). a,f,k) bright field channel; b,g,l) blue channel (λ_{em}=417-477 nm); c,h,m) green channel (λ_{em}=500-550 nm); d,i,n) red channel (λ_{em}=570-750 nm); e,j,o) overlay of the blue-green-red channels. a-e) λ_{ex}=405 nm; f-j) λ_{ex}=488 nm; k-o) λ_{ex}=561.0 nm. Scale bar: 20 µm.

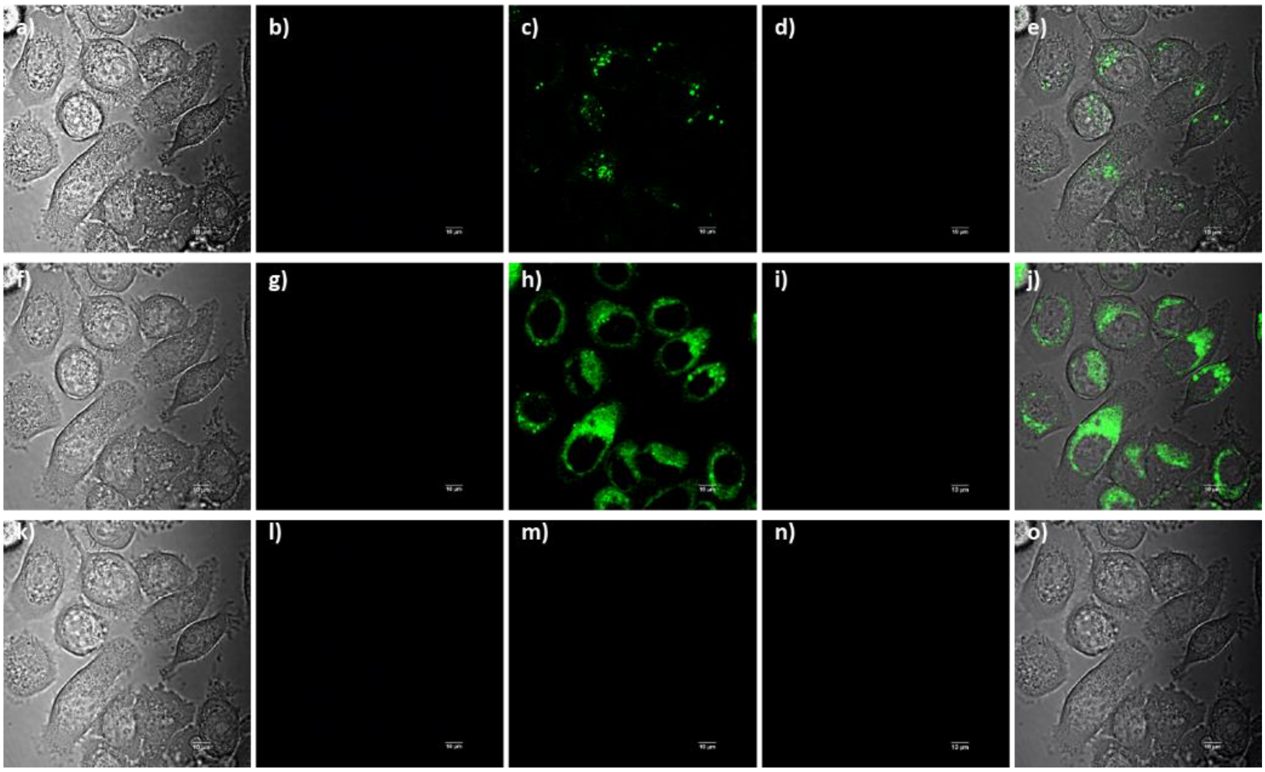

Figure S83. Confocal laser-scanning microscopy of PC-3 cells incubated at 37 °C for 18 h with a-o) ***L*-6** (100 µM in 1:99 DMSO:serum-free medium). a,f,k) bright field channel; b,g,l) blue channel (λ_{em}=417-477 nm); c,h,m) green channel (λ_{em}=500-550 nm); d,i,n) red channel (λ_{em}=570-750 nm); e,j,o) overlay of the blue-green-red channels. a-e) λ_{ex}=405 nm; f-j) λ_{ex}=488 nm; k-o) λ_{ex}=561 nm. Scale bar: 20 µm.

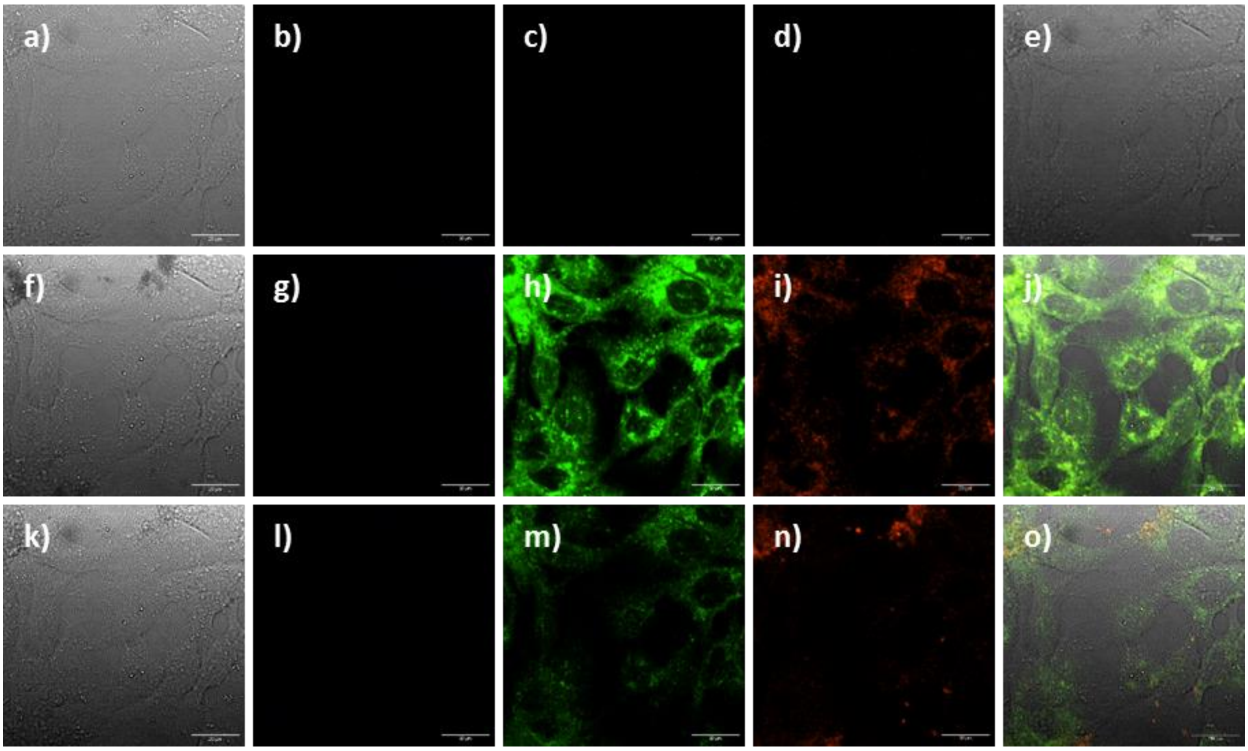

Figure S84. Confocal laser-scanning microscopy of A431 cells incubated at 37 °C for 18 h with a-o) ***L*-6** (100 µM in 1:99 DMSO:serum-free medium). a,f,k) bright field channel; b,g,l) blue channel (λ_{em}=417-477 nm); c,h,m) green channel (λ_{em}=500-550 nm); d,i,n) red channel (λ_{em}=570-750 nm); e,j,o) overlay of the blue-green-red channels. a-e) λ_{ex}=405 nm; f-j) λ_{ex}=488 nm; k-o) λ_{ex}=561 nm. Scale bar: 20 µm.

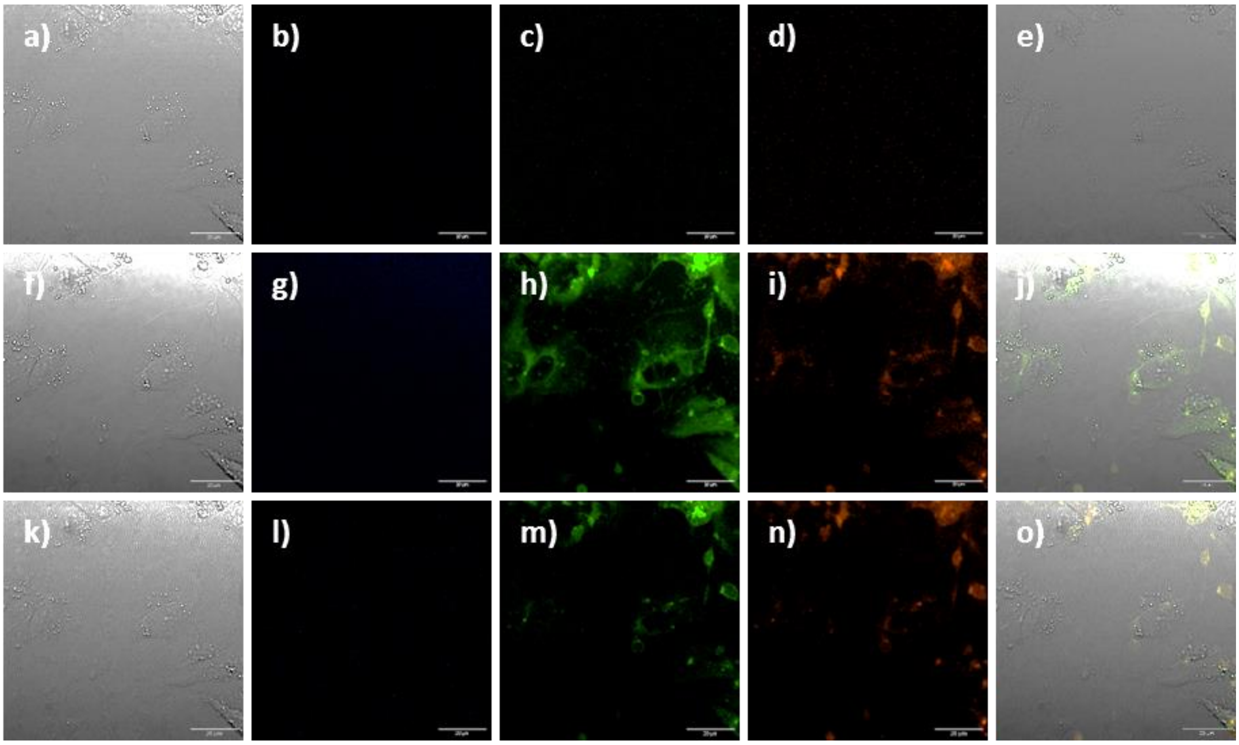

Figure S85. Confocal laser-scanning microscopy of CHO cells incubated at 37 °C for 18 h with a-o) ***L*-6** (100 µM in 1:99 DMSO:serum-free medium). a,f,k) bright field channel; b,g,l) blue channel (λ_{em}=417-477 nm); c,h,m) green channel (λ_{em}=500-550 nm); d,i,n) red channel (λ_{em}=570-750 nm); e,j,o) overlay of the blue-green-red channels. a-e) λ_{ex}=405 nm; f-j) λ_{ex}=488 nm; k-o) λ_{ex}=561.0 nm. Scale bar: 20 µm.

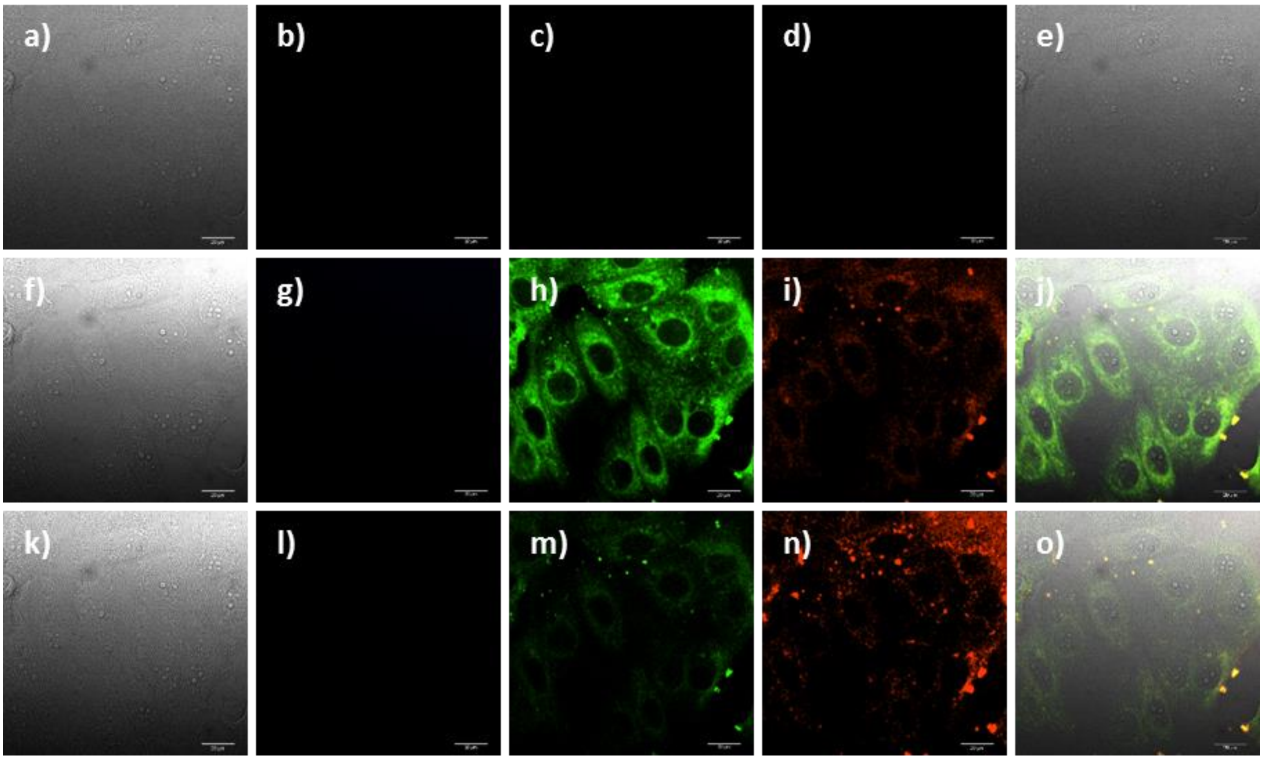

Figure S86. Confocal laser-scanning microscopy of MCF-7 cells incubated at 37 °C for 18 h with a-o) ***L*-6** (100 µM in 1:99 DMSO:serum-free medium). a,f,k) bright field channel; b,g,l) blue channel (λ_{em}=417-477 nm); c,h,m) green channel (λ_{em}=500-550 nm); d,i,n) red channel (λ_{em}=570-750 nm); e,j,o) overlay of the blue-green-red channels. a-e) λ_{ex}=405 nm; f-j) λ_{ex}=488 nm; k-o) λ_{ex}=561 nm. Scale bar: 20 µm.

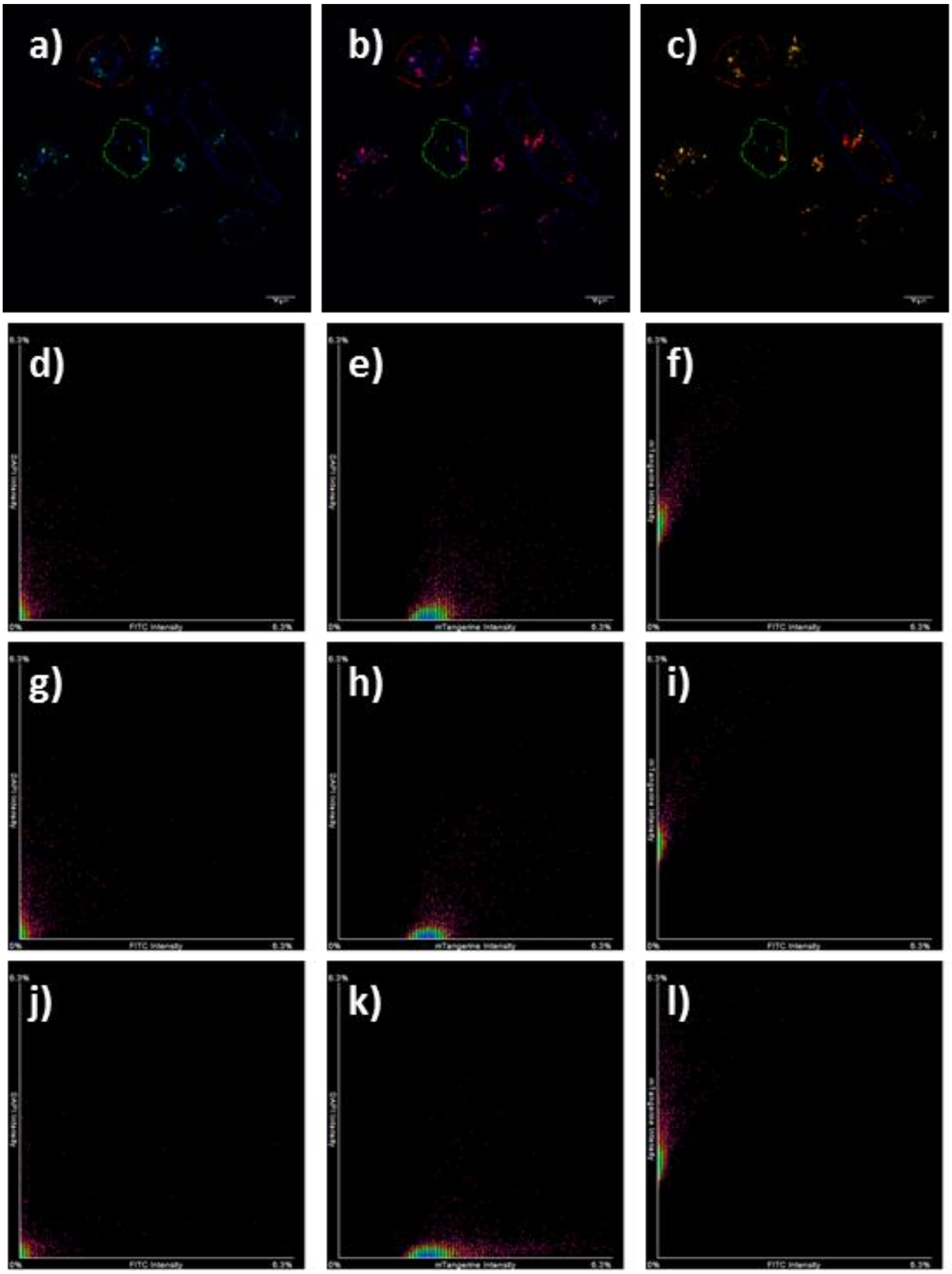

Figure S87. Confocal laser-scanning microscopy of PC-3 cells incubated at 37 °C for 18 h with a-c) ***L*-2** and Lysotracker® Blue DND-22 (100 µM and 100 nM, respectively, in 1:99 DMSO:serum-free medium). a) overlay of blue (λ_{ex}=405 nm, λ_{em}=417-477 nm) and green channels (λ_{ex}=488 nm, λ_{em}=500-550 nm); b) overlay of blue (λ_{ex}=405 nm, λ_{em}=417-477 nm) and red channels (λ_{ex}=488 nm, λ_{em}=570-750 nm); c) overlay of green (λ_{ex}=488 nm, λ_{em}=500-550 nm) and red channels (λ_{ex}=488 nm, λ_{em}=570-750 nm). Scale bar: 20 µm. d,g,j) scatterplots of blue and green pixel intensities of the cells in the indicated red ROI (d), green ROI (g) and blue ROI (j) in the micrograph a; f,i,l) scatterplots of blue and red pixel intensities of the cells in the indicated red ROI (e), green ROI (h) and blue ROI (k) in the micrograph b; scatterplots of green and red pixel intensities of the cells in the indicated red ROI (f), green ROI (i) and blue ROI (l) in the micrograph c.

Table S5. Table of the parameters of ***L*-2** extrapolated by scatterplot analysis using the software *Nikon Elements-AR Analysis 4.30.02*.

ROI	Figure	Pearson's correlation	Mander's overlap	Mander's overlap coeff. k1	Mander's overlap coeff. k2	Correlation coeff. c1	Correlation coeff. c2
Red	**a**	0.48	0.55	1.09	0.28	0.97	0.63
	**b**	0.54	0.57	0.10	3.25	0.88	1.00
	**c**	0.83	0.44	5.00	0.04	1.00	0.44
Green	**a**	0.47	0.50	0.95	0.27	0.95	0.56
	**b**	0.55	0.51	0.09	2.85	0.87	1.00
	**c**	0.85	0.36	3.84	0.03	1.00	0.35
Blue	**a**	0.34	0.40	0.49	0.32	0.92	0.45
	**b**	0.36	0.56	0.05	6.54	0.82	1.00
	**c**	0.66	0.35	5.06	0.02	1.00	0.36

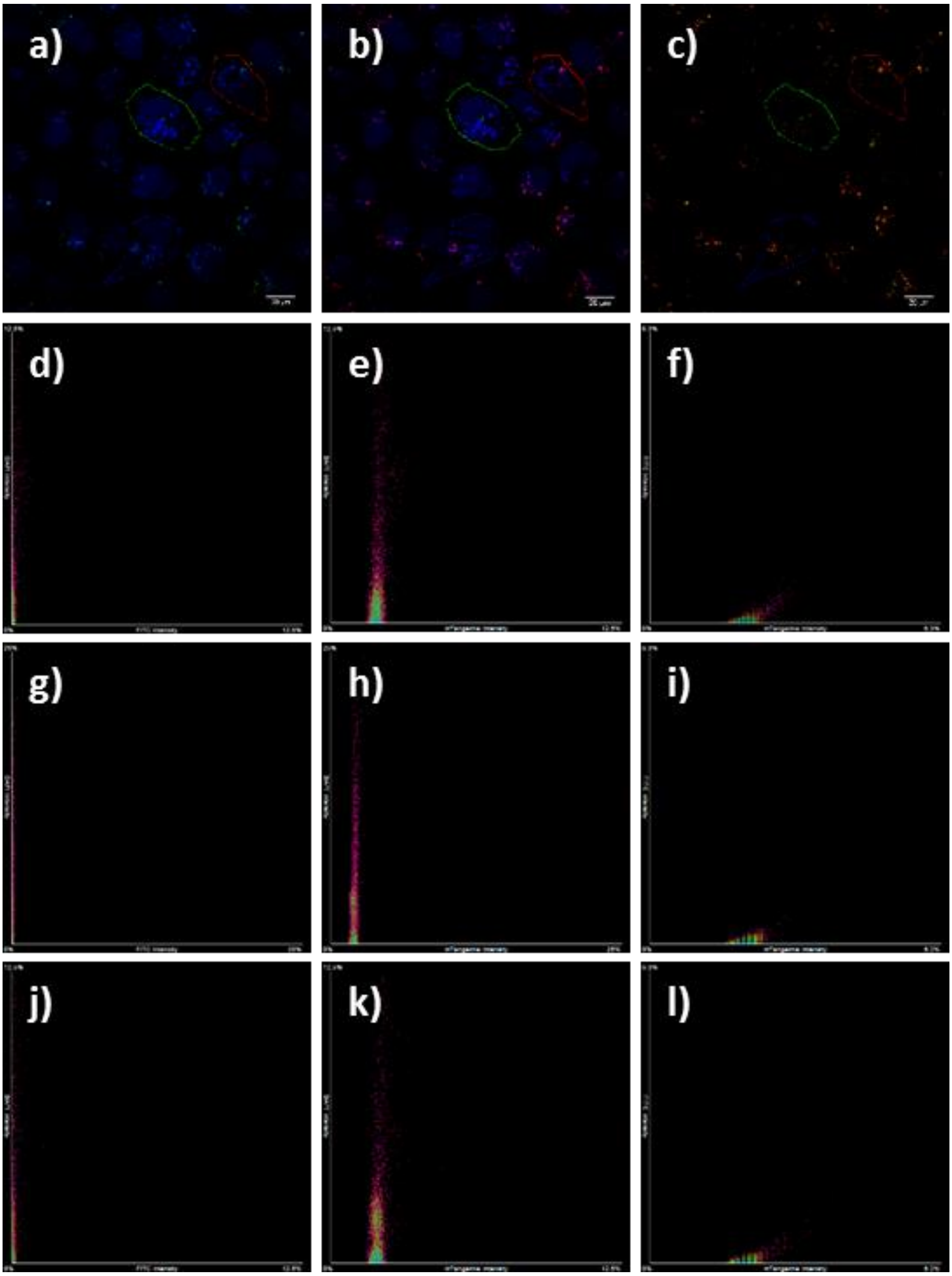

Figure S88. Confocal laser-scanning microscopy of PC-3 cells incubated at 37 °C for 18 h with a-c) ***D*-2** and Lysotracker® Blue DND-22 (100 µM and 100 nM, respectively, in 1:99 DMSO:serum-free medium). a) overlay of blue (λ_{ex}=405 nm, λ_{em}=417-477 nm) and green channels (λ_{ex}=488 nm, λ_{em}=500-550 nm); b) overlay of blue (λ_{ex}=405 nm, λ_{em}=417-477 nm) and red channels (λ_{ex}=488 nm, λ_{em}=570-750 nm); c) overlay of green (λ_{ex}=488 nm, λ_{em}=500-550 nm) and red channels (λ_{ex}=488 nm, λ_{em}=570-750 nm). Scale bar: 20 µm. d,g,j) scatterplots of blue and green pixel intensities of the cells in the indicated red ROI (d), green ROI (g) and blue ROI (j) in the micrograph a; f,i,l) scatterplots of blue and red pixel intensities of the cells in the indicated red ROI (e), green ROI (h) and blue ROI (k) in the micrograph b; scatterplots of green and red pixel intensities of the cells in the indicated red ROI (f), green ROI (i) and blue ROI (l) in the micrograph c.

Table S6. Table of the parameters of ***D*-2** extrapolated by scatterplot analysis using the software *Nikon Elements-AR Analysis 4.30.02*.

ROI	Figure	Pearson's correlation	Mander's overlap	Mander's overlap coeff. k1	Mander's overlap coeff. k2	Correlation coeff. c1	Correlation coeff. c2
Red	**a**	0.36	0.50	15.86	0.02	0.98	0.60
	**b**	0.30	0.53	0.60	0.46	0.96	1.00
	**c**	0.65	0.48	0.02	13.30	0.45	1.00
Green	**a**	0.33	0.53	37.37	0.01	0.99	0.59
	**b**	0.30	0.59	1.19	0.29	0.98	1.00
	**c**	0.57	0.56	0.02	19.70	0.47	1.00
Blue	**a**	0.31	0.47	14.51	0.02	0.98	0.57
	**b**	0.32	0.58	0.67	0.51	0.97	1.00
	**c**	0.70	0.48	0.02	13.00	0.46	1.00

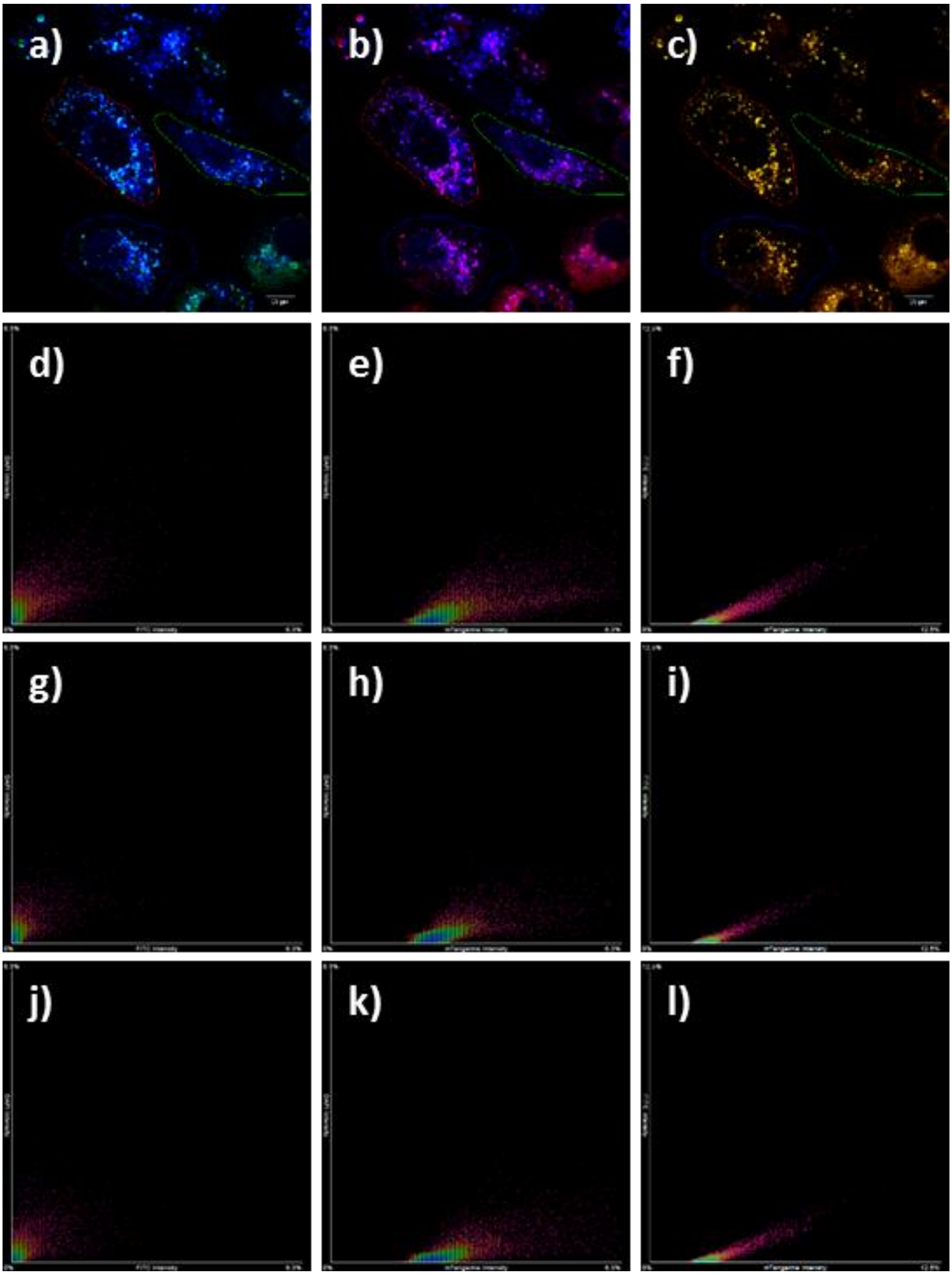

Figure S89. Confocal laser-scanning microscopy of PC-3 cells incubated at 37 °C for 18 h with a-c) ***L*-3** and Lysotracker® Blue DND-22 (100 µM and 100 nM, respectively, in 1:99 DMSO:serum-free medium). a) overlay of blue (λ_{ex} = 405.0 nm, λ_{em} = 417–477 nm) and green channels (λ_{ex} = 488.0 nm, λ_{em} = 500–550 nm); b) overlay of blue (λ_{ex} = 405.0 nm, λ_{em} = 417–477 nm) and red channels (λ_{ex} = 488.0 nm, λ_{em} = 570–750 nm); c) overlay of green (λ_{ex} = 488.0 nm, λ_{em} = 500–550 nm) and red channels (λ_{ex} = 488.0 nm, λ_{em} = 570–750 nm). Scale bar: 20 µm. d,g,j) scatterplots of blue and green pixel intensities of the cells in the indicated red ROI (d), green ROI (g) and blue ROI (j) in the micrograph a; f,i,l) scatterplots of blue and red pixel intensities of the cells in the indicated red ROI (e), green ROI (h) and blue ROI (k) in the micrograph b; scatterplots of green and red pixel intensities of the cells in the indicated red ROI (f), green ROI (i) and blue ROI (l) in the micrograph c.

Table S7. Table of the parameters of ***L*-3** extrapolated by scatterplot analysis using the software *Nikon Elements-AR Analysis 4.30.02*.

ROI	Figure	Pearson's correlation	Mander's overlap	Mander's overlap coeff. k1	Mander's overlap coeff. k2	Correlation coeff. c1	Correlation coeff. c2
Red	**a**	0.75	0.79	0.82	0.77	1.00	0.84
	**b**	0.79	0.76	0.10	5.78	1.00	1.00
	**c**	0.95	0.64	0.08	5.00	0.74	1.00
Green	**a**	0.61	0.71	0.85	0.60	1.00	0.82
	**b**	0.71	0.79	0.08	7.66	1.00	1.00
	**c**	0.92	0.62	0.05	7.14	0.75	1.00
Blue	**a**	0.63	0.72	0.71	0.73	1.00	0.84
	**b**	0.71	0.73	0.06	8.22	0.99	1.00
	**c**	0.92	0.63	0.06	7.06	0.75	1.00

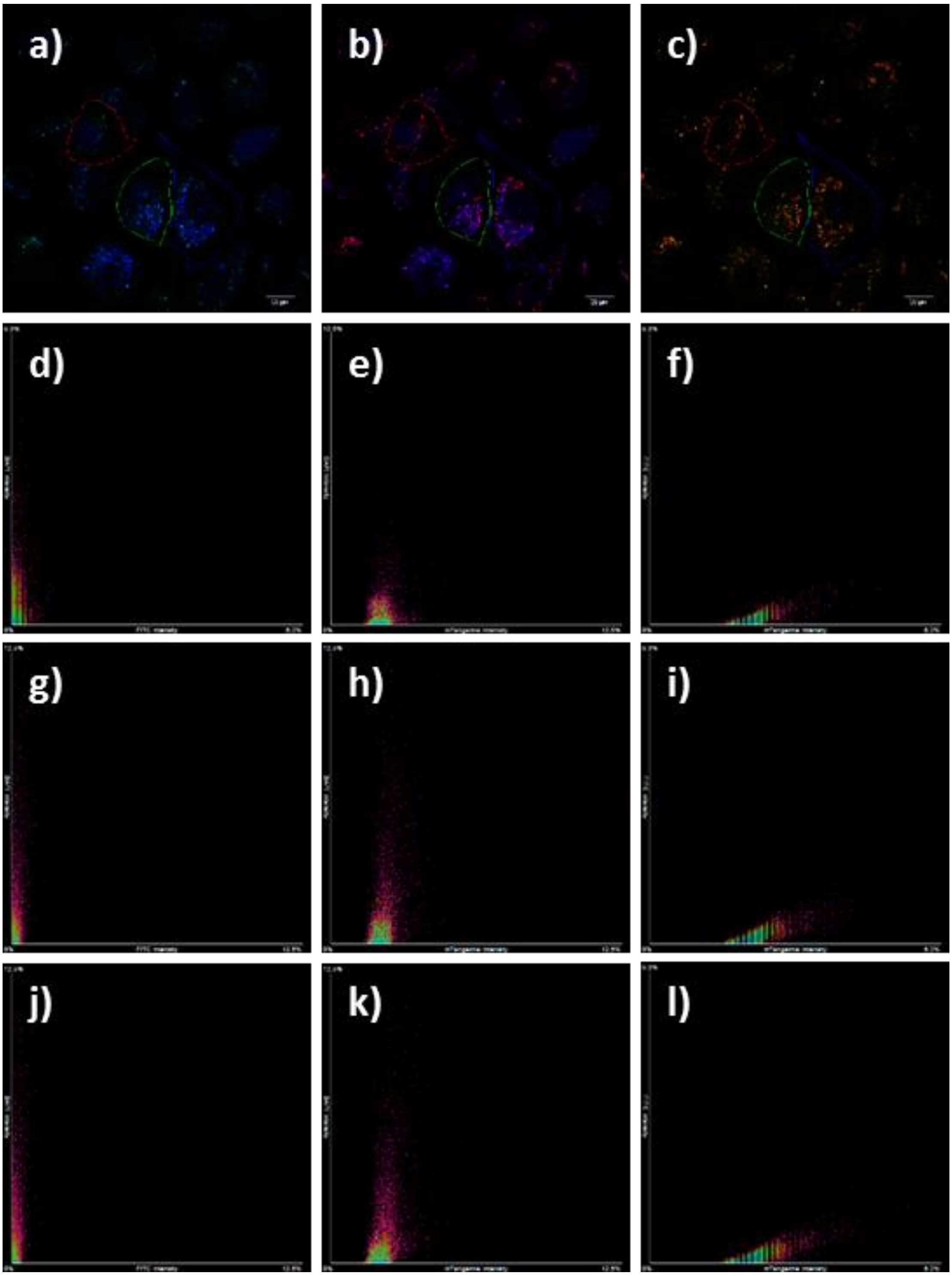

Figure S90. Confocal laser-scanning microscopy of PC-3 cells incubated at 37 °C for 18 h with a-c) ***D-3*** and Lysotracker® Blue DND-22 (100 µM and 100 nM, respectively, in 1:99 DMSO:serum-free medium). a) overlay of blue (λex=405 nm, λem=417-477 nm) and green channels (λex=488 nm, λem=500-550 nm); b) overlay of blue (λex=405 nm, λem=417-477 nm) and red channels (λex=488 nm, λem=570-750 nm); c) overlay of green (λex = 488.0 nm, λem=500-550 nm) and red channels (λex=488 nm, λem=570-750 nm). Scale bar: 20 µm. d,g,j) scatterplots of blue and green pixel intensities of the cells in the indicated red ROI (d), green ROI (g) and blue ROI (j) in the micrograph a; f,i,l) scatterplots of blue and red pixel intensities of the cells in the indicated red ROI (e), green ROI (h) and blue ROI (k) in the micrograph b; scatterplots of green and red pixel intensities of the cells in the indicated red ROI (f), green ROI (i) and blue ROI (l) in the micrograph c.

Table S8. Table of the parameters of ***D*-3** extrapolated by scatterplot analysis using the software *Nikon Elements-AR Analysis 4.30.02*.

ROI	Figure	Pearson's correlation	Mander's overlap	Mander's overlap coeff. k1	Mander's overlap coeff. k2	Correlation coeff. c1	Correlation coeff. c2
Red	**a**	0.16	0.47	1.78	0.12	0.92	0.77
	**b**	0.20	0.58	0.14	2.45	0.87	1.00
	**c**	0.80	0.73	0.05	11.90	0.75	1.00
Green	**a**	0.42	0.58	4.91	0.07	0.97	0.89
	**b**	0.43	0.49	0.34	0.69	0.94	1.00
	**c**	0.83	0.75	0.06	9.04	0.82	1.00
Blue	**a**	0.40	0.55	4.73	0.06	0.93	0.86
	**b**	0.41	0.49	0.29	0.82	0.89	1.00
	**c**	0.83	0.72	0.05	10.45	0.76	1.00

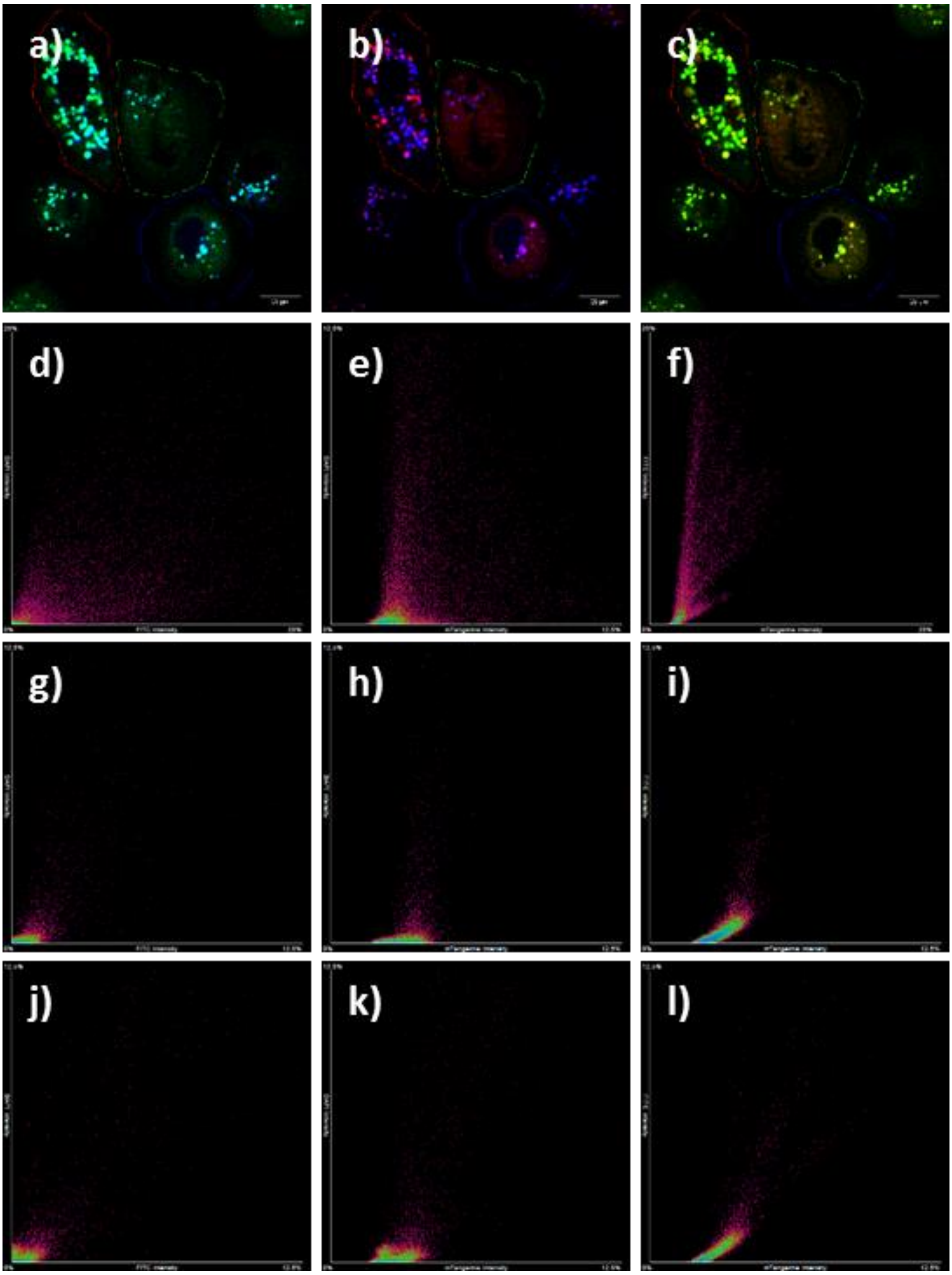

Figure S91. Confocal laser-scanning microscopy of PC-3 cells incubated at 37 °C for 18 h with a-c) ***L*-4** and Lysotracker® Blue DND-22 (100 µM and 100 nM, respectively, in 1:99 DMSO:serum-free medium). a) overlay of blue (λ_{ex}=405 nm, λ_{em}=417-477 nm) and green channels (λ_{ex}=488 nm, λ_{em}=500-550 nm); b) overlay of blue (λ_{ex}=405 nm, λ_{em}=417-477 nm) and red channels (λ_{ex}=488 nm, λ_{em}=570-750 nm); c) overlay of green (λ_{ex}=488 nm, λ_{em}=500-550 nm) and red channels (λ_{ex}=488 nm, λ_{em}=570-750 nm). Scale bar: 20 µm. d,g,j) scatterplots of blue and green pixel intensities of the cells in the indicated red ROI (d), green ROI (g) and blue ROI (j) in the micrograph a; f,i,l) scatterplots of blue and red pixel intensities of the cells in the indicated red ROI (e), green ROI (h) and blue ROI (k) in the micrograph b; scatterplots of green and red pixel intensities of the cells in the indicated red ROI (f), green ROI (i) and blue ROI (l) in the micrograph c.

Table S9. Table of the parameters of ***L*-4** extrapolated by scatterplot analysis using the software *Nikon Elements-AR Analysis 4.30.02*.

ROI	Figure	Pearson's correlation	Mander's overlap	Mander's overlap coeff. k1	Mander's overlap coeff. k2	Correlation coeff. c1	Correlation coeff. c2
Red	a	0.70	0.74	0.44	1.25	0.98	1.00
	b	0.40	0.45	0.34	0.60	0.86	1.00
	c	0.72	0.62	0.79	0.48	0.96	1.00
Green	a	0.70	0.68	0.61	0.75	0.85	0.99
	b	0.31	0.29	0.05	1.54	0.77	1.00
	c	0.73	0.73	0.15	3.50	0.98	1.00
Blue	a	0.83	0.84	0.80	0.87	0.94	0.98
	b	0.60	0.38	0.15	0.95	0.80	1.00
	c	0.70	0.51	0.21	1.22	0.93	1.00

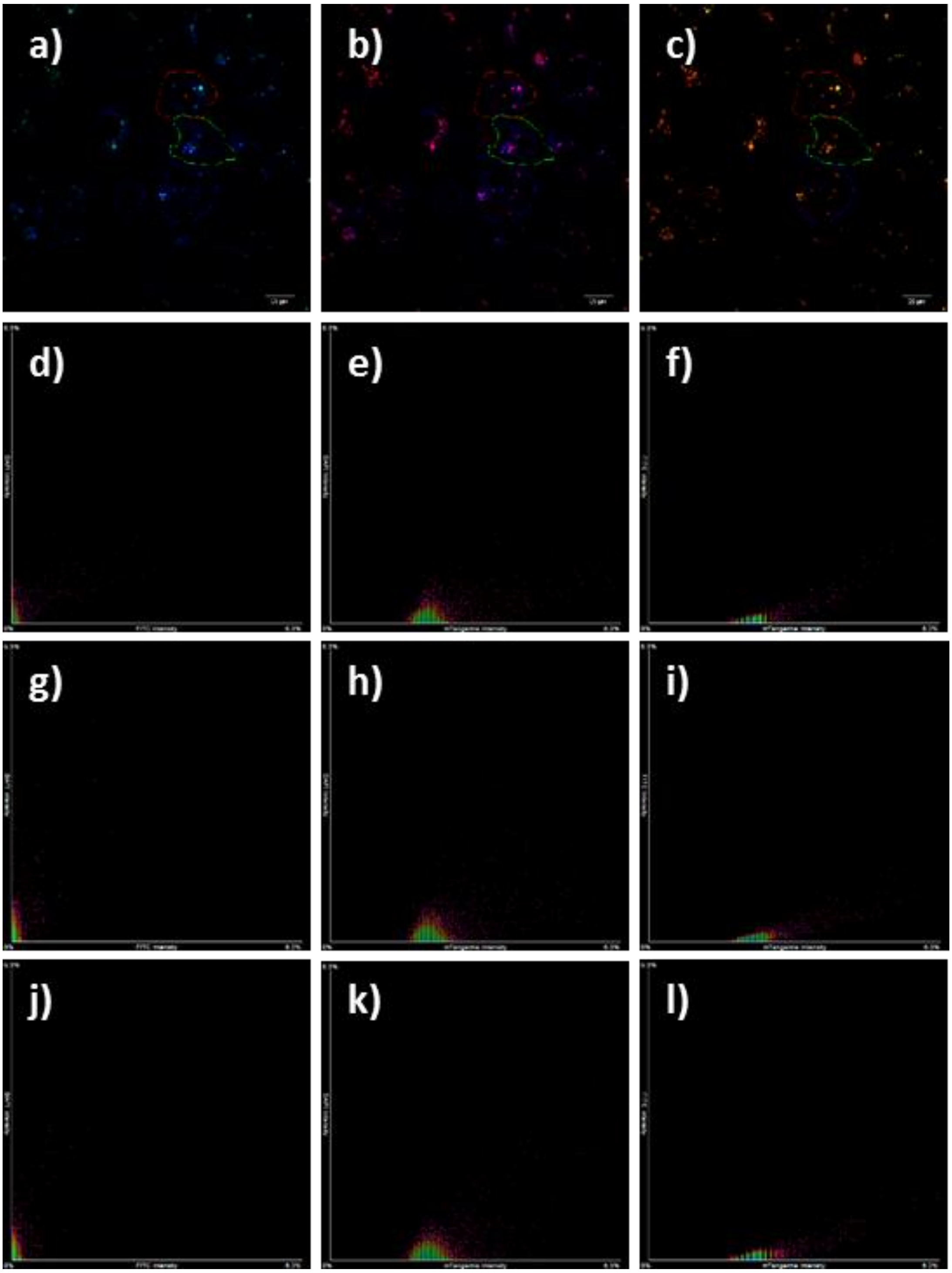

Figure S92. Confocal laser-scanning microscopy of PC-3 cells incubated at 37 °C for 18 h with a-c) ***D*-4** and Lysotracker® Blue DND-22 (100 µM and 100 nM, respectively, in 1:99 DMSO:serum-free medium). a) overlay of blue (λ_{ex}=405 nm, λ_{em}=417-477 nm) and green channels (λ_{ex}=488 nm, λ_{em}=500-550 nm); b) overlay of blue (λ_{ex}=405 nm, λ_{em}=417-477 nm) and red channels (λ_{ex}=488 nm, λ_{em}=570-750 nm); c) overlay of green (λ_{ex}=488 nm, λ_{em}=500-550 nm) and red channels (λ_{ex}=488 nm, λ_{em}=570-750 nm). Scale bar: 20 µm. d,g,j) scatterplots of blue and green pixel intensities of the cells in the indicated red ROI (d), green ROI (g) and blue ROI (j) in the micrograph a; f,i,l) scatterplots of blue and red pixel intensities of the cells in the indicated red ROI (e), green ROI (h) and blue ROI (k) in the micrograph b; scatterplots of green and red pixel intensities of the cells in the indicated red ROI (f), green ROI (i) and blue ROI (l) in the micrograph c.

Table S10. Table of the parameters of ***D*-4** extrapolated by scatterplot analysis using the software *Nikon Elements-AR Analysis 4.30.02*.

ROI	Figure	Pearson's correlation	Mander's overlap	Mander's overlap coeff. k1	Mander's overlap coeff. k2	Correlation coeff. c1	Correlation coeff. c2
Red	a	0.55	0.59	0.62	0.57	0.72	0.56
	b	0.49	0.59	0.05	6.79	0.58	1.00
	c	0.89	0.43	0.04	5.22	0.53	1.00
Green	a	0.56	0.63	1.88	0.21	0.72	0.65
	b	0.39	0.45	0.07	2.93	0.65	1.00
	c	0.83	0.53	0.03	10.29	0.59	1.00
Blue	a	0.51	0.62	1.46	0.26	0.61	0.58
	b	0.43	0.51	0.05	5.00	0.59	1.00
	c	0.81	0.55	0.02	12.50	0.56	1.00

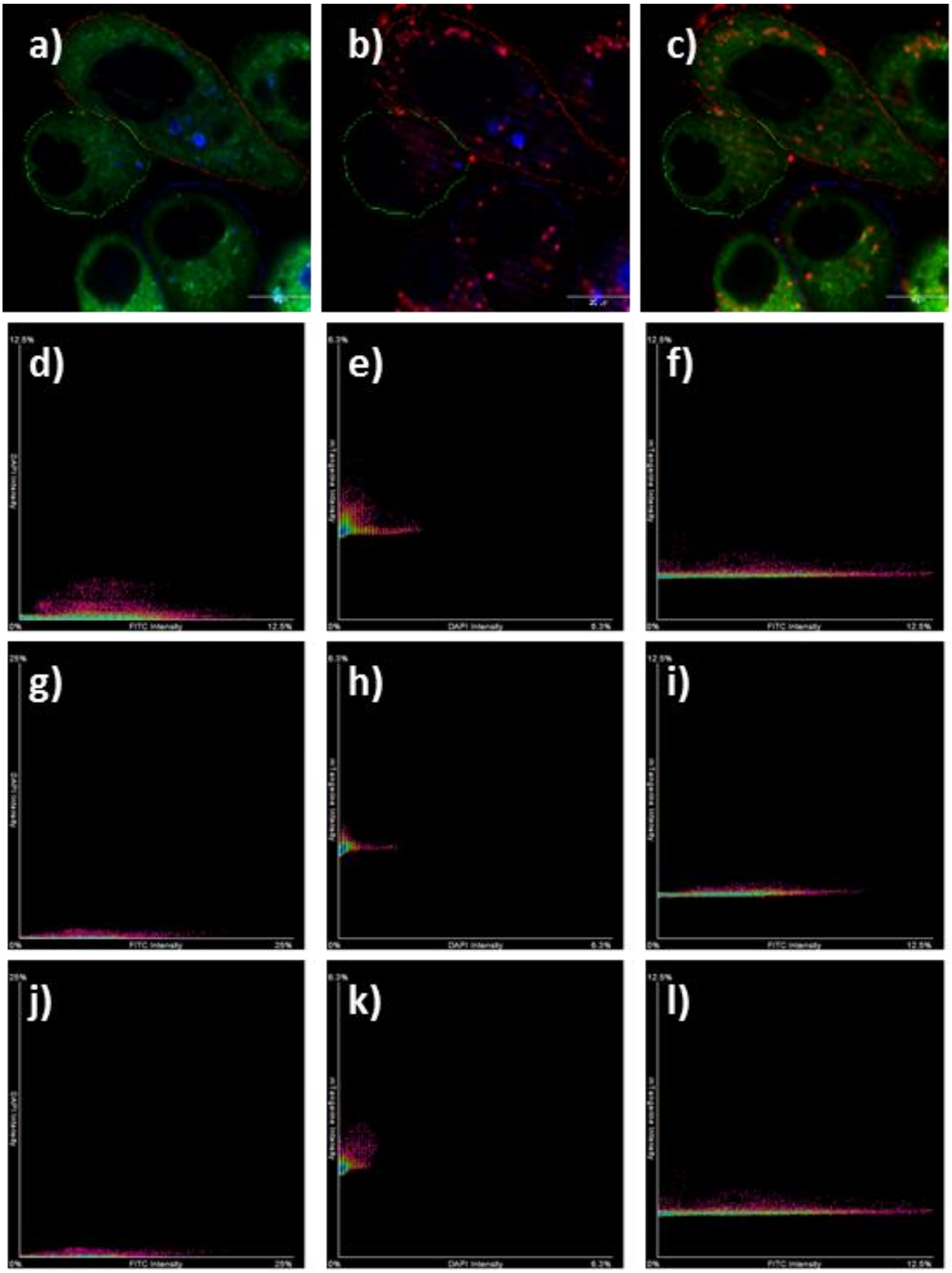

Figure S93. Confocal laser-scanning microscopy of PC-3 cells incubated at 37 °C for 18 h with a-c) ***L*-6** and Lysotracker® Blue DND-22 (100 µM and 100 nM, respectively, in 1:99 DMSO:serum-free medium). a) overlay of blue (λ_{ex}=405 nm, λ_{em}=417-477 nm) and green channels (λ_{ex}=488 nm, λ_{em}=500-550 nm); b) overlay of blue (λ_{ex}=405 nm, λ_{em}=417-477 nm) and red channels (λ_{ex}=561 nm, λ_{em}=570750 nm); c) overlay of green (λ_{ex}=488 nm, λ_{em}=500-550 nm) and red channels (λ_{ex}=561 nm, λ_{em}=570-750 nm). Scale bar: 20 µm. d,g,j) scatterplots of blue and green pixel intensities of the cells in the indicated red ROI (d), green ROI (g) and blue ROI (j) in the micrograph a; f,i,l) scatterplots of blue and red pixel intensities of the cells in the indicated red ROI (e), green ROI (h) and blue ROI (k) in the micrograph b; scatterplots of green and red pixel intensities of the cells in the indicated red ROI (f), green ROI (i) and blue ROI (l) in the micrograph c.

Table S11. Table of the parameters of ***L*-6** extrapolated by scatterplot analysis using the software *Nikon Elements-AR Analysis 4.30.02*.

ROI	Figure	Pearson's correlation	Mander's overlap	Mander's overlap coeff. k1	Mander's overlap coeff. k2	Correlation coeff. c1	Correlation coeff. c2
Red	a	0.24	0.68	0.04	11.45	1.00	0.98
	b	0.25	0.71	8.13	0.06	1.00	1.00
	c	0.40	0.83	0.56	1.23	1.00	0.98
Green	a	0.43	0.79	0.03	19.29	1.00	0.99
	b	0.42	0.79	13.63	0.05	1.00	1.00
	c	0.54	0.80	0.57	1.15	1.00	0.98
Blue	a	0.51	0.79	0.03	24.72	1.00	0.99
	b	0.32	0.58	0.67	0.51	0.97	0.99
	c	0.35	0.74	0.43	1.27	1.00	0.99

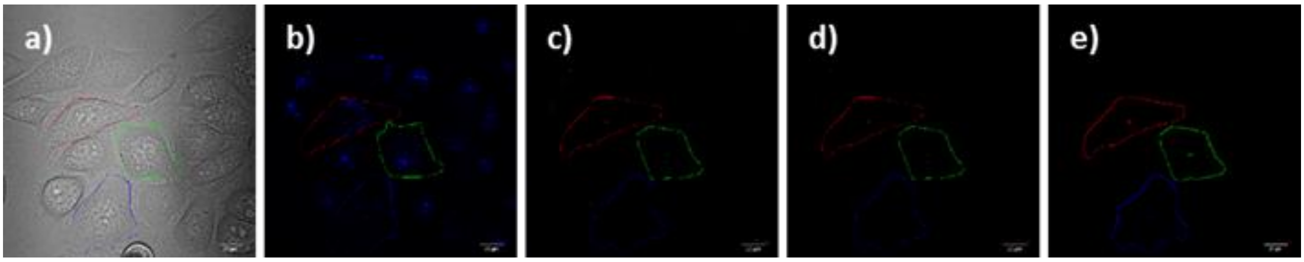

Figure S94. Control: confocal laser-scanning microscopy of PC-3 cells incubated at 37 °C for 18 h with a-e) Lysotracker® Blue DND-22 (100 nM , in 1:99 DMSO:serum-free medium). a) bright field channel; b) blue channel (λ_{ex}=405 nm, λ_{em}=417-477 nm); c) green channel (λ_{ex}=488 nm, λ_{em}=500-550 nm); d) red channel (λ_{ex}=488 nm, λ_{em}=570-750 nm); e) overlay of the blu-green and red channels. Scale bar: 20 µm.

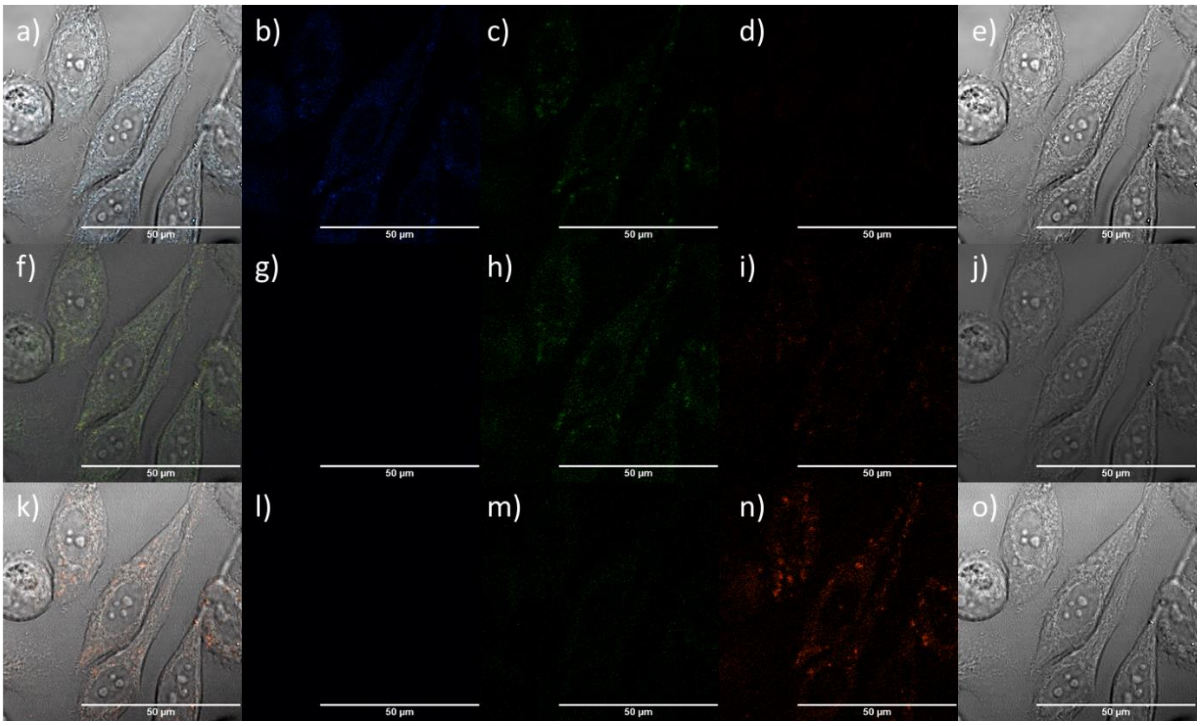

Figure S95. Confocal laser-scanning microscopy of PC-3 cells incubated at 37 °C for 60 min with a-o) ***L*-3** (100 µM in 1:99 DMSO:serum-free medium). a,f,k) bright field channel; b,g,l) blue channel (λ_{em}=417-477 nm); c,h,m) green channel (λ_{em}=500-550 nm); d,i,n) red channel (λ_{em}=570-750 nm); e,j,o) overlay of the blue-green-red channels. a-e) λ_{ex}=405 nm; f-j) λ_{ex}=488 nm; k-o) λ_{ex}=561 nm. Scale bar: 50 µm.

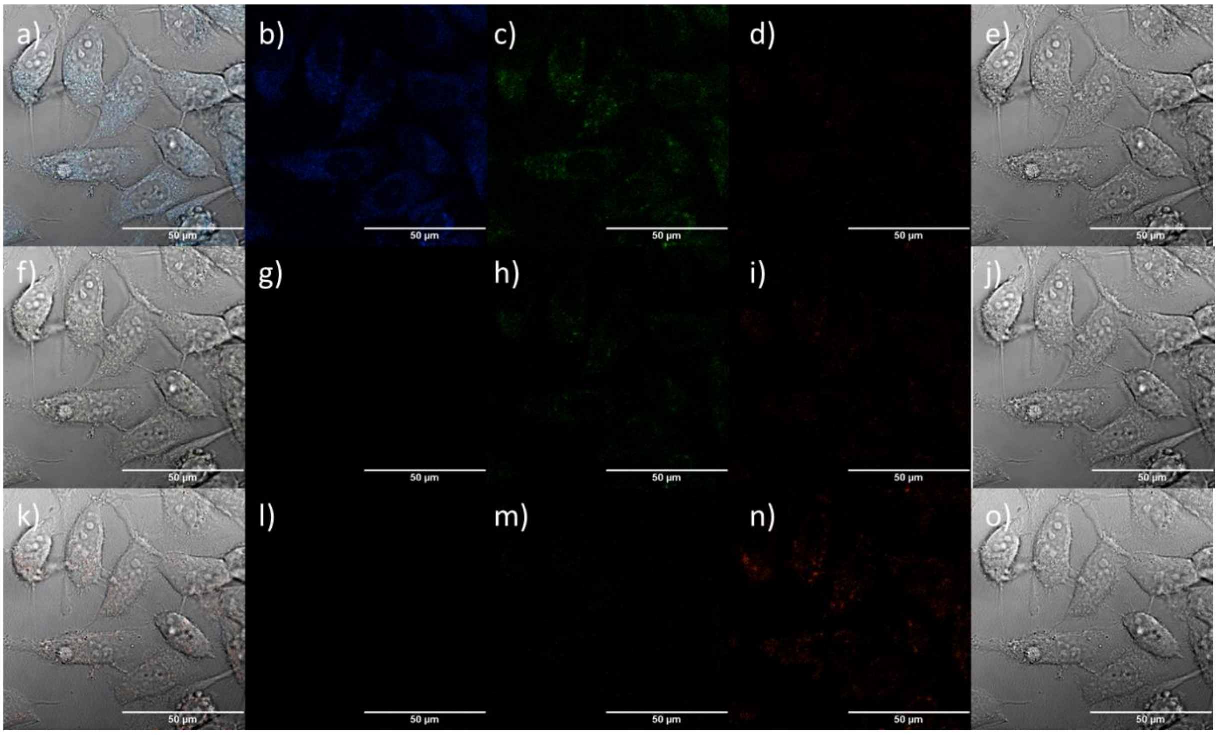

Figure S96. Confocal laser-scanning microscopy of PC-3 cells incubated at 37 °C for 20 min with a-o) ***L*-4** (100 µM in 1:99 DMSO:serum-free medium). a,f,k) bright field channel; b,g,l) blue channel (λ_{em}=417-477 nm); c,h,m) green channel (λ_{em}=500-550 nm); d,i,n) red channel (λ_{em}=570-750 nm); e,j,o) overlay of the blue-green-red channels. a-e) λ_{ex}=405 nm; f-j) λ_{ex}=488 nm; k-o) λ_{ex}=561 nm. Scale bar: 50 µm.

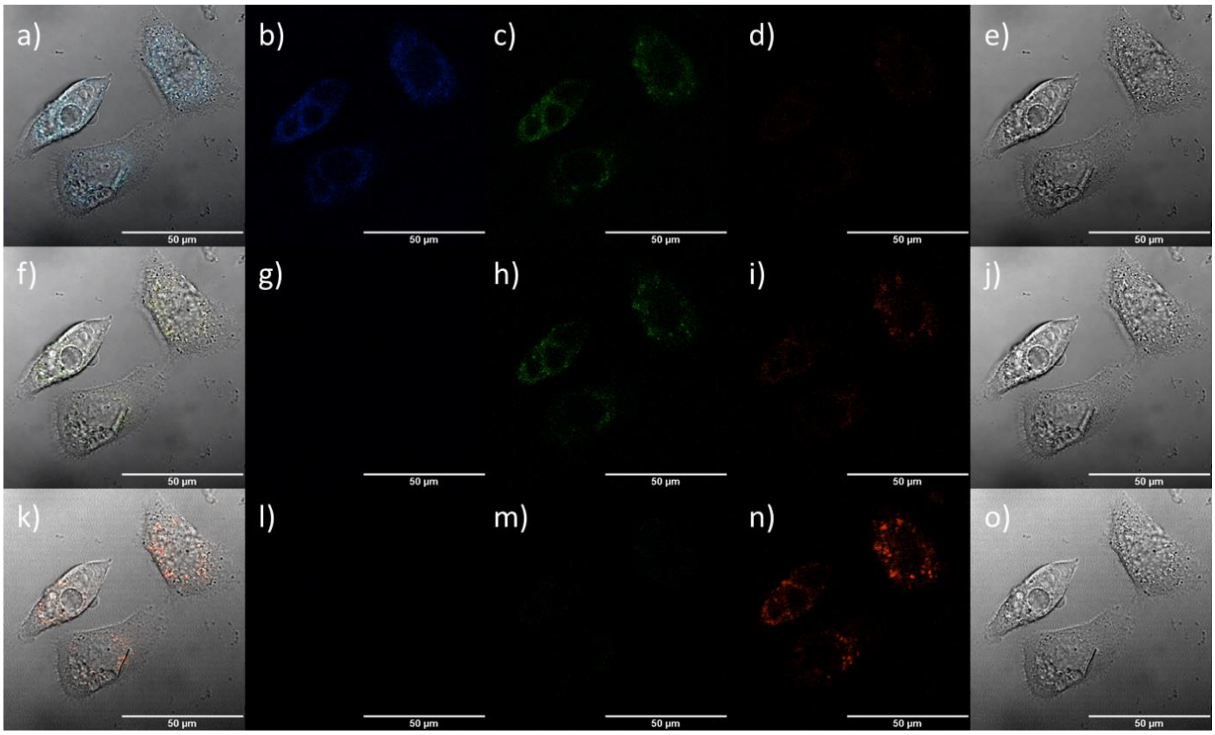

Figure S97. Confocal laser-scanning microscopy of PC-3 cells incubated at 37 °C for 18h min with a-o) ***L*-4** (100 µM in 1:99 DMSO:serum-free medium). a,f,k) bright field channel; b,g,l) blue channel (λ_{em}=417-477 nm); c,h,m) green channel (λ_{em}=500-550 nm); d,i,n) red channel (λ_{em}=570-750 nm); e,j,o) overlay of the blue-green-red channels. a-e) λ_{ex}=405 nm; f-j) λ_{ex}=488 nm; k-o) λ_{ex}=561 nm. Scale bar: 50 µm.

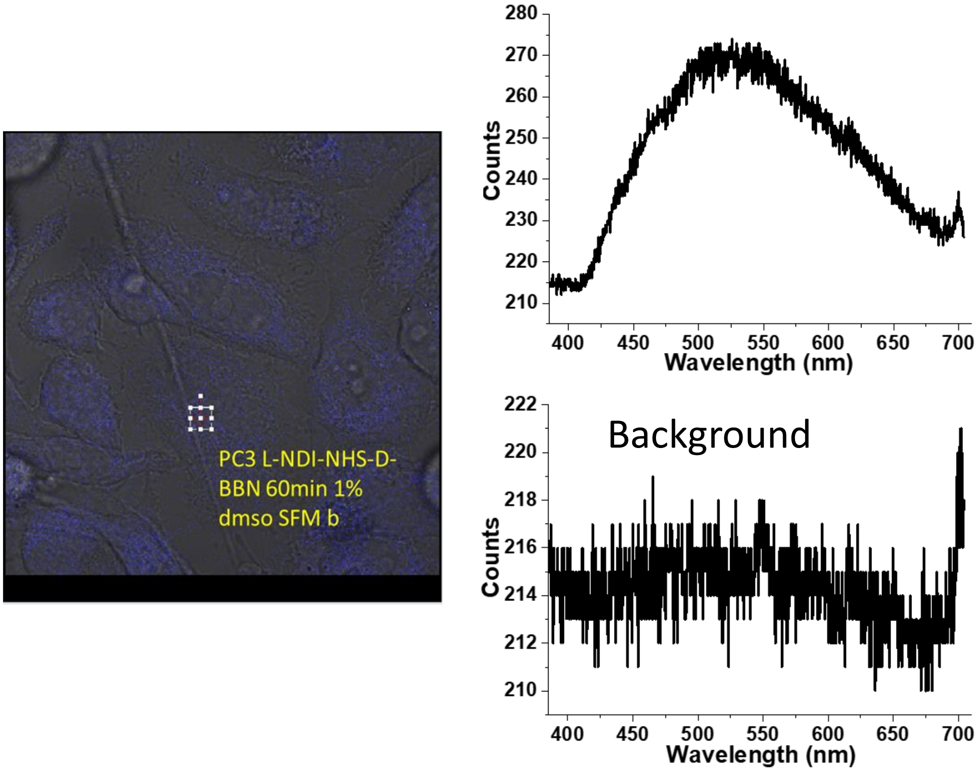

Figure S98. 2 Photon fluorescence spectroscopy in PC-3 cells incubated at 37 °C for 60 min with the **D-L** (L-NDI-D-Bombesin) mixed mono-substituted compound (100 µM in 1:99 DMSO:serum-free medium)

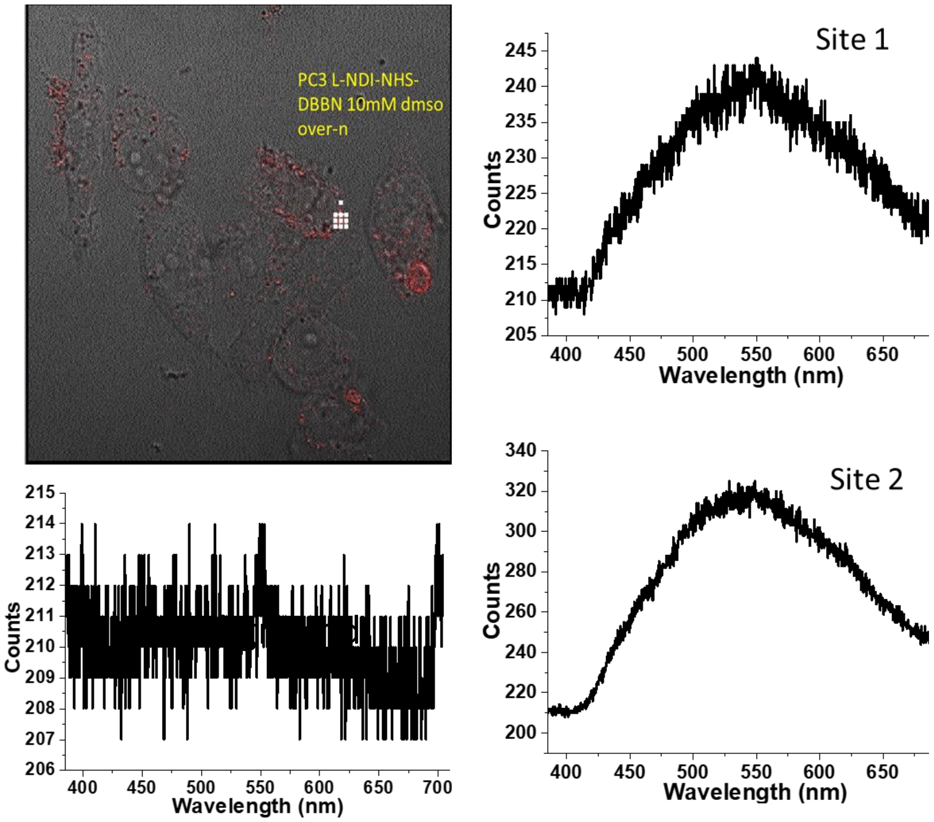

Figure S99. 2 Photon fluorescence spectroscopy in PC-3 cells incubated at 37 °C for 18 h with ***D-L*** **(**L-NDI-D-Bombesin) mono-substituted conjugate (100 µM and 1:99 DMSO:serum-free medium resulting from 10 mM DMSO stock treatment on cell plate)

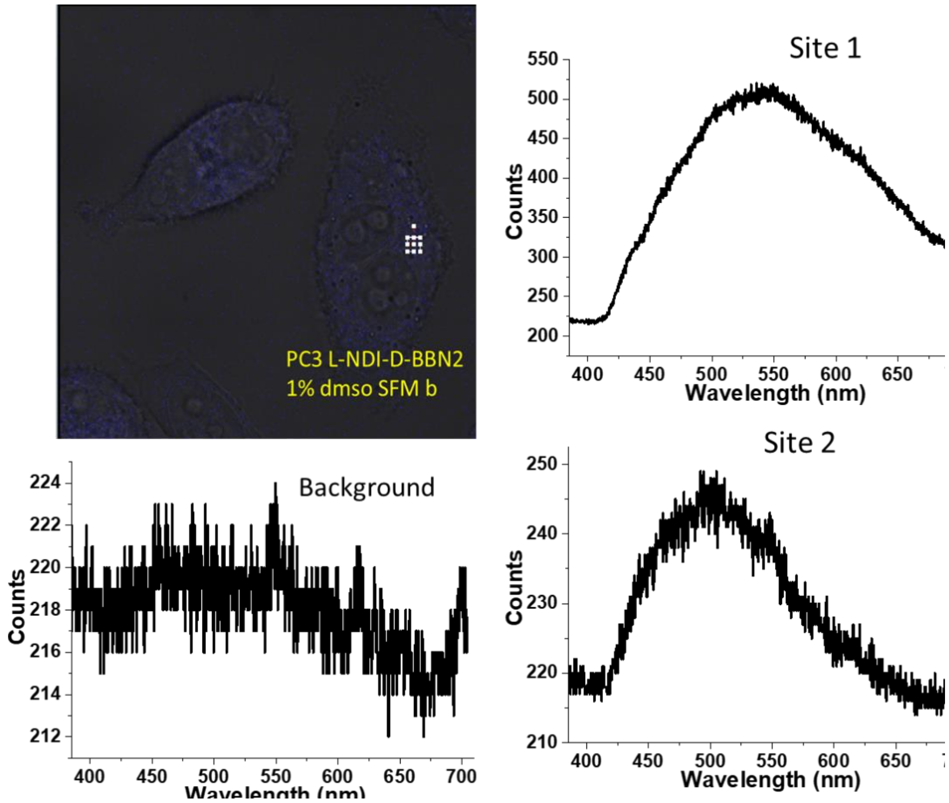

Figure S100. 2 Photon fluorescence spectroscopy in PC-3 cells incubated at 37 °C for 18h with ***D-L*** (L-NDI-D-Bombesin2) bis-substituted compound (1:99 DMSO:serum-free medium final treatment on cell plates)

Cytotoxicity assays in PC-3 cells

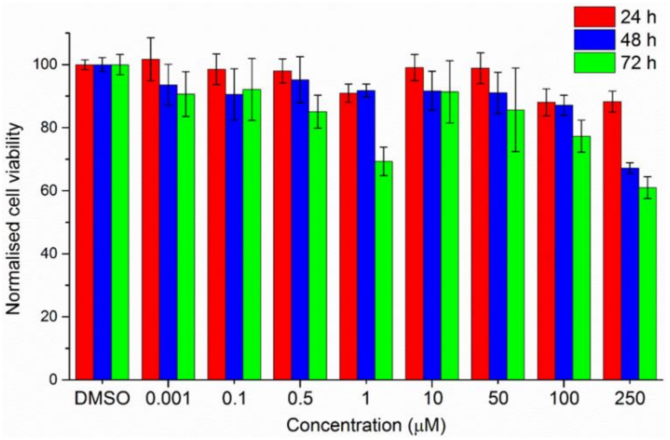

(a)

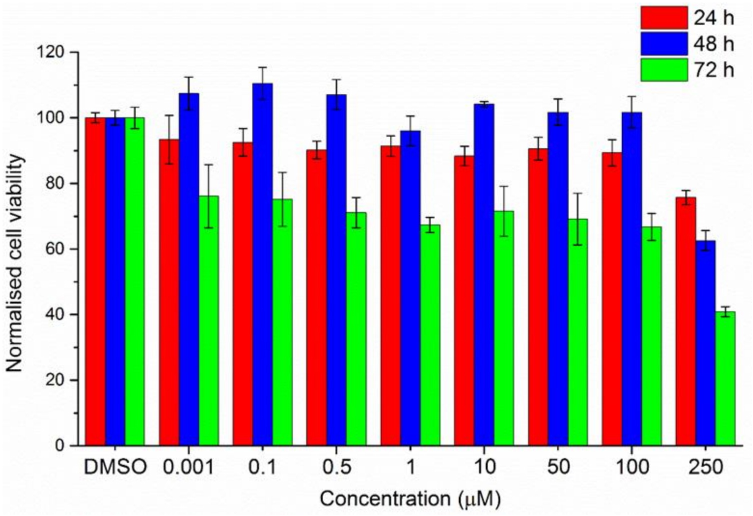

(b)

Figure S101. (a) Crystal violet assays for compound ***L*-3** to evaluate its cytotoxicity over 24 (red), 48 (blue) and 72 h (green) compared to DMSO control. Data indicated 48 h IC_{50} >100 microM. (b) Crystal violet assays for compound ***L*-4** to evaluate its cytotoxicity over 24 (red), 48 (blue) and 72 h (green) compared to DMSO control (48 h IC_{50} >100 microM).

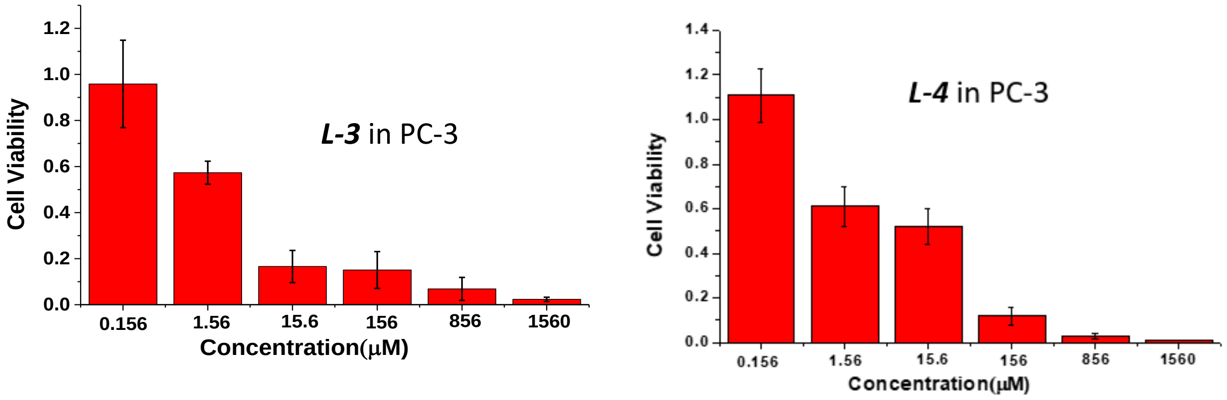

Figure S102. MTT assays for estimating cytotoxicity over 72 h at high concentration ranges.

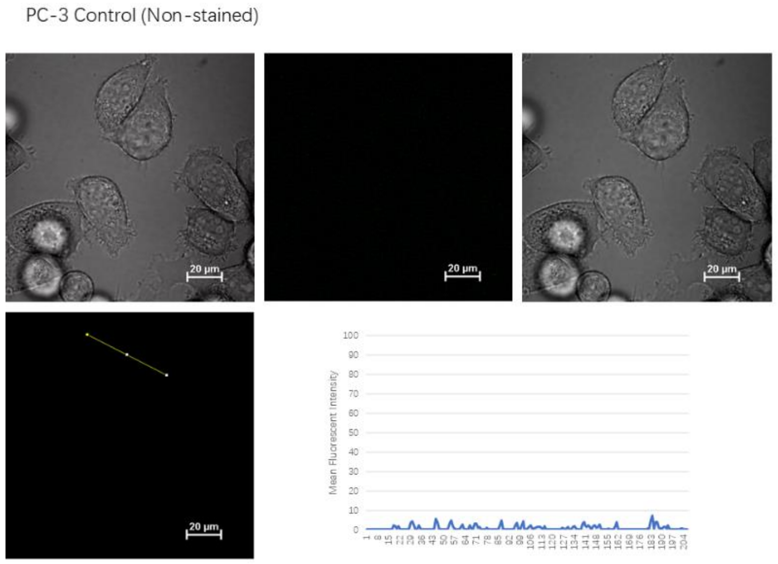
PC-3 Control (Non-stained)
20 µm
Mean Fluorescent Intensity

(A)

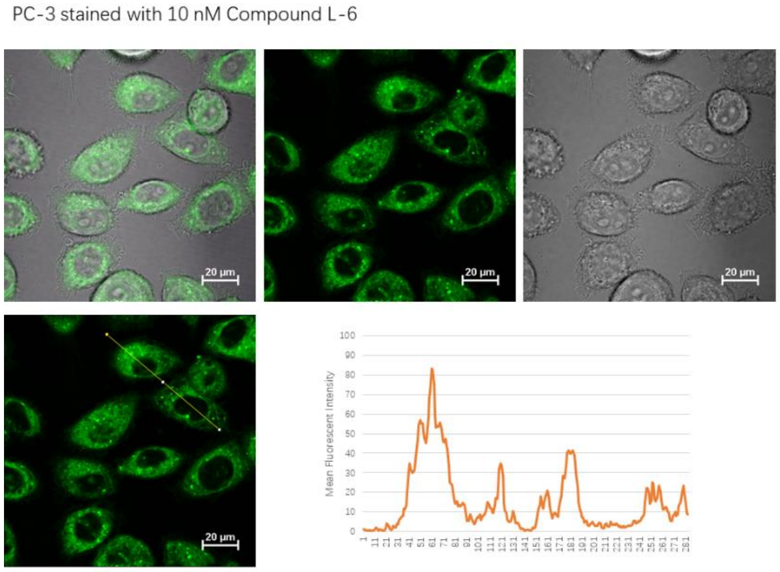
PC-3 stained with 10 nM Compound L-6
20 µm
Mean Fluorescent Intensity

(B)

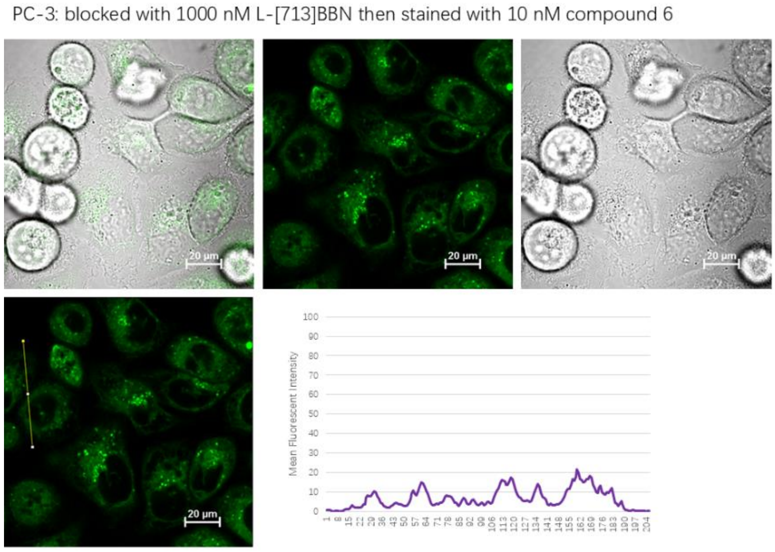
PC-3: blocked with 1000 nM L-[713]BBN then stained with 10 nM compound 6
20 µm
Mean Fluorescent Intensity

(C)

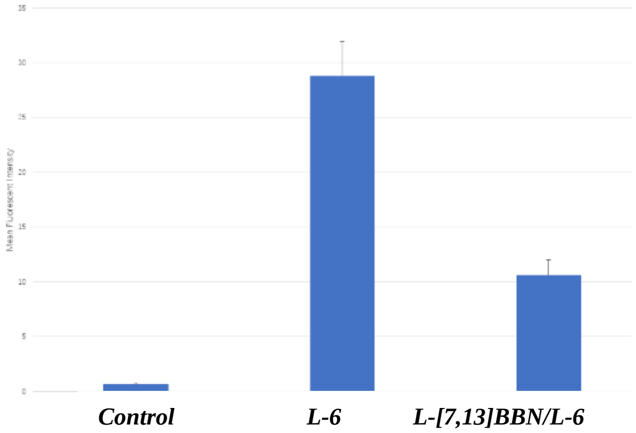

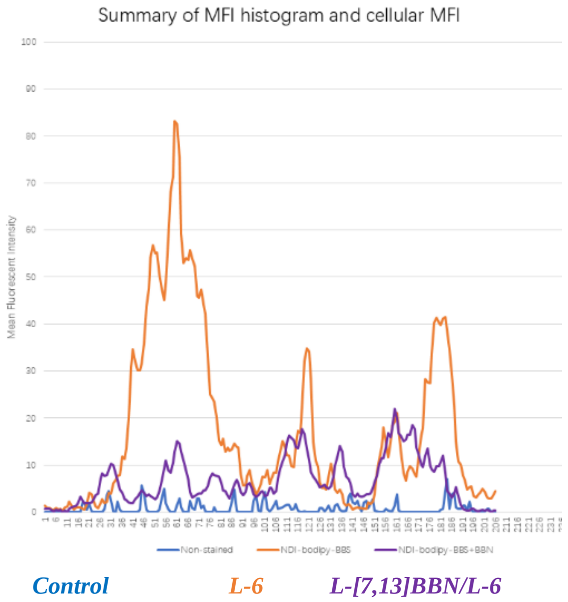

(D)

Figure S103. Micrographs depicting the confocal imaging experiments of a standard blocking assays in living PC-3 cells, (1:99 DMSO:DMEM), 20 min incubation, 37 ^{o}C, ex 488 nm: **(A)-(C),** top rows, from left to right: DIC, green channel emission, overlay with corresponding mean intensity measurements given below, in each case. **(A)**: non-treated, control PC-3 cells, 1% DMSO; **(B):** PC-3 cells, stained with 10 nM copound L-6 in 1% DMSO; **(C):** PC-3 cells, stained with excess L-[7,13]BBN, incubated for 20 min, followed by co-staining with 10 nM compound ***L-6*** (total 1%DMSO). **(D)** A comparison of the mean fluorescence intensity estimated from confocal imaging experiments of the standard blocking assays in living PC-3 cells (Figure S103), under 20 min incubation, 37 ^{o}C, ex 488 nm.

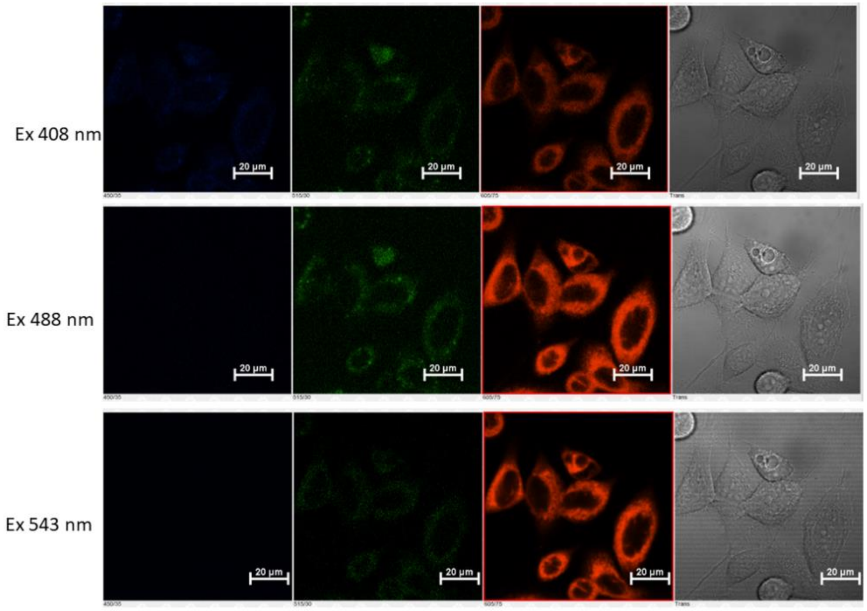

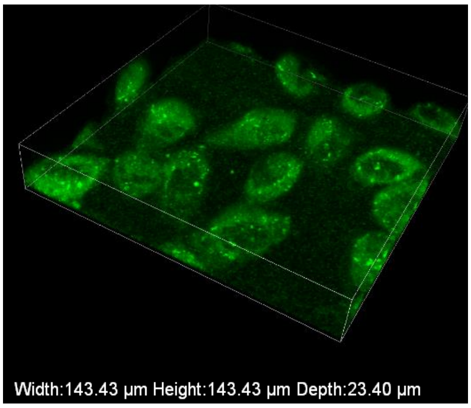

Figure S103. Micrographs depicting the confocal imaging experiments of Endoplasmic Reticulum staining experiment in living PC-3 cells (with ER Tracker Red), and a comparison with the 3D representation (Z-stacking) of copound L-6.

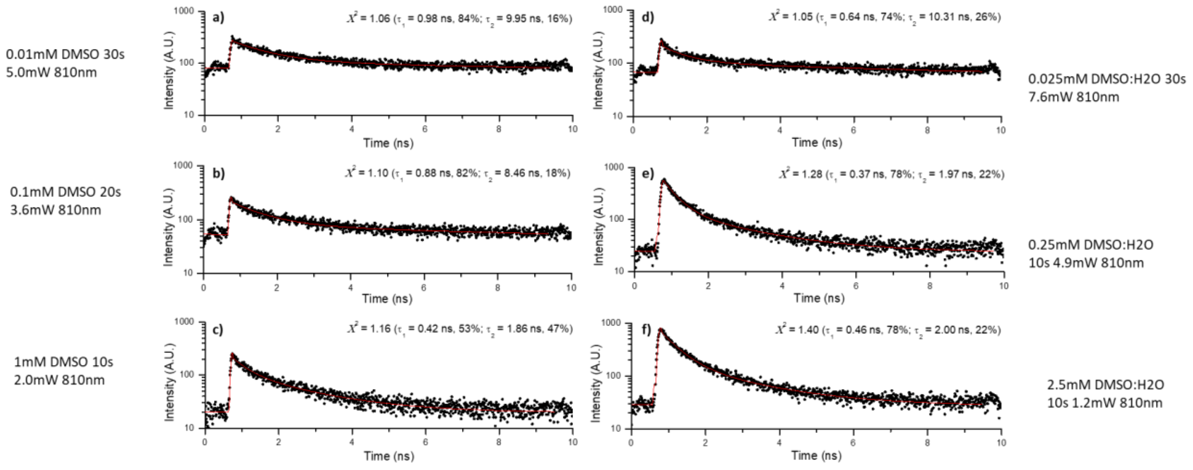

Figure S105. TCSPC, two-photon excitation, fluorescence lifetime decays for L-NDI-OSu (***L*-1**)

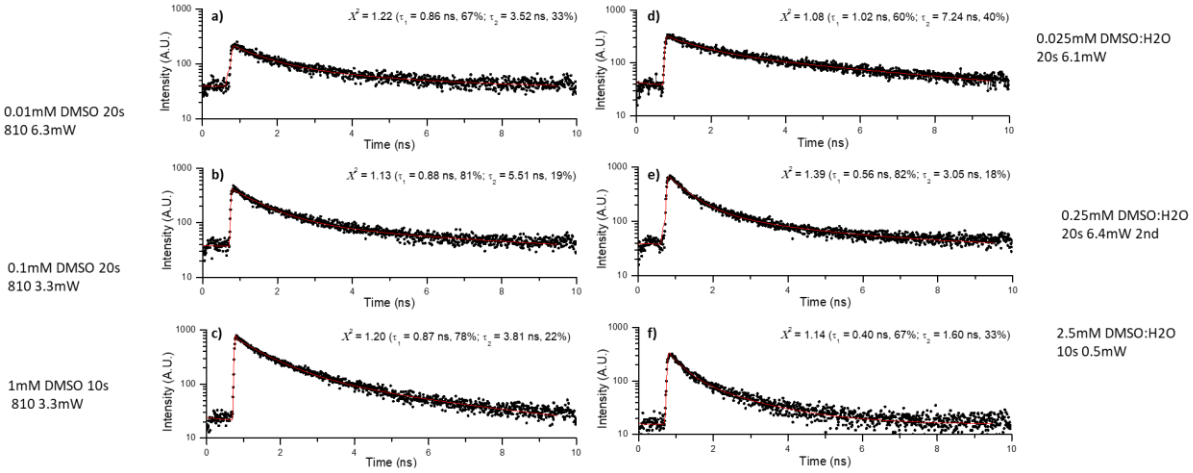

Figure S106. TCSPC, two-photon excitation, fluorescence lifetime decays for D-NDI-OSu (***D*-1**)

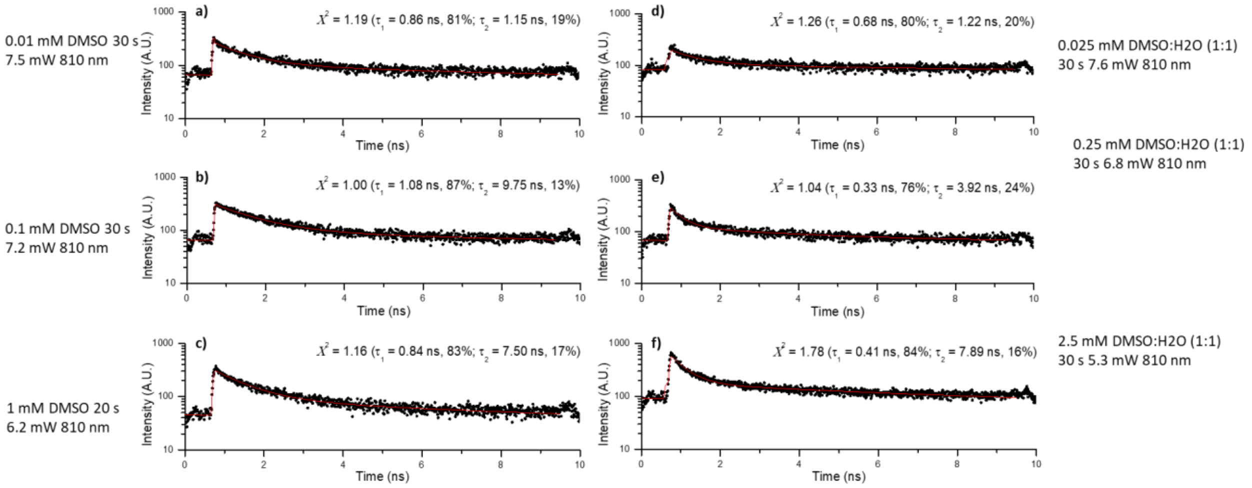

Figure S107. TCSPC, two-photon excitation, fluorescence lifetime decays for L-[7,13]BBN (***L*-2**)

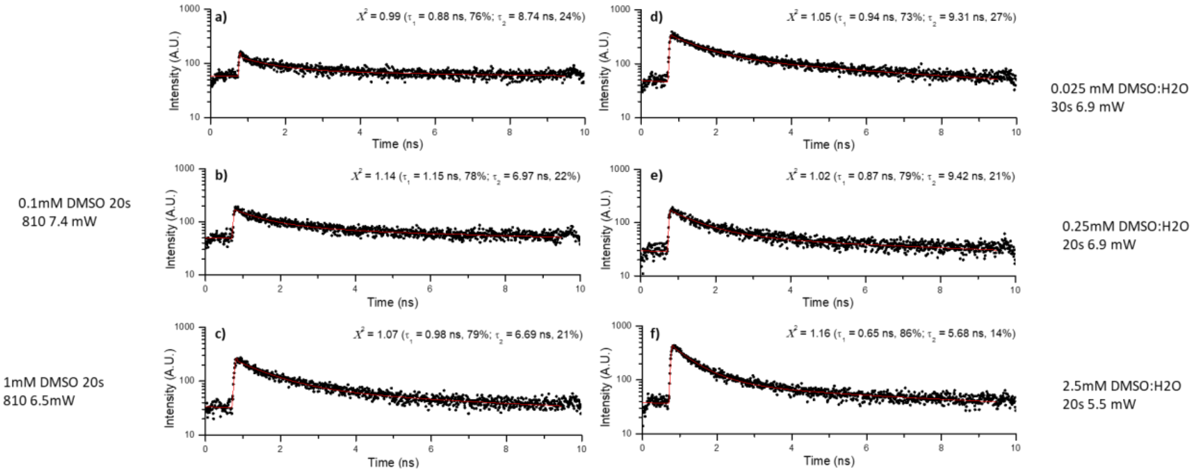

Figure 108. TCSPC, two-photon excitation, fluorescence lifetime decays for D-[7,13]BBN (***D*-2**)

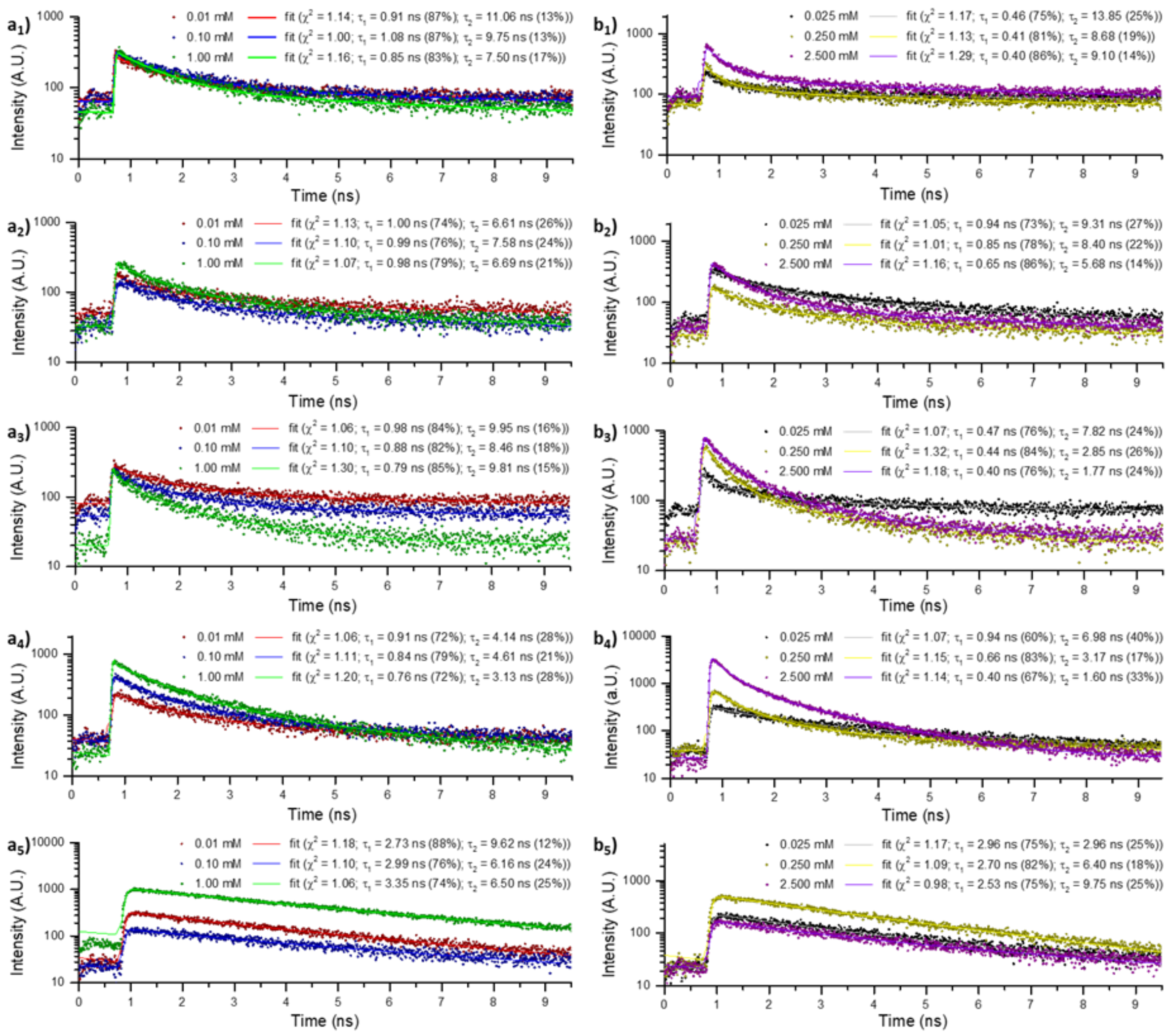

Figure S109. Two-photon time-correlated single photon counting (λ_{ex}=810 nm from a_1-b_1 to a_4-b_4 and λ_{ex}=910 nm for a_5-b_5). a_1-b_1) ***L*-2**; a_2-b_2) ***D*-2**; a_3-b_3) ***L*-1**; a_4-b_4) ***D*-1**; a_5-d_5) **5**. a_{1-5} in DMSO; b_{1-5}) in DMSO:H_2O (1:1).

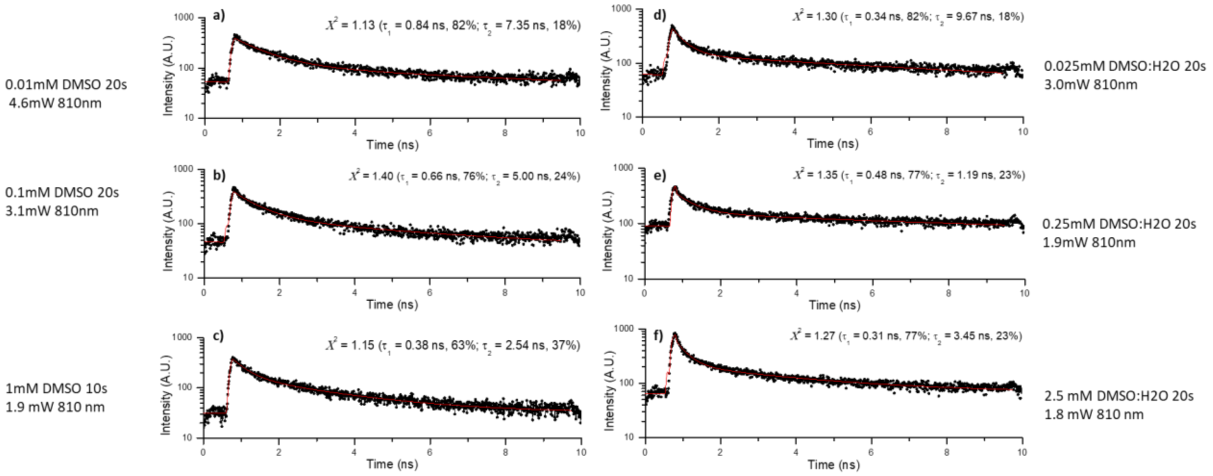

Figure S110. TCSPC, two-photon excitation, fluorescence lifetime decays for compound ***L*-3**

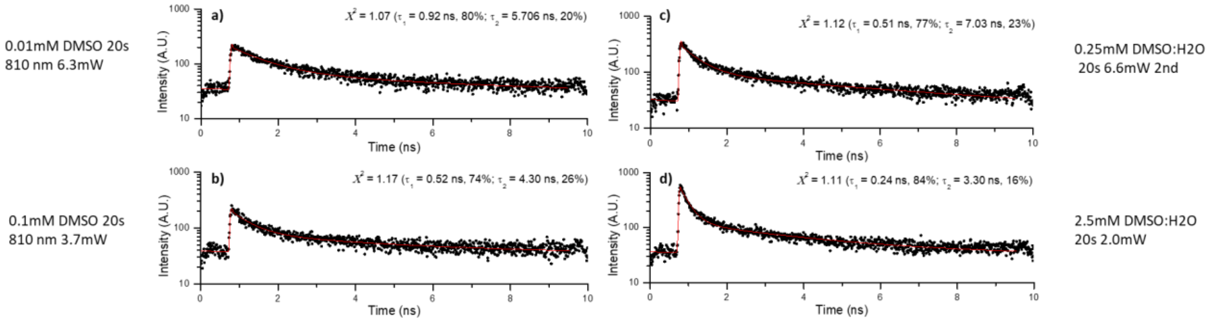

Figure S111. TCSPC, two-photon excitation, fluorescence lifetime decays for compound ***D*-3**

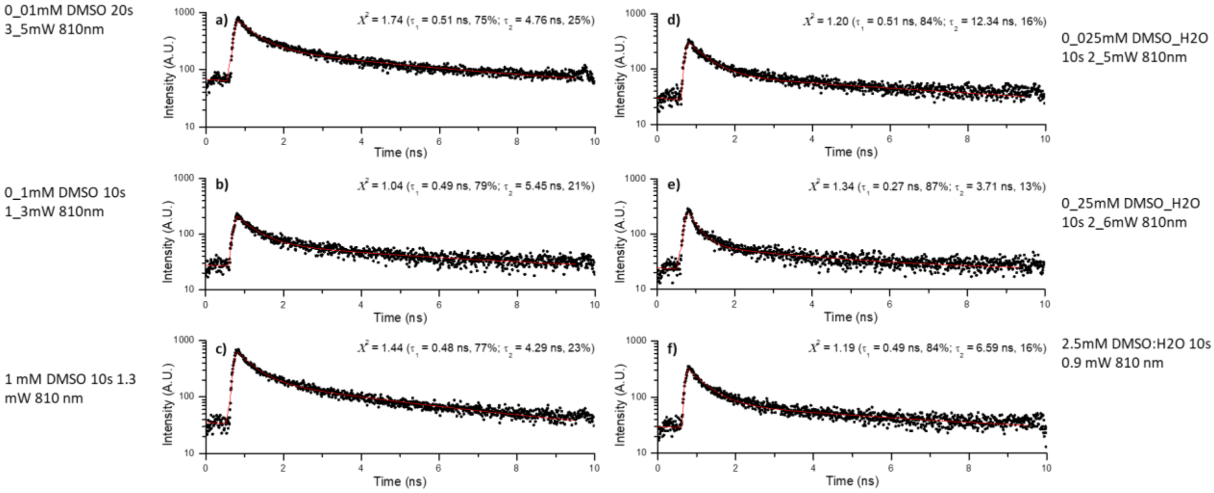

Figure S112. TCSPC, two-photon excitation, fluorescence lifetime decays for compound ***D*-4**

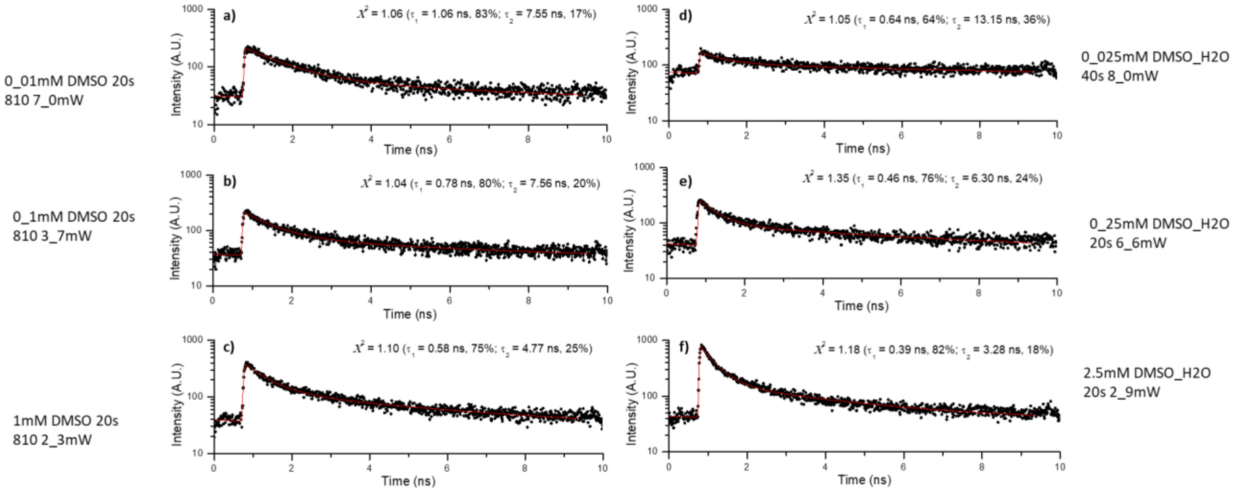

Figure S113. TCSPC, two-photon excitation, fluorescence lifetime decays for compound ***L*-4**

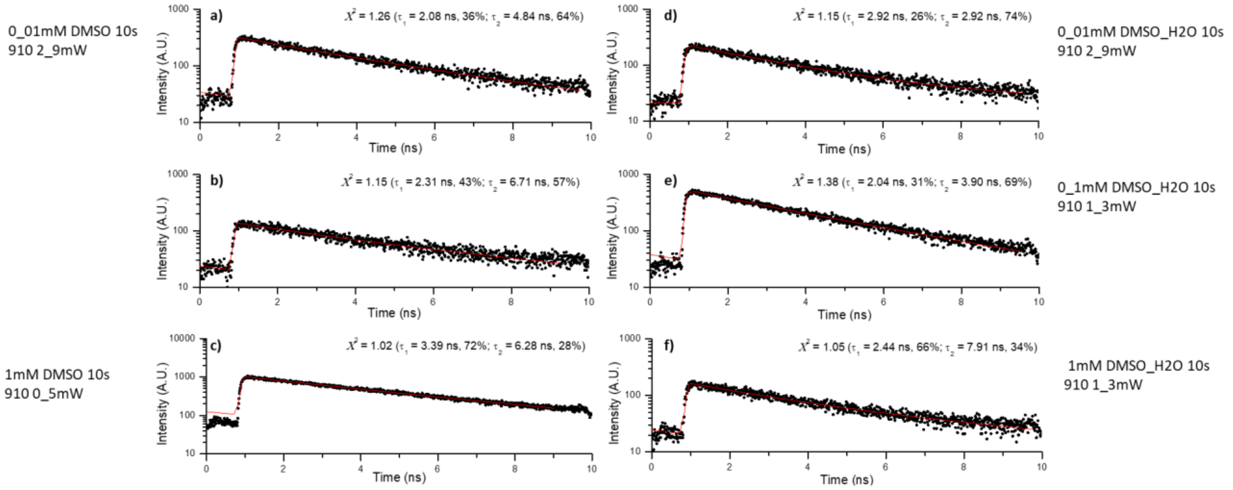

Figure S114. TCSPC, two-photon excitation, fluorescence lifetime decays for compound **5** (Bodipy-ethylenediimine)

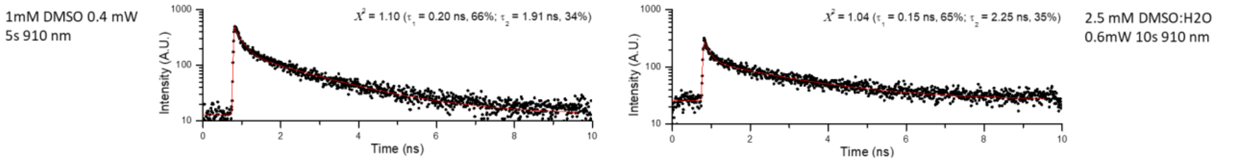

Figure S115. TCSPC, two-photon excitation, fluorescence lifetime decays for compound ***L*-6**

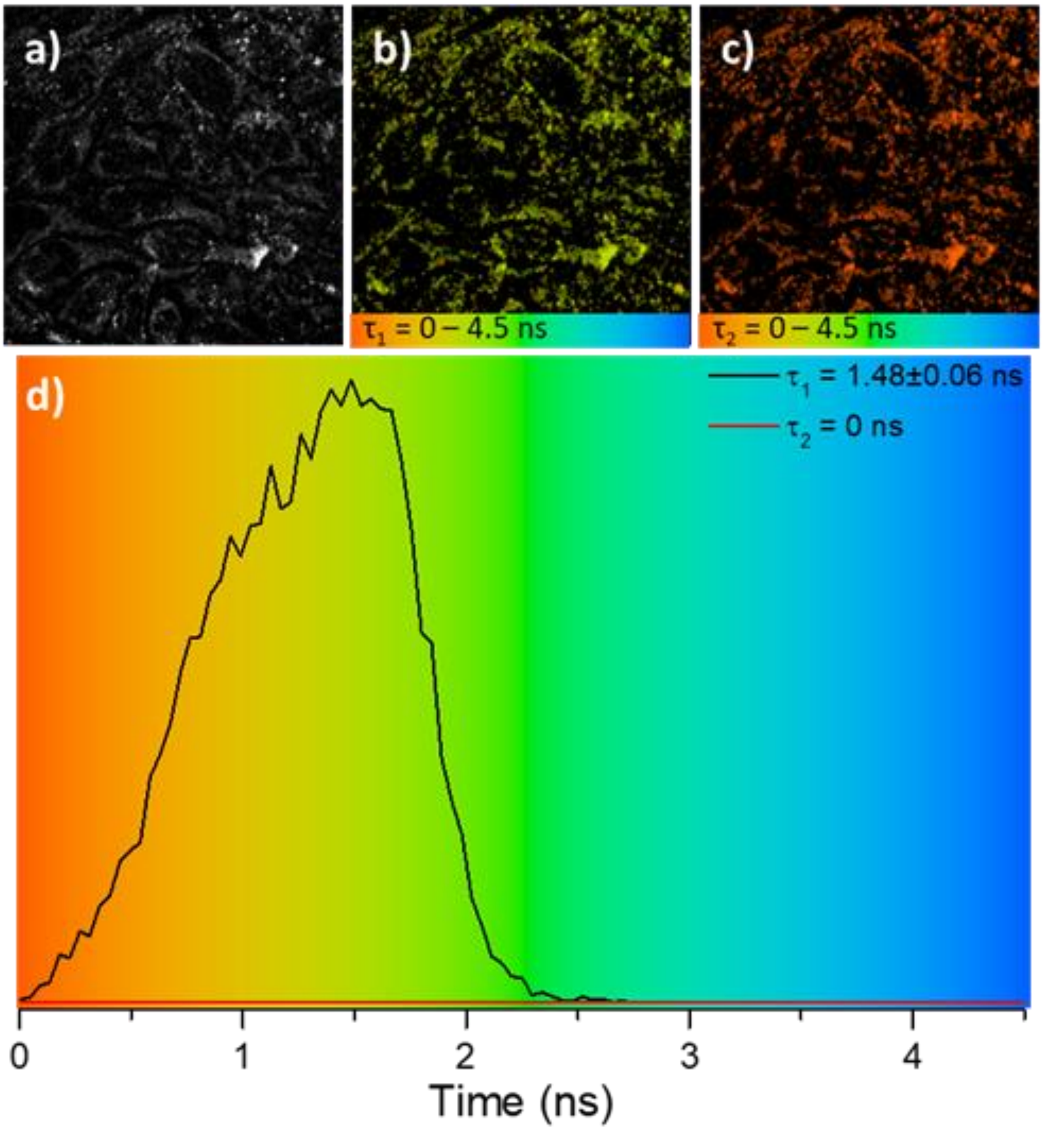

Figure S116. Two-photon fluorescence lifetime imaging in A431 cells line incubated with 1% DMSO (1:99 DMSO:serum-free medium) including a) intensity map, b) and c) lifetime maps in coloured code and d) lifetime distribution. Field of view was 100 µm, λ_{ex}=810 nm; laser power 6. mW, acquisition time 20 s.

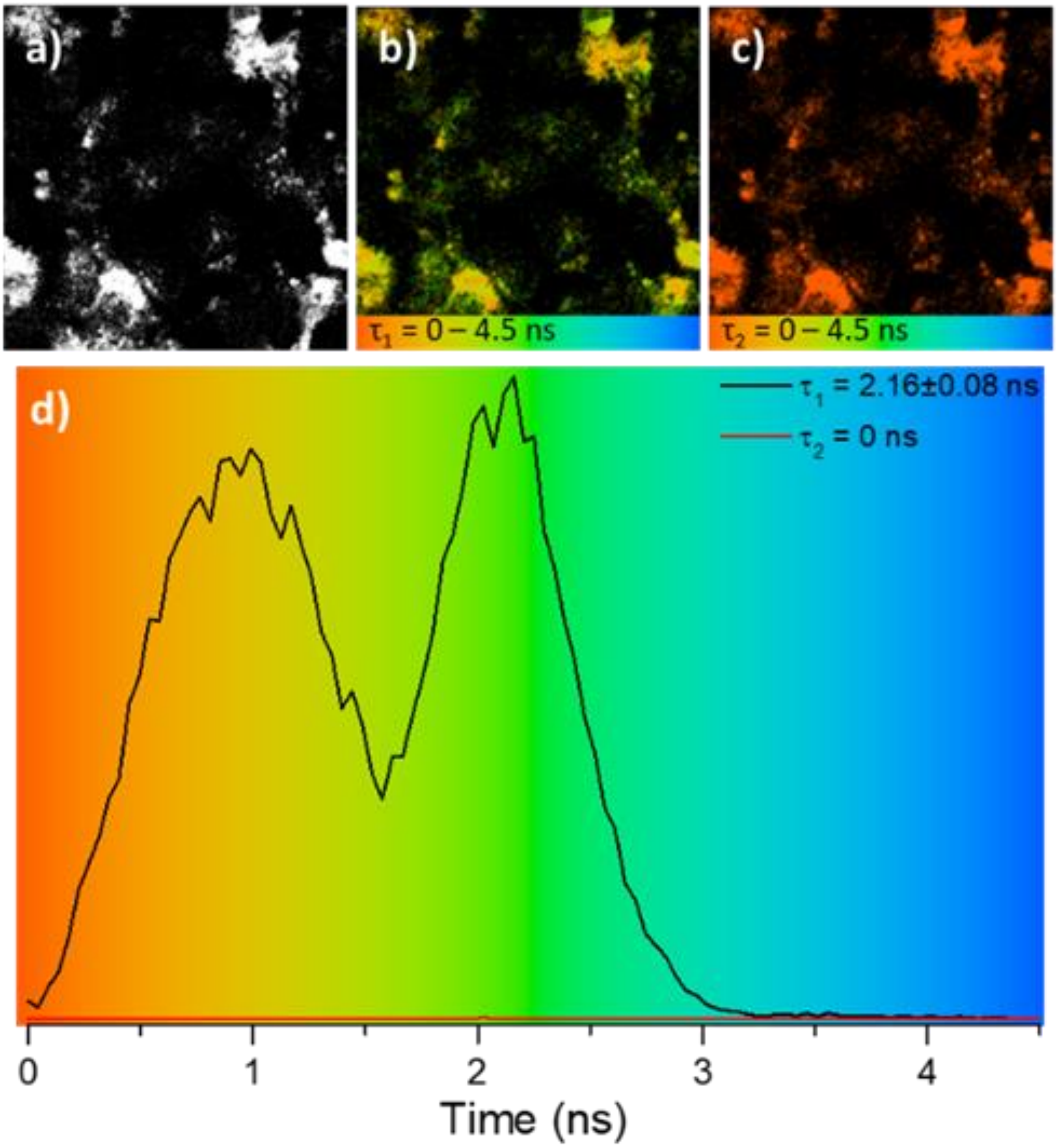

Figure S117. Two-photon fluorescence lifetime imaging in A431 cells line of compound ***L*-3** (100 µM in 1:99 DMSO:serum-free medium) including a) intensity map, b) and c) lifetime maps in coloured code and d) lifetime distribution. Field of view was 100 µm, λ_{ex}=810 nm, laser power 5.0 mW, acquisition time 90 s.

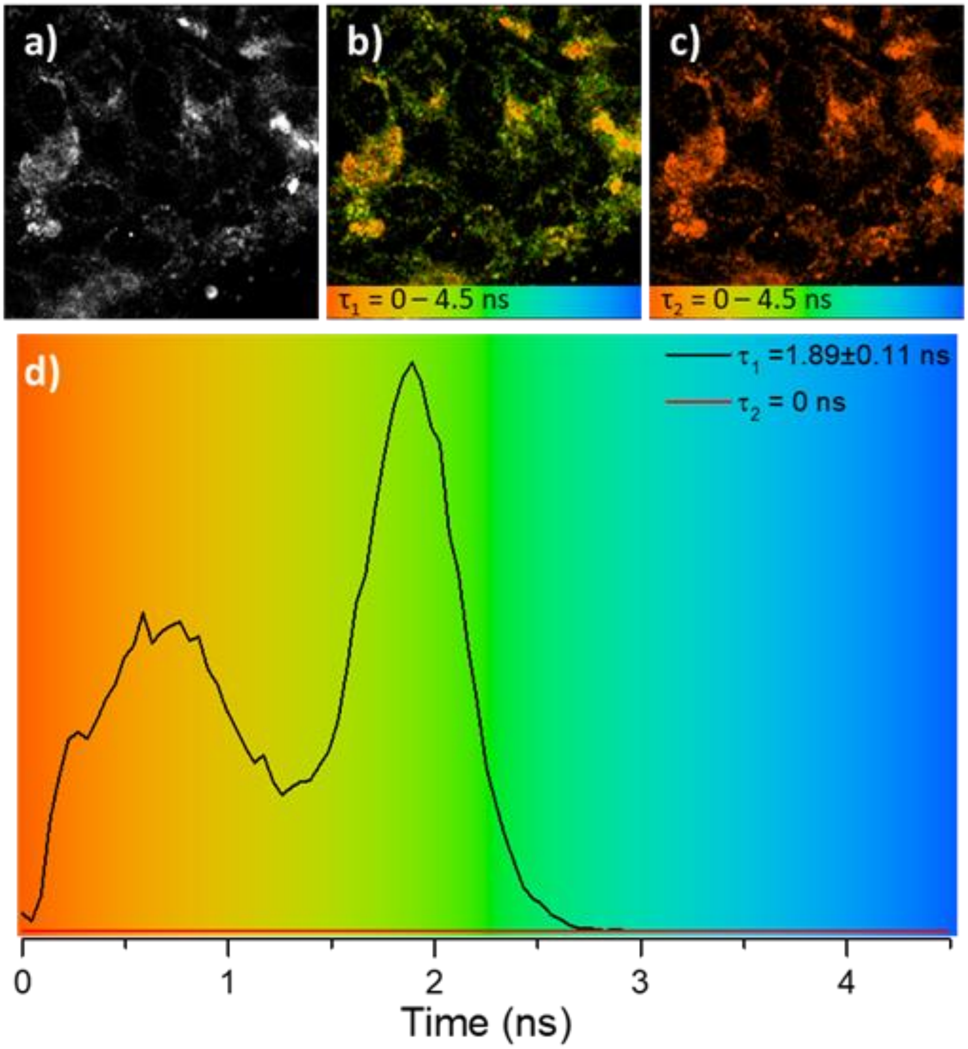

Figure S118. Two-photon fluorescence lifetime imaging in A431 cells line of compound ***L*-4** (100 µM in 1:99 DMSO:serum-free medium) including a) intensity map, b) and c) lifetime maps in coloured code and d) lifetime distribution. Field of view was 100 µm, λ_{ex}=810 nm, laser power 4.7 mW, acquisition time 60 s.

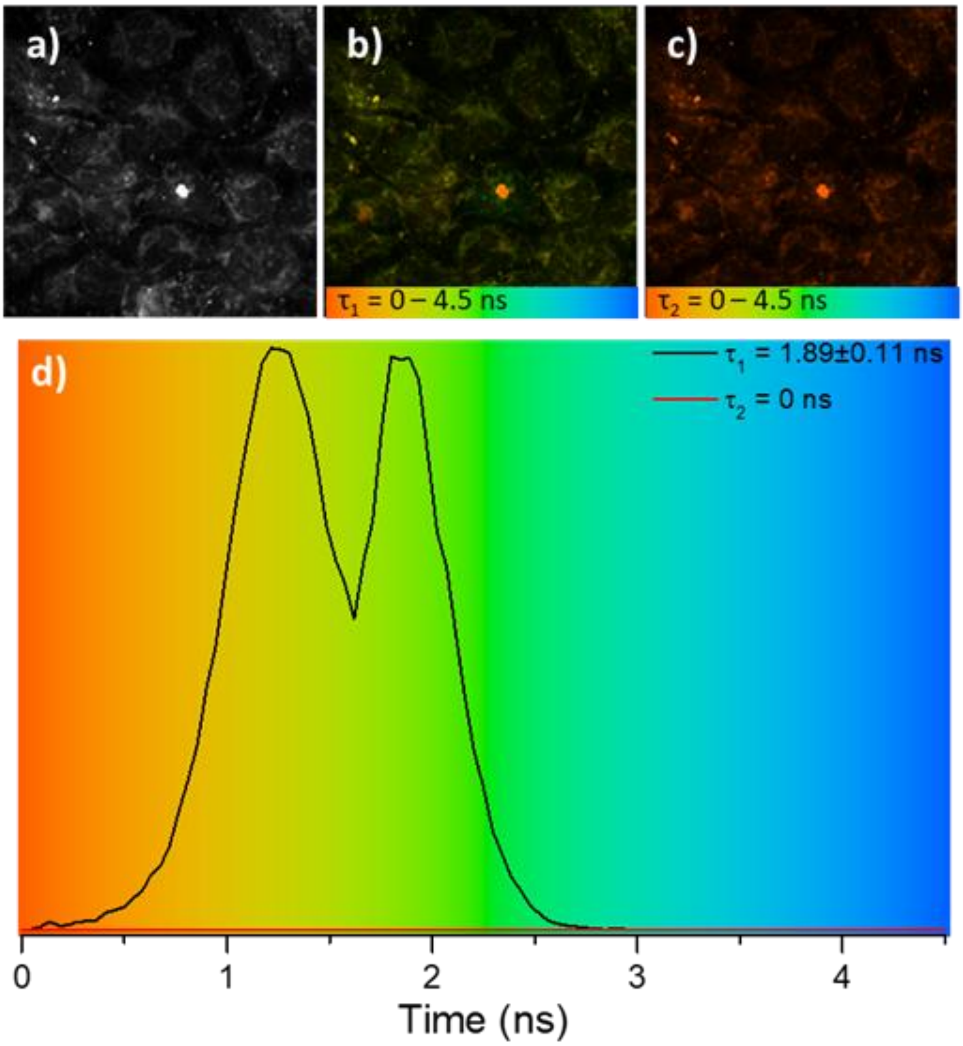

Figure S119. Two-photon fluorescence lifetime imaging in A431 cells line of compound ***D*-3** (100 μM in 1:99 DMSO:serum-free medium) including a) intensity map, b) and c) lifetime maps in coloured code and d) lifetime distribution. Field of view was 100 μm, λ_{ex}=810 nm. laser power 4.9 mW, acquisition time 60 s.

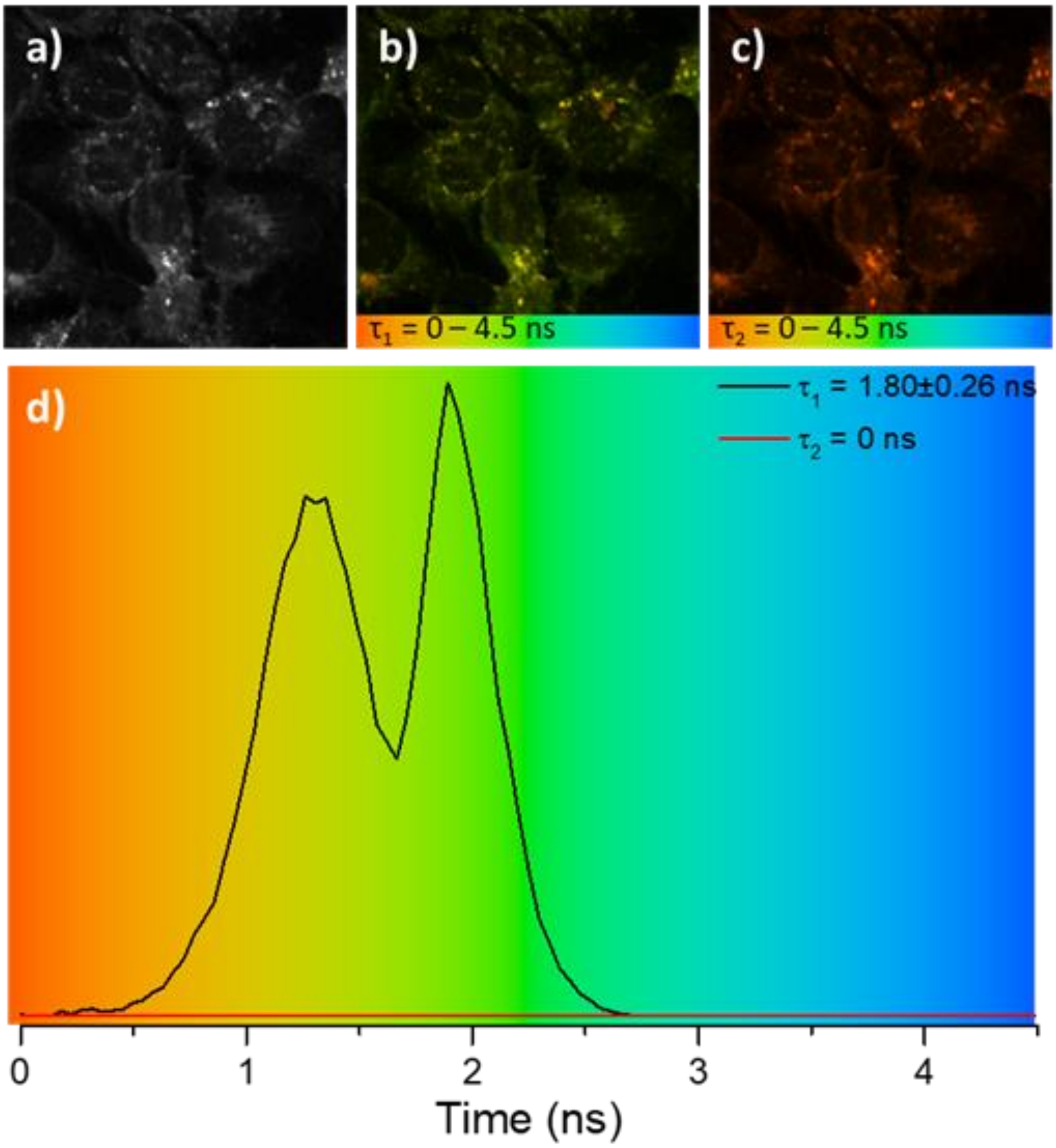

Figure S120. Two-photon fluorescence lifetime imaging in A431 cells line of compound ***D*-4** (100 µM in 1:99 DMSO:serum-free medium) including a) intensity map, b) and c) lifetime maps in coloured code and d) lifetime distribution. Field of view was 100 µm, λ_{ex}=810 nm, laser power, 4.9 mW, acquisition time 90 s.

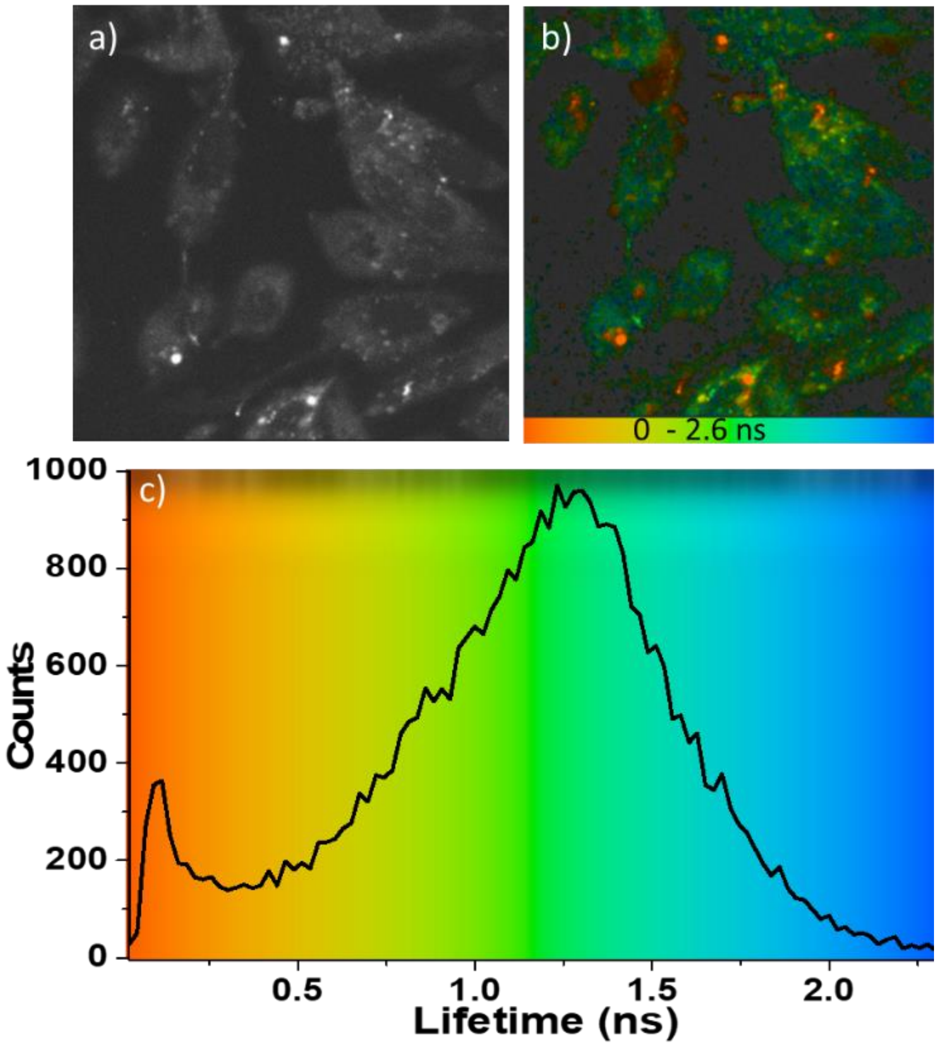

Figure S121. Two-photon fluorescence lifetime imaging in PC-3 cells line of compound ***L-3*** for 20 min (100 µM in 1:99 DMSO:serum-free medium) including $a_{1,2}$) intensity map, $b_{1,2}$) lifetime maps in coloured code and lifetime distribution. a_1-b_1) τ_1=2.66±0.04 ns and a_2-b_2) τ_2=0.00±0.00 ns. λ_{ex}=810 nm; laser power 6.0 mW; acquisition time 40 s; field of view 100 µm.

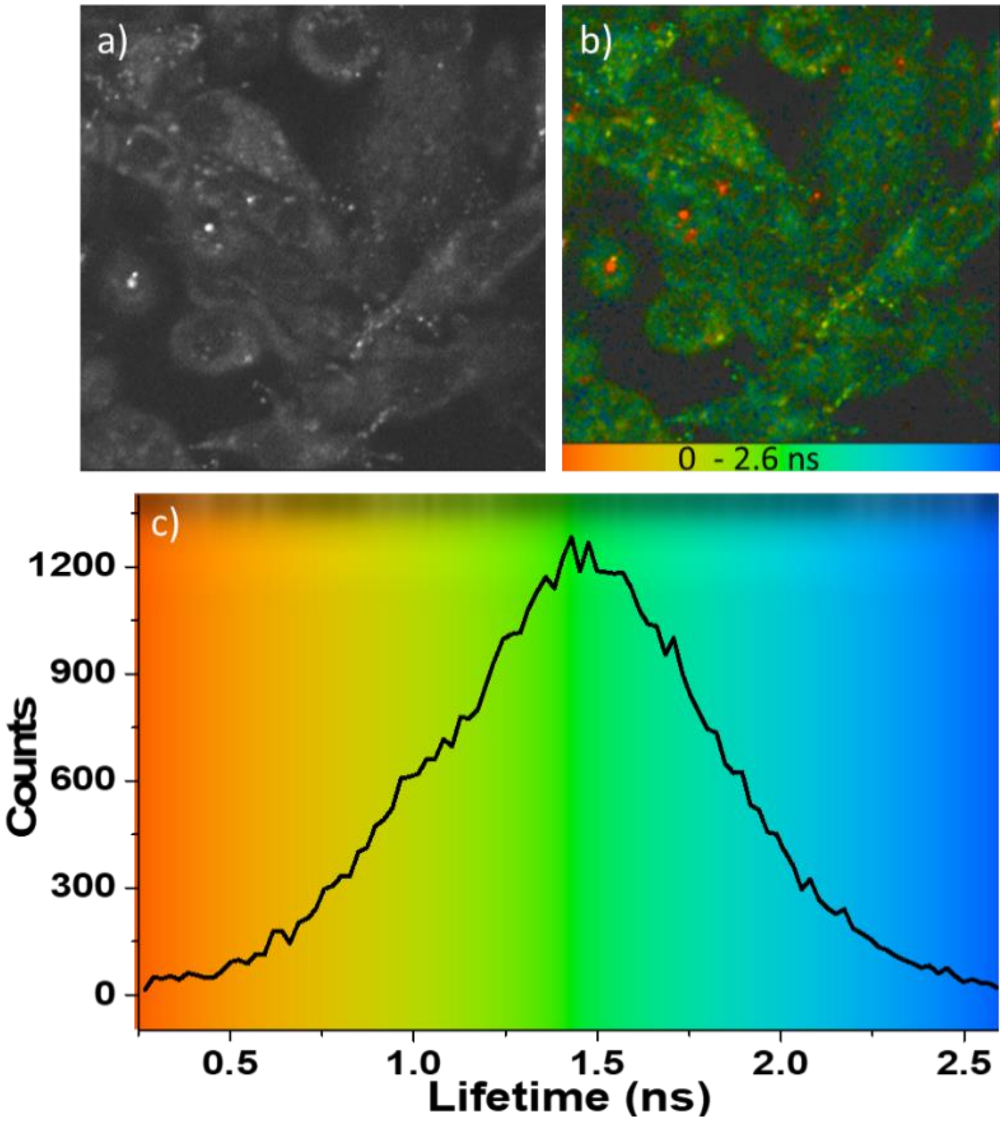

Figure S122. Two-photon fluorescence lifetime imaging in PC-3 cells line of compound ***L-3*** for 20 min (100 µM in 1:99 DMSO:serum-free medium) including $a_{1,2}$) intensity map, $b_{1,2}$) lifetime maps in coloured code and lifetime distribution. a_1-b_1) τ_1=2.66±0.04 ns and a_2-b_2) τ_2=0.00±0.00 ns. λ_{ex}=810 nm; laser power 6.0 mW; acquisition time 40 s, field of view 100 µm..

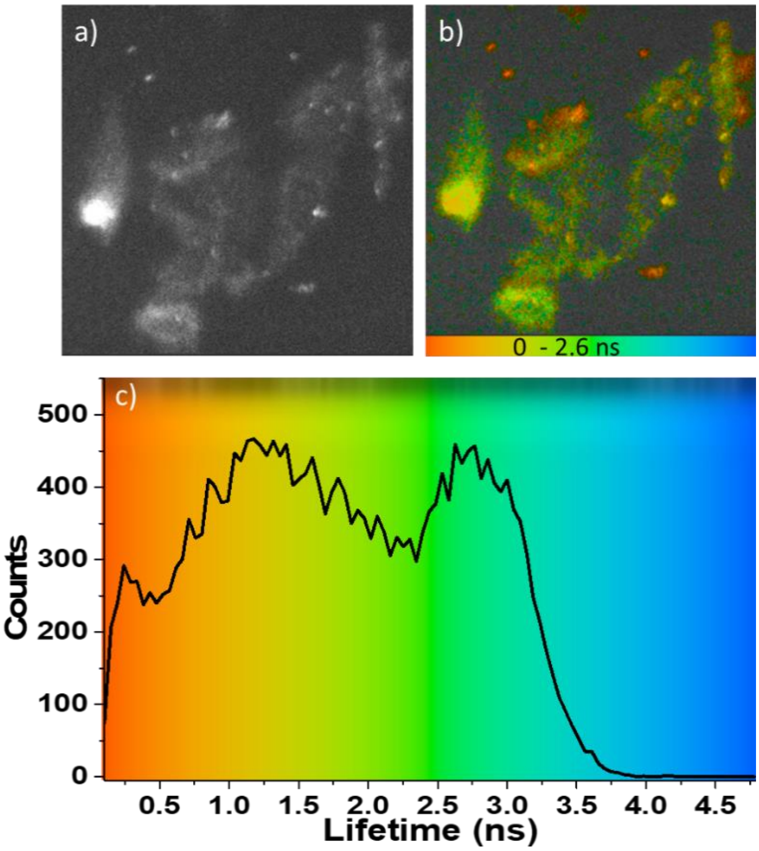

Figure S123. Two-photon fluorescence lifetime imaging in PC-3 cells line of compound ***L-3*** for 18 h (100 µM in 1:99 DMSO:serum-free medium) including $a_{1,2}$) intensity map, $b_{1,2}$) lifetime maps in coloured code and lifetime distribution. a_1-b_1) τ_1=2.66±0.04 ns and a_2-b_2) τ_2=0.00±0.00 ns. λ_{ex}=810 nm; laser power 6.0 mW; acquisition time 40 s; field of view 100 µm..

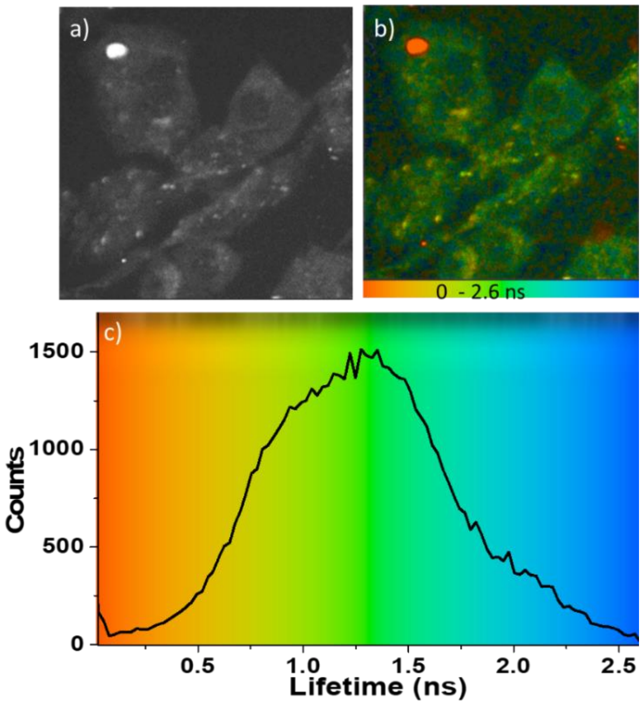

Figure S124. Two-photon fluorescence lifetime imaging in PC-3 cells line of compound ***L-4*** for 20 min (100 µM in 1:99 DMSO:serum-free medium) including $a_{1,2}$) intensity map, $b_{1,2}$) lifetime maps in coloured code and lifetime distribution. a_1-b_1) τ_1=2.66±0.04 ns and a_2-b_2) τ_2=0.00±0.00 ns. λ_{ex}=810 nm; laser power 6.0 mW; acquisition time 40 s; field of view 100 µm..

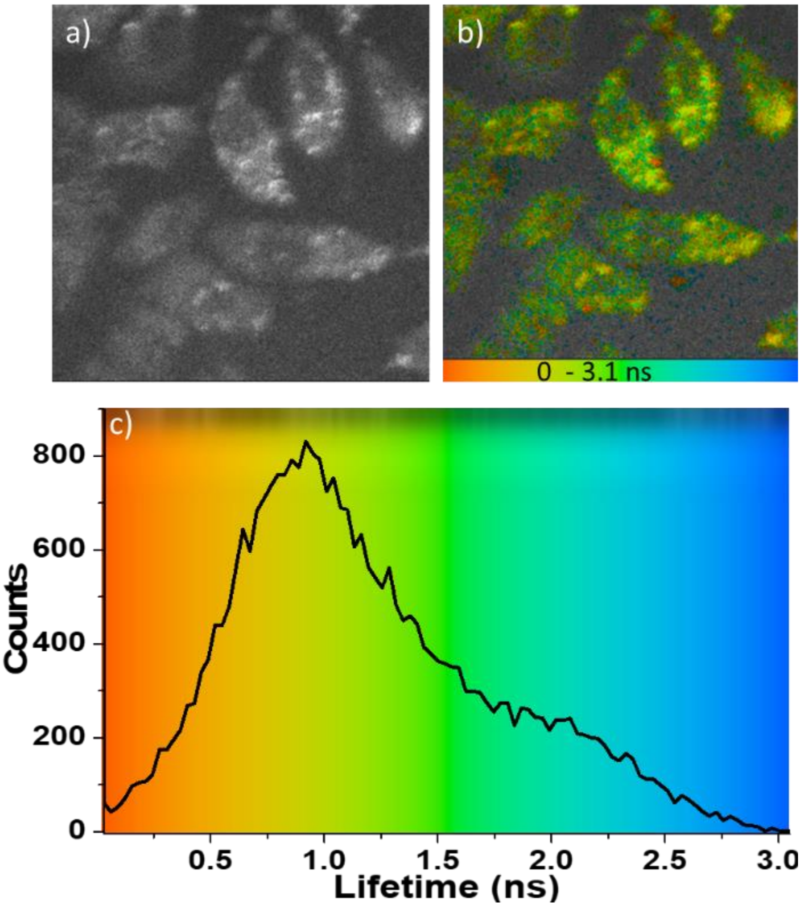

Figure S125. Two-photon fluorescence lifetime imaging in PC-3 cells line of compound ***L-4*** for 60 min (100 µM in 1:99 DMSO:serum-free medium) including $a_{1,2}$) intensity map, $b_{1,2}$) lifetime maps in coloured code and lifetime distribution. a_1-b_1) τ_1=2.66±0.04 ns and a_2-b_2) τ_2=0.00±0.00 ns. λ_{ex}=810 nm; laser power 6.0 mW; acquisition time 40 s; field of view 100 µm..

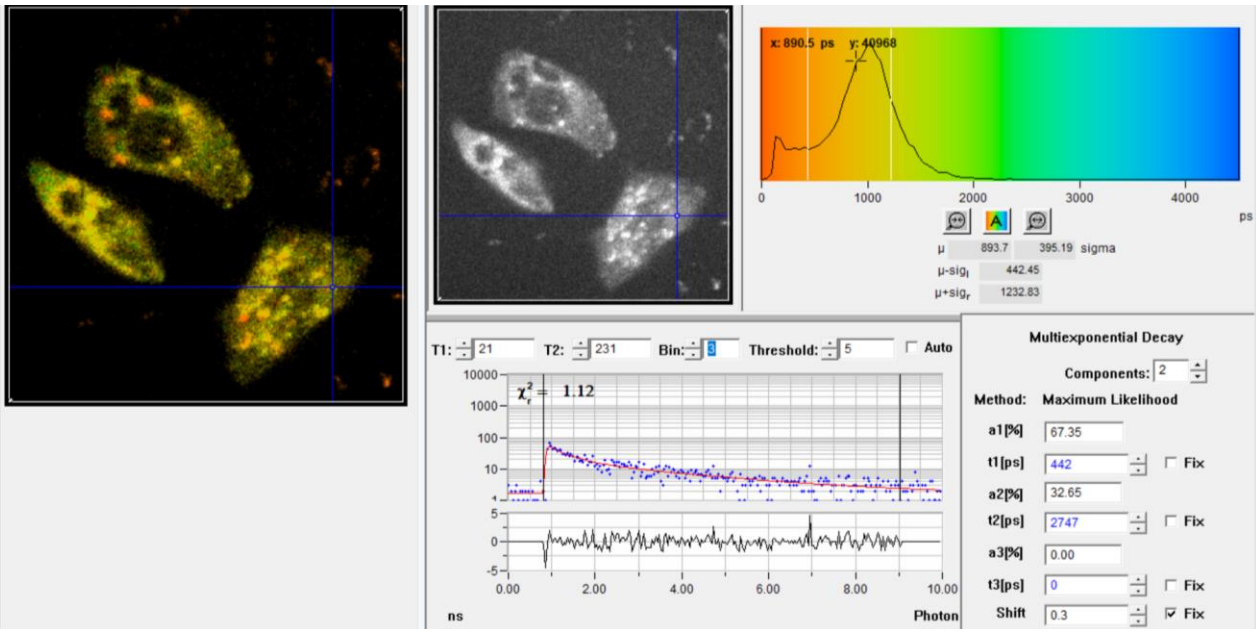

Figure S126. Two-photon fluorescence lifetime imaging in PC-3 cells line of ***D-L*** bis-substituted compound ***L-NDI-D-BBS*** **(**100 µM in 1:99 DMSO:serum-free medium, 16 h incubation time at 37 °C) including lifetime maps in coloured code and lifetime distribution, intensity map and TCSPC lifettie spectrum in a random spot; λ_{ex}=810 nm; laser power 6.0 mW; acquisition time 40 s; field of view 50 µm.

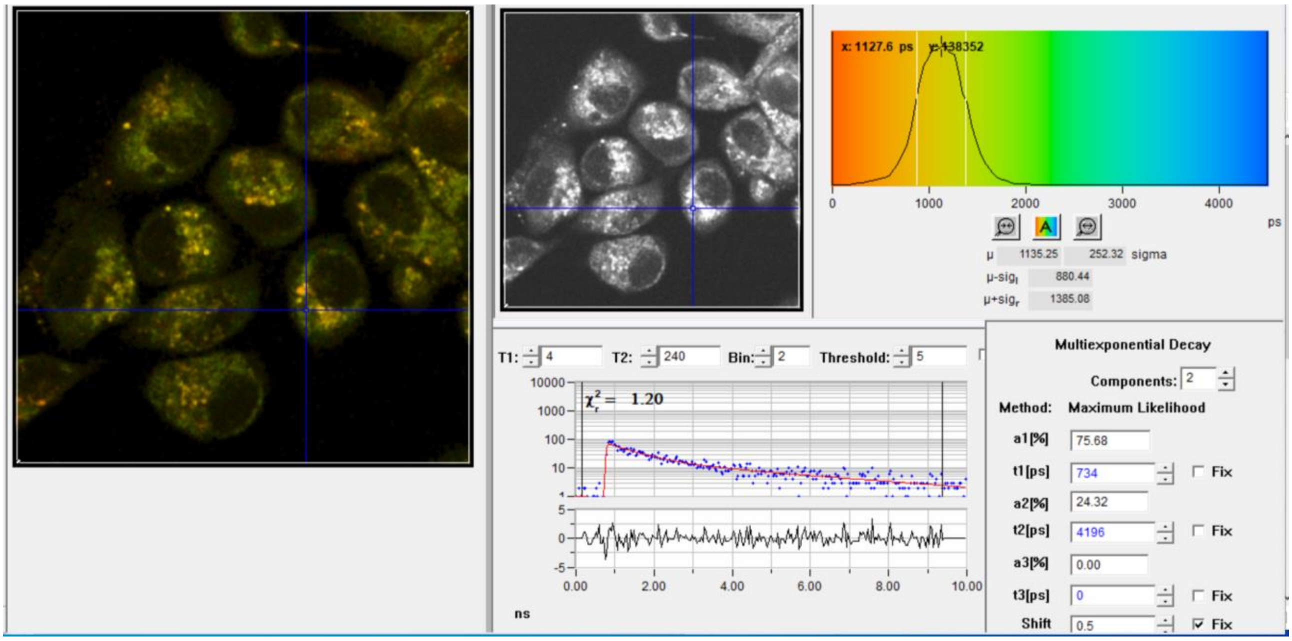

Figure S127. Two-photon fluorescence lifetime imaging in PC-3 cells line of the mono-substituted ***D-L*** compound ***L-NDI-D-BBS*** (100 µM in 1:99 DMSO:serum-free medium, 16 h incubation time at 37 °C) including lifetime maps in coloured code and lifetime distribution, intensity map and TCSPC lifettie spectrum in a random spot. λ_{ex}=810 nm, laser power 3.5 mW, acquisition time 40 s; field of view 100 µm.

Alternative PC-3 uptake images and timeline comparisons:

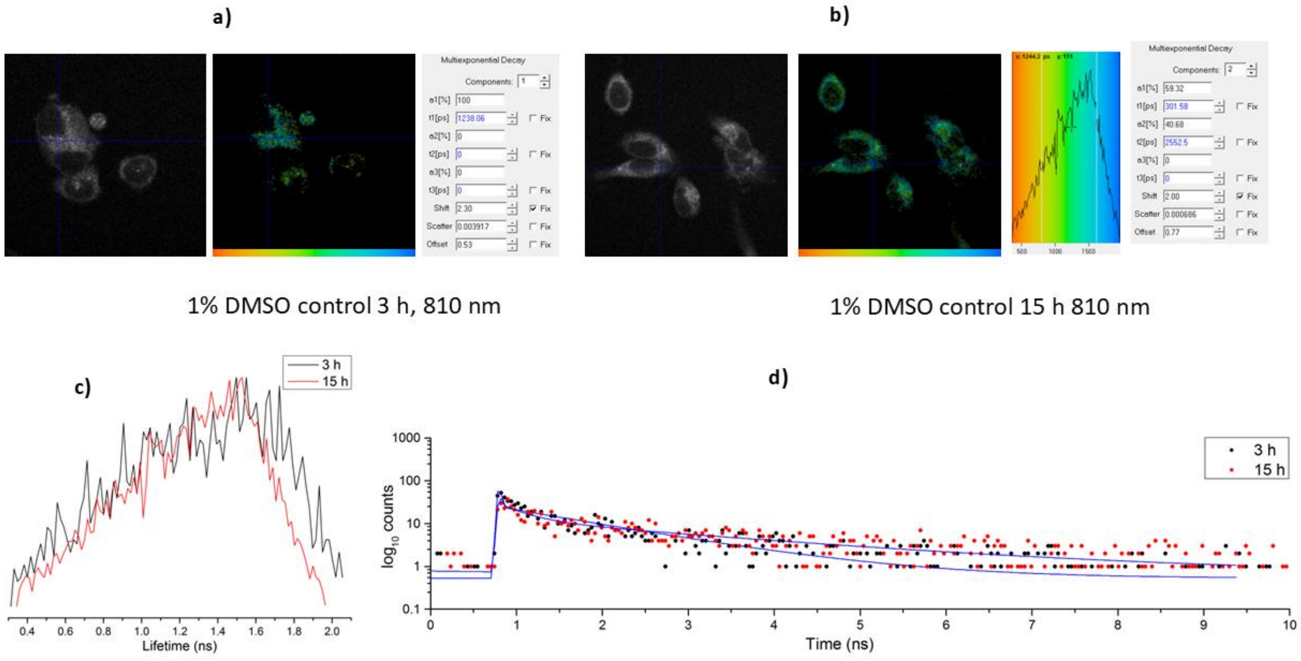

Figure S128. Control experiments to evaluate autofluorescence with time: two-photon fluorescence lifetime imaging in PC-3 cells line under λ_{ex}=810 nm excitation, 1:99 DMSO:serum-free medium including: a) intensity map and corresponding lifetime maps in coloured code and lifetime distribution under 3 h incubation at 37 ^{o}C; b) intensity map and corresponding lifetime maps in coloured code; c) overlay of lifetime distribution under 3 h and 15 h incubation at 37 ^{o}C; d) overlay of TCSPC in random spots in cytoplasm, τ_1=1.23 ns (100%, 3 h) τ_1=0.6 ns (59 %) and τ_2=2.55 (41%).

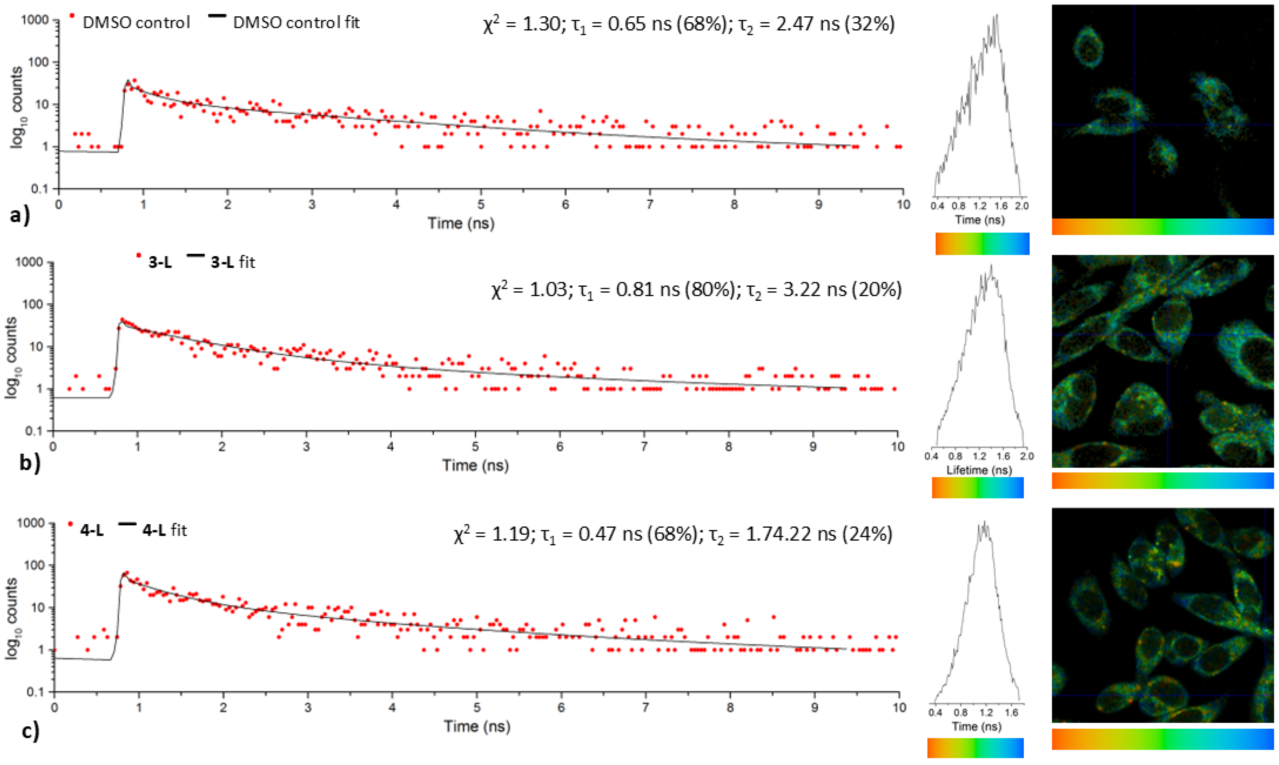

Figure S129. Two-photon fluorescence lifetime imaging in PC-3 cells line using λ_{ex}=810 nm excitation for (a): control (1:99 DMSO:serum-free medium); (b) ***L*-3** (100 µM in 1:99 DMSO:serum-free medium) and (c) ***L*-4** (100 µM in 1:99 DMSO:serum-free medium), including, from left to right: point decay lifetime spectra (TCSPC) measurements in random spots in

cytoplasm, lifetime distributions under 16 h incubation at 37 °C, and lifetime map in coloured code (scale: 0 – 2 ns)

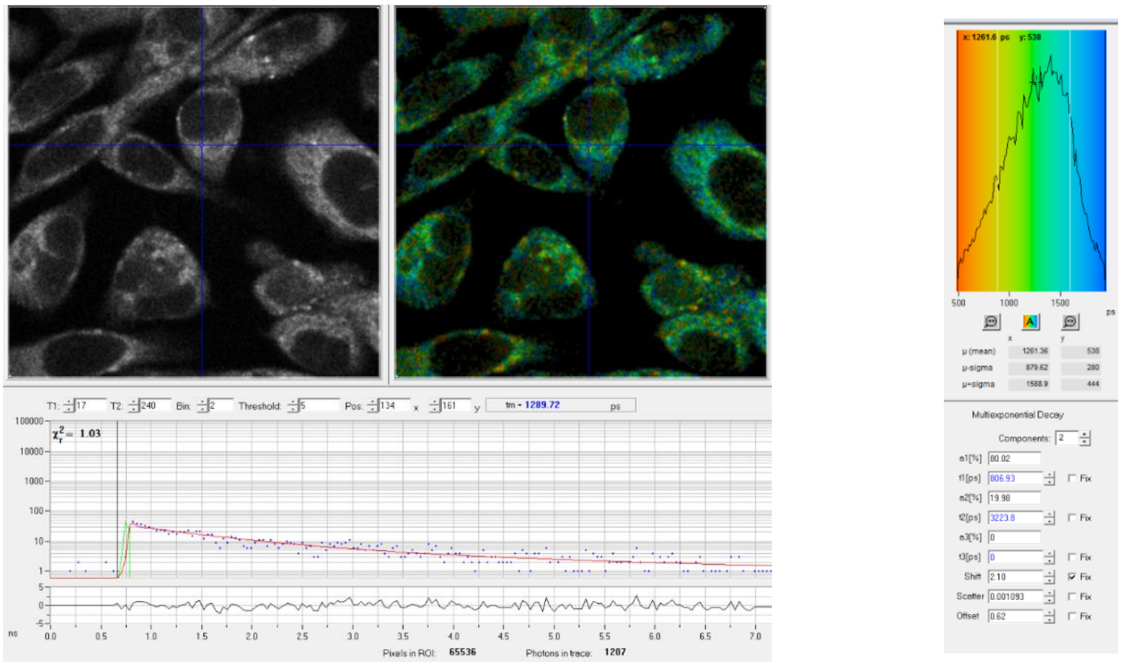

Figure S130. Two-photon fluorescence lifetime imaging in PC-3 cells line using λ_{ex}=810 nm excitation for ***L*-3** (100 µM in 1:99 DMSO:serum-free medium, including clockwise: intensity emission, lifetime map in rainbow-coloured code (scale: 0 – 2 ns), lifetime distributions under 16 h incubation at 37 °C, and point decay lifetime spectra (TCSPC) measurements in a random spot in cytoplasm (laser power: 4.9 mW, field of view 100 µm.).

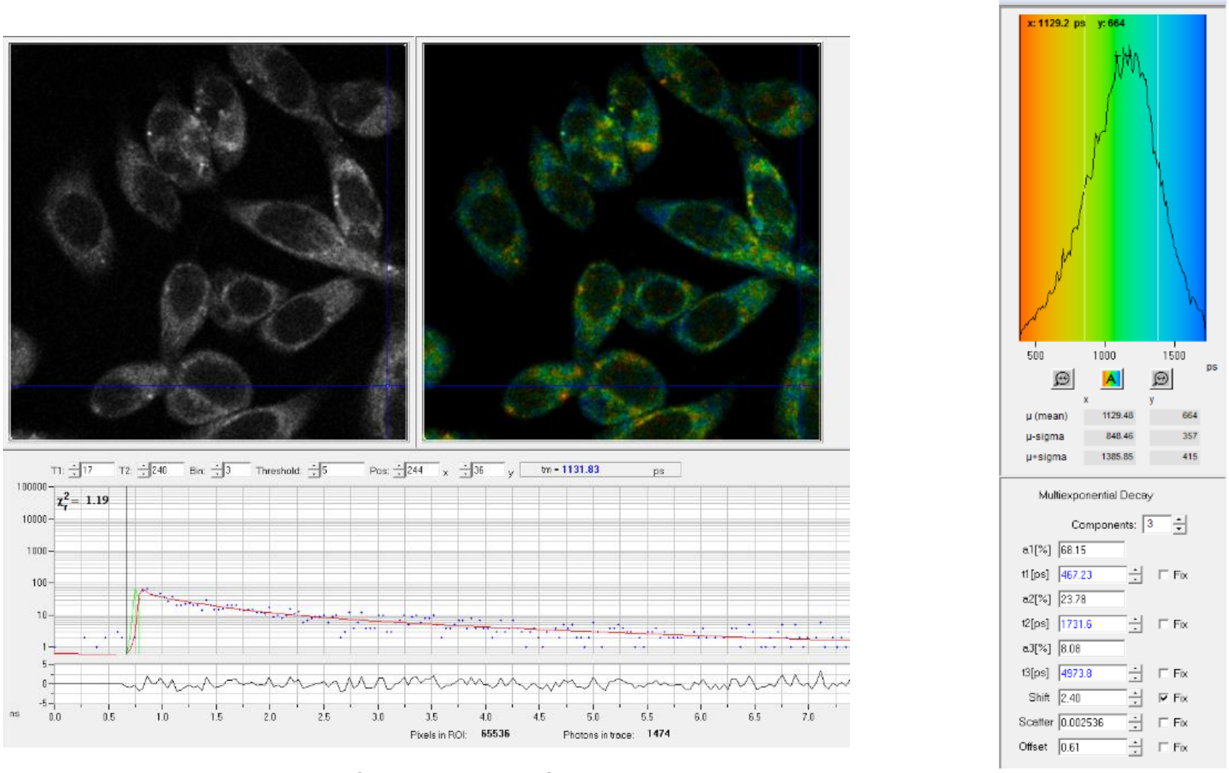

Figure S131. Two-photon fluorescence lifetime imaging in PC-3 cells line using λ_{ex}=810 nm excitation for ***L*-4** (100 µM in 1:99 DMSO:serum-free medium, including clockwise: intensity emission, lifetime map in rainbow-coloured code (scale: 0 – 2 ns), lifetime distributions under 16 h incubation at 37 °C, and point decay lifetime spectra (TCSPC) measurements in a random spot in cytoplasm (laser power: 3.9 mW, field of view 100 µm.).

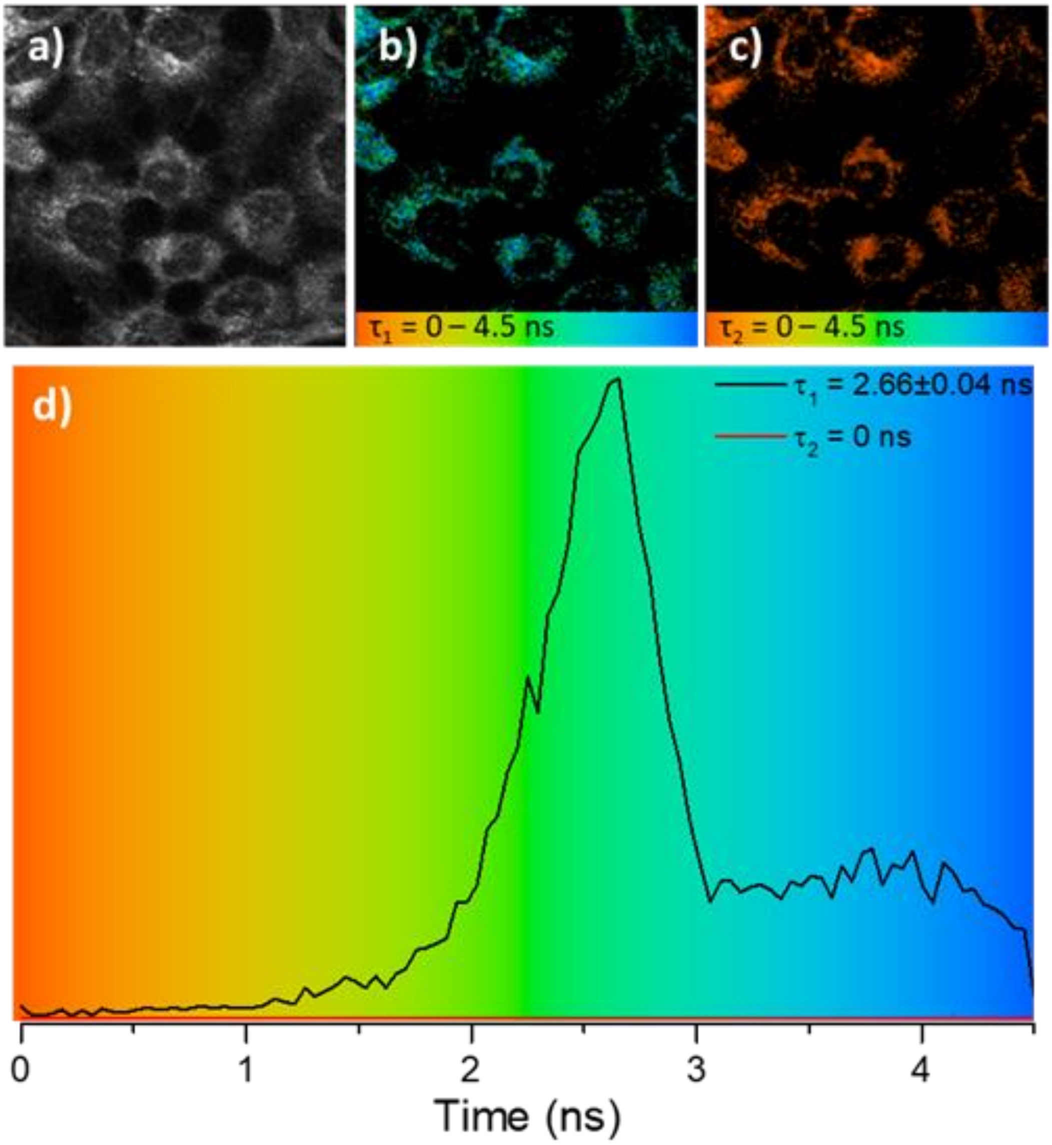

Figure S132. Two-photon fluorescence lifetime imaging in A431 cells line of compound ***L*-6** (50 µM in 1:99 DMSO:serum-free medium) including a) 2P emission intensity map, b) and c) lifetime maps in coloured code lifetime range (0 – 4.5 ns) and d) lifetime distribution. λ_{ex}=910 nm; laser power 1.2 mW; acquisition time 40 s; field of view 100 µm.

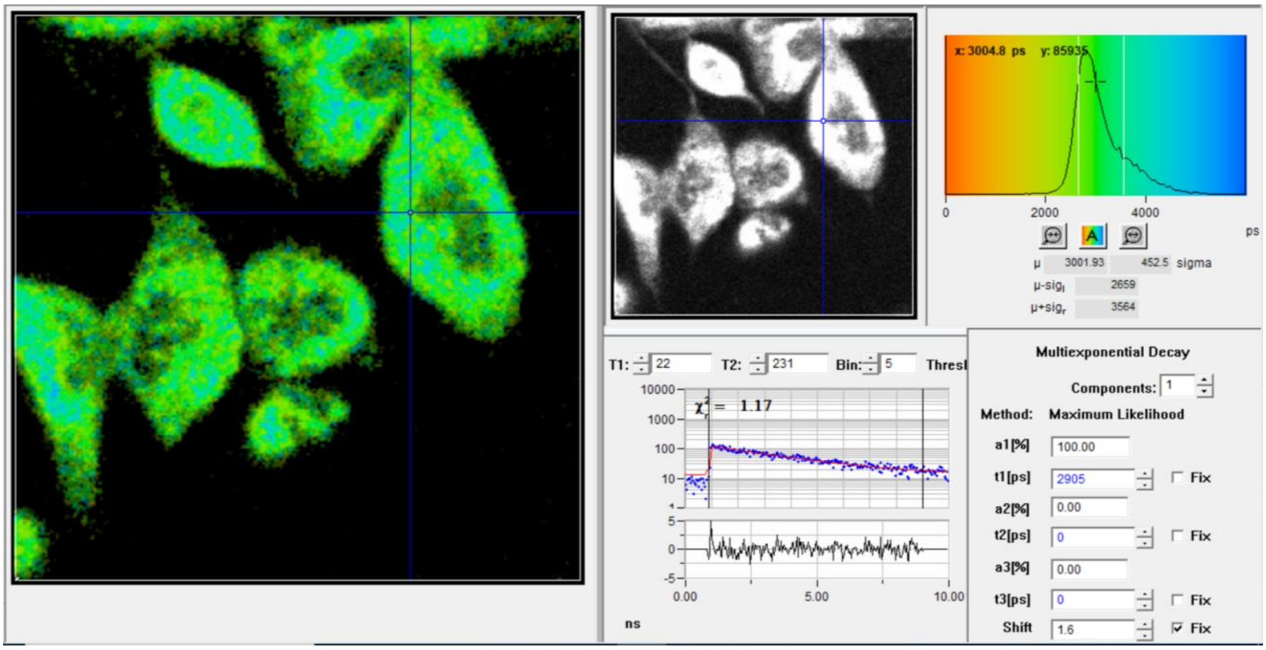

Figure S133. Two-photon fluorescence lifetime imaging in PC3 cells line stained with ER Tracker including lifetime maps in coloured coded lifetime range (0 – 4.5 ns), 2P emission intensity, lifetime distribution. λ_{ex}=810 nm, laser power 3.0 mW; acquisition time 40 s; field of view 50 µm.

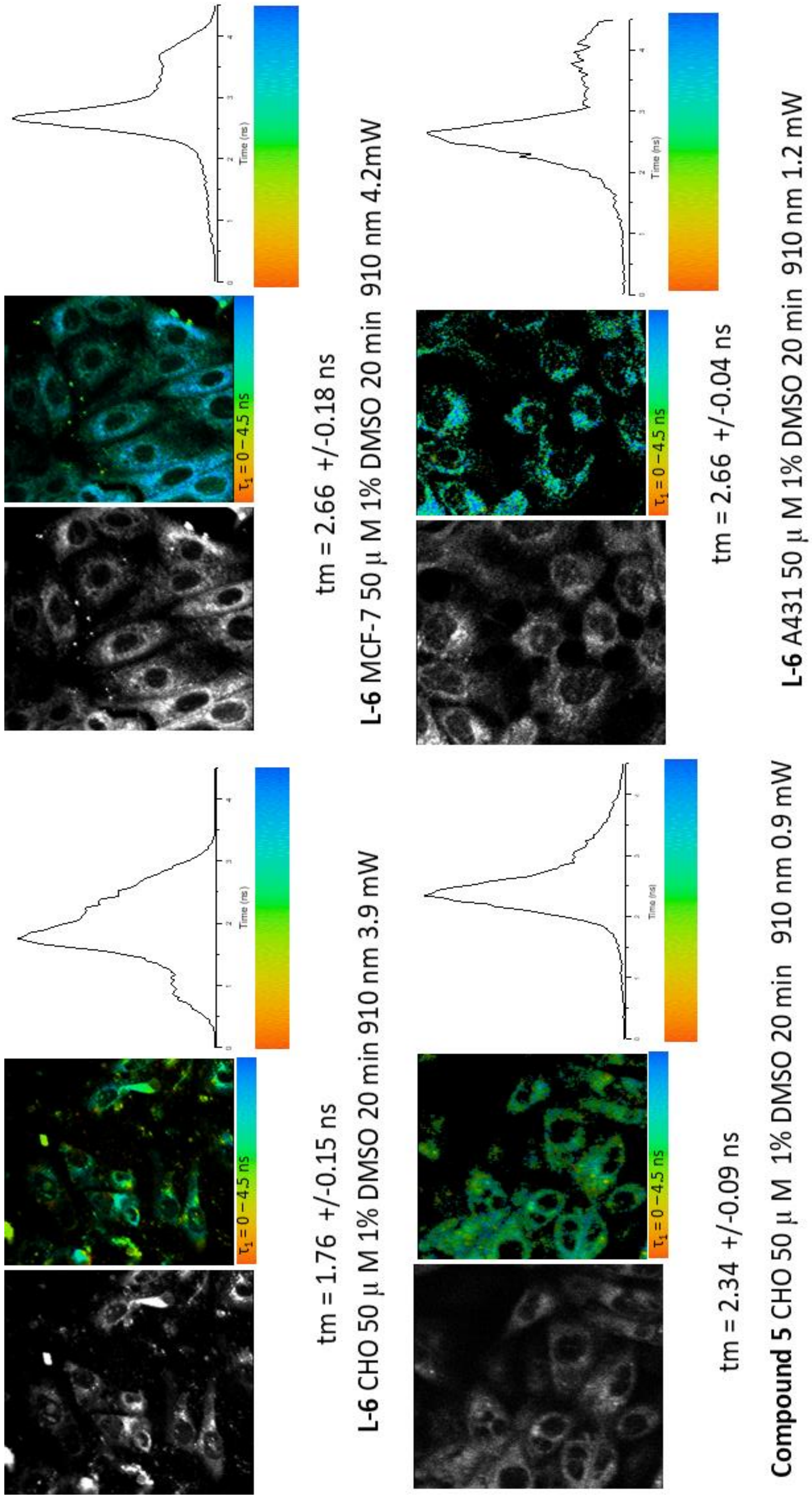

Figure S134. Two-photon fluorescence lifetime imaging in a range of cells lines and conditions of compound ***L*-6** (50 μM in 1:99 DMSO:serum-free medium) including, in each case, 2P emission intensity, lifetime distribution maps in coloured coded scale and corresponding lifetime distribution. λ_{ex}=910 nm. A comparison with the similar parameters for Compound **5** in healthy cells (CHO) is included, and the ER Tracker staining experiment is given in Figure S133. Fields of view 100 μm.

References:

1. A. M. Brouwer, *Pure Appl. Chem.*, 2011, **83**, 2213-2228;
2. A. T. R. Williams, S. A. Winfield and J. N. Miller, *The Analyst*, 1983, **108**, 1067.
3. Z. Hu, R. L. Arrowsmith, J. A. Tyson, V. Mirabello, H. Ge, I. M. Eggleston, S. W. Botchway, G. Dan Pantos and S. I. Pascu, *Chem. Commun.*, 2015, **51**, 6901-6904.
4. R. Markwalder and J. C. Reubi, *Cancer Res.*, 1999, **59**, 1152-1159.
